# Statistical Inference for a Two‐Stage Adaptive Seamless Design Using Different Binary Endpoints

**DOI:** 10.1002/sim.70003

**Published:** 2025-03-06

**Authors:** Ryota Ishii, Kenichi Takahashi, Kazushi Maruo, Masahiko Gosho

**Affiliations:** ^1^ Department of Biostatistics, Institute of Medicine University of Tsukuba Ibaraki Japan; ^2^ Japan Development, MSD K. K. Tokyo Japan

**Keywords:** bias adjustment, confidence interval, seamless phase II/III design, short‐term endpoint

## Abstract

Adaptive seamless design, which integrates phases II and III into a single trial comprising two stages, is garnering increasing interest in the efficient drug development. The first stage involves selecting promising treatment group(s), followed by comparing the efficacy between the selected and control groups in the second stage. This study focused on a two‐stage adaptive seamless design where treatment selection is based on a short‐term binary endpoint, while the comparison is based on a long‐term binary endpoint. Recently, exact and mid‐p tests were proposed in this setting. However, treatment effects at the second stage were estimated using the conventional maximum likelihood estimator (MLE), leading to upward bias owing to treatment selection. We propose the conditional mean‐adjusted estimator (CMAE) and uniformly minimum variance conditional unbiased estimator (UMVCUE) to address the bias in this setting. Additionally, confidence intervals for exact and mid‐p tests were constructed using the Clopper‐Pearson method. Simulation studies were performed to compare the six inference methods defined by combinations of the three estimators and two statistical tests. The simulation results showed that MLE of the treatment effect at the second stage exhibited a notable bias, while CMAE and UMVCUE substantially reduced the bias. The exact test was conservative in terms of the type‐I error rate of the comparison at the second stage, while the mid‐p test yielded results close to the nominal level. In conclusion, we recommend statistical inferences based on the CMAE + mid‐p test or UMVCUE + mid‐p test in our setting.

## 1 Introduction

An efficient drug development plan is required to provide effective and safe drugs to patients because the time and cost of drug development are enormous. An adaptive design is an efficient clinical study design that allows for prospectively planned modifications of a design element based on accumulating data from subjects in an ongoing study without compromising the validity and integrity of the study [1, 2, 3]. Unlike conventional clinical studies, an adaptive design can reconsider a design element using information obtained during the study, which is expected to increase the probability of success. Clinical studies incorporating adaptive design are on the rise, especially since the issuance of guidelines by the European Medicines Agency and the Food and Drug Administration [2, 3].

The adaptive seamless design integrates objectives typically addressed through individual studies, facilitating a seamless transition between them (e.g., Phase II and III) [4]. This design typically comprises two stages. Usually, stage 1 entails a randomized, parallel‐group comparative study comprising multiple treatment and control groups. Its primary objective mirrors that of conventional Phase II trials, focusing on selecting the most promising treatment group(s). The study may be terminated prematurely due to either efficacy or lack‐of‐benefit. Early termination is warranted if no promising treatment group emerges or if a particular treatment group demonstrates clear superiority to the control group in the interim analysis. In stage 2, the enrollment of additional subjects is limited to the selected treatment and control groups. The primary objective of stage 2 is to compare the treatment effect between the selected treatment and control groups. After completing stage 2, a final analysis is performed on the data from subjects enrolled in stages 1 and 2. This design has advantages over conventional drug development, such as a shorter development timeline, fewer total number of subjects, and long‐term data collection.

If conducting the interim analysis promptly is preferred, a more rapidly observable endpoint (short‐term endpoint) can be utilized in the interim analysis [5, 6]. That is, the interim analysis is conducted using the short‐term endpoint, while the final analysis relies on the primary (long‐term) endpoint. This study focuses on seamless phase II/III trials with an interim analysis using the short‐term endpoint.

In the presence of a treatment selection, a conventional treatment effect estimator in the final analysis, such as the maximum likelihood estimator (MLE), has a bias conditional on the treatment selection in the interim analysis [7, 8]. Furthermore, in the final analysis, the type‐I error rate of comparison between selected treatment and control groups exceeds the nominal level. Several statistical tests for the final analysis were proposed in this setting [5, 9, 10, 11]. In particular, Friede et al. [12] proposed a combination test that handles different endpoints by combining p values in the interim and final analyses. This combination test can be widely used for any type of endpoint under the assumption that test statistics follow a normal distribution. However, its performance would decrease when the normality cannot be ensured; for example, when the endpoints are binary responses of rare events and the sample size is small.

Recently, in a setting where both short‐ and long‐term endpoints are binary, Takahashi et al. [13] proposed the exact and mid‐p tests that do not assume normality and demonstrated that their proposed tests are better than the combination test. However, three problems need to be solved before implementing their tests. First, Takahashi et al. used MLE as a treatment effect estimator in the final analysis; thus, its bias adjustments and the development of an unbiased estimator in this setting remain unsolved. Second, in some scenarios, the mid‐p test inflates the type‐I error rate of the comparison in the final analysis owing to the bias of MLE. Hence, the exact test leading to lower statistical power should be used to avoid inflation. The third problem is that Takahashi et al. did not consider confidence intervals for the treatment effect. Hence, we additionally assume that the short‐ and long‐term endpoints are binary and aim to develop a valid statistical inference by constructing an adjusted estimator and confidence interval.

Many researchers have studied bias adjustment in several adaptive design settings [14, 15, 16, 17, 18]. Additionally, Robertson et al. [8] reviewed these adjusted estimators. In our setting, three approaches can be considered to avoid bias. The first involves estimating the conditional bias of the MLE and subtracting the bias estimator from the MLE [19, 20]. This adjusted estimator is referred to as the conditional mean‐adjusted estimator (CMAE). The second approach is an unbiased estimator based on Rao‐Blackwell's theorem and is referred to as the uniformly minimum variance conditional unbiased estimator (UMVCUE) [16, 21]. The third approach is the estimator defined by maximization of the likelihood function conditioned on the interim analysis results instead of the unconditional likelihood function [19]. Although the third estimator is based on a natural idea, it could lead to an infinite mean absolute error [22], and some simulation studies showed that this estimator overcorrects the bias and increases the mean squared error [22, 23]. Therefore, this study aims to develop CMAE and UMVCUE in the framework of adaptive seamless phase II/III trials with short‐ and long‐term binary endpoints. We also provide confidence intervals corresponding to exact and mid‐p tests proposed by Takahashi et al.

The remainder of this paper is organized as follows. Section 2 reviews the adaptive seamless design and the hypothesis tests proposed by Takahashi et al. Section 3 describes CMAE, UMVCUE, and the confidence interval. The results of the simulation and case studies are presented in Sections 4 and 5, respectively. The R code for our simulation and case studies is available on GitHub (https://github.com/rtishii). Finally, Section 6 discusses the results and concludes the paper.

## 2 Hypothesis Test

### 2.1 Design Outline and Notation

Our settings and notation are the same as Takahashi et al. [13]. We assume a seamless phase II/III trial with an interim analysis. In stage 1, the subjects are randomized into G treatment groups or a control group. Let g be a group index and let g=0 and g=1,…,G represent the control and treatment groups, respectively. We assume that the short‐term endpoint for $N_{g}^{\left(1\right)}$ (g=0,1,…,G) subjects is used in the interim analysis, regardless of the collection of long‐term endpoint data. Let $X_{g}^{\left(1\right)}$ (g=0,1,…,G) denote the number of responses for the short‐term endpoint from $N_{g}^{\left(1\right)}$ subjects that follows a binomial distribution $\mathrm{Bin}(N_{g}^{\left(1\right)},\xi_{g})$. In the interim analysis, treatment selection is performed based on short‐term endpoints; that is, the selected group s∈{1,…,G} is defined as a function Q of $X_{g}^{\left(1\right)}$. For example, if the control group is not used in the interim analysis and the treatment group with the highest response rate is selected, the treatment selection is defined as follows:

(1) $$Q(X_{1}^{\left(1\right)},\ldots ,X_{G}^{\left(1\right)})=\underset{g\in \{1,\ldots ,G\}}{\text{arg max}}X_{g}^{\left(1\right)}/N_{g}^{\left(1\right)}$$

Here, if two or more treatment groups show the highest response rate, the group with the smallest index is assumed to be selected, which means that the treatment with the lowest dose is selected for safety. Furthermore, if early stopping due to lack‐of‐benefit needs to be included in the interim analysis, the function Q can be extended as

$$Q(X_{0}^{\left(1\right)},X_{1}^{\left(1\right)},\ldots ,X_{G}^{\left(1\right)})=\underset{g\in \{0,1,\ldots ,G\}}{\text{arg max}}X_{g}^{\left(1\right)}/N_{g}^{\left(1\right)}.$$

In this case, Q=0 means that the highest response rate is observed in the control group and the trial should be stopped early.

After interim analysis, additional data are only collected for the control and selected groups. Let $N_{g}$ be the total number of subjects in group g=0,s and $N_{g}^{\left(2\right)}=N_{g}-N_{g}^{\left(1\right)}$. We further let $Y_{g}^{\left(1\right)}$ and $Y_{g}^{\left(2\right)}$ be the number of responses for the long‐term endpoint from the $N_{g}^{\left(1\right)}$ and $N_{g}^{\left(2\right)}$ subjects, respectively, and $Y_{g}^{\left(j\right)}$ follows a binomial distribution $\mathrm{Bin}(N_{g}^{\left(j\right)},\pi_{g})$ for g=0,s and j=1,2. The correlation coefficient between the short‐ and long‐term endpoints of group g=0,s is denoted by $\rho_{g}$. At the end of stage 2, the final analysis is performed by comparing the long‐term endpoints $Y_{0}=Y_{0}^{\left(1\right)}+Y_{0}^{\left(2\right)}$ and $Y_{s}=Y_{s}^{\left(1\right)}+Y_{s}^{\left(2\right)}$.

### 2.2 Exact and Mid‐p Tests

Exact and mid‐p tests were proposed for group comparison at the final analysis [13]. Let $\Delta_{g}$ be the difference in the log odds between treatment g and the control group (i.e., $\Delta_{g}=\log(\pi_{g}/(1-\pi_{g}))-\log(\pi_{0}/(1-\pi_{0}))$). The null hypothesis is $H_{0}:\Delta_{g}\leq 0$ for all g=1,…,G. The two tests are constructed based on Fisher's exact test, taking treatment selection into account. Let $T=Y_{0}+Y_{s}$ be the total number of responses and let $y_{s}$ and t be the observed values of $Y_{s}$ and T, respectively. Then, the conditional probability of $Y_{s}$ given T and the treatment selection is expressed as

Pr(Ys=ys|Q=s,T=t)=(N0t−ys)hπs(ys)exp(ysΔs)∑k=max{0,t−N0}min{t,Ns}(N0t−k)hπs(k)exp(kΔs),

where

hπs(k)=∑r=max{0,k−Ns(2)}min{k,Ns(1)}(Ns(1)r)(Ns(2)k−r)Pr(Q=s|Ys(1)=r),

for $k=\max\{0,t-N_{0}\},\ldots ,\min\{t,N_{s}\}$. We need to model the joint distribution of $\left(X_{s}^{\left(1\right)},Y_{s}^{\left(1\right)}\right)$ to calculate the conditional probability $\mathrm{Pr}(Q=s|Y_{s}^{\left(1\right)}=r)$. We use the following bivariate binomial distribution introduced by Biswas and Hwang [24]:

(2)  Pr(Xg(j)=x|Yg(j)=y) =(1+αg)−Ng(j)∑z=max{0,x+y−Ng(j)}min{x,y}(yz)(Ng(j)−yx−z) ×(βg+αg)z(1−βg)y−zβgx−z(1−βg+αg)Ng(j)+z−x−y

where

$$\alpha_{g}={\left(\sqrt{\frac{\pi_{g}(1-\pi_{g})}{\xi_{g}(1-\xi_{g})}}-\rho_{g}\right)}^{-1}\rho_{g}$$

is the parameter corresponding to the correlation coefficient and $\beta_{g}=\xi_{g}+\alpha_{g}(\xi_{g}-\pi_{g})$. The one‐sided p values for the exact and mid‐p tests are defined as

(3) $$\underset{\Delta_{s}\leq 0}{\sup}\mathrm{Pr}(Y_{s}\geq y_{s}|Q=s,T=t)$$

and

(4) $$\underset{\Delta_{s}\leq 0}{\sup}\left\{\mathrm{Pr}(Y_{s}>y_{s}|Q=s,T=t)+\frac{1}{2}\mathrm{Pr}(Y_{s}=y_{s}|Q=s,T=t)\right\}$$

respectively. These tests are constructed under the global null hypothesis and control the type‐I error rate in the weak sense [25, 26].

The conditional probability $\mathrm{Pr}(Q=s|Y_{s}^{\left(1\right)}=r)$ depends on the parameter $\left(\xi_{0},\xi_{1},\ldots ,\xi_{G},\pi_{s},\rho_{s}\right)$; thus, the p values are functions of $\left(\xi_{0},\xi_{1},\ldots ,\xi_{G},\pi_{s},\rho_{s}\right)$. Hence, the p values are calculated by substituting the estimates of $\left(\xi_{0},\xi_{1},\ldots ,\xi_{G},\pi_{s},\rho_{s}\right)$. To calculate p values, Takahashi et al. [13] used MLE defined as follows:

$$\begin{array}{ll} {\hat{\xi }}_{\text{MLE},g} & =\frac{X_{g}}{N_{g}},\;{\hat{\pi }}_{\text{MLE},g}=\frac{Y_{g}}{N_{g}},\;\text{for}\;g=0,s,\\ {\hat{\xi }}_{\text{MLE},g} & =\frac{X_{g}^{\left(1\right)}}{N_{g}^{\left(1\right)}}\;\text{for}\;g\neq 0,s, \end{array}$$

and ${\hat{\rho }}_{\text{MLE},s}$ is Pearson's correlation coefficient, where $X_{g}=X_{g}^{\left(1\right)}+X_{g}^{\left(2\right)}$ for g=0,s.

## 3 Adjusted Estimator and Confidence Interval

In this section, we develop adjusted estimators and confidence intervals for the treatment effect at the final analysis in the framework of a seamless phase II/III design using short‐ and long‐term binary endpoints.

### 3.1 Conditional Mean‐Adjusted Estimator

Let $\xi =(\xi_{0},\xi_{1},\ldots ,\xi_{G})$ and ${\hat{\xi }}_{\text{MLE}}=({\hat{\xi }}_{\text{MLE},0},{\hat{\xi }}_{\text{MLE},1},\ldots ,{\hat{\xi }}_{\text{MLE},G})$. The conditional biases of MLE ${\hat{\xi }}_{g}$ in the selected group (g=0,s) and unselected group (g≠0,s) are expressed as

$$\begin{array}{ll} b_{\xi ,g} & =E[{\hat{\xi }}_{\text{MLE},g}|Q=s]-\xi_{g}\\ & =\sum_{k_{x}=0}^{N_{g}^{\left(1\right)}}\frac{k_{x}\mathrm{Pr}(X_{g}^{\left(1\right)}=k_{x})\mathrm{Pr}(Q=s|X_{g}^{\left(1\right)}=k_{x})}{N_{g}\mathrm{Pr}(Q=s)}+\frac{N_{g}^{\left(2\right)}}{N_{g}}\xi_{g}-\xi_{g} \end{array}$$

and

$$\begin{array}{ll} b_{\xi ,g} & =E[{\hat{\xi }}_{\text{MLE},g}|Q=s]-\xi_{g}\\ & =\sum_{k_{x}=0}^{N_{g}^{\left(1\right)}}\frac{k_{x}\mathrm{Pr}(X_{g}^{\left(1\right)}=k_{x})\mathrm{Pr}(Q=s|X_{g}^{\left(1\right)}=k_{x})}{N_{g}\mathrm{Pr}(Q=s)}-\xi_{g}, \end{array}$$

respectively. On the other hand, the conditional bias of MLE ${\hat{\pi }}_{g}$ for g=0,s is expressed as

$$\begin{array}{ll} b_{\pi ,g} & =E[{\hat{\pi }}_{\text{MLE},g}|Q=s]-\pi_{g}\\ & =\sum_{k_{x},k_{y}=0}^{N_{g}^{\left(1\right)}}\frac{k_{y}\mathrm{Pr}(X_{g}^{\left(1\right)}=k_{x},Y_{g}^{\left(1\right)}=k_{y})\mathrm{Pr}(Q=s|X_{g}^{\left(1\right)}=k_{x})}{N_{g}\mathrm{Pr}(Q=s)}\\ & \;+\frac{N_{g}^{\left(2\right)}}{N_{g}}\pi_{g}-\pi_{g}. \end{array}$$

Here, the conditional probability $\mathrm{Pr}(X_{g}^{\left(1\right)}=k_{x}|Y_{g}^{\left(1\right)}=k_{y})$ can be calculated from (2). Hence, the conditional bias can be derived analytically given the selection rule and the selected treatment group. However, it is obvious that biases depend on true parameters, that is, $b_{\xi ,g}=b_{\xi ,g}(\xi )$ and $b_{\pi ,g}=b_{\pi ,g}(\xi ,\pi_{g},\rho_{g})$. Thus, the conditional bias is estimated by substituting MLEs into biases and the CMAEs are defined as follows:

$$\begin{array}{ll} {\hat{\xi }}_{\text{CMAE},g} & ={\hat{\xi }}_{\text{MLE},g}-b_{\xi ,g}({\hat{\xi }}_{\text{MLE}})\\ {\hat{\pi }}_{\text{CMAE},g} & ={\hat{\pi }}_{\text{MLE},g}-b_{\pi ,g}({\hat{\xi }}_{\text{MLE}},{\hat{\pi }}_{\text{MLE},g},{\hat{\rho }}_{\text{MLE},g}). \end{array}$$

The vector of CMAEs is denoted by ${\hat{\xi }}_{\text{CMAE}}=({\hat{\xi }}_{\text{CMAE},0},{\hat{\xi }}_{\text{CMAE},1},\ldots ,{\hat{\xi }}_{\text{CMAE},G})$.

The estimator of $\rho_{g}$ for g=0,s corresponding to CMAE is constructed as follows. Let $\ell (\xi_{g},\pi_{g},\rho_{g})$ be the log‐likelihood function of a bivariate binomial distribution of $\left(X_{g},Y_{g}\right)$. In this study, we use the distribution proposed by Biswas and Hwang [24]. Subsequently, we fix the log‐likelihood function at $\left(\xi_{g},\pi_{g})=({\hat{\xi }}_{\text{CMAE},g},{\hat{\pi }}_{\text{CMAE},g}\right)$ and maximize the function $\ell ({\hat{\xi }}_{\text{CMAE},g},{\hat{\pi }}_{\text{CMAE},g},\rho_{g})$ with respect to $\rho_{g}$. The CMAE of $\rho_{g}$ satisfies the condition

$$\begin{array}{l} \ell ({\hat{\xi }}_{\text{CMAE},g},{\hat{\pi }}_{\text{CMAE},g},{\hat{\rho }}_{\text{CMAE},g})=\underset{\rho_{g}}{\max}\ell ({\hat{\xi }}_{\text{CMAE},g},{\hat{\pi }}_{\text{CMAE},g},\rho_{g}). \end{array}$$

### 3.2 Uniformly Minimum Variance Conditional Unbiased Estimator

Given the result of the interim analysis Q=s, UMVCUE is constructed using an unbiased estimator and sufficient statistics. Clearly, the numbers $X_{g}^{\left(2\right)}$ and $Y_{g}^{\left(2\right)}$ of responses for the short‐ and long‐term endpoints from the $N_{g}^{\left(2\right)}$ subjects are independent of the interim analysis for g=0,s. Hence, $X_{g}^{\left(2\right)}/N_{g}^{\left(2\right)}$ and $Y_{g}^{\left(2\right)}/N_{g}^{\left(2\right)}$ are unbiased estimators of $\xi_{g}$ and $\pi_{g}$ for g=0,s even conditional on Q=s, respectively. Let $Z_{g}^{\left(j\right)}$ be the number of subjects that responded to both short‐ and long‐term endpoints from the $N_{g}^{\left(j\right)}$ subjects for g=0,s and j=1,2. We also let $Z_{g}=Z_{g}^{\left(1\right)}+Z_{g}^{\left(2\right)}$ for g=0,s. $W_{1}=(X_{0},X_{1}^{\left(1\right)},\ldots ,X_{s-1}^{\left(1\right)},X_{s},X_{s+1}^{\left(1\right)},\ldots ,X_{G}^{\left(1\right)})$ and $W_{2}=(X_{0},X_{1}^{\left(1\right)},\ldots ,X_{s-1}^{\left(1\right)},X_{s},X_{s+1}^{\left(1\right)},\ldots ,X_{G}^{\left(1\right)},Y_{0},Y_{s},Z_{0},Z_{s})$ are sufficient statistics of ξ and $\left(\xi ,\pi_{0},\pi_{s},\rho_{0},\rho_{s}\right)$ given Q=s, respectively. Hence, Rao‐Blackwell's theorem shows that UMVCUE of $\xi_{g}$ for g=0,s can be obtained using the conditional expectation

 ξ^UMVCUE,g =E[Xg(2)⁄Ng(2)|Q=s,W1] =1Ng(2)∑kx=0Ng(1)(Xg−kx)Pr(Xg(1)=kx|Q=s,W1) =XgNg(2)−∑kx=0Ng(1)kx(Ng(1)kx)(Ng(2)Xg−kx)Pr(Q=s|W1,Xg(1)=kx)Ng(2)∑kx=0Ng(1)(Ng(1)kx)(Ng(2)Xg−kx)Pr(Q=s|W1,Xg(1)=kx).

On the other hand, UMVCUE of $\pi_{g}$ for g=0,s is expressed as

 π^UMVCUE,g =E[Yg(2)⁄Ng(2)|Q=s,W2] =1Ng(2)∑ky=0Ng(1)(Yg−ky)Pr(Yg(1)=ky|Q=s,W2) =YgNg(2)−∑ky=0Ng(1)ky(Ng(1)ky)(Ng(2)Yg−ky)Pr(Q=s|W2,Yg(1)=ky)Ng(2)∑ky=0Ng(1)(Ng(1)ky)(Ng(2)Yg−ky)Pr(Q=s|W2,Yg(1)=ky) =YgNg(2)−∑kx,ky,kz=0Ng(1)kyc(kx,ky,kz)Pr(Q=s|W1,Xg(1)=kx)Ng(2)∑kx,ky,kz=0Ng(1)c(kx,ky,kz)Pr(Q=s|W1,Xg(1)=kx),

where

c(kx,ky,kz)=Ng(1)kyNg(2)Yg−kykykzNg(1)−kykx−kz×Yg−kyZg−kzNg(2)−Yg+kyXg−kx−Zg+kz.

The estimator of $\rho_{g}$ for g=0,s corresponding to $\left({\hat{\xi }}_{\text{UMVCUE},g},{\hat{\pi }}_{\text{UMVCUE},g}\right)$ is defined similarly to that of CMAE, that is,

$$\begin{array}{ll} & \ell ({\hat{\xi }}_{\text{UMVCUE},g},{\hat{\pi }}_{\text{UMVCUE},g},{\hat{\rho }}_{\text{UMVCUE},g})\;\\ & \;=\underset{\rho_{g}}{\max}\ell ({\hat{\xi }}_{\text{UMVCUE},g},{\hat{\pi }}_{\text{UMVCUE},g},\rho_{g}).\; \end{array}$$

We used the unbiased estimator $X_{g}^{\left(2\right)}/N_{g}^{\left(2\right)}$ from the subjects on stage 2 to obtain UMVCUE of $\xi_{g}$ for g=0,s. However, after interim analysis, additional subjects are not included in the unselected treatment group g≠0,s. Hence, it is impossible to obtain data that are independent of the interim analysis and the UMVCUE for $\xi_{g}$ for g≠0,s cannot be defined. We use ${\hat{\xi }}_{\text{MLE},g}$ as an estimator of $\xi_{g}$ for g≠0,s to calculate p values for UMVCUE. Hence, the vector ${\hat{\xi }}_{\text{UMVCUE}}$ is defined as $\left({\hat{\xi }}_{\text{UMVCUE},0},{\hat{\xi }}_{\text{MLE},1},\ldots ,{\hat{\xi }}_{\text{MLE},s-1},{\hat{\xi }}_{\text{UMVCUE},s},{\hat{\xi }}_{\text{MLE},s+1},\ldots ,{\hat{\xi }}_{\text{MLE},G}\right)$.

### 3.3 Confidence Interval

The confidence intervals for the treatment effect at the final analysis are constructed using the p values (3) and (4) as functions of $\Delta_{s}$. This idea is the same as that of the Clopper‐Pearson confidence interval [27], and our confidence intervals are compatible with the abovementioned hypothesis tests. In this section, we only describe the exact confidence interval of $\Delta_{s}$ because the interval corresponding to the mid‐p test can be similarly defined. In the definition of the p value, the conditional probability $\mathrm{Pr}(Y_{s}\geq y_{s}|Q=s)$ depends on $\left(\xi ,\pi_{s},\rho_{s}\right)$. This probability can also be regarded as a function of $\left(\xi_{s},\Delta_{s},\rho_{s}\right)$; that is, $\mathrm{Pr}(Y_{s}\geq y_{s}|Q=s,\xi ,\Delta_{s},\rho_{s})$. Let $\hat{\xi }\in \{{\hat{\xi }}_{\text{MLE}},{\hat{\xi }}_{\text{CMAE}},{\hat{\xi }}_{\text{UMVCUE}}\}$ and ${\hat{\rho }}_{s}$ denote the corresponding estimator of $\rho_{s}$. Subsequently, the lower and upper (1−α)% exact confidence limits of $\Delta_{s}$ are given by

$$\begin{array}{l} \inf\left\{\Delta_{s}|\mathrm{Pr}(Y_{s}\geq y_{s}|Q=s,\hat{\xi },\Delta_{s},{\hat{\rho }}_{s})>\frac{\alpha }{2}\right\} \end{array}$$

and

$$\begin{array}{l} \sup\left\{\Delta_{s}|\mathrm{Pr}(Y_{s}\leq y_{s}|Q=s,\hat{\xi },\Delta_{s},{\hat{\rho }}_{s})>\frac{\alpha }{2}\right\}, \end{array}$$

respectively.

## 4 Simulation Study

### 4.1 Simulation Setting

We conducted simulation studies to evaluate the performance of six statistical inference methods, defined as combinations of three estimation methods (MLE, CMAE, and UMVCUE) and two hypothesis tests (exact and mid‐p). We assumed G=2,3,4 treatment groups, and one control group. An interim analysis was performed when 100τ% of all subjects had short‐term endpoint data for each treatment group (i.e., τ is the information fraction). In the interim analysis, we used the selection rule defined in (1). The total sample size $N_{g}$ in each group was assumed to be the same, and we set $N=N_{g}$ to 50 or 200. The correlation coefficient in each group was assumed to be the same, and we let $\rho =\rho_{g}$ be 0.2, 0.4, or 0.6. Short‐ and long‐term endpoints were generated using the bivariate binomial distribution proposed by Biswas and Hwang [24].

The true binomial probabilities of the endpoints are summarized in Table 1. Here, I(·) is the indicator function. We considered two null and three alternative hypotheses. The first null hypothesis (scenario 1) was $\xi_{0}=\xi_{1}=\cdots =\xi_{G}=\pi_{0}=\pi_{1}=\cdots =\pi_{G}$, whereas the second (scenario 2) was $\xi_{0}=\xi_{1}=\cdots =\xi_{G}\neq \pi_{0}=\pi_{1}=\cdots =\pi_{G}$. When $\xi_{g}=0.7$ and $\pi_{g}=0.5$, the parameter α corresponding to ρ=0.6 falls outside the possible range of α defined in Biswas and Hwang, and thus, ρ=0.6 cannot be defined. Hence, we set ρ=0.2,0.4,0.5 in scenario 2. Under the alternative hypotheses, we considered three types of relationships between the treatment group and binomial probability. δ was the maximum difference in the binomial probability between the control and treatment groups. We let δ=0.280 if N=50 and δ=0.145 if N=200.

**TABLE 1:** Simulation scenarios for binomial probabilities.

Scenario		$\xi_{0}$	$\xi_{g}$	$\pi_{0}$	$\pi_{g}$
1	Null hypothesis	0.1	0.1	0.1	0.1
0.5	0.5	0.5	0.5
0.7	0.7	0.7	0.7
2	Null hypothesis	0.3	0.3	0.5	0.5
0.7	0.7	0.5	0.5
3	Only one treatment is effective	0.5	0.5+δI(g=G)	0.5	0.5+δI(g=G)
4	Linear effect	0.5	0.5+gδ/G	0.5	0.5+gδ/G
5	All treatments are effective	0.5	0.5+δ	0.5	0.5+δ

For each generated data set, we calculated the estimates of the binomial probabilities using MLE, CMAE, and UMVCUE. The null hypothesis $H_{0}:\Delta_{g}\leq 0$ for all g=1,…,G was tested using the one‐sided exact and mid‐p tests at a significance level 0.025 by comparing the long‐term endpoints between selected treatment and control groups. We also calculated two‐sided 95% confidence intervals of $\Delta_{s}$. Furthermore, we calculated the expectations $E[\xi_{s}]$ and $E[\pi_{s}]$ which were defined as $\sum_{s=1}^{G}\xi_{s}\mathrm{Pr}(Q=s)$ and $\sum_{s=1}^{G}\pi_{s}\mathrm{Pr}(Q=s)$, respectively. These values can be regarded as the true values of the binomial probabilities.

The number R of simulated data sets for each simulation setting was set to be 100 000 and 10 000 for the null and alternative hypotheses, respectively. The mean and root mean squared error (RMSE) of ${\hat{\xi }}_{s}\in \{{\hat{\xi }}_{\text{MLE},s},{\hat{\xi }}_{\text{CMAE},s},{\hat{\xi }}_{\text{UMVCUE},s}\}$ and ${\hat{\pi }}_{s}\in \{{\hat{\pi }}_{\text{MLE},s},{\hat{\pi }}_{\text{CMAE},s},{\hat{\pi }}_{\text{UMVCUE},s}\}$ and the mean of estimates of the correlation coefficients corresponding to MLE, CMAE, and UMVCUE were calculated. The RMSEs are defined as the square roots of $\sum_{r=1}^{R}{\left({\hat{\xi }}_{s_{r}}-\xi_{s_{r}}\right)}^{2}/R$ and $\sum_{r=1}^{R}{\left({\hat{\pi }}_{s_{r}}-\pi_{s_{r}}\right)}^{2}/R$, where $s_{r}$ is the selected treatment group in the r‐th simulated data set. We calculated the proportion of times that the null hypothesis was rejected, which indicates the type‐I error rate if the null hypothesis is true and the power if the alternative hypothesis is true. We also calculated the coverage probability (CP), defined as the proportion of times the confidence interval contains $\Delta_{s_{r}}$ in the r‐th simulated data set.

### 4.2 Simulation Result

Table 2 summarizes the simulation results for all settings. The upper part of Table 2 presents the distribution of the ranks of the absolute value of the bias and RMSE of ${\hat{\pi }}_{s}\in \{{\hat{\pi }}_{\text{MLE},s},{\hat{\pi }}_{\text{CMAE},s},{\hat{\pi }}_{\text{UMVCUE},s}\}$. UMVCUE exhibited the smallest absolute bias in most cases, followed by CMAE and MLE. In contrast, CMAE showed the smallest RMSE, followed by MLE and UMVCUE. The middle part of Table 2 presents the frequency and proportion of the type‐I error rate falling within 2.4% to 2.6%, less than 2.4%, and greater than 2.6%. The lower part of Table 2 presents the frequency and proportion of the CP falling within 94.5% to 96.5%, less than 94.5%, and greater than 96.5%. Here, the cutoff values for the type‐I error rate and CP were determined based on twice the maximum Monte Carlo errors. The type‐I error rate and CP indicated that the exact test was conservative across all simulation settings regardless of the estimators. The mid‐p test improved the conservativeness of the exact test. However, the mid‐p tests with MLE and UMVCUE showed the type‐I error rate greater than 2.6% in 33.0% and 6.3% of simulation settings, respectively.

**TABLE 2:** Summary of simulation results.

		MLE	CMAE	UMVCUE
Rank of absolute bias	1	10 (2.3%)	52 (12.0%)	419 (97.0%)
	2	7 (1.6%)	376 (87.0%)	13 (3.0%)
	3	415 (96.1%)	4 (0.9%)	0 (0.0%)
Rank of RMSE	1	319 (73.8%)	360 (83.3%)	92 (21.3%)
	2	90 (20.8%)	66 (15.3%)	64 (14.8%)
	3	23 (5.3%)	6 (1.4%)	276 (63.9%)
Type‐I error rate of the exact test	<2.4%	270 (100.0%)	270 (100.0%)	270 (100.0%)
	2.4%≤≤2.6%	0 (0.0%)	0 (0.0%)	0 (0.0%)
	>2.6%	0 (0.0%)	0 (0.0%)	0 (0.0%)
Type‐I error rate of the mid‐p test	<2.4%	64 (23.7%)	179 (66.3%)	98 (36.3%)
	2.4%≤≤2.6%	117 (43.3%)	91 (33.7%)	155 (57.4%)
	>2.6%	89 (33.0%)	0 (0.0%)	17 (6.3%)
Coverage probability of the exact test	<94.5%	0 (0.0%)	0 (0.0%)	0 (0.0%)
	94.5%≤≤95.5%	0 (0.0%)	0 (0.0%)	0 (0.0%)
	>95.5%	432 (100.0%)	432 (100.0%)	432 (100.0%)
Coverage probability of the mid‐p test	<94.5%	0 (0.0%)	0 (0.0%)	0 (0.0%)
	94.5%≤≤95.5%	313 (72.5%)	374 (86.6%)	383 (88.7%)
	>95.5%	119 (27.5%)	58 (13.4%)	49 (11.3%)

*Note:* Rank indicates the descending order of the magnitude of the absolute bias or RMSE. The number of frequencies (%) is presented.

We show the simulation results only for scenario 1 with $\xi_{g}=\pi_{g}\in \{0.1,0.7\}$ and scenarios 4 and 5 when G=4, τ∈{0.5,0.75}, and ρ∈{0.4,0.6}. All simulation results are presented in the Supporting Information. Monte Carlo errors for the mean and RMSE of ${\hat{\pi }}_{s}$, type‐I error rate (%), power (%), and CP (%) were equal to or less than 0.0013, 0.0006, 0.0531, 0.50, and 0.23, respectively.

Table 3 shows the mean and RMSE of the estimators of $\pi_{s}$. The results for the estimators of $\xi_{s}$ and $\rho_{s}$ are provided in the Supporting Information. The MLE had an upward bias in all simulation settings. The bias increased as the information fraction τ, correlation coefficient ρ, and the number of treatment groups G increased. Furthermore, MLE showed a larger bias if the binomial probabilities in the treatment groups were the same. Although the CMAE substantially reduced the bias of MLE, slight bias was observed. In contrast, UMVCUE was unbiased in all settings, coinciding with the unbiasedness of Rao‐Blackwell's theorem. However, the RMSE of UMVCUE is larger than that of CMAE in most cases. In particular, the RMSE of UMVCUE was large when τ, ρ, and G were large.

**TABLE 3:** Mean and RMSE of estimators of $\pi_{s}$ under G=4.

					Mean			RMSE		
	N	τ	$E[\pi_{s}]$	ρ	MLE	CMAE	UMVCUE	MLE	CMAE	UMVCUE
Scenario 1 ($\xi_{g}=\pi_{g}=0.1$)	50	0.50	0.100	0.4	0.113	0.104	0.100	0.045	0.042	0.043
				0.6	0.119	0.105	0.100	0.047	0.042	0.045
		0.75	0.100	0.4	0.116	0.106	0.100	0.046	0.042	0.045
				0.6	0.124	0.109	0.100	0.048	0.043	0.048
	200	0.50	0.100	0.4	0.106	0.102	0.100	0.022	0.021	0.022
				0.6	0.110	0.103	0.100	0.023	0.021	0.023
		0.75	0.100	0.4	0.108	0.103	0.100	0.022	0.021	0.023
				0.6	0.112	0.104	0.100	0.023	0.021	0.025
Scenario 1 ($\xi_{g}=\pi_{g}=0.7$)	50	0.50	0.700	0.4	0.719	0.706	0.700	0.065	0.065	0.069
				0.6	0.727	0.708	0.700	0.067	0.066	0.072
		0.75	0.700	0.4	0.723	0.710	0.700	0.066	0.065	0.074
				0.6	0.734	0.714	0.700	0.068	0.065	0.081
	200	0.50	0.700	0.4	0.709	0.703	0.700	0.033	0.033	0.034
				0.6	0.714	0.704	0.700	0.034	0.033	0.036
		0.75	0.700	0.4	0.712	0.705	0.700	0.033	0.033	0.036
				0.6	0.717	0.707	0.700	0.034	0.033	0.040
Scenario 4	50	0.50	0.738	0.4	0.752	0.743	0.739	0.061	0.062	0.065
				0.6	0.758	0.743	0.737	0.063	0.063	0.068
		0.75	0.750	0.4	0.764	0.755	0.750	0.062	0.062	0.068
				0.6	0.771	0.758	0.750	0.062	0.062	0.073
	200	0.50	0.625	0.4	0.631	0.626	0.624	0.034	0.034	0.035
				0.6	0.636	0.628	0.626	0.035	0.035	0.037
		0.75	0.630	0.4	0.637	0.632	0.630	0.034	0.034	0.037
				0.6	0.642	0.634	0.630	0.035	0.034	0.039
Scenario 5	50	0.50	0.780	0.4	0.797	0.786	0.780	0.058	0.059	0.063
				0.6	0.806	0.789	0.781	0.060	0.060	0.066
		0.75	0.780	0.4	0.800	0.788	0.779	0.059	0.060	0.069
				0.6	0.810	0.793	0.780	0.061	0.060	0.075
	200	0.50	0.645	0.4	0.655	0.648	0.645	0.034	0.034	0.036
				0.6	0.660	0.650	0.645	0.035	0.034	0.037
		0.75	0.645	0.4	0.657	0.650	0.645	0.035	0.034	0.037
				0.6	0.663	0.652	0.645	0.036	0.034	0.041

Table 4 lists the type‐I error rate and power, while Table 5 lists the CP. The type‐I error rate and CP of the exact test were conservative regardless of the estimators of $\pi_{s}$. The mid‐p test improved the conservativeness of the exact test. The mid‐p tests using CMAE and UMVCUE controlled the type‐I error rates at the nominal level in many cases, while the mid‐p test using MLE was inflated when τ and ρ were large. In particular, the type‐I error rate of the two tests using MLE increased as the correlation coefficient became large because the bias of MLE increased. However, in the adjusted estimators, the bias of MLE was substantially reduced and the monotonic relationship between the type‐I error rate and correlation coefficient was not observed. The reason for the non‐monotonicity would be the complex relationship between the correlation coefficient and p value functions (3) and (4). The power of the two tests using MLE was the largest among the three methods owing to the upward bias of MLE. There were little differences in the power of the two tests between CMAE and UMVCUE. The CP of the exact test was higher than the nominal level in all settings, while the CP of the mid‐p test was close to the nominal level.

**TABLE 4:** Type‐I error rate (%) and power (%) of the exact and mid‐p tests at the significance level 0.025 under G=4.

					Exact			Mid‐p		
	N	τ	$E[\pi_{s}]$	ρ	MLE	CMAE	UMVCUE	MLE	CMAE	UMVCUE
Scenario 1 ($\xi_{g}=\pi_{g}=0.1$)	50	0.50	0.100	0.4	1.15	1.05	1.10	2.21	2.02	2.13
				0.6	1.26	1.10	1.20	2.29	2.04	2.18
		0.75	0.100	0.4	1.29	1.12	1.21	2.37	2.09	2.24
				0.6	1.25	1.08	1.19	2.36	2.02	2.22
	200	0.50	0.100	0.4	1.84	1.63	1.72	2.65	2.40	2.50
				0.6	1.89	1.66	1.77	2.70	2.35	2.53
		0.75	0.100	0.4	1.98	1.72	1.78	2.79	2.44	2.54
				0.6	1.93	1.65	1.78	2.72	2.32	2.49
Scenario 1 ($\xi_{g}=\pi_{g}=0.7$)	50	0.50	0.700	0.4	1.66	1.49	1.55	2.57	2.29	2.40
				0.6	1.58	1.34	1.46	2.46	2.11	2.31
		0.75	0.700	0.4	1.79	1.58	1.65	2.75	2.41	2.51
				0.6	1.65	1.37	1.51	2.58	2.18	2.36
	200	0.50	0.700	0.4	2.08	1.86	1.94	2.66	2.36	2.46
				0.6	2.18	1.88	2.04	2.74	2.35	2.55
		0.75	0.700	0.4	2.29	1.98	2.05	2.88	2.49	2.58
				0.6	2.20	1.83	2.00	2.79	2.32	2.53
Scenario 4	50	0.50	0.738	0.4	62.6	60.7	61.4	67.9	66.0	66.7
				0.6	61.0	58.0	59.5	66.6	64.0	65.2
		0.75	0.750	0.4	67.3	64.8	65.2	72.4	70.1	70.1
				0.6	65.5	61.9	63.1	71.4	67.8	68.7
	200	0.50	0.625	0.4	67.2	65.4	66.0	70.0	68.6	69.0
				0.6	67.2	65.0	65.9	70.3	67.7	68.9
		0.75	0.630	0.4	71.1	69.1	69.5	73.9	72.0	71.9
				0.6	70.2	67.0	67.7	73.0	70.2	70.6
Scenario 5	50	0.50	0.780	0.4	79.0	76.2	77.0	83.9	81.5	82.1
				0.6	77.9	74.4	75.7	83.0	79.6	81.0
		0.75	0.780	0.4	78.8	75.2	75.1	83.3	80.4	79.8
				0.6	77.8	72.5	72.6	82.9	78.1	78.0
	200	0.50	0.645	0.4	82.0	80.1	80.6	84.2	82.6	83.1
				0.6	81.5	78.6	79.6	83.9	81.3	82.1
		0.75	0.645	0.4	82.7	79.7	79.2	84.9	82.4	81.8
				0.6	82.1	77.7	77.5	84.6	80.6	80.4

**TABLE 5:** Coverage probability (%) of the 95% confidence intervals of the exact and mid‐p tests under G=4.

					Exact			Mid‐p		
	N	τ	$E[\pi_{s}]$	ρ	MLE	CMAE	UMVCUE	MLE	CMAE	UMVCUE
Scenario 1 ($\xi_{g}=\pi_{g}=0.1$)	50	0.50	0.100	0.4	97.9	97.8	97.8	96.1	95.8	95.9
				0.6	97.9	97.7	97.8	96.2	95.8	95.9
		0.75	0.100	0.4	97.9	97.7	97.6	96.1	95.9	95.7
				0.6	98.1	97.9	97.7	96.4	96.1	95.9
	200	0.50	0.100	0.4	96.8	96.7	96.7	95.3	95.2	95.2
				0.6	96.9	96.7	96.7	95.5	95.3	95.3
		0.75	0.100	0.4	96.8	96.7	96.5	95.4	95.3	95.1
				0.6	97.2	96.9	96.6	95.9	95.6	95.2
Scenario 1 ($\xi_{g}=\pi_{g}=0.7$)	50	0.50	0.700	0.4	97.1	97.0	97.0	95.5	95.4	95.3
				0.6	97.2	97.1	97.1	95.7	95.4	95.4
		0.75	0.700	0.4	97.1	97.0	96.8	95.5	95.4	95.1
				0.6	97.5	97.3	97.0	96.0	95.7	95.3
	200	0.50	0.700	0.4	96.3	96.2	96.2	95.3	95.2	95.2
				0.6	96.3	96.1	96.1	95.3	95.1	95.1
		0.75	0.700	0.4	96.2	96.1	95.9	95.2	95.1	94.8
				0.6	96.6	96.4	96.1	95.6	95.4	95.0
Scenario 4	50	0.50	0.738	0.4	97.5	97.2	97.3	95.8	95.6	95.6
				0.6	97.1	96.8	96.9	95.5	95.3	95.3
		0.75	0.750	0.4	97.1	97.0	96.9	95.8	95.7	95.4
				0.6	97.2	97.0	96.9	95.6	95.3	95.2
	200	0.50	0.625	0.4	96.2	96.2	96.2	95.2	95.1	95.1
				0.6	96.2	96.1	96.1	95.0	94.9	95.0
		0.75	0.630	0.4	96.1	96.0	96.0	95.1	95.0	95.0
				0.6	96.4	96.2	96.0	95.4	95.2	94.9
Scenario 5	50	0.50	0.780	0.4	97.2	97.1	97.0	95.7	95.4	95.4
				0.6	97.0	96.7	96.8	95.3	95.0	95.0
		0.75	0.780	0.4	97.3	97.1	96.8	95.8	95.5	95.0
				0.6	97.2	96.9	96.6	95.5	95.4	94.9
	200	0.50	0.645	0.4	96.3	96.3	96.2	95.4	95.3	95.3
				0.6	96.3	96.2	96.2	95.3	95.2	95.2
		0.75	0.645	0.4	96.5	96.3	96.0	95.5	95.4	95.0
				0.6	96.5	96.4	96.0	95.6	95.5	95.0

## 5 Case Study

Our proposals were applied to a real data set from the BRAVE‐AA1 trial [28, 29], a phase II/III adaptive seamless, double‐blind, randomized, placebo‐controlled trial evaluating the efficacy of baricitinib (BARI) in patients with at least 50% scalp hair loss. The short‐term endpoint was the proportion of patients achieving at least a 30% improvement from baseline in a severity of alopecia tool (SALT) score at week 12, while the long‐term endpoint was the proportion of patients achieving a SALT score of 20 or less at week 36. In stage 1, patients were randomly assigned to receive a placebo, BARI 1 mg, BARI 2 mg, or BARI 4 mg, and the numbers of patients were 28, 28, 27, and 27, respectively. The interim analysis showed short‐term endpoints of 3 (10.7%), 5 (17.9%), 8 (29.6%), and 9 (33.3%), respectively, leading to the treatment selection of BARI 2 mg and BARI 4 mg. The numbers of additional patients for the placebo, BARI 2 mg, and BARI 4 mg groups in stage 2 were 161, 157, and 254, respectively.

In our case study, we assumed that the only one treatment group (BARI 4 mg) was selected in the interim analysis. We further assumed that the short‐term endpoint from patients in stage 2 was the same as that from patients in stage 1, and the correlation coefficient between short‐ and long‐term endpoints was approximately 0.6 because the short‐term endpoint from patients in stage 2 and the correlation coefficient were not reported. In the original data from the BRAVE‐AA1 trial, the interim analysis was performed early (τ=0.12). Hence, we additionally considered two small data where the numbers of patients in stage 2 were reduced to set τ to 0.5 or 0.75. For example, the number of patients for the BARI 4 mg group in stage 2 was set to 27 in the small data with τ=0.5. The number of responses for the endpoint was determined by multiplying the number of responses in the original data by 27/254 to maintain the proportion of the endpoint in stage 2. We assumed the treatment selection rule given by (1). We calculated estimates of the long‐term endpoint, p values (exact and mid‐p), and 95% confidence intervals (exact and mid‐p) using MLE, CMAE, and UMVCUE for three datasets.

Table 6 presents the estimates and 95% confidence intervals. All hypothesis tests yielded p values less than 0.001. There was little difference in estimates of the long‐term endpoint for the original data due to the early timing of the interim analysis. In contrast, CMAE and UMVCUE were smaller than MLE in small data, particularly when τ=0.75. The mid‐p test yielded narrower confidence intervals than the exact test. These results are consistent with the definition of proposals and our simulation results.

**TABLE 6:** Parameter estimates of the long‐term endpoint and 95% confidence interval for the logarithm of the odds ratio for BRAVE‐AA1 trial.

		Original data (τ=0.12)		Small data 1 (τ=0.5)		Small data 2 (τ=0.75)	
		Placebo	BARI 4 mg	Placebo	BARI 4 mg	Placebo	BARI 4 mg
Method	Parameter	($Y_{0}/N_{0}=10/189$)	($Y_{s}/N_{s}=99/281$)	($Y_{0}/N_{0}=3/56$)	($Y_{s}/N_{s}=23/54$)	($Y_{0}/N_{0}=2/37$)	($Y_{s}/N_{s}=17/36$)
MLE	Estimates of $\pi_{g}$	5.3%	35.2%	5.4%	42.6%	5.4%	47.2%
	95% CI for $\Delta_{s}$ (exact)		[1.57,3.06]		[1.16,4.22]		[0.98,4.91]
	95% CI for $\Delta_{s}$ (mid‐p)		[1.61,2.99]		[1.27,3.99]		[1.13,4.57]
CMAE	Estimates of $\pi_{g}$	5.3%	34.9%	5.4%	40.7%	5.4%	44.4%
	95% CI for $\Delta_{s}$ (exact)		[1.56,3.05]		[1.14,4.20]		[0.93,4.86]
	95% CI for $\Delta_{s}$ (mid‐p)		[1.60,2.99]		[1.25,3.97]		[1.08,4.52]
UMVCUE	Estimates of $\pi_{g}$	5.3%	34.9%	5.4%	40.1%	5.4%	42.0%
	95% CI for $\Delta_{s}$ (exact)		[1.57,3.06]		[1.15,4.21]		[0.95,4.88]
	95% CI for $\Delta_{s}$ (mid‐p)		[1.61,2.99]		[1.26,3.98]		[1.09,4.53]

## 6 Discussion

An adaptive seamless design combines phase II and III trials, which are traditionally conducted separately in drug development, into a single trial. This approach can lead to a shorter development period, requiring fewer total subjects, and has garnered increasing interest in enhancing the efficiency of drug development. For example, the ADVENT [30], PROBE [31], and FOCUS [32] trials used this design. Furthermore, the INHANCE study [33] used a short‐term endpoint in the interim analysis. Hence, establishing a valid statistical inference method for a seamless phase II/III trial with an interim analysis using a short‐term endpoint is important.

This study assumed a seamless phase II/III trial with an interim analysis that uses different binary endpoints in the interim and final analyses and developed a statistical inference based on CMAE and UMVCUE. Furthermore, we evaluated the performance of MLE‐based inference and two proposals via a simulation study. The MLE does not consider the presence of an interim analysis; hence, MLE yielded an upward bias owing to treatment selection in all simulation settings. In contrast, CMAE and UMVCUE substantially reduced the bias. Therefore, researchers should use CMAE or UMVCUE rather than MLE to avoid bias.

Notably, CMAE has a slight bias because the conditional bias is estimated using MLE, which is a biased estimator. However, CMAE sufficiently reduced the bias, and the remaining bias would be negligible. Meanwhile, UMVCUE is theoretically unbiased by Rao‐Blackwell's theorem, and our simulation results support this unbiasedness. However, UMVCUE had the maximum RMSE among the three estimators in many settings because UMVCUE is defined by an expectation conditional on an informative statistic about the parameter of interest and discards the information contained in that statistic [8]. Furthermore, UMVCUE can apply only to the selected group since additional subjects are not included in unselected groups. Hence, UMVCUE cannot be used when we are also interested in estimating the parameters of unselected groups.

The type‐I error rate of the exact test was too conservative regardless of estimators. Although the mid‐p test improved the conservativeness, using MLE inflates the type‐I error rate in some settings. In contrast, the mid‐p tests using CMAE and UMVCUE showed the type‐I error rate controlled at the nominal level. Similarly, the CP of the exact test was uniformly higher than the nominal level, while the CP of the mid‐p test was close to the nominal level. Overall, our study showed that statistical inferences based on CMAE + mid‐p or UMVCUE + mid‐p can work well in the setting of adaptive seamless phase II/III trials with short‐ and long‐term binary endpoints.

Our study has a limitation. We assumed that only one treatment group was selected in the interim analysis. If multiple treatment groups are selected in the interim analysis, we should reflect the selection rule in function Q and modify the hypothesis test at the final analysis. The extension should be investigated in future research.

## Conflicts of Interest

The authors declare no conflicts of interest.

## Supporting information




**Data S1.** Supporting Information.

## Data Availability

The data used in the case study are reported in King et al. [28] and King et al. [29].
