# Supplementary material for: Statistical Inference for a Two‐Stage Adaptive Seamless Design Using Different Binary Endpoints
Source: Stat Med. 2025 Mar 6;44(6):e70003. doi: 10.1002/sim.70003 (PMC11884423; doi:10.1002/sim.70003)
Supplement: Supplementary file 1 — Data S1. Supporting Information. [file SIM-44-0-s001.pdf]

# Supporting Information for “Statistical inference for a two-stage adaptive seamless design using different binary endpoints”

Ryota Ishii, Kenichi Takahashi, Kazushi Maruo, and Masahiko Gosho

This supporting information provides all simulation results. Table S1 shows the list of simulation results. The results are presented in Tables S2–S129.

Table S1: List of simulation results.

| Outcome | Parameter | $G$ | Table number |
|---|---|---|---|
| Mean of estimators | $\xi_g = \pi_g = 0.1$ | 2 | S2 |
| | | 3 | S3 |
| | | 4 | S4 |
| | $\xi_g = \pi_g = 0.5$ | 2 | S5 |
| | | 3 | S6 |
| | | 4 | S7 |
| | $\xi_g = \pi_g = 0.7$ | 2 | S8 |
| | | 3 | S9 |
| | | 4 | S10 |
| | $\xi_g = 0.3$ and $\pi_g = 0.5$ | 2 | S11 |
| | | 3 | S12 |
| | | 4 | S13 |
| | $\xi_g = 0.7$ and $\pi_g = 0.5$ | 2 | S14 |
| | | 3 | S15 |
| | | 4 | S16 |
| | Only one treatment is effective | 2 | S17 |
| | | 3 | S18 |
| | | 4 | S19 |
| | Linear effect | 2 | S20 |
| | | 3 | S21 |
| | | 4 | S22 |
| | All treatments are effective | 2 | S23 |
| | | 3 | S24 |
| | | 4 | S25 |
| RMSE of estimators | $\xi_g = \pi_g = 0.1$ | 2 | S26 |
| | | 3 | S27 |
| | | 4 | S28 |
| | $\xi_g = \pi_g = 0.5$ | 2 | S29 |
| | | 3 | S30 |
| | | 4 | S31 |
| | $\xi_g = \pi_g = 0.7$ | 2 | S32 |
| | | 3 | S33 |

Table S1: List of simulation results. *(continued)*

| Outcome | Parameter | $G$ | Table number |
|---|---|---|---|
| | | 4 | S34 |
| | $\xi_g = 0.3$ and $\pi_g = 0.5$ | 2 | S35 |
| | | 3 | S36 |
| | | 4 | S37 |
| | $\xi_g = 0.7$ and $\pi_g = 0.5$ | 2 | S38 |
| | | 3 | S39 |
| | | 4 | S40 |
| | Only one treatment is effective | 2 | S41 |
| | | 3 | S42 |
| | | 4 | S43 |
| | Linear effect | 2 | S44 |
| | | 3 | S45 |
| | | 4 | S46 |
| | All treatments are effective | 2 | S47 |
| | | 3 | S48 |
| | | 4 | S49 |
| Type-I error rate | $\xi_g = \pi_g = 0.1$ | 2 | S50 |
| | | 3 | S51 |
| | | 4 | S52 |
| | $\xi_g = \pi_g = 0.5$ | 2 | S53 |
| | | 3 | S54 |
| | | 4 | S55 |
| | $\xi_g = \pi_g = 0.7$ | 2 | S56 |
| | | 3 | S57 |
| | | 4 | S58 |
| | $\xi_g = 0.3$ and $\pi_g = 0.5$ | 2 | S59 |
| | | 3 | S60 |
| | | 4 | S61 |
| | $\xi_g = 0.7$ and $\pi_g = 0.5$ | 2 | S62 |
| | | 3 | S63 |
| | | 4 | S64 |
| Power | Only one treatment is effective | 2 | S65 |
| | | 3 | S66 |
| | | 4 | S67 |
| | Linear effect | 2 | S68 |
| | | 3 | S69 |
| | | 4 | S70 |
| | All treatments are effective | 2 | S71 |
| | | 3 | S72 |
| | | 4 | S73 |
| Coverage probability | $\xi_g = \pi_g = 0.1$ | 2 | S74 |
| | | 3 | S75 |
| | | 4 | S76 |
| | $\xi_g = \pi_g = 0.5$ | 2 | S77 |

Table S1: List of simulation results. *(continued)*

| Outcome | Parameter | $G$ | Table number |
|---|---|---|---|
| | | 3 | S78 |
| | | 4 | S79 |
| | $\xi_g = \pi_g = 0.7$ | 2 | S80 |
| | | 3 | S81 |
| | | 4 | S82 |
| | $\xi_g = 0.3$ and $\pi_g = 0.5$ | 2 | S83 |
| | | 3 | S84 |
| | | 4 | S85 |
| | $\xi_g = 0.7$ and $\pi_g = 0.5$ | 2 | S86 |
| | | 3 | S87 |
| | | 4 | S88 |
| | Only one treatment is effective | 2 | S89 |
| | | 3 | S90 |
| | | 4 | S91 |
| | Linear effect | 2 | S92 |
| | | 3 | S93 |
| | | 4 | S94 |
| | All treatments are effective | 2 | S95 |
| | | 3 | S96 |
| | | 4 | S97 |
| Probability that the lower confidence limit is greater than the true value | $\xi_g = \pi_g = 0.1$ | 2 | S98 |
| | | 3 | S99 |
| | | 4 | S100 |
| | $\xi_g = \pi_g = 0.5$ | 2 | S101 |
| | | 3 | S102 |
| | | 4 | S103 |
| | $\xi_g = \pi_g = 0.7$ | 2 | S104 |
| | | 3 | S105 |
| | | 4 | S106 |
| | $\xi_g = 0.3$ and $\pi_g = 0.5$ | 2 | S107 |
| | | 3 | S108 |
| | | 4 | S109 |
| | $\xi_g = 0.7$ and $\pi_g = 0.5$ | 2 | S110 |
| | | 3 | S111 |
| | | 4 | S112 |
| | Only one treatment is effective | 2 | S113 |
| | | 3 | S114 |
| | | 4 | S115 |
| | Linear effect | 2 | S116 |
| | | 3 | S117 |
| | | 4 | S118 |
| | All treatments are effective | 2 | S119 |
| | | 3 | S120 |
| | | 4 | S121 |

Table S1: List of simulation results. *(continued)*

| Outcome | Parameter | $G$ | Table number |
|---|---|---|---|
| Mean of $\hat{\rho}$ | $\xi_g = \pi_g = 0.1$ | | S122 |
| | $\xi_g = \pi_g = 0.5$ | | S123 |
| | $\xi_g = \pi_g = 0.7$ | | S124 |
| | $\xi_g = 0.3$ and $\pi_g = 0.5$ | | S125 |
| | $\xi_g = 0.7$ and $\pi_g = 0.5$ | | S126 |
| | Only one treatment is effective | | S127 |
| | Linear effect | | S128 |
| | All treatments are effective | | S129 |

Table S2: Mean of estimators when the null hypothesis is true, $\xi_g = \pi_g = 0.1$, and $G = 2$.

| | | | | | Estimator of $\xi_s$ | | | Estimator of $\pi_s$ | | |
|---|---|---|---|---|---|---|---|---|---|---|
| $N$ | $\tau$ | E[$\xi_s$] | E[$\pi_s$] | $\rho$ | MLE | CMAE | UMVCUE | MLE | CMAE | UMVCUE |
| 50 | 0.25 | 0.100 | 0.100 | 0.2 | 0.112 | 0.104 | 0.100 | 0.102 | 0.101 | 0.100 |
| | | | | 0.4 | 0.112 | 0.104 | 0.100 | 0.105 | 0.102 | 0.100 |
| | | | | 0.6 | 0.112 | 0.104 | 0.100 | 0.107 | 0.103 | 0.100 |
| | 0.50 | 0.100 | 0.100 | 0.2 | 0.117 | 0.107 | 0.100 | 0.103 | 0.101 | 0.100 |
| | | | | 0.4 | 0.117 | 0.107 | 0.100 | 0.106 | 0.102 | 0.100 |
| | | | | 0.6 | 0.117 | 0.107 | 0.100 | 0.110 | 0.104 | 0.100 |
| | 0.75 | 0.100 | 0.100 | 0.2 | 0.120 | 0.109 | 0.100 | 0.104 | 0.102 | 0.100 |
| | | | | 0.4 | 0.121 | 0.109 | 0.100 | 0.109 | 0.104 | 0.100 |
| | | | | 0.6 | 0.121 | 0.109 | 0.100 | 0.112 | 0.105 | 0.100 |
| 200 | 0.25 | 0.100 | 0.100 | 0.2 | 0.106 | 0.102 | 0.100 | 0.101 | 0.100 | 0.100 |
| | | | | 0.4 | 0.106 | 0.102 | 0.100 | 0.102 | 0.101 | 0.100 |
| | | | | 0.6 | 0.106 | 0.102 | 0.100 | 0.103 | 0.101 | 0.100 |
| | 0.50 | 0.100 | 0.100 | 0.2 | 0.108 | 0.103 | 0.100 | 0.102 | 0.101 | 0.100 |
| | | | | 0.4 | 0.108 | 0.103 | 0.100 | 0.103 | 0.101 | 0.100 |
| | | | | 0.6 | 0.108 | 0.103 | 0.100 | 0.105 | 0.102 | 0.100 |
| | 0.75 | 0.100 | 0.100 | 0.2 | 0.110 | 0.104 | 0.100 | 0.102 | 0.101 | 0.100 |
| | | | | 0.4 | 0.110 | 0.104 | 0.100 | 0.104 | 0.102 | 0.100 |
| | | | | 0.6 | 0.110 | 0.104 | 0.100 | 0.106 | 0.103 | 0.100 |

Table S3: Mean of estimators when the null hypothesis is true, $\xi_g = \pi_g = 0.1$, and $G = 3$.

| | | | | | Estimator of $\xi_s$ | | | Estimator of $\pi_s$ | | |
|---|---|---|---|---|---|---|---|---|---|---|
| $N$ | $\tau$ | E[$\xi_s$] | E[$\pi_s$] | $\rho$ | MLE | CMAE | UMVCUE | MLE | CMAE | UMVCUE |
| 50 | 0.25 | 0.100 | 0.100 | 0.2 | 0.118 | 0.106 | 0.100 | 0.104 | 0.101 | 0.100 |
| | | | | 0.4 | 0.118 | 0.106 | 0.100 | 0.107 | 0.102 | 0.100 |
| | | | | 0.6 | 0.118 | 0.106 | 0.100 | 0.111 | 0.103 | 0.100 |
| | 0.50 | 0.100 | 0.100 | 0.2 | 0.126 | 0.109 | 0.100 | 0.105 | 0.102 | 0.100 |
| | | | | 0.4 | 0.126 | 0.108 | 0.100 | 0.110 | 0.103 | 0.100 |
| | | | | 0.6 | 0.126 | 0.108 | 0.100 | 0.115 | 0.105 | 0.100 |
| | 0.75 | 0.100 | 0.100 | 0.2 | 0.132 | 0.112 | 0.100 | 0.106 | 0.102 | 0.100 |
| | | | | 0.4 | 0.131 | 0.112 | 0.100 | 0.113 | 0.105 | 0.100 |
| | | | | 0.6 | 0.132 | 0.112 | 0.100 | 0.119 | 0.107 | 0.100 |
| 200 | 0.25 | 0.100 | 0.100 | 0.2 | 0.109 | 0.102 | 0.100 | 0.102 | 0.100 | 0.100 |
| | | | | 0.4 | 0.109 | 0.102 | 0.100 | 0.104 | 0.101 | 0.100 |
| | | | | 0.6 | 0.109 | 0.102 | 0.100 | 0.106 | 0.101 | 0.100 |
| | 0.50 | 0.100 | 0.100 | 0.2 | 0.113 | 0.104 | 0.100 | 0.102 | 0.101 | 0.100 |
| | | | | 0.4 | 0.113 | 0.104 | 0.100 | 0.105 | 0.102 | 0.100 |
| | | | | 0.6 | 0.113 | 0.104 | 0.100 | 0.108 | 0.102 | 0.100 |
| | 0.75 | 0.100 | 0.100 | 0.2 | 0.116 | 0.106 | 0.100 | 0.103 | 0.101 | 0.100 |
| | | | | 0.4 | 0.116 | 0.106 | 0.100 | 0.106 | 0.102 | 0.100 |
| | | | | 0.6 | 0.116 | 0.106 | 0.100 | 0.109 | 0.104 | 0.100 |

Table S4: Mean of estimators when the null hypothesis is true, $\xi_g = \pi_g = 0.1$, and $G = 4$.

| | | | | | Estimator of $\xi_s$ | | | Estimator of $\pi_s$ | | |
|---|---|---|---|---|---|---|---|---|---|---|
| $N$ | $\tau$ | E[$\xi_s$] | E[$\pi_s$] | $\rho$ | MLE | CMAE | UMVCUE | MLE | CMAE | UMVCUE |
| 50 | 0.25 | 0.100 | 0.100 | 0.2 | 0.123 | 0.106 | 0.100 | 0.105 | 0.101 | 0.100 |
| | | | | 0.4 | 0.123 | 0.106 | 0.100 | 0.109 | 0.103 | 0.100 |
| | | | | 0.6 | 0.123 | 0.106 | 0.100 | 0.114 | 0.104 | 0.100 |
| | 0.50 | 0.100 | 0.100 | 0.2 | 0.132 | 0.109 | 0.100 | 0.106 | 0.102 | 0.100 |
| | | | | 0.4 | 0.132 | 0.109 | 0.100 | 0.113 | 0.104 | 0.100 |
| | | | | 0.6 | 0.132 | 0.109 | 0.100 | 0.119 | 0.105 | 0.100 |
| | 0.75 | 0.100 | 0.100 | 0.2 | 0.139 | 0.114 | 0.100 | 0.108 | 0.103 | 0.100 |
| | | | | 0.4 | 0.139 | 0.114 | 0.100 | 0.116 | 0.106 | 0.100 |
| | | | | 0.6 | 0.139 | 0.114 | 0.100 | 0.124 | 0.109 | 0.100 |
| 200 | 0.25 | 0.100 | 0.100 | 0.2 | 0.111 | 0.102 | 0.100 | 0.102 | 0.100 | 0.100 |
| | | | | 0.4 | 0.111 | 0.102 | 0.100 | 0.104 | 0.101 | 0.100 |
| | | | | 0.6 | 0.111 | 0.102 | 0.100 | 0.107 | 0.101 | 0.100 |
| | 0.50 | 0.100 | 0.100 | 0.2 | 0.116 | 0.104 | 0.100 | 0.103 | 0.101 | 0.100 |
| | | | | 0.4 | 0.116 | 0.104 | 0.100 | 0.106 | 0.102 | 0.100 |
| | | | | 0.6 | 0.116 | 0.105 | 0.100 | 0.110 | 0.103 | 0.100 |
| | 0.75 | 0.100 | 0.100 | 0.2 | 0.119 | 0.107 | 0.100 | 0.104 | 0.101 | 0.100 |
| | | | | 0.4 | 0.119 | 0.107 | 0.100 | 0.108 | 0.103 | 0.100 |
| | | | | 0.6 | 0.119 | 0.107 | 0.100 | 0.112 | 0.104 | 0.100 |

Table S5: Mean of estimators when the null hypothesis is true, $\xi_g = \pi_g = 0.5$, and $G = 2$.

| | | | | | Estimator of $\xi_s$ | | | Estimator of $\pi_s$ | | |
|---|---|---|---|---|---|---|---|---|---|---|
| $N$ | $\tau$ | E[$\xi_s$] | E[$\pi_s$] | $\rho$ | MLE | CMAE | UMVCUE | MLE | CMAE | UMVCUE |
| 50 | 0.25 | 0.500 | 0.500 | 0.2 | 0.520 | 0.506 | 0.500 | 0.504 | 0.501 | 0.500 |
| | | | | 0.4 | 0.520 | 0.506 | 0.500 | 0.508 | 0.503 | 0.500 |
| | | | | 0.6 | 0.520 | 0.506 | 0.500 | 0.512 | 0.504 | 0.500 |
| | 0.50 | 0.500 | 0.500 | 0.2 | 0.528 | 0.510 | 0.500 | 0.506 | 0.502 | 0.500 |
| | | | | 0.4 | 0.528 | 0.510 | 0.499 | 0.511 | 0.504 | 0.500 |
| | | | | 0.6 | 0.528 | 0.510 | 0.500 | 0.517 | 0.506 | 0.500 |
| | 0.75 | 0.500 | 0.500 | 0.2 | 0.535 | 0.515 | 0.500 | 0.507 | 0.503 | 0.500 |
| | | | | 0.4 | 0.535 | 0.515 | 0.500 | 0.514 | 0.506 | 0.500 |
| | | | | 0.6 | 0.535 | 0.515 | 0.500 | 0.521 | 0.509 | 0.500 |
| 200 | 0.25 | 0.500 | 0.500 | 0.2 | 0.510 | 0.503 | 0.500 | 0.502 | 0.501 | 0.500 |
| | | | | 0.4 | 0.510 | 0.503 | 0.500 | 0.504 | 0.501 | 0.500 |
| | | | | 0.6 | 0.510 | 0.503 | 0.500 | 0.506 | 0.502 | 0.500 |
| | 0.50 | 0.500 | 0.500 | 0.2 | 0.514 | 0.505 | 0.500 | 0.503 | 0.501 | 0.500 |
| | | | | 0.4 | 0.514 | 0.505 | 0.500 | 0.506 | 0.502 | 0.500 |
| | | | | 0.6 | 0.514 | 0.505 | 0.500 | 0.508 | 0.503 | 0.500 |
| | 0.75 | 0.500 | 0.500 | 0.2 | 0.517 | 0.507 | 0.500 | 0.504 | 0.502 | 0.500 |
| | | | | 0.4 | 0.517 | 0.507 | 0.500 | 0.507 | 0.503 | 0.500 |
| | | | | 0.6 | 0.517 | 0.507 | 0.500 | 0.510 | 0.504 | 0.500 |

Table S6: Mean of estimators when the null hypothesis is true, $\xi_g = \pi_g = 0.5$, and $G = 3$.

| | | | | | Estimator of $\xi_s$ | | | Estimator of $\pi_s$ | | |
|---|---|---|---|---|---|---|---|---|---|---|
| $N$ | $\tau$ | $E[\xi_s]$ | $E[\pi_s]$ | $\rho$ | MLE | CMAE | UMVCUE | MLE | CMAE | UMVCUE |
| 50 | 0.25 | 0.500 | 0.500 | 0.2 | 0.530 | 0.507 | 0.500 | 0.506 | 0.502 | 0.500 |
| | | | | 0.4 | 0.530 | 0.507 | 0.500 | 0.512 | 0.503 | 0.500 |
| | | | | 0.6 | 0.530 | 0.507 | 0.500 | 0.518 | 0.504 | 0.500 |
| | 0.50 | 0.500 | 0.500 | 0.2 | 0.542 | 0.514 | 0.500 | 0.508 | 0.503 | 0.500 |
| | | | | 0.4 | 0.542 | 0.513 | 0.500 | 0.516 | 0.505 | 0.500 |
| | | | | 0.6 | 0.542 | 0.514 | 0.500 | 0.525 | 0.508 | 0.500 |
| | 0.75 | 0.500 | 0.500 | 0.2 | 0.552 | 0.521 | 0.500 | 0.510 | 0.504 | 0.500 |
| | | | | 0.4 | 0.552 | 0.521 | 0.500 | 0.521 | 0.508 | 0.500 |
| | | | | 0.6 | 0.553 | 0.522 | 0.501 | 0.531 | 0.513 | 0.500 |
| 200 | 0.25 | 0.500 | 0.500 | 0.2 | 0.515 | 0.503 | 0.500 | 0.503 | 0.501 | 0.500 |
| | | | | 0.4 | 0.515 | 0.504 | 0.500 | 0.506 | 0.501 | 0.500 |
| | | | | 0.6 | 0.515 | 0.504 | 0.500 | 0.509 | 0.502 | 0.500 |
| | 0.50 | 0.500 | 0.500 | 0.2 | 0.521 | 0.506 | 0.500 | 0.504 | 0.501 | 0.500 |
| | | | | 0.4 | 0.521 | 0.507 | 0.500 | 0.509 | 0.503 | 0.500 |
| | | | | 0.6 | 0.521 | 0.507 | 0.500 | 0.513 | 0.504 | 0.500 |
| | 0.75 | 0.500 | 0.500 | 0.2 | 0.526 | 0.510 | 0.500 | 0.505 | 0.502 | 0.500 |
| | | | | 0.4 | 0.526 | 0.510 | 0.500 | 0.510 | 0.504 | 0.500 |
| | | | | 0.6 | 0.526 | 0.510 | 0.500 | 0.515 | 0.506 | 0.500 |

Table S7: Mean of estimators when the null hypothesis is true, $\xi_g = \pi_g = 0.5$, and $G = 4$.

| | | | | | Estimator of $\xi_s$ | | | Estimator of $\pi_s$ | | |
|---|---|---|---|---|---|---|---|---|---|---|
| $N$ | $\tau$ | $E[\xi_s]$ | $E[\pi_s]$ | $\rho$ | MLE | CMAE | UMVCUE | MLE | CMAE | UMVCUE |
| 50 | 0.25 | 0.500 | 0.500 | 0.2 | 0.537 | 0.507 | 0.500 | 0.507 | 0.501 | 0.500 |
| | | | | 0.4 | 0.537 | 0.507 | 0.500 | 0.515 | 0.503 | 0.500 |
| | | | | 0.6 | 0.537 | 0.508 | 0.500 | 0.522 | 0.504 | 0.500 |
| | 0.50 | 0.500 | 0.500 | 0.2 | 0.551 | 0.515 | 0.500 | 0.510 | 0.503 | 0.500 |
| | | | | 0.4 | 0.551 | 0.515 | 0.499 | 0.520 | 0.506 | 0.500 |
| | | | | 0.6 | 0.551 | 0.516 | 0.500 | 0.531 | 0.509 | 0.500 |
| | 0.75 | 0.500 | 0.500 | 0.2 | 0.563 | 0.526 | 0.501 | 0.512 | 0.505 | 0.500 |
| | | | | 0.4 | 0.563 | 0.525 | 0.500 | 0.525 | 0.510 | 0.500 |
| | | | | 0.6 | 0.563 | 0.526 | 0.500 | 0.538 | 0.515 | 0.500 |
| 200 | 0.25 | 0.500 | 0.500 | 0.2 | 0.518 | 0.504 | 0.500 | 0.504 | 0.501 | 0.500 |
| | | | | 0.4 | 0.518 | 0.503 | 0.500 | 0.507 | 0.501 | 0.500 |
| | | | | 0.6 | 0.518 | 0.503 | 0.500 | 0.511 | 0.502 | 0.500 |
| | 0.50 | 0.500 | 0.500 | 0.2 | 0.526 | 0.508 | 0.500 | 0.505 | 0.501 | 0.500 |
| | | | | 0.4 | 0.526 | 0.507 | 0.500 | 0.510 | 0.503 | 0.500 |
| | | | | 0.6 | 0.526 | 0.507 | 0.500 | 0.515 | 0.504 | 0.500 |
| | 0.75 | 0.500 | 0.500 | 0.2 | 0.531 | 0.512 | 0.500 | 0.506 | 0.502 | 0.500 |
| | | | | 0.4 | 0.532 | 0.512 | 0.500 | 0.513 | 0.505 | 0.500 |
| | | | | 0.6 | 0.531 | 0.512 | 0.500 | 0.519 | 0.507 | 0.500 |

Table S8: Mean of estimators when the null hypothesis is true, $\xi_g = \pi_g = 0.7$, and $G = 2$.

| | | | | | Estimator of $\xi_s$ | | | Estimator of $\pi_s$ | | |
|---|---|---|---|---|---|---|---|---|---|---|
| $N$ | $\tau$ | E[$\xi_s$] | E[$\pi_s$] | $\rho$ | MLE | CMAE | UMVCUE | MLE | CMAE | UMVCUE |
| 50 | 0.25 | 0.700 | 0.700 | 0.2 | 0.718 | 0.705 | 0.700 | 0.704 | 0.701 | 0.700 |
| | | | | 0.4 | 0.718 | 0.705 | 0.700 | 0.707 | 0.702 | 0.700 |
| | | | | 0.6 | 0.719 | 0.706 | 0.700 | 0.711 | 0.704 | 0.700 |
| | 0.50 | 0.700 | 0.700 | 0.2 | 0.726 | 0.709 | 0.700 | 0.705 | 0.702 | 0.700 |
| | | | | 0.4 | 0.726 | 0.710 | 0.700 | 0.710 | 0.704 | 0.700 |
| | | | | 0.6 | 0.726 | 0.709 | 0.700 | 0.715 | 0.705 | 0.700 |
| | 0.75 | 0.700 | 0.700 | 0.2 | 0.731 | 0.713 | 0.700 | 0.706 | 0.703 | 0.700 |
| | | | | 0.4 | 0.732 | 0.714 | 0.700 | 0.712 | 0.705 | 0.700 |
| | | | | 0.6 | 0.732 | 0.714 | 0.700 | 0.719 | 0.708 | 0.700 |
| 200 | 0.25 | 0.700 | 0.700 | 0.2 | 0.709 | 0.703 | 0.700 | 0.702 | 0.701 | 0.700 |
| | | | | 0.4 | 0.709 | 0.703 | 0.700 | 0.704 | 0.701 | 0.700 |
| | | | | 0.6 | 0.709 | 0.703 | 0.700 | 0.705 | 0.702 | 0.700 |
| | 0.50 | 0.700 | 0.700 | 0.2 | 0.713 | 0.705 | 0.700 | 0.703 | 0.701 | 0.700 |
| | | | | 0.4 | 0.713 | 0.705 | 0.700 | 0.705 | 0.702 | 0.700 |
| | | | | 0.6 | 0.713 | 0.705 | 0.700 | 0.708 | 0.703 | 0.700 |
| | 0.75 | 0.700 | 0.700 | 0.2 | 0.716 | 0.706 | 0.700 | 0.703 | 0.701 | 0.700 |
| | | | | 0.4 | 0.716 | 0.707 | 0.700 | 0.706 | 0.703 | 0.700 |
| | | | | 0.6 | 0.716 | 0.707 | 0.700 | 0.709 | 0.704 | 0.700 |

Table S9: Mean of estimators when the null hypothesis is true, $\xi_g = \pi_g = 0.7$, and $G = 3$.

| | | | | | Estimator of $\xi_s$ | | | Estimator of $\pi_s$ | | |
|---|---|---|---|---|---|---|---|---|---|---|
| $N$ | $\tau$ | E[$\xi_s$] | E[$\pi_s$] | $\rho$ | MLE | CMAE | UMVCUE | MLE | CMAE | UMVCUE |
| 50 | 0.25 | 0.700 | 0.700 | 0.2 | 0.727 | 0.706 | 0.700 | 0.705 | 0.701 | 0.700 |
| | | | | 0.4 | 0.727 | 0.706 | 0.700 | 0.711 | 0.703 | 0.700 |
| | | | | 0.6 | 0.727 | 0.706 | 0.700 | 0.717 | 0.704 | 0.700 |
| | 0.50 | 0.700 | 0.700 | 0.2 | 0.738 | 0.712 | 0.700 | 0.708 | 0.703 | 0.700 |
| | | | | 0.4 | 0.738 | 0.712 | 0.700 | 0.715 | 0.705 | 0.700 |
| | | | | 0.6 | 0.738 | 0.713 | 0.700 | 0.723 | 0.707 | 0.700 |
| | 0.75 | 0.700 | 0.700 | 0.2 | 0.747 | 0.720 | 0.700 | 0.710 | 0.704 | 0.700 |
| | | | | 0.4 | 0.747 | 0.720 | 0.700 | 0.719 | 0.708 | 0.700 |
| | | | | 0.6 | 0.747 | 0.720 | 0.700 | 0.728 | 0.712 | 0.700 |
| 200 | 0.25 | 0.700 | 0.700 | 0.2 | 0.713 | 0.703 | 0.700 | 0.703 | 0.701 | 0.700 |
| | | | | 0.4 | 0.714 | 0.703 | 0.700 | 0.705 | 0.701 | 0.700 |
| | | | | 0.6 | 0.714 | 0.703 | 0.700 | 0.708 | 0.702 | 0.700 |
| | 0.50 | 0.700 | 0.700 | 0.2 | 0.719 | 0.706 | 0.700 | 0.704 | 0.701 | 0.700 |
| | | | | 0.4 | 0.719 | 0.706 | 0.700 | 0.708 | 0.703 | 0.700 |
| | | | | 0.6 | 0.719 | 0.706 | 0.700 | 0.712 | 0.704 | 0.700 |
| | 0.75 | 0.700 | 0.700 | 0.2 | 0.724 | 0.710 | 0.700 | 0.705 | 0.702 | 0.700 |
| | | | | 0.4 | 0.724 | 0.710 | 0.700 | 0.710 | 0.704 | 0.700 |
| | | | | 0.6 | 0.724 | 0.710 | 0.700 | 0.714 | 0.706 | 0.700 |

Table S10: Mean of estimators when the null hypothesis is true, $\xi_g = \pi_g = 0.7$, and $G = 4$.

| | | | | | Estimator of $\xi_s$ | | | Estimator of $\pi_s$ | | |
|---|---|---|---|---|---|---|---|---|---|---|
| $N$ | $\tau$ | E[$\xi_s$] | E[$\pi_s$] | $\rho$ | MLE | CMAE | UMVCUE | MLE | CMAE | UMVCUE |
| 50 | 0.25 | 0.700 | 0.700 | 0.2 | 0.733 | 0.707 | 0.700 | 0.707 | 0.702 | 0.700 |
| | | | | 0.4 | 0.733 | 0.707 | 0.700 | 0.713 | 0.703 | 0.700 |
| | | | | 0.6 | 0.733 | 0.706 | 0.700 | 0.720 | 0.704 | 0.700 |
| | 0.50 | 0.700 | 0.700 | 0.2 | 0.746 | 0.714 | 0.700 | 0.709 | 0.703 | 0.700 |
| | | | | 0.4 | 0.746 | 0.714 | 0.700 | 0.719 | 0.706 | 0.700 |
| | | | | 0.6 | 0.746 | 0.714 | 0.700 | 0.727 | 0.708 | 0.700 |
| | 0.75 | 0.700 | 0.700 | 0.2 | 0.757 | 0.724 | 0.700 | 0.711 | 0.704 | 0.700 |
| | | | | 0.4 | 0.757 | 0.724 | 0.700 | 0.723 | 0.710 | 0.700 |
| | | | | 0.6 | 0.757 | 0.724 | 0.700 | 0.734 | 0.714 | 0.700 |
| 200 | 0.25 | 0.700 | 0.700 | 0.2 | 0.716 | 0.703 | 0.700 | 0.703 | 0.701 | 0.700 |
| | | | | 0.4 | 0.716 | 0.703 | 0.700 | 0.707 | 0.701 | 0.700 |
| | | | | 0.6 | 0.716 | 0.703 | 0.700 | 0.710 | 0.702 | 0.700 |
| | 0.50 | 0.700 | 0.700 | 0.2 | 0.724 | 0.707 | 0.700 | 0.705 | 0.701 | 0.700 |
| | | | | 0.4 | 0.723 | 0.707 | 0.700 | 0.709 | 0.703 | 0.700 |
| | | | | 0.6 | 0.723 | 0.707 | 0.700 | 0.714 | 0.704 | 0.700 |
| | 0.75 | 0.700 | 0.700 | 0.2 | 0.729 | 0.711 | 0.700 | 0.706 | 0.702 | 0.700 |
| | | | | 0.4 | 0.729 | 0.712 | 0.700 | 0.712 | 0.705 | 0.700 |
| | | | | 0.6 | 0.729 | 0.711 | 0.700 | 0.717 | 0.707 | 0.700 |

Table S11: Mean of estimators when the null hypothesis is true, $\xi_g$ =0.3, $\pi_g = 0.5$, and $G = 2$.

| | | | | | Estimator of $\xi_s$ | | | Estimator of $\pi_s$ | | |
|---|---|---|---|---|---|---|---|---|---|---|
| $N$ | $\tau$ | E[$\xi_s$] | E[$\pi_s$] | $\rho$ | MLE | CMAE | UMVCUE | MLE | CMAE | UMVCUE |
| 50 | 0.25 | 0.300 | 0.500 | 0.2 | 0.318 | 0.306 | 0.300 | 0.504 | 0.502 | 0.500 |
| | | | | 0.4 | 0.318 | 0.306 | 0.300 | 0.508 | 0.503 | 0.500 |
| | | | | 0.5 | 0.319 | 0.306 | 0.300 | 0.510 | 0.503 | 0.500 |
| | 0.50 | 0.300 | 0.500 | 0.2 | 0.326 | 0.310 | 0.300 | 0.505 | 0.502 | 0.500 |
| | | | | 0.4 | 0.326 | 0.309 | 0.300 | 0.511 | 0.504 | 0.500 |
| | | | | 0.5 | 0.326 | 0.309 | 0.300 | 0.514 | 0.505 | 0.500 |
| | 0.75 | 0.300 | 0.500 | 0.2 | 0.332 | 0.314 | 0.300 | 0.507 | 0.503 | 0.500 |
| | | | | 0.4 | 0.332 | 0.314 | 0.300 | 0.514 | 0.506 | 0.500 |
| | | | | 0.5 | 0.332 | 0.313 | 0.300 | 0.517 | 0.507 | 0.500 |
| 200 | 0.25 | 0.300 | 0.500 | 0.2 | 0.309 | 0.303 | 0.300 | 0.502 | 0.501 | 0.500 |
| | | | | 0.4 | 0.309 | 0.303 | 0.300 | 0.504 | 0.501 | 0.500 |
| | | | | 0.5 | 0.309 | 0.303 | 0.300 | 0.505 | 0.501 | 0.500 |
| | 0.50 | 0.300 | 0.500 | 0.2 | 0.313 | 0.305 | 0.300 | 0.503 | 0.501 | 0.500 |
| | | | | 0.4 | 0.313 | 0.305 | 0.300 | 0.506 | 0.502 | 0.500 |
| | | | | 0.5 | 0.313 | 0.305 | 0.300 | 0.507 | 0.502 | 0.500 |
| | 0.75 | 0.300 | 0.500 | 0.2 | 0.316 | 0.307 | 0.300 | 0.504 | 0.502 | 0.500 |
| | | | | 0.4 | 0.316 | 0.307 | 0.300 | 0.507 | 0.503 | 0.500 |
| | | | | 0.5 | 0.316 | 0.306 | 0.300 | 0.508 | 0.503 | 0.500 |

Table S12: Mean of estimators when the null hypothesis is true, $\xi_g$ =0.3, $\pi_g$ = 0.5, and $G = 3$.

| | | | | | Estimator of $\xi_s$ | | | Estimator of $\pi_s$ | | |
|---|---|---|---|---|---|---|---|---|---|---|
| $N$ | $\tau$ | E[$\xi_s$] | E[$\pi_s$] | $\rho$ | MLE | CMAE | UMVCUE | MLE | CMAE | UMVCUE |
| 50 | 0.25 | 0.300 | 0.500 | 0.2 | 0.328 | 0.307 | 0.300 | 0.506 | 0.501 | 0.500 |
| | | | | 0.4 | 0.328 | 0.307 | 0.300 | 0.512 | 0.503 | 0.500 |
| | | | | 0.5 | 0.328 | 0.307 | 0.300 | 0.515 | 0.504 | 0.500 |
| | 0.50 | 0.300 | 0.500 | 0.2 | 0.339 | 0.313 | 0.300 | 0.508 | 0.502 | 0.500 |
| | | | | 0.4 | 0.339 | 0.312 | 0.300 | 0.517 | 0.505 | 0.500 |
| | | | | 0.5 | 0.339 | 0.313 | 0.300 | 0.521 | 0.507 | 0.500 |
| | 0.75 | 0.300 | 0.500 | 0.2 | 0.348 | 0.319 | 0.300 | 0.511 | 0.504 | 0.500 |
| | | | | 0.4 | 0.348 | 0.319 | 0.300 | 0.521 | 0.508 | 0.500 |
| | | | | 0.5 | 0.348 | 0.319 | 0.300 | 0.526 | 0.510 | 0.499 |
| 200 | 0.25 | 0.300 | 0.500 | 0.2 | 0.314 | 0.303 | 0.300 | 0.503 | 0.501 | 0.500 |
| | | | | 0.4 | 0.314 | 0.303 | 0.300 | 0.506 | 0.501 | 0.500 |
| | | | | 0.5 | 0.314 | 0.303 | 0.300 | 0.508 | 0.502 | 0.500 |
| | 0.50 | 0.300 | 0.500 | 0.2 | 0.319 | 0.306 | 0.300 | 0.504 | 0.501 | 0.500 |
| | | | | 0.4 | 0.319 | 0.306 | 0.300 | 0.509 | 0.503 | 0.500 |
| | | | | 0.5 | 0.319 | 0.306 | 0.300 | 0.511 | 0.503 | 0.500 |
| | 0.75 | 0.300 | 0.500 | 0.2 | 0.324 | 0.309 | 0.300 | 0.505 | 0.502 | 0.500 |
| | | | | 0.4 | 0.324 | 0.309 | 0.300 | 0.510 | 0.504 | 0.500 |
| | | | | 0.5 | 0.324 | 0.309 | 0.300 | 0.513 | 0.505 | 0.500 |

Table S13: Mean of estimators when the null hypothesis is true, $\xi_g$ =0.3, $\pi_g$ = 0.5, and $G = 4$.

| | | | | | Estimator of $\xi_s$ | | | Estimator of $\pi_s$ | | |
|---|---|---|---|---|---|---|---|---|---|---|
| $N$ | $\tau$ | E[$\xi_s$] | E[$\pi_s$] | $\rho$ | MLE | CMAE | UMVCUE | MLE | CMAE | UMVCUE |
| 50 | 0.25 | 0.300 | 0.500 | 0.2 | 0.334 | 0.307 | 0.300 | 0.507 | 0.501 | 0.500 |
| | | | | 0.4 | 0.334 | 0.307 | 0.300 | 0.515 | 0.503 | 0.500 |
| | | | | 0.5 | 0.334 | 0.307 | 0.300 | 0.519 | 0.504 | 0.500 |
| | 0.50 | 0.300 | 0.500 | 0.2 | 0.348 | 0.314 | 0.300 | 0.510 | 0.503 | 0.500 |
| | | | | 0.4 | 0.347 | 0.314 | 0.300 | 0.520 | 0.506 | 0.500 |
| | | | | 0.5 | 0.348 | 0.314 | 0.300 | 0.526 | 0.507 | 0.500 |
| | 0.75 | 0.300 | 0.500 | 0.2 | 0.359 | 0.323 | 0.301 | 0.513 | 0.505 | 0.500 |
| | | | | 0.4 | 0.359 | 0.323 | 0.300 | 0.526 | 0.510 | 0.500 |
| | | | | 0.5 | 0.359 | 0.323 | 0.300 | 0.532 | 0.513 | 0.500 |
| 200 | 0.25 | 0.300 | 0.500 | 0.2 | 0.317 | 0.303 | 0.300 | 0.504 | 0.501 | 0.500 |
| | | | | 0.4 | 0.317 | 0.303 | 0.300 | 0.507 | 0.501 | 0.500 |
| | | | | 0.5 | 0.317 | 0.303 | 0.300 | 0.509 | 0.502 | 0.500 |
| | 0.50 | 0.300 | 0.500 | 0.2 | 0.324 | 0.307 | 0.300 | 0.505 | 0.502 | 0.500 |
| | | | | 0.4 | 0.324 | 0.307 | 0.300 | 0.510 | 0.503 | 0.500 |
| | | | | 0.5 | 0.324 | 0.307 | 0.300 | 0.513 | 0.504 | 0.500 |
| | 0.75 | 0.300 | 0.500 | 0.2 | 0.329 | 0.311 | 0.300 | 0.506 | 0.502 | 0.500 |
| | | | | 0.4 | 0.329 | 0.311 | 0.300 | 0.513 | 0.505 | 0.500 |
| | | | | 0.5 | 0.329 | 0.311 | 0.300 | 0.516 | 0.506 | 0.500 |

Table S14: Mean of estimators when the null hypothesis is true, $\xi_g$ =0.7, $\pi_g = 0.5$, and $G = 2$.

| | | | | | Estimator of $\xi_s$ | | | Estimator of $\pi_s$ | | |
|---|---|---|---|---|---|---|---|---|---|---|
| $N$ | $\tau$ | E[$\xi_s$] | E[$\pi_s$] | $\rho$ | MLE | CMAE | UMVCUE | MLE | CMAE | UMVCUE |
| 50 | 0.25 | 0.700 | 0.500 | 0.2 | 0.718 | 0.706 | 0.700 | 0.504 | 0.501 | 0.500 |
| | | | | 0.4 | 0.718 | 0.705 | 0.700 | 0.508 | 0.502 | 0.500 |
| | | | | 0.5 | 0.718 | 0.705 | 0.700 | 0.510 | 0.503 | 0.500 |
| | 0.50 | 0.700 | 0.500 | 0.2 | 0.726 | 0.709 | 0.700 | 0.506 | 0.502 | 0.500 |
| | | | | 0.4 | 0.726 | 0.709 | 0.700 | 0.511 | 0.504 | 0.500 |
| | | | | 0.5 | 0.726 | 0.710 | 0.701 | 0.514 | 0.505 | 0.500 |
| | 0.75 | 0.700 | 0.500 | 0.2 | 0.732 | 0.714 | 0.700 | 0.507 | 0.503 | 0.500 |
| | | | | 0.4 | 0.732 | 0.714 | 0.700 | 0.514 | 0.506 | 0.500 |
| | | | | 0.5 | 0.732 | 0.714 | 0.700 | 0.518 | 0.508 | 0.500 |
| 200 | 0.25 | 0.700 | 0.500 | 0.2 | 0.709 | 0.703 | 0.700 | 0.502 | 0.501 | 0.500 |
| | | | | 0.4 | 0.709 | 0.703 | 0.700 | 0.504 | 0.501 | 0.500 |
| | | | | 0.5 | 0.709 | 0.703 | 0.700 | 0.505 | 0.501 | 0.500 |
| | 0.50 | 0.700 | 0.500 | 0.2 | 0.713 | 0.705 | 0.700 | 0.502 | 0.501 | 0.500 |
| | | | | 0.4 | 0.713 | 0.705 | 0.700 | 0.505 | 0.502 | 0.500 |
| | | | | 0.5 | 0.713 | 0.705 | 0.700 | 0.507 | 0.502 | 0.500 |
| | 0.75 | 0.700 | 0.500 | 0.2 | 0.716 | 0.707 | 0.700 | 0.503 | 0.501 | 0.500 |
| | | | | 0.4 | 0.716 | 0.707 | 0.700 | 0.507 | 0.503 | 0.500 |
| | | | | 0.5 | 0.716 | 0.707 | 0.700 | 0.509 | 0.504 | 0.500 |

Table S15: Mean of estimators when the null hypothesis is true, $\xi_g$ =0.7, $\pi_g = 0.5$, and $G = 3$.

| | | | | | Estimator of $\xi_s$ | | | Estimator of $\pi_s$ | | |
|---|---|---|---|---|---|---|---|---|---|---|
| $N$ | $\tau$ | E[$\xi_s$] | E[$\pi_s$] | $\rho$ | MLE | CMAE | UMVCUE | MLE | CMAE | UMVCUE |
| 50 | 0.25 | 0.700 | 0.500 | 0.2 | 0.727 | 0.706 | 0.700 | 0.506 | 0.501 | 0.500 |
| | | | | 0.4 | 0.727 | 0.706 | 0.700 | 0.512 | 0.503 | 0.500 |
| | | | | 0.5 | 0.727 | 0.707 | 0.700 | 0.515 | 0.503 | 0.500 |
| | 0.50 | 0.700 | 0.500 | 0.2 | 0.738 | 0.713 | 0.700 | 0.509 | 0.503 | 0.500 |
| | | | | 0.4 | 0.738 | 0.713 | 0.700 | 0.517 | 0.506 | 0.500 |
| | | | | 0.5 | 0.738 | 0.712 | 0.700 | 0.521 | 0.507 | 0.500 |
| | 0.75 | 0.700 | 0.500 | 0.2 | 0.748 | 0.720 | 0.701 | 0.510 | 0.505 | 0.500 |
| | | | | 0.4 | 0.747 | 0.720 | 0.700 | 0.521 | 0.509 | 0.500 |
| | | | | 0.5 | 0.747 | 0.720 | 0.700 | 0.526 | 0.512 | 0.501 |
| 200 | 0.25 | 0.700 | 0.500 | 0.2 | 0.714 | 0.703 | 0.700 | 0.503 | 0.501 | 0.500 |
| | | | | 0.4 | 0.713 | 0.703 | 0.700 | 0.506 | 0.501 | 0.500 |
| | | | | 0.5 | 0.713 | 0.703 | 0.700 | 0.507 | 0.501 | 0.500 |
| | 0.50 | 0.700 | 0.500 | 0.2 | 0.719 | 0.706 | 0.700 | 0.504 | 0.501 | 0.500 |
| | | | | 0.4 | 0.719 | 0.706 | 0.700 | 0.508 | 0.503 | 0.500 |
| | | | | 0.5 | 0.719 | 0.706 | 0.700 | 0.510 | 0.503 | 0.500 |
| | 0.75 | 0.700 | 0.500 | 0.2 | 0.724 | 0.710 | 0.700 | 0.505 | 0.502 | 0.500 |
| | | | | 0.4 | 0.724 | 0.710 | 0.700 | 0.510 | 0.504 | 0.500 |
| | | | | 0.5 | 0.724 | 0.710 | 0.700 | 0.513 | 0.505 | 0.500 |

Table S16: Mean of estimators when the null hypothesis is true, $\xi_g$ =0.7, $\pi_g = 0.5$, and $G = 4$.

| | | | | | Estimator of $\xi_s$ | | | Estimator of $\pi_s$ | | |
|---|---|---|---|---|---|---|---|---|---|---|
| $N$ | $\tau$ | E[$\xi_s$] | E[$\pi_s$] | $\rho$ | MLE | CMAE | UMVCUE | MLE | CMAE | UMVCUE |
| 50 | 0.25 | 0.700 | 0.500 | 0.2 | 0.732 | 0.706 | 0.699 | 0.507 | 0.502 | 0.500 |
| | | | | 0.4 | 0.732 | 0.706 | 0.700 | 0.514 | 0.503 | 0.500 |
| | | | | 0.5 | 0.733 | 0.707 | 0.700 | 0.518 | 0.504 | 0.500 |
| | 0.50 | 0.700 | 0.500 | 0.2 | 0.746 | 0.714 | 0.700 | 0.510 | 0.503 | 0.500 |
| | | | | 0.4 | 0.746 | 0.714 | 0.700 | 0.520 | 0.506 | 0.500 |
| | | | | 0.5 | 0.746 | 0.714 | 0.700 | 0.525 | 0.508 | 0.500 |
| | 0.75 | 0.700 | 0.500 | 0.2 | 0.757 | 0.724 | 0.700 | 0.513 | 0.505 | 0.500 |
| | | | | 0.4 | 0.757 | 0.724 | 0.700 | 0.525 | 0.511 | 0.500 |
| | | | | 0.5 | 0.757 | 0.724 | 0.700 | 0.531 | 0.513 | 0.500 |
| 200 | 0.25 | 0.700 | 0.500 | 0.2 | 0.716 | 0.703 | 0.700 | 0.504 | 0.501 | 0.500 |
| | | | | 0.4 | 0.717 | 0.703 | 0.700 | 0.507 | 0.501 | 0.500 |
| | | | | 0.5 | 0.716 | 0.703 | 0.700 | 0.509 | 0.501 | 0.500 |
| | 0.50 | 0.700 | 0.500 | 0.2 | 0.723 | 0.707 | 0.700 | 0.505 | 0.502 | 0.500 |
| | | | | 0.4 | 0.723 | 0.707 | 0.700 | 0.510 | 0.503 | 0.500 |
| | | | | 0.5 | 0.723 | 0.707 | 0.700 | 0.513 | 0.504 | 0.500 |
| | 0.75 | 0.700 | 0.500 | 0.2 | 0.729 | 0.711 | 0.700 | 0.506 | 0.503 | 0.500 |
| | | | | 0.4 | 0.729 | 0.711 | 0.700 | 0.513 | 0.505 | 0.500 |
| | | | | 0.5 | 0.729 | 0.712 | 0.700 | 0.516 | 0.506 | 0.500 |

Table S17: Mean of estimators when the alternative hypothesis is true, and only one treatment is effective, and $G = 2$.

| | | | | | Estimator of $\xi_s$ | | | Estimator of $\pi_s$ | | |
|---|---|---|---|---|---|---|---|---|---|---|
| $N$ | $\tau$ | E[$\xi_s$] | E[$\pi_s$] | $\rho$ | MLE | CMAE | UMVCUE | MLE | CMAE | UMVCUE |
| 50 | 0.25 | 0.755 | 0.755 | 0.2 | 0.763 | 0.756 | 0.755 | 0.756 | 0.755 | 0.754 |
| | | | | 0.4 | 0.763 | 0.756 | 0.755 | 0.758 | 0.756 | 0.755 |
| | | | | 0.6 | 0.761 | 0.755 | 0.753 | 0.759 | 0.755 | 0.754 |
| | 0.50 | 0.773 | 0.773 | 0.2 | 0.776 | 0.772 | 0.772 | 0.775 | 0.774 | 0.775 |
| | | | | 0.4 | 0.778 | 0.774 | 0.774 | 0.776 | 0.774 | 0.774 |
| | | | | 0.6 | 0.777 | 0.773 | 0.774 | 0.776 | 0.773 | 0.774 |
| | 0.75 | 0.778 | 0.778 | 0.2 | 0.779 | 0.777 | 0.778 | 0.779 | 0.779 | 0.779 |
| | | | | 0.4 | 0.780 | 0.778 | 0.779 | 0.779 | 0.778 | 0.779 |
| | | | | 0.6 | 0.779 | 0.776 | 0.778 | 0.779 | 0.778 | 0.778 |
| 200 | 0.25 | 0.633 | 0.633 | 0.2 | 0.637 | 0.634 | 0.633 | 0.634 | 0.633 | 0.633 |
| | | | | 0.4 | 0.637 | 0.633 | 0.633 | 0.634 | 0.633 | 0.633 |
| | | | | 0.6 | 0.636 | 0.632 | 0.632 | 0.634 | 0.632 | 0.632 |
| | 0.50 | 0.642 | 0.642 | 0.2 | 0.644 | 0.641 | 0.642 | 0.642 | 0.641 | 0.641 |
| | | | | 0.4 | 0.644 | 0.641 | 0.642 | 0.643 | 0.642 | 0.642 |
| | | | | 0.6 | 0.644 | 0.642 | 0.642 | 0.643 | 0.641 | 0.641 |
| | 0.75 | 0.644 | 0.644 | 0.2 | 0.645 | 0.643 | 0.644 | 0.644 | 0.643 | 0.643 |
| | | | | 0.4 | 0.644 | 0.643 | 0.643 | 0.644 | 0.644 | 0.644 |
| | | | | 0.6 | 0.645 | 0.643 | 0.644 | 0.644 | 0.643 | 0.644 |

Table S18: Mean of estimators when the alternative hypothesis is true, and only one treatment is effective, and $G = 3$.

| | | | | | Estimator of $\xi_s$ | | | Estimator of $\pi_s$ | | |
|---|---|---|---|---|---|---|---|---|---|---|
| $N$ | $\tau$ | E[$\xi_s$] | E[$\pi_s$] | $\rho$ | MLE | CMAE | UMVCUE | MLE | CMAE | UMVCUE |
| 50 | 0.25 | 0.736 | 0.736 | 0.2 | 0.751 | 0.740 | 0.738 | 0.740 | 0.737 | 0.737 |
| | | | | 0.4 | 0.749 | 0.737 | 0.735 | 0.740 | 0.735 | 0.734 |
| | | | | 0.6 | 0.749 | 0.737 | 0.735 | 0.743 | 0.736 | 0.735 |
| | 0.50 | 0.768 | 0.768 | 0.2 | 0.775 | 0.768 | 0.768 | 0.769 | 0.768 | 0.768 |
| | | | | 0.4 | 0.776 | 0.768 | 0.769 | 0.772 | 0.769 | 0.769 |
| | | | | 0.6 | 0.774 | 0.766 | 0.767 | 0.772 | 0.767 | 0.768 |
| | 0.75 | 0.777 | 0.777 | 0.2 | 0.780 | 0.775 | 0.777 | 0.777 | 0.776 | 0.776 |
| | | | | 0.4 | 0.780 | 0.775 | 0.777 | 0.778 | 0.776 | 0.777 |
| | | | | 0.6 | 0.779 | 0.774 | 0.776 | 0.777 | 0.774 | 0.775 |
| 200 | 0.25 | 0.624 | 0.624 | 0.2 | 0.631 | 0.625 | 0.624 | 0.625 | 0.624 | 0.624 |
| | | | | 0.4 | 0.631 | 0.625 | 0.624 | 0.627 | 0.625 | 0.625 |
| | | | | 0.6 | 0.630 | 0.624 | 0.623 | 0.627 | 0.624 | 0.623 |
| | 0.50 | 0.639 | 0.639 | 0.2 | 0.642 | 0.639 | 0.639 | 0.640 | 0.639 | 0.639 |
| | | | | 0.4 | 0.642 | 0.638 | 0.639 | 0.641 | 0.639 | 0.639 |
| | | | | 0.6 | 0.644 | 0.640 | 0.640 | 0.642 | 0.640 | 0.640 |
| | 0.75 | 0.643 | 0.643 | 0.2 | 0.644 | 0.642 | 0.643 | 0.644 | 0.643 | 0.643 |
| | | | | 0.4 | 0.644 | 0.641 | 0.643 | 0.644 | 0.643 | 0.643 |
| | | | | 0.6 | 0.644 | 0.642 | 0.643 | 0.643 | 0.642 | 0.643 |

Table S19: Mean of estimators when the alternative hypothesis is true, and only one treatment is effective, and $G = 4$.

| | | | | | Estimator of $\xi_s$ | | | Estimator of $\pi_s$ | | |
|---|---|---|---|---|---|---|---|---|---|---|
| $N$ | $\tau$ | E[$\xi_s$] | E[$\pi_s$] | $\rho$ | MLE | CMAE | UMVCUE | MLE | CMAE | UMVCUE |
| 50 | 0.25 | 0.723 | 0.723 | 0.2 | 0.739 | 0.723 | 0.721 | 0.725 | 0.721 | 0.721 |
| | | | | 0.4 | 0.740 | 0.724 | 0.722 | 0.730 | 0.723 | 0.723 |
| | | | | 0.6 | 0.739 | 0.723 | 0.721 | 0.733 | 0.723 | 0.722 |
| | 0.50 | 0.763 | 0.763 | 0.2 | 0.774 | 0.763 | 0.764 | 0.766 | 0.763 | 0.764 |
| | | | | 0.4 | 0.774 | 0.763 | 0.764 | 0.767 | 0.763 | 0.763 |
| | | | | 0.6 | 0.772 | 0.762 | 0.763 | 0.768 | 0.762 | 0.763 |
| | 0.75 | 0.775 | 0.775 | 0.2 | 0.779 | 0.773 | 0.776 | 0.777 | 0.775 | 0.776 |
| | | | | 0.4 | 0.780 | 0.773 | 0.776 | 0.777 | 0.775 | 0.776 |
| | | | | 0.6 | 0.779 | 0.773 | 0.776 | 0.778 | 0.774 | 0.776 |
| 200 | 0.25 | 0.618 | 0.618 | 0.2 | 0.627 | 0.619 | 0.618 | 0.620 | 0.618 | 0.618 |
| | | | | 0.4 | 0.627 | 0.619 | 0.618 | 0.622 | 0.618 | 0.618 |
| | | | | 0.6 | 0.626 | 0.618 | 0.617 | 0.622 | 0.617 | 0.617 |
| | 0.50 | 0.637 | 0.637 | 0.2 | 0.642 | 0.637 | 0.637 | 0.638 | 0.637 | 0.637 |
| | | | | 0.4 | 0.641 | 0.636 | 0.637 | 0.638 | 0.636 | 0.637 |
| | | | | 0.6 | 0.641 | 0.636 | 0.637 | 0.639 | 0.636 | 0.637 |
| | 0.75 | 0.643 | 0.643 | 0.2 | 0.645 | 0.641 | 0.643 | 0.643 | 0.642 | 0.643 |
| | | | | 0.4 | 0.645 | 0.641 | 0.643 | 0.643 | 0.642 | 0.642 |
| | | | | 0.6 | 0.645 | 0.641 | 0.643 | 0.644 | 0.642 | 0.643 |

Table S20: Mean of estimators when the alternative hypothesis is true, and relationship between the treatment group and binomial probability is linear, and $G = 2$.

| | | | | | Estimator of $\xi_s$ | | | Estimator of $\pi_s$ | | |
|---|---|---|---|---|---|---|---|---|---|---|
| $N$ | $\tau$ | E[$\xi_s$] | E[$\pi_s$] | $\rho$ | MLE | CMAE | UMVCUE | MLE | CMAE | UMVCUE |
| 50 | 0.25 | 0.741 | 0.741 | 0.2 | 0.756 | 0.745 | 0.740 | 0.744 | 0.742 | 0.741 |
| | | | | 0.4 | 0.755 | 0.744 | 0.740 | 0.747 | 0.743 | 0.741 |
| | | | | 0.6 | 0.755 | 0.743 | 0.739 | 0.749 | 0.743 | 0.740 |
| | 0.50 | 0.756 | 0.756 | 0.2 | 0.772 | 0.761 | 0.756 | 0.760 | 0.758 | 0.757 |
| | | | | 0.4 | 0.772 | 0.761 | 0.756 | 0.763 | 0.759 | 0.757 |
| | | | | 0.6 | 0.772 | 0.760 | 0.755 | 0.766 | 0.759 | 0.755 |
| | 0.75 | 0.765 | 0.765 | 0.2 | 0.778 | 0.768 | 0.763 | 0.767 | 0.765 | 0.764 |
| | | | | 0.4 | 0.779 | 0.768 | 0.764 | 0.770 | 0.765 | 0.764 |
| | | | | 0.6 | 0.779 | 0.769 | 0.765 | 0.773 | 0.766 | 0.764 |
| 200 | 0.25 | 0.626 | 0.626 | 0.2 | 0.635 | 0.629 | 0.627 | 0.628 | 0.627 | 0.627 |
| | | | | 0.4 | 0.634 | 0.628 | 0.626 | 0.630 | 0.627 | 0.626 |
| | | | | 0.6 | 0.635 | 0.630 | 0.627 | 0.631 | 0.628 | 0.627 |
| | 0.50 | 0.633 | 0.633 | 0.2 | 0.642 | 0.635 | 0.633 | 0.635 | 0.634 | 0.633 |
| | | | | 0.4 | 0.641 | 0.635 | 0.633 | 0.636 | 0.634 | 0.633 |
| | | | | 0.6 | 0.641 | 0.635 | 0.633 | 0.638 | 0.634 | 0.633 |
| | 0.75 | 0.637 | 0.637 | 0.2 | 0.646 | 0.640 | 0.638 | 0.639 | 0.638 | 0.638 |
| | | | | 0.4 | 0.645 | 0.640 | 0.638 | 0.641 | 0.638 | 0.638 |
| | | | | 0.6 | 0.645 | 0.639 | 0.637 | 0.642 | 0.638 | 0.637 |

Table S21: Mean of estimators when the alternative hypothesis is true, and relationship between the treatment group and binomial probability is linear, and $G = 3$.

| | | | | | Estimator of $\xi_s$ | | | Estimator of $\pi_s$ | | |
|---|---|---|---|---|---|---|---|---|---|---|
| $N$ | $\tau$ | E[$\xi_s$] | E[$\pi_s$] | $\rho$ | MLE | CMAE | UMVCUE | MLE | CMAE | UMVCUE |
| 50 | 0.25 | 0.725 | 0.725 | 0.2 | 0.749 | 0.730 | 0.725 | 0.730 | 0.726 | 0.725 |
| | | | | 0.4 | 0.749 | 0.730 | 0.725 | 0.735 | 0.727 | 0.725 |
| | | | | 0.6 | 0.750 | 0.732 | 0.727 | 0.741 | 0.730 | 0.726 |
| | 0.50 | 0.744 | 0.744 | 0.2 | 0.772 | 0.753 | 0.745 | 0.751 | 0.747 | 0.746 |
| | | | | 0.4 | 0.772 | 0.753 | 0.745 | 0.755 | 0.747 | 0.744 |
| | | | | 0.6 | 0.772 | 0.753 | 0.745 | 0.761 | 0.750 | 0.745 |
| | 0.75 | 0.755 | 0.755 | 0.2 | 0.782 | 0.764 | 0.755 | 0.761 | 0.758 | 0.756 |
| | | | | 0.4 | 0.783 | 0.765 | 0.756 | 0.766 | 0.759 | 0.755 |
| | | | | 0.6 | 0.781 | 0.763 | 0.754 | 0.771 | 0.760 | 0.755 |
| 200 | 0.25 | 0.619 | 0.619 | 0.2 | 0.631 | 0.622 | 0.619 | 0.621 | 0.620 | 0.619 |
| | | | | 0.4 | 0.632 | 0.623 | 0.620 | 0.625 | 0.621 | 0.620 |
| | | | | 0.6 | 0.632 | 0.623 | 0.620 | 0.627 | 0.621 | 0.620 |
| | 0.50 | 0.628 | 0.628 | 0.2 | 0.642 | 0.631 | 0.627 | 0.631 | 0.629 | 0.628 |
| | | | | 0.4 | 0.642 | 0.632 | 0.628 | 0.634 | 0.630 | 0.629 |
| | | | | 0.6 | 0.641 | 0.631 | 0.627 | 0.637 | 0.630 | 0.628 |
| | 0.75 | 0.632 | 0.632 | 0.2 | 0.647 | 0.637 | 0.633 | 0.635 | 0.633 | 0.632 |
| | | | | 0.4 | 0.647 | 0.637 | 0.633 | 0.638 | 0.634 | 0.632 |
| | | | | 0.6 | 0.647 | 0.637 | 0.633 | 0.641 | 0.635 | 0.633 |

Table S22: Mean of estimators when the alternative hypothesis is true, and relationship between the treatment group and binomial probability is linear, and $G = 4$.

| | | | | | Estimator of $\xi_s$ | | | Estimator of $\pi_s$ | | |
|---|---|---|---|---|---|---|---|---|---|---|
| $N$ | $\tau$ | E[$\xi_s$] | E[$\pi_s$] | $\rho$ | MLE | CMAE | UMVCUE | MLE | CMAE | UMVCUE |
| 50 | 0.25 | 0.717 | 0.717 | 0.2 | 0.746 | 0.722 | 0.717 | 0.722 | 0.718 | 0.717 |
| | | | | 0.4 | 0.746 | 0.722 | 0.716 | 0.730 | 0.720 | 0.718 |
| | | | | 0.6 | 0.748 | 0.724 | 0.718 | 0.735 | 0.721 | 0.718 |
| | 0.50 | 0.738 | 0.738 | 0.2 | 0.773 | 0.748 | 0.739 | 0.746 | 0.741 | 0.739 |
| | | | | 0.4 | 0.772 | 0.748 | 0.739 | 0.752 | 0.743 | 0.739 |
| | | | | 0.6 | 0.772 | 0.748 | 0.738 | 0.758 | 0.743 | 0.737 |
| | 0.75 | 0.750 | 0.750 | 0.2 | 0.785 | 0.762 | 0.749 | 0.757 | 0.752 | 0.749 |
| | | | | 0.4 | 0.785 | 0.762 | 0.749 | 0.764 | 0.755 | 0.750 |
| | | | | 0.6 | 0.786 | 0.763 | 0.750 | 0.771 | 0.758 | 0.750 |
| 200 | 0.25 | 0.616 | 0.616 | 0.2 | 0.631 | 0.618 | 0.616 | 0.619 | 0.616 | 0.616 |
| | | | | 0.4 | 0.631 | 0.618 | 0.616 | 0.621 | 0.616 | 0.615 |
| | | | | 0.6 | 0.630 | 0.618 | 0.615 | 0.625 | 0.617 | 0.616 |
| | 0.50 | 0.625 | 0.625 | 0.2 | 0.642 | 0.629 | 0.624 | 0.628 | 0.626 | 0.625 |
| | | | | 0.4 | 0.643 | 0.629 | 0.625 | 0.631 | 0.626 | 0.624 |
| | | | | 0.6 | 0.643 | 0.630 | 0.626 | 0.636 | 0.628 | 0.626 |
| | 0.75 | 0.630 | 0.630 | 0.2 | 0.649 | 0.637 | 0.631 | 0.634 | 0.631 | 0.630 |
| | | | | 0.4 | 0.649 | 0.637 | 0.630 | 0.637 | 0.632 | 0.630 |
| | | | | 0.6 | 0.649 | 0.636 | 0.630 | 0.642 | 0.634 | 0.630 |

Table S23: Mean of estimators when the alternative hypothesis is true, and all treatments are effective, and $G = 2$.

| | | | | | Estimator of $\xi_s$ | | | Estimator of $\pi_s$ | | |
|---|---|---|---|---|---|---|---|---|---|---|
| $N$ | $\tau$ | E[$\xi_s$] | E[$\pi_s$] | $\rho$ | MLE | CMAE | UMVCUE | MLE | CMAE | UMVCUE |
| 50 | 0.25 | 0.780 | 0.780 | 0.2 | 0.796 | 0.785 | 0.780 | 0.783 | 0.781 | 0.780 |
| | | | | 0.4 | 0.797 | 0.785 | 0.780 | 0.788 | 0.783 | 0.781 |
| | | | | 0.6 | 0.797 | 0.785 | 0.780 | 0.790 | 0.783 | 0.780 |
| | 0.50 | 0.780 | 0.780 | 0.2 | 0.804 | 0.789 | 0.781 | 0.785 | 0.782 | 0.780 |
| | | | | 0.4 | 0.804 | 0.790 | 0.781 | 0.789 | 0.783 | 0.780 |
| | | | | 0.6 | 0.804 | 0.789 | 0.780 | 0.794 | 0.785 | 0.780 |
| | 0.75 | 0.780 | 0.780 | 0.2 | 0.809 | 0.793 | 0.780 | 0.786 | 0.783 | 0.780 |
| | | | | 0.4 | 0.808 | 0.792 | 0.780 | 0.792 | 0.785 | 0.780 |
| | | | | 0.6 | 0.809 | 0.793 | 0.781 | 0.798 | 0.788 | 0.781 |
| 200 | 0.25 | 0.645 | 0.645 | 0.2 | 0.655 | 0.648 | 0.646 | 0.647 | 0.646 | 0.645 |
| | | | | 0.4 | 0.655 | 0.648 | 0.645 | 0.649 | 0.646 | 0.645 |
| | | | | 0.6 | 0.654 | 0.648 | 0.645 | 0.651 | 0.647 | 0.645 |
| | 0.50 | 0.645 | 0.645 | 0.2 | 0.659 | 0.650 | 0.645 | 0.648 | 0.646 | 0.645 |
| | | | | 0.4 | 0.659 | 0.650 | 0.645 | 0.651 | 0.648 | 0.646 |
| | | | | 0.6 | 0.658 | 0.649 | 0.644 | 0.653 | 0.648 | 0.645 |
| | 0.75 | 0.645 | 0.645 | 0.2 | 0.662 | 0.652 | 0.645 | 0.648 | 0.646 | 0.645 |
| | | | | 0.4 | 0.662 | 0.652 | 0.645 | 0.652 | 0.648 | 0.645 |
| | | | | 0.6 | 0.662 | 0.652 | 0.645 | 0.655 | 0.649 | 0.645 |

Table S24: Mean of estimators when the alternative hypothesis is true, and all treatments are effective, and $G = 3$.

| | | | | | Estimator of $\xi_s$ | | | Estimator of $\pi_s$ | | |
|---|---|---|---|---|---|---|---|---|---|---|
| $N$ | $\tau$ | E[$\xi_s$] | E[$\pi_s$] | $\rho$ | MLE | CMAE | UMVCUE | MLE | CMAE | UMVCUE |
| 50 | 0.25 | 0.780 | 0.780 | 0.2 | 0.804 | 0.785 | 0.779 | 0.784 | 0.781 | 0.779 |
| | | | | 0.4 | 0.804 | 0.786 | 0.780 | 0.790 | 0.783 | 0.781 |
| | | | | 0.6 | 0.805 | 0.787 | 0.781 | 0.795 | 0.784 | 0.781 |
| | 0.50 | 0.780 | 0.780 | 0.2 | 0.814 | 0.791 | 0.780 | 0.788 | 0.784 | 0.781 |
| | | | | 0.4 | 0.814 | 0.791 | 0.779 | 0.793 | 0.784 | 0.779 |
| | | | | 0.6 | 0.813 | 0.791 | 0.779 | 0.800 | 0.786 | 0.780 |
| | 0.75 | 0.780 | 0.780 | 0.2 | 0.822 | 0.798 | 0.780 | 0.788 | 0.783 | 0.780 |
| | | | | 0.4 | 0.822 | 0.799 | 0.780 | 0.797 | 0.788 | 0.780 |
| | | | | 0.6 | 0.823 | 0.799 | 0.780 | 0.806 | 0.792 | 0.780 |
| 200 | 0.25 | 0.645 | 0.645 | 0.2 | 0.659 | 0.648 | 0.645 | 0.648 | 0.646 | 0.645 |
| | | | | 0.4 | 0.659 | 0.648 | 0.645 | 0.650 | 0.646 | 0.645 |
| | | | | 0.6 | 0.659 | 0.648 | 0.645 | 0.653 | 0.646 | 0.645 |
| | 0.50 | 0.645 | 0.645 | 0.2 | 0.665 | 0.651 | 0.645 | 0.649 | 0.646 | 0.645 |
| | | | | 0.4 | 0.665 | 0.651 | 0.645 | 0.653 | 0.647 | 0.645 |
| | | | | 0.6 | 0.665 | 0.651 | 0.645 | 0.657 | 0.649 | 0.645 |
| | 0.75 | 0.645 | 0.645 | 0.2 | 0.669 | 0.655 | 0.644 | 0.650 | 0.647 | 0.645 |
| | | | | 0.4 | 0.670 | 0.655 | 0.644 | 0.655 | 0.649 | 0.645 |
| | | | | 0.6 | 0.670 | 0.655 | 0.645 | 0.660 | 0.651 | 0.645 |

Table S25: Mean of estimators when the alternative hypothesis is true, and all treatments are effective, and $G = 4$.

| | | | | | Estimator of $\xi_s$ | | | Estimator of $\pi_s$ | | |
|---|---|---|---|---|---|---|---|---|---|---|
| $N$ | $\tau$ | E[$\xi_s$] | E[$\pi_s$] | $\rho$ | MLE | CMAE | UMVCUE | MLE | CMAE | UMVCUE |
| 50 | 0.25 | 0.780 | 0.780 | 0.2 | 0.809 | 0.786 | 0.780 | 0.786 | 0.781 | 0.780 |
| | | | | 0.4 | 0.809 | 0.785 | 0.780 | 0.792 | 0.782 | 0.780 |
| | | | | 0.6 | 0.808 | 0.785 | 0.779 | 0.797 | 0.782 | 0.779 |
| | 0.50 | 0.780 | 0.780 | 0.2 | 0.820 | 0.792 | 0.779 | 0.788 | 0.782 | 0.780 |
| | | | | 0.4 | 0.821 | 0.793 | 0.780 | 0.797 | 0.786 | 0.780 |
| | | | | 0.6 | 0.822 | 0.793 | 0.780 | 0.806 | 0.789 | 0.781 |
| | 0.75 | 0.780 | 0.780 | 0.2 | 0.832 | 0.803 | 0.781 | 0.790 | 0.785 | 0.780 |
| | | | | 0.4 | 0.831 | 0.802 | 0.780 | 0.800 | 0.788 | 0.779 |
| | | | | 0.6 | 0.831 | 0.803 | 0.780 | 0.810 | 0.793 | 0.780 |
| 200 | 0.25 | 0.645 | 0.645 | 0.2 | 0.663 | 0.649 | 0.646 | 0.649 | 0.646 | 0.645 |
| | | | | 0.4 | 0.662 | 0.648 | 0.645 | 0.652 | 0.646 | 0.645 |
| | | | | 0.6 | 0.662 | 0.648 | 0.645 | 0.655 | 0.647 | 0.645 |
| | 0.50 | 0.645 | 0.645 | 0.2 | 0.670 | 0.652 | 0.645 | 0.650 | 0.646 | 0.645 |
| | | | | 0.4 | 0.670 | 0.653 | 0.645 | 0.655 | 0.648 | 0.645 |
| | | | | 0.6 | 0.670 | 0.652 | 0.645 | 0.660 | 0.650 | 0.645 |
| | 0.75 | 0.645 | 0.645 | 0.2 | 0.675 | 0.657 | 0.645 | 0.651 | 0.647 | 0.645 |
| | | | | 0.4 | 0.675 | 0.657 | 0.645 | 0.657 | 0.650 | 0.645 |
| | | | | 0.6 | 0.675 | 0.657 | 0.645 | 0.663 | 0.652 | 0.645 |

Table S26: RMSE of estimators when the null hypothesis is true, $\xi_g = \pi_g = 0.1$, and $G = 2$.

| | | | | | Estimator of $\xi_s$ | | | Estimator of $\pi_s$ | | |
|---|---|---|---|---|---|---|---|---|---|---|
| $N$ | $\tau$ | $E[\xi_s]$ | $E[\pi_s]$ | $\rho$ | MLE | CMAE | UMVCUE | MLE | CMAE | UMVCUE |
| 50 | 0.25 | 0.100 | 0.100 | 0.2 | 0.044 | 0.043 | 0.044 | 0.043 | 0.042 | 0.042 |
| | | | | 0.4 | 0.044 | 0.043 | 0.044 | 0.043 | 0.042 | 0.042 |
| | | | | 0.6 | 0.044 | 0.042 | 0.044 | 0.044 | 0.042 | 0.043 |
| | 0.50 | 0.100 | 0.100 | 0.2 | 0.044 | 0.042 | 0.047 | 0.043 | 0.042 | 0.042 |
| | | | | 0.4 | 0.044 | 0.042 | 0.047 | 0.043 | 0.042 | 0.043 |
| | | | | 0.6 | 0.044 | 0.042 | 0.047 | 0.044 | 0.042 | 0.044 |
| | 0.75 | 0.100 | 0.100 | 0.2 | 0.044 | 0.042 | 0.052 | 0.043 | 0.042 | 0.043 |
| | | | | 0.4 | 0.044 | 0.042 | 0.052 | 0.044 | 0.042 | 0.044 |
| | | | | 0.6 | 0.044 | 0.042 | 0.052 | 0.044 | 0.042 | 0.046 |
| 200 | 0.25 | 0.100 | 0.100 | 0.2 | 0.021 | 0.021 | 0.022 | 0.021 | 0.021 | 0.021 |
| | | | | 0.4 | 0.021 | 0.021 | 0.022 | 0.021 | 0.021 | 0.021 |
| | | | | 0.6 | 0.022 | 0.021 | 0.022 | 0.022 | 0.021 | 0.022 |
| | 0.50 | 0.100 | 0.100 | 0.2 | 0.022 | 0.021 | 0.024 | 0.021 | 0.021 | 0.021 |
| | | | | 0.4 | 0.022 | 0.021 | 0.024 | 0.021 | 0.021 | 0.021 |
| | | | | 0.6 | 0.022 | 0.021 | 0.024 | 0.021 | 0.021 | 0.022 |
| | 0.75 | 0.100 | 0.100 | 0.2 | 0.022 | 0.021 | 0.027 | 0.021 | 0.021 | 0.021 |
| | | | | 0.4 | 0.022 | 0.021 | 0.027 | 0.022 | 0.021 | 0.022 |
| | | | | 0.6 | 0.022 | 0.021 | 0.027 | 0.022 | 0.021 | 0.023 |

Table S27: RMSE of estimators when the null hypothesis is true, $\xi_g = \pi_g = 0.1$, and $G = 3$.

| | | | | | Estimator of $\xi_s$ | | | Estimator of $\pi_s$ | | |
|---|---|---|---|---|---|---|---|---|---|---|
| $N$ | $\tau$ | $E[\xi_s]$ | $E[\pi_s]$ | $\rho$ | MLE | CMAE | UMVCUE | MLE | CMAE | UMVCUE |
| 50 | 0.25 | 0.100 | 0.100 | 0.2 | 0.045 | 0.043 | 0.045 | 0.043 | 0.042 | 0.042 |
| | | | | 0.4 | 0.046 | 0.043 | 0.045 | 0.044 | 0.042 | 0.042 |
| | | | | 0.6 | 0.046 | 0.043 | 0.045 | 0.044 | 0.042 | 0.043 |
| | 0.50 | 0.100 | 0.100 | 0.2 | 0.047 | 0.043 | 0.049 | 0.043 | 0.042 | 0.042 |
| | | | | 0.4 | 0.047 | 0.043 | 0.049 | 0.044 | 0.042 | 0.043 |
| | | | | 0.6 | 0.047 | 0.043 | 0.049 | 0.045 | 0.042 | 0.045 |
| | 0.75 | 0.100 | 0.100 | 0.2 | 0.049 | 0.043 | 0.056 | 0.044 | 0.042 | 0.043 |
| | | | | 0.4 | 0.049 | 0.043 | 0.056 | 0.045 | 0.042 | 0.045 |
| | | | | 0.6 | 0.049 | 0.043 | 0.056 | 0.046 | 0.042 | 0.047 |
| 200 | 0.25 | 0.100 | 0.100 | 0.2 | 0.022 | 0.021 | 0.023 | 0.021 | 0.021 | 0.021 |
| | | | | 0.4 | 0.022 | 0.021 | 0.023 | 0.022 | 0.021 | 0.021 |
| | | | | 0.6 | 0.022 | 0.021 | 0.023 | 0.022 | 0.021 | 0.022 |
| | 0.50 | 0.100 | 0.100 | 0.2 | 0.023 | 0.021 | 0.025 | 0.021 | 0.021 | 0.021 |
| | | | | 0.4 | 0.023 | 0.021 | 0.025 | 0.022 | 0.021 | 0.022 |
| | | | | 0.6 | 0.023 | 0.021 | 0.025 | 0.022 | 0.021 | 0.023 |
| | 0.75 | 0.100 | 0.100 | 0.2 | 0.024 | 0.021 | 0.029 | 0.022 | 0.021 | 0.021 |
| | | | | 0.4 | 0.024 | 0.021 | 0.029 | 0.022 | 0.021 | 0.022 |
| | | | | 0.6 | 0.024 | 0.021 | 0.029 | 0.023 | 0.021 | 0.024 |

Table S28: RMSE of estimators when the null hypothesis is true, $\xi_g = \pi_g = 0.1$, and $G = 4$.

| | | | | | Estimator of $\xi_s$ | | | Estimator of $\pi_s$ | | |
|---|---|---|---|---|---|---|---|---|---|---|
| $N$ | $\tau$ | E[$\xi_s$] | E[$\pi_s$] | $\rho$ | MLE | CMAE | UMVCUE | MLE | CMAE | UMVCUE |
| 50 | 0.25 | 0.100 | 0.100 | 0.2 | 0.047 | 0.043 | 0.046 | 0.043 | 0.042 | 0.042 |
| | | | | 0.4 | 0.047 | 0.043 | 0.046 | 0.044 | 0.042 | 0.042 |
| | | | | 0.6 | 0.047 | 0.043 | 0.046 | 0.045 | 0.042 | 0.043 |
| | 0.50 | 0.100 | 0.100 | 0.2 | 0.051 | 0.043 | 0.050 | 0.044 | 0.042 | 0.043 |
| | | | | 0.4 | 0.051 | 0.043 | 0.050 | 0.045 | 0.042 | 0.043 |
| | | | | 0.6 | 0.050 | 0.043 | 0.050 | 0.047 | 0.042 | 0.045 |
| | 0.75 | 0.100 | 0.100 | 0.2 | 0.054 | 0.043 | 0.058 | 0.044 | 0.043 | 0.043 |
| | | | | 0.4 | 0.054 | 0.043 | 0.058 | 0.046 | 0.042 | 0.045 |
| | | | | 0.6 | 0.054 | 0.043 | 0.058 | 0.048 | 0.043 | 0.048 |
| 200 | 0.25 | 0.100 | 0.100 | 0.2 | 0.023 | 0.021 | 0.023 | 0.022 | 0.021 | 0.021 |
| | | | | 0.4 | 0.023 | 0.021 | 0.023 | 0.022 | 0.021 | 0.021 |
| | | | | 0.6 | 0.023 | 0.022 | 0.023 | 0.022 | 0.021 | 0.022 |
| | 0.50 | 0.100 | 0.100 | 0.2 | 0.025 | 0.022 | 0.026 | 0.022 | 0.021 | 0.021 |
| | | | | 0.4 | 0.025 | 0.022 | 0.026 | 0.022 | 0.021 | 0.022 |
| | | | | 0.6 | 0.025 | 0.022 | 0.026 | 0.023 | 0.021 | 0.023 |
| | 0.75 | 0.100 | 0.100 | 0.2 | 0.026 | 0.021 | 0.030 | 0.022 | 0.021 | 0.022 |
| | | | | 0.4 | 0.026 | 0.021 | 0.030 | 0.022 | 0.021 | 0.023 |
| | | | | 0.6 | 0.026 | 0.021 | 0.030 | 0.023 | 0.021 | 0.025 |

Table S29: RMSE of estimators when the null hypothesis is true, $\xi_g = \pi_g = 0.5$, and $G = 2$.

| | | | | | Estimator of $\xi_s$ | | | Estimator of $\pi_s$ | | |
|---|---|---|---|---|---|---|---|---|---|---|
| $N$ | $\tau$ | E[$\xi_s$] | E[$\pi_s$] | $\rho$ | MLE | CMAE | UMVCUE | MLE | CMAE | UMVCUE |
| 50 | 0.25 | 0.500 | 0.500 | 0.2 | 0.071 | 0.071 | 0.076 | 0.071 | 0.071 | 0.071 |
| | | | | 0.4 | 0.071 | 0.071 | 0.076 | 0.071 | 0.071 | 0.072 |
| | | | | 0.6 | 0.071 | 0.071 | 0.076 | 0.071 | 0.071 | 0.073 |
| | 0.50 | 0.500 | 0.500 | 0.2 | 0.070 | 0.070 | 0.082 | 0.071 | 0.071 | 0.072 |
| | | | | 0.4 | 0.070 | 0.070 | 0.082 | 0.071 | 0.071 | 0.073 |
| | | | | 0.6 | 0.071 | 0.071 | 0.082 | 0.071 | 0.071 | 0.075 |
| | 0.75 | 0.500 | 0.500 | 0.2 | 0.071 | 0.069 | 0.095 | 0.071 | 0.071 | 0.072 |
| | | | | 0.4 | 0.071 | 0.069 | 0.095 | 0.071 | 0.071 | 0.076 |
| | | | | 0.6 | 0.071 | 0.069 | 0.095 | 0.071 | 0.071 | 0.081 |
| 200 | 0.25 | 0.500 | 0.500 | 0.2 | 0.035 | 0.035 | 0.038 | 0.035 | 0.035 | 0.036 |
| | | | | 0.4 | 0.035 | 0.035 | 0.038 | 0.035 | 0.035 | 0.036 |
| | | | | 0.6 | 0.036 | 0.036 | 0.038 | 0.035 | 0.036 | 0.036 |
| | 0.50 | 0.500 | 0.500 | 0.2 | 0.035 | 0.035 | 0.041 | 0.035 | 0.035 | 0.036 |
| | | | | 0.4 | 0.035 | 0.035 | 0.041 | 0.035 | 0.035 | 0.036 |
| | | | | 0.6 | 0.035 | 0.035 | 0.041 | 0.035 | 0.035 | 0.037 |
| | 0.75 | 0.500 | 0.500 | 0.2 | 0.035 | 0.035 | 0.047 | 0.035 | 0.035 | 0.036 |
| | | | | 0.4 | 0.035 | 0.035 | 0.047 | 0.035 | 0.035 | 0.037 |
| | | | | 0.6 | 0.035 | 0.035 | 0.047 | 0.035 | 0.035 | 0.040 |

Table S30: RMSE of estimators when the null hypothesis is true, $\xi_g = \pi_g = 0.5$, and $G = 3$.

| | | | | | Estimator of $\xi_s$ | | | Estimator of $\pi_s$ | | |
|---|---|---|---|---|---|---|---|---|---|---|
| $N$ | $\tau$ | E[$\xi_s$] | E[$\pi_s$] | $\rho$ | MLE | CMAE | UMVCUE | MLE | CMAE | UMVCUE |
| 50 | 0.25 | 0.500 | 0.500 | 0.2 | 0.073 | 0.072 | 0.077 | 0.071 | 0.071 | 0.072 |
| | | | | 0.4 | 0.073 | 0.072 | 0.077 | 0.071 | 0.071 | 0.072 |
| | | | | 0.6 | 0.073 | 0.072 | 0.077 | 0.072 | 0.071 | 0.073 |
| | 0.50 | 0.500 | 0.500 | 0.2 | 0.075 | 0.072 | 0.086 | 0.071 | 0.071 | 0.072 |
| | | | | 0.4 | 0.075 | 0.072 | 0.086 | 0.071 | 0.071 | 0.074 |
| | | | | 0.6 | 0.075 | 0.072 | 0.086 | 0.072 | 0.071 | 0.077 |
| | 0.75 | 0.500 | 0.500 | 0.2 | 0.078 | 0.071 | 0.103 | 0.071 | 0.071 | 0.073 |
| | | | | 0.4 | 0.078 | 0.071 | 0.103 | 0.072 | 0.071 | 0.078 |
| | | | | 0.6 | 0.078 | 0.071 | 0.103 | 0.073 | 0.071 | 0.084 |
| 200 | 0.25 | 0.500 | 0.500 | 0.2 | 0.036 | 0.036 | 0.038 | 0.035 | 0.035 | 0.036 |
| | | | | 0.4 | 0.037 | 0.036 | 0.039 | 0.036 | 0.036 | 0.036 |
| | | | | 0.6 | 0.037 | 0.036 | 0.039 | 0.036 | 0.036 | 0.037 |
| | 0.50 | 0.500 | 0.500 | 0.2 | 0.038 | 0.036 | 0.043 | 0.035 | 0.035 | 0.036 |
| | | | | 0.4 | 0.038 | 0.036 | 0.043 | 0.036 | 0.035 | 0.037 |
| | | | | 0.6 | 0.038 | 0.036 | 0.043 | 0.036 | 0.036 | 0.038 |
| | 0.75 | 0.500 | 0.500 | 0.2 | 0.039 | 0.035 | 0.051 | 0.035 | 0.035 | 0.036 |
| | | | | 0.4 | 0.039 | 0.035 | 0.051 | 0.036 | 0.035 | 0.038 |
| | | | | 0.6 | 0.039 | 0.035 | 0.051 | 0.037 | 0.035 | 0.042 |

Table S31: RMSE of estimators when the null hypothesis is true, $\xi_g = \pi_g = 0.5$, and $G = 4$.

| | | | | | Estimator of $\xi_s$ | | | Estimator of $\pi_s$ | | |
|---|---|---|---|---|---|---|---|---|---|---|
| $N$ | $\tau$ | E[$\xi_s$] | E[$\pi_s$] | $\rho$ | MLE | CMAE | UMVCUE | MLE | CMAE | UMVCUE |
| 50 | 0.25 | 0.500 | 0.500 | 0.2 | 0.075 | 0.072 | 0.078 | 0.071 | 0.071 | 0.071 |
| | | | | 0.4 | 0.075 | 0.073 | 0.078 | 0.071 | 0.071 | 0.072 |
| | | | | 0.6 | 0.076 | 0.073 | 0.078 | 0.072 | 0.072 | 0.074 |
| | 0.50 | 0.500 | 0.500 | 0.2 | 0.080 | 0.073 | 0.088 | 0.071 | 0.071 | 0.072 |
| | | | | 0.4 | 0.080 | 0.073 | 0.088 | 0.073 | 0.072 | 0.075 |
| | | | | 0.6 | 0.080 | 0.072 | 0.088 | 0.074 | 0.071 | 0.078 |
| | 0.75 | 0.500 | 0.500 | 0.2 | 0.084 | 0.072 | 0.107 | 0.071 | 0.071 | 0.074 |
| | | | | 0.4 | 0.084 | 0.072 | 0.107 | 0.073 | 0.071 | 0.079 |
| | | | | 0.6 | 0.084 | 0.072 | 0.107 | 0.076 | 0.071 | 0.086 |
| 200 | 0.25 | 0.500 | 0.500 | 0.2 | 0.038 | 0.036 | 0.039 | 0.035 | 0.035 | 0.035 |
| | | | | 0.4 | 0.038 | 0.036 | 0.039 | 0.036 | 0.036 | 0.036 |
| | | | | 0.6 | 0.038 | 0.036 | 0.039 | 0.036 | 0.036 | 0.037 |
| | 0.50 | 0.500 | 0.500 | 0.2 | 0.040 | 0.036 | 0.044 | 0.036 | 0.036 | 0.036 |
| | | | | 0.4 | 0.040 | 0.036 | 0.044 | 0.036 | 0.036 | 0.037 |
| | | | | 0.6 | 0.040 | 0.036 | 0.044 | 0.037 | 0.036 | 0.039 |
| | 0.75 | 0.500 | 0.500 | 0.2 | 0.042 | 0.036 | 0.053 | 0.036 | 0.035 | 0.036 |
| | | | | 0.4 | 0.042 | 0.036 | 0.053 | 0.036 | 0.035 | 0.039 |
| | | | | 0.6 | 0.042 | 0.036 | 0.053 | 0.038 | 0.035 | 0.042 |

Table S32: RMSE of estimators when the null hypothesis is true, $\xi_g = \pi_g = 0.7$, and $G = 2$.

| | | | | | Estimator of $\xi_s$ | | | Estimator of $\pi_s$ | | |
|---|---|---|---|---|---|---|---|---|---|---|
| $N$ | $\tau$ | $\mathrm{E}[\xi_s]$ | $\mathrm{E}[\pi_s]$ | $\rho$ | MLE | CMAE | UMVCUE | MLE | CMAE | UMVCUE |
| 50 | 0.25 | 0.700 | 0.700 | 0.2 | 0.064 | 0.066 | 0.070 | 0.064 | 0.065 | 0.065 |
| | | | | 0.4 | 0.064 | 0.066 | 0.070 | 0.064 | 0.065 | 0.066 |
| | | | | 0.6 | 0.064 | 0.066 | 0.070 | 0.064 | 0.065 | 0.067 |
| | 0.50 | 0.700 | 0.700 | 0.2 | 0.064 | 0.065 | 0.076 | 0.064 | 0.065 | 0.066 |
| | | | | 0.4 | 0.064 | 0.065 | 0.077 | 0.064 | 0.065 | 0.068 |
| | | | | 0.6 | 0.064 | 0.065 | 0.077 | 0.064 | 0.065 | 0.070 |
| | 0.75 | 0.700 | 0.700 | 0.2 | 0.064 | 0.063 | 0.089 | 0.064 | 0.065 | 0.067 |
| | | | | 0.4 | 0.064 | 0.063 | 0.089 | 0.064 | 0.064 | 0.070 |
| | | | | 0.6 | 0.064 | 0.063 | 0.089 | 0.064 | 0.064 | 0.075 |
| 200 | 0.25 | 0.700 | 0.700 | 0.2 | 0.032 | 0.033 | 0.035 | 0.032 | 0.033 | 0.033 |
| | | | | 0.4 | 0.032 | 0.033 | 0.035 | 0.032 | 0.032 | 0.033 |
| | | | | 0.6 | 0.032 | 0.033 | 0.035 | 0.032 | 0.032 | 0.033 |
| | 0.50 | 0.700 | 0.700 | 0.2 | 0.032 | 0.032 | 0.038 | 0.032 | 0.032 | 0.033 |
| | | | | 0.4 | 0.032 | 0.032 | 0.038 | 0.032 | 0.032 | 0.033 |
| | | | | 0.6 | 0.032 | 0.032 | 0.038 | 0.032 | 0.032 | 0.034 |
| | 0.75 | 0.700 | 0.700 | 0.2 | 0.032 | 0.032 | 0.043 | 0.032 | 0.032 | 0.033 |
| | | | | 0.4 | 0.032 | 0.032 | 0.044 | 0.032 | 0.032 | 0.035 |
| | | | | 0.6 | 0.032 | 0.032 | 0.044 | 0.032 | 0.032 | 0.037 |

Table S33: RMSE of estimators when the null hypothesis is true, $\xi_g = \pi_g = 0.7$, and $G = 3$.

| | | | | | Estimator of $\xi_s$ | | | Estimator of $\pi_s$ | | |
|---|---|---|---|---|---|---|---|---|---|---|
| $N$ | $\tau$ | $\mathrm{E}[\xi_s]$ | $\mathrm{E}[\pi_s]$ | $\rho$ | MLE | CMAE | UMVCUE | MLE | CMAE | UMVCUE |
| 50 | 0.25 | 0.700 | 0.700 | 0.2 | 0.066 | 0.066 | 0.071 | 0.064 | 0.065 | 0.065 |
| | | | | 0.4 | 0.066 | 0.066 | 0.072 | 0.065 | 0.065 | 0.066 |
| | | | | 0.6 | 0.066 | 0.066 | 0.071 | 0.065 | 0.065 | 0.068 |
| | 0.50 | 0.700 | 0.700 | 0.2 | 0.068 | 0.066 | 0.080 | 0.065 | 0.065 | 0.066 |
| | | | | 0.4 | 0.068 | 0.066 | 0.080 | 0.065 | 0.065 | 0.068 |
| | | | | 0.6 | 0.068 | 0.066 | 0.080 | 0.065 | 0.066 | 0.072 |
| | 0.75 | 0.700 | 0.700 | 0.2 | 0.070 | 0.064 | 0.097 | 0.065 | 0.065 | 0.068 |
| | | | | 0.4 | 0.070 | 0.065 | 0.098 | 0.065 | 0.065 | 0.072 |
| | | | | 0.6 | 0.070 | 0.065 | 0.097 | 0.066 | 0.065 | 0.079 |
| 200 | 0.25 | 0.700 | 0.700 | 0.2 | 0.033 | 0.033 | 0.036 | 0.032 | 0.032 | 0.033 |
| | | | | 0.4 | 0.033 | 0.033 | 0.035 | 0.032 | 0.033 | 0.033 |
| | | | | 0.6 | 0.033 | 0.033 | 0.035 | 0.033 | 0.033 | 0.034 |
| | 0.50 | 0.700 | 0.700 | 0.2 | 0.034 | 0.033 | 0.040 | 0.032 | 0.033 | 0.033 |
| | | | | 0.4 | 0.034 | 0.033 | 0.040 | 0.033 | 0.033 | 0.034 |
| | | | | 0.6 | 0.034 | 0.033 | 0.040 | 0.033 | 0.033 | 0.035 |
| | 0.75 | 0.700 | 0.700 | 0.2 | 0.035 | 0.032 | 0.047 | 0.032 | 0.032 | 0.033 |
| | | | | 0.4 | 0.035 | 0.032 | 0.047 | 0.033 | 0.032 | 0.035 |
| | | | | 0.6 | 0.035 | 0.032 | 0.047 | 0.033 | 0.032 | 0.039 |

Table S34: RMSE of estimators when the null hypothesis is true, $\xi_g = \pi_g = 0.7$, and $G = 4$.

| | | | | | Estimator of $\xi_s$ | | | Estimator of $\pi_s$ | | |
|---|---|---|---|---|---|---|---|---|---|---|
| $N$ | $\tau$ | E[$\xi_s$] | E[$\pi_s$] | $\rho$ | MLE | CMAE | UMVCUE | MLE | CMAE | UMVCUE |
| 50 | 0.25 | 0.700 | 0.700 | 0.2 | 0.068 | 0.067 | 0.072 | 0.065 | 0.066 | 0.066 |
| | | | | 0.4 | 0.068 | 0.067 | 0.073 | 0.065 | 0.065 | 0.067 |
| | | | | 0.6 | 0.068 | 0.067 | 0.073 | 0.066 | 0.066 | 0.068 |
| | 0.50 | 0.700 | 0.700 | 0.2 | 0.072 | 0.067 | 0.082 | 0.065 | 0.066 | 0.067 |
| | | | | 0.4 | 0.072 | 0.067 | 0.082 | 0.065 | 0.065 | 0.069 |
| | | | | 0.6 | 0.072 | 0.067 | 0.082 | 0.067 | 0.066 | 0.072 |
| | 0.75 | 0.700 | 0.700 | 0.2 | 0.075 | 0.066 | 0.102 | 0.065 | 0.065 | 0.069 |
| | | | | 0.4 | 0.075 | 0.066 | 0.101 | 0.066 | 0.065 | 0.074 |
| | | | | 0.6 | 0.075 | 0.066 | 0.102 | 0.068 | 0.065 | 0.081 |
| 200 | 0.25 | 0.700 | 0.700 | 0.2 | 0.034 | 0.034 | 0.036 | 0.033 | 0.033 | 0.033 |
| | | | | 0.4 | 0.034 | 0.033 | 0.036 | 0.033 | 0.033 | 0.033 |
| | | | | 0.6 | 0.034 | 0.033 | 0.036 | 0.033 | 0.033 | 0.034 |
| | 0.50 | 0.700 | 0.700 | 0.2 | 0.036 | 0.033 | 0.041 | 0.032 | 0.032 | 0.033 |
| | | | | 0.4 | 0.036 | 0.033 | 0.040 | 0.033 | 0.033 | 0.034 |
| | | | | 0.6 | 0.036 | 0.033 | 0.040 | 0.034 | 0.033 | 0.036 |
| | 0.75 | 0.700 | 0.700 | 0.2 | 0.038 | 0.033 | 0.049 | 0.032 | 0.032 | 0.033 |
| | | | | 0.4 | 0.038 | 0.033 | 0.049 | 0.033 | 0.033 | 0.036 |
| | | | | 0.6 | 0.038 | 0.033 | 0.049 | 0.034 | 0.033 | 0.040 |

Table S35: RMSE of estimators when the null hypothesis is true, $\xi_g$ =0.3, $\pi_g = 0.5$, and $G = 2$.

| | | | | | Estimator of $\xi_s$ | | | Estimator of $\pi_s$ | | |
|---|---|---|---|---|---|---|---|---|---|---|
| $N$ | $\tau$ | E[$\xi_s$] | E[$\pi_s$] | $\rho$ | MLE | CMAE | UMVCUE | MLE | CMAE | UMVCUE |
| 50 | 0.25 | 0.300 | 0.500 | 0.2 | 0.066 | 0.065 | 0.069 | 0.071 | 0.071 | 0.071 |
| | | | | 0.4 | 0.065 | 0.065 | 0.069 | 0.071 | 0.071 | 0.072 |
| | | | | 0.5 | 0.065 | 0.065 | 0.069 | 0.071 | 0.071 | 0.072 |
| | 0.50 | 0.300 | 0.500 | 0.2 | 0.066 | 0.065 | 0.074 | 0.071 | 0.071 | 0.071 |
| | | | | 0.4 | 0.066 | 0.065 | 0.074 | 0.071 | 0.071 | 0.073 |
| | | | | 0.5 | 0.065 | 0.065 | 0.074 | 0.071 | 0.071 | 0.074 |
| | 0.75 | 0.300 | 0.500 | 0.2 | 0.066 | 0.064 | 0.084 | 0.071 | 0.071 | 0.072 |
| | | | | 0.4 | 0.066 | 0.064 | 0.084 | 0.071 | 0.071 | 0.075 |
| | | | | 0.5 | 0.066 | 0.064 | 0.084 | 0.070 | 0.071 | 0.077 |
| 200 | 0.25 | 0.300 | 0.500 | 0.2 | 0.033 | 0.033 | 0.034 | 0.035 | 0.035 | 0.035 |
| | | | | 0.4 | 0.032 | 0.033 | 0.034 | 0.035 | 0.035 | 0.036 |
| | | | | 0.5 | 0.032 | 0.032 | 0.034 | 0.035 | 0.035 | 0.036 |
| | 0.50 | 0.300 | 0.500 | 0.2 | 0.033 | 0.032 | 0.037 | 0.035 | 0.035 | 0.036 |
| | | | | 0.4 | 0.032 | 0.032 | 0.037 | 0.035 | 0.035 | 0.036 |
| | | | | 0.5 | 0.033 | 0.032 | 0.037 | 0.035 | 0.035 | 0.037 |
| | 0.75 | 0.300 | 0.500 | 0.2 | 0.033 | 0.032 | 0.042 | 0.035 | 0.035 | 0.036 |
| | | | | 0.4 | 0.033 | 0.032 | 0.042 | 0.035 | 0.035 | 0.037 |
| | | | | 0.5 | 0.033 | 0.032 | 0.042 | 0.035 | 0.035 | 0.039 |

Table S36: RMSE of estimators when the null hypothesis is true, $\xi_g$ =0.3, $\pi_g = 0.5$, and $G = 3$.

| | | | | | Estimator of $\xi_s$ | | | Estimator of $\pi_s$ | | |
|---|---|---|---|---|---|---|---|---|---|---|
| $N$ | $\tau$ | E[$\xi_s$] | E[$\pi_s$] | $\rho$ | MLE | CMAE | UMVCUE | MLE | CMAE | UMVCUE |
| 50 | 0.25 | 0.300 | 0.500 | 0.2 | 0.068 | 0.066 | 0.070 | 0.071 | 0.071 | 0.071 |
| | | | | 0.4 | 0.068 | 0.065 | 0.070 | 0.071 | 0.071 | 0.072 |
| | | | | 0.5 | 0.068 | 0.066 | 0.070 | 0.071 | 0.071 | 0.073 |
| | 0.50 | 0.300 | 0.500 | 0.2 | 0.070 | 0.066 | 0.078 | 0.071 | 0.071 | 0.072 |
| | | | | 0.4 | 0.070 | 0.066 | 0.078 | 0.071 | 0.071 | 0.074 |
| | | | | 0.5 | 0.070 | 0.066 | 0.077 | 0.071 | 0.071 | 0.075 |
| | 0.75 | 0.300 | 0.500 | 0.2 | 0.072 | 0.065 | 0.091 | 0.071 | 0.071 | 0.073 |
| | | | | 0.4 | 0.073 | 0.065 | 0.091 | 0.072 | 0.071 | 0.077 |
| | | | | 0.5 | 0.072 | 0.065 | 0.091 | 0.072 | 0.071 | 0.081 |
| 200 | 0.25 | 0.300 | 0.500 | 0.2 | 0.034 | 0.033 | 0.035 | 0.035 | 0.035 | 0.035 |
| | | | | 0.4 | 0.034 | 0.033 | 0.035 | 0.035 | 0.035 | 0.036 |
| | | | | 0.5 | 0.034 | 0.033 | 0.035 | 0.036 | 0.036 | 0.036 |
| | 0.50 | 0.300 | 0.500 | 0.2 | 0.035 | 0.033 | 0.039 | 0.036 | 0.036 | 0.036 |
| | | | | 0.4 | 0.035 | 0.033 | 0.039 | 0.036 | 0.035 | 0.037 |
| | | | | 0.5 | 0.035 | 0.033 | 0.039 | 0.036 | 0.036 | 0.038 |
| | 0.75 | 0.300 | 0.500 | 0.2 | 0.036 | 0.032 | 0.046 | 0.035 | 0.035 | 0.036 |
| | | | | 0.4 | 0.036 | 0.032 | 0.046 | 0.036 | 0.035 | 0.038 |
| | | | | 0.5 | 0.036 | 0.032 | 0.046 | 0.036 | 0.035 | 0.040 |

Table S37: RMSE of estimators when the null hypothesis is true, $\xi_g$ =0.3, $\pi_g = 0.5$, and $G = 4$.

| | | | | | Estimator of $\xi_s$ | | | Estimator of $\pi_s$ | | |
|---|---|---|---|---|---|---|---|---|---|---|
| $N$ | $\tau$ | E[$\xi_s$] | E[$\pi_s$] | $\rho$ | MLE | CMAE | UMVCUE | MLE | CMAE | UMVCUE |
| 50 | 0.25 | 0.300 | 0.500 | 0.2 | 0.070 | 0.066 | 0.071 | 0.071 | 0.071 | 0.071 |
| | | | | 0.4 | 0.070 | 0.066 | 0.071 | 0.071 | 0.071 | 0.072 |
| | | | | 0.5 | 0.070 | 0.066 | 0.071 | 0.072 | 0.071 | 0.073 |
| | 0.50 | 0.300 | 0.500 | 0.2 | 0.074 | 0.066 | 0.079 | 0.071 | 0.071 | 0.072 |
| | | | | 0.4 | 0.074 | 0.066 | 0.079 | 0.072 | 0.071 | 0.074 |
| | | | | 0.5 | 0.074 | 0.066 | 0.079 | 0.073 | 0.072 | 0.076 |
| | 0.75 | 0.300 | 0.500 | 0.2 | 0.079 | 0.066 | 0.095 | 0.071 | 0.071 | 0.074 |
| | | | | 0.4 | 0.079 | 0.066 | 0.095 | 0.073 | 0.071 | 0.078 |
| | | | | 0.5 | 0.079 | 0.066 | 0.095 | 0.074 | 0.072 | 0.082 |
| 200 | 0.25 | 0.300 | 0.500 | 0.2 | 0.035 | 0.033 | 0.035 | 0.036 | 0.035 | 0.036 |
| | | | | 0.4 | 0.035 | 0.033 | 0.035 | 0.036 | 0.036 | 0.036 |
| | | | | 0.5 | 0.035 | 0.033 | 0.035 | 0.036 | 0.036 | 0.036 |
| | 0.50 | 0.300 | 0.500 | 0.2 | 0.037 | 0.033 | 0.040 | 0.036 | 0.036 | 0.036 |
| | | | | 0.4 | 0.037 | 0.033 | 0.040 | 0.036 | 0.036 | 0.037 |
| | | | | 0.5 | 0.037 | 0.033 | 0.040 | 0.036 | 0.036 | 0.038 |
| | 0.75 | 0.300 | 0.500 | 0.2 | 0.039 | 0.033 | 0.047 | 0.036 | 0.035 | 0.036 |
| | | | | 0.4 | 0.039 | 0.033 | 0.048 | 0.036 | 0.036 | 0.039 |
| | | | | 0.5 | 0.039 | 0.033 | 0.048 | 0.037 | 0.036 | 0.040 |

Table S38: RMSE of estimators when the null hypothesis is true, $\xi_g$ =0.7, $\pi_g = 0.5$, and $G = 2$.

| | | | | | Estimator of $\xi_s$ | | | Estimator of $\pi_s$ | | |
|---|---|---|---|---|---|---|---|---|---|---|
| $N$ | $\tau$ | E[$\xi_s$] | E[$\pi_s$] | $\rho$ | MLE | CMAE | UMVCUE | MLE | CMAE | UMVCUE |
| 50 | 0.25 | 0.700 | 0.500 | 0.2 | 0.064 | 0.066 | 0.070 | 0.071 | 0.071 | 0.071 |
| | | | | 0.4 | 0.064 | 0.066 | 0.070 | 0.071 | 0.071 | 0.072 |
| | | | | 0.5 | 0.064 | 0.066 | 0.070 | 0.071 | 0.071 | 0.072 |
| | 0.50 | 0.700 | 0.500 | 0.2 | 0.064 | 0.065 | 0.076 | 0.071 | 0.071 | 0.071 |
| | | | | 0.4 | 0.064 | 0.065 | 0.076 | 0.071 | 0.070 | 0.072 |
| | | | | 0.5 | 0.064 | 0.065 | 0.076 | 0.071 | 0.071 | 0.074 |
| | 0.75 | 0.700 | 0.500 | 0.2 | 0.064 | 0.064 | 0.090 | 0.071 | 0.071 | 0.072 |
| | | | | 0.4 | 0.064 | 0.063 | 0.089 | 0.071 | 0.071 | 0.076 |
| | | | | 0.5 | 0.064 | 0.063 | 0.090 | 0.071 | 0.071 | 0.078 |
| 200 | 0.25 | 0.700 | 0.500 | 0.2 | 0.032 | 0.033 | 0.035 | 0.035 | 0.035 | 0.036 |
| | | | | 0.4 | 0.032 | 0.033 | 0.035 | 0.035 | 0.035 | 0.036 |
| | | | | 0.5 | 0.032 | 0.033 | 0.035 | 0.035 | 0.035 | 0.036 |
| | 0.50 | 0.700 | 0.500 | 0.2 | 0.032 | 0.032 | 0.038 | 0.035 | 0.035 | 0.036 |
| | | | | 0.4 | 0.032 | 0.032 | 0.038 | 0.035 | 0.035 | 0.036 |
| | | | | 0.5 | 0.032 | 0.032 | 0.038 | 0.035 | 0.035 | 0.037 |
| | 0.75 | 0.700 | 0.500 | 0.2 | 0.032 | 0.032 | 0.044 | 0.035 | 0.035 | 0.036 |
| | | | | 0.4 | 0.032 | 0.032 | 0.044 | 0.035 | 0.035 | 0.038 |
| | | | | 0.5 | 0.032 | 0.032 | 0.043 | 0.035 | 0.035 | 0.039 |

Table S39: RMSE of estimators when the null hypothesis is true, $\xi_g$ =0.7, $\pi_g = 0.5$, and $G = 3$.

| | | | | | Estimator of $\xi_s$ | | | Estimator of $\pi_s$ | | |
|---|---|---|---|---|---|---|---|---|---|---|
| $N$ | $\tau$ | E[$\xi_s$] | E[$\pi_s$] | $\rho$ | MLE | CMAE | UMVCUE | MLE | CMAE | UMVCUE |
| 50 | 0.25 | 0.700 | 0.500 | 0.2 | 0.066 | 0.067 | 0.072 | 0.071 | 0.071 | 0.071 |
| | | | | 0.4 | 0.066 | 0.066 | 0.072 | 0.071 | 0.071 | 0.072 |
| | | | | 0.5 | 0.066 | 0.066 | 0.071 | 0.071 | 0.071 | 0.072 |
| | 0.50 | 0.700 | 0.500 | 0.2 | 0.068 | 0.066 | 0.080 | 0.071 | 0.071 | 0.072 |
| | | | | 0.4 | 0.068 | 0.066 | 0.080 | 0.072 | 0.071 | 0.074 |
| | | | | 0.5 | 0.068 | 0.066 | 0.080 | 0.072 | 0.071 | 0.075 |
| | 0.75 | 0.700 | 0.500 | 0.2 | 0.070 | 0.065 | 0.097 | 0.071 | 0.071 | 0.073 |
| | | | | 0.4 | 0.070 | 0.065 | 0.097 | 0.072 | 0.071 | 0.077 |
| | | | | 0.5 | 0.070 | 0.064 | 0.097 | 0.073 | 0.071 | 0.081 |
| 200 | 0.25 | 0.700 | 0.500 | 0.2 | 0.033 | 0.033 | 0.035 | 0.035 | 0.035 | 0.035 |
| | | | | 0.4 | 0.033 | 0.033 | 0.035 | 0.036 | 0.035 | 0.036 |
| | | | | 0.5 | 0.033 | 0.033 | 0.036 | 0.036 | 0.035 | 0.036 |
| | 0.50 | 0.700 | 0.500 | 0.2 | 0.034 | 0.033 | 0.040 | 0.035 | 0.035 | 0.036 |
| | | | | 0.4 | 0.034 | 0.033 | 0.040 | 0.036 | 0.035 | 0.037 |
| | | | | 0.5 | 0.034 | 0.033 | 0.040 | 0.036 | 0.035 | 0.037 |
| | 0.75 | 0.700 | 0.500 | 0.2 | 0.035 | 0.032 | 0.047 | 0.035 | 0.035 | 0.036 |
| | | | | 0.4 | 0.035 | 0.032 | 0.047 | 0.036 | 0.035 | 0.038 |
| | | | | 0.5 | 0.035 | 0.032 | 0.047 | 0.037 | 0.035 | 0.040 |

Table S40: RMSE of estimators when the null hypothesis is true, $\xi_g$ =0.7, $\pi_g$ = 0.5, and $G = 4$.

| | | | | | Estimator of $\xi_s$ | | | Estimator of $\pi_s$ | | |
|---|---|---|---|---|---|---|---|---|---|---|
| $N$ | $\tau$ | E[$\xi_s$] | E[$\pi_s$] | $\rho$ | MLE | CMAE | UMVCUE | MLE | CMAE | UMVCUE |
| 50 | 0.25 | 0.700 | 0.500 | 0.2 | 0.068 | 0.067 | 0.072 | 0.071 | 0.071 | 0.071 |
| | | | | 0.4 | 0.068 | 0.067 | 0.072 | 0.072 | 0.071 | 0.072 |
| | | | | 0.5 | 0.068 | 0.067 | 0.073 | 0.072 | 0.071 | 0.072 |
| | 0.50 | 0.700 | 0.500 | 0.2 | 0.072 | 0.067 | 0.082 | 0.071 | 0.071 | 0.072 |
| | | | | 0.4 | 0.071 | 0.067 | 0.082 | 0.072 | 0.071 | 0.074 |
| | | | | 0.5 | 0.072 | 0.067 | 0.082 | 0.073 | 0.071 | 0.075 |
| | 0.75 | 0.700 | 0.500 | 0.2 | 0.075 | 0.066 | 0.101 | 0.071 | 0.071 | 0.073 |
| | | | | 0.4 | 0.075 | 0.066 | 0.101 | 0.073 | 0.071 | 0.079 |
| | | | | 0.5 | 0.075 | 0.066 | 0.102 | 0.075 | 0.071 | 0.082 |
| 200 | 0.25 | 0.700 | 0.500 | 0.2 | 0.034 | 0.033 | 0.036 | 0.036 | 0.036 | 0.036 |
| | | | | 0.4 | 0.034 | 0.033 | 0.036 | 0.036 | 0.035 | 0.036 |
| | | | | 0.5 | 0.034 | 0.033 | 0.036 | 0.036 | 0.036 | 0.036 |
| | 0.50 | 0.700 | 0.500 | 0.2 | 0.036 | 0.033 | 0.041 | 0.036 | 0.035 | 0.036 |
| | | | | 0.4 | 0.036 | 0.033 | 0.041 | 0.036 | 0.036 | 0.037 |
| | | | | 0.5 | 0.036 | 0.033 | 0.041 | 0.037 | 0.036 | 0.038 |
| | 0.75 | 0.700 | 0.500 | 0.2 | 0.038 | 0.033 | 0.049 | 0.036 | 0.035 | 0.036 |
| | | | | 0.4 | 0.038 | 0.033 | 0.049 | 0.037 | 0.035 | 0.039 |
| | | | | 0.5 | 0.038 | 0.033 | 0.049 | 0.037 | 0.035 | 0.040 |

Table S41: RMSE of estimators when the alternative hypothesis is true, and only one treatment is effective, and $G = 2$.

| | | | | | Estimator of $\xi_s$ | | | Estimator of $\pi_s$ | | |
|---|---|---|---|---|---|---|---|---|---|---|
| $N$ | $\tau$ | E[$\xi_s$] | E[$\pi_s$] | $\rho$ | MLE | CMAE | UMVCUE | MLE | CMAE | UMVCUE |
| 50 | 0.25 | 0.755 | 0.755 | 0.2 | 0.060 | 0.060 | 0.062 | 0.060 | 0.060 | 0.061 |
| | | | | 0.4 | 0.061 | 0.062 | 0.063 | 0.060 | 0.060 | 0.060 |
| | | | | 0.6 | 0.060 | 0.061 | 0.063 | 0.060 | 0.061 | 0.061 |
| | 0.50 | 0.773 | 0.773 | 0.2 | 0.059 | 0.061 | 0.061 | 0.058 | 0.059 | 0.059 |
| | | | | 0.4 | 0.059 | 0.060 | 0.060 | 0.058 | 0.059 | 0.059 |
| | | | | 0.6 | 0.059 | 0.061 | 0.061 | 0.059 | 0.059 | 0.059 |
| | 0.75 | 0.778 | 0.778 | 0.2 | 0.059 | 0.061 | 0.060 | 0.059 | 0.059 | 0.059 |
| | | | | 0.4 | 0.058 | 0.060 | 0.060 | 0.058 | 0.058 | 0.058 |
| | | | | 0.6 | 0.060 | 0.062 | 0.061 | 0.059 | 0.060 | 0.059 |
| 200 | 0.25 | 0.633 | 0.633 | 0.2 | 0.034 | 0.035 | 0.035 | 0.034 | 0.034 | 0.034 |
| | | | | 0.4 | 0.034 | 0.034 | 0.035 | 0.034 | 0.034 | 0.034 |
| | | | | 0.6 | 0.034 | 0.034 | 0.035 | 0.035 | 0.035 | 0.035 |
| | 0.50 | 0.642 | 0.642 | 0.2 | 0.034 | 0.035 | 0.035 | 0.034 | 0.034 | 0.034 |
| | | | | 0.4 | 0.034 | 0.035 | 0.035 | 0.034 | 0.034 | 0.034 |
| | | | | 0.6 | 0.034 | 0.035 | 0.035 | 0.034 | 0.035 | 0.035 |
| | 0.75 | 0.644 | 0.644 | 0.2 | 0.034 | 0.035 | 0.034 | 0.034 | 0.034 | 0.034 |
| | | | | 0.4 | 0.034 | 0.035 | 0.035 | 0.034 | 0.034 | 0.034 |
| | | | | 0.6 | 0.034 | 0.035 | 0.034 | 0.034 | 0.034 | 0.034 |

Table S42: RMSE of estimators when the alternative hypothesis is true, and only one treatment is effective, and $G = 3$.

| | | | | | Estimator of $\xi_s$ | | | Estimator of $\pi_s$ | | |
|---|---|---|---|---|---|---|---|---|---|---|
| $N$ | $\tau$ | $E[\xi_s]$ | $E[\pi_s]$ | $\rho$ | MLE | CMAE | UMVCUE | MLE | CMAE | UMVCUE |
| 50 | 0.25 | 0.736 | 0.736 | 0.2 | 0.062 | 0.063 | 0.064 | 0.060 | 0.061 | 0.061 |
| | | | | 0.4 | 0.063 | 0.063 | 0.065 | 0.061 | 0.062 | 0.062 |
| | | | | 0.6 | 0.062 | 0.063 | 0.065 | 0.061 | 0.062 | 0.063 |
| | 0.50 | 0.768 | 0.768 | 0.2 | 0.060 | 0.062 | 0.063 | 0.059 | 0.059 | 0.059 |
| | | | | 0.4 | 0.061 | 0.063 | 0.064 | 0.060 | 0.060 | 0.060 |
| | | | | 0.6 | 0.060 | 0.062 | 0.063 | 0.059 | 0.060 | 0.060 |
| | 0.75 | 0.777 | 0.777 | 0.2 | 0.059 | 0.062 | 0.061 | 0.059 | 0.059 | 0.059 |
| | | | | 0.4 | 0.060 | 0.063 | 0.062 | 0.059 | 0.060 | 0.060 |
| | | | | 0.6 | 0.059 | 0.062 | 0.061 | 0.058 | 0.060 | 0.059 |
| 200 | 0.25 | 0.624 | 0.624 | 0.2 | 0.035 | 0.035 | 0.036 | 0.034 | 0.034 | 0.034 |
| | | | | 0.4 | 0.034 | 0.035 | 0.035 | 0.034 | 0.034 | 0.034 |
| | | | | 0.6 | 0.035 | 0.035 | 0.036 | 0.034 | 0.034 | 0.034 |
| | 0.50 | 0.639 | 0.639 | 0.2 | 0.034 | 0.035 | 0.035 | 0.034 | 0.034 | 0.034 |
| | | | | 0.4 | 0.034 | 0.035 | 0.035 | 0.034 | 0.034 | 0.034 |
| | | | | 0.6 | 0.034 | 0.036 | 0.036 | 0.034 | 0.034 | 0.035 |
| | 0.75 | 0.643 | 0.643 | 0.2 | 0.034 | 0.036 | 0.035 | 0.034 | 0.034 | 0.034 |
| | | | | 0.4 | 0.034 | 0.036 | 0.035 | 0.034 | 0.034 | 0.034 |
| | | | | 0.6 | 0.034 | 0.036 | 0.035 | 0.034 | 0.035 | 0.034 |

Table S43: RMSE of estimators when the alternative hypothesis is true, and only one treatment is effective, and $G = 4$.

| | | | | | Estimator of $\xi_s$ | | | Estimator of $\pi_s$ | | |
|---|---|---|---|---|---|---|---|---|---|---|
| $N$ | $\tau$ | $E[\xi_s]$ | $E[\pi_s]$ | $\rho$ | MLE | CMAE | UMVCUE | MLE | CMAE | UMVCUE |
| 50 | 0.25 | 0.723 | 0.723 | 0.2 | 0.063 | 0.063 | 0.065 | 0.061 | 0.062 | 0.062 |
| | | | | 0.4 | 0.064 | 0.064 | 0.067 | 0.062 | 0.062 | 0.063 |
| | | | | 0.6 | 0.064 | 0.064 | 0.066 | 0.062 | 0.062 | 0.062 |
| | 0.50 | 0.763 | 0.763 | 0.2 | 0.061 | 0.063 | 0.064 | 0.059 | 0.059 | 0.059 |
| | | | | 0.4 | 0.061 | 0.063 | 0.064 | 0.060 | 0.061 | 0.061 |
| | | | | 0.6 | 0.061 | 0.063 | 0.064 | 0.060 | 0.061 | 0.061 |
| | 0.75 | 0.775 | 0.775 | 0.2 | 0.060 | 0.064 | 0.062 | 0.059 | 0.059 | 0.059 |
| | | | | 0.4 | 0.060 | 0.064 | 0.062 | 0.059 | 0.060 | 0.059 |
| | | | | 0.6 | 0.059 | 0.063 | 0.062 | 0.059 | 0.061 | 0.060 |
| 200 | 0.25 | 0.618 | 0.618 | 0.2 | 0.035 | 0.035 | 0.036 | 0.034 | 0.034 | 0.034 |
| | | | | 0.4 | 0.035 | 0.036 | 0.036 | 0.034 | 0.034 | 0.035 |
| | | | | 0.6 | 0.035 | 0.035 | 0.036 | 0.034 | 0.035 | 0.035 |
| | 0.50 | 0.637 | 0.637 | 0.2 | 0.034 | 0.036 | 0.036 | 0.034 | 0.034 | 0.034 |
| | | | | 0.4 | 0.034 | 0.036 | 0.036 | 0.034 | 0.034 | 0.034 |
| | | | | 0.6 | 0.034 | 0.036 | 0.036 | 0.034 | 0.035 | 0.035 |
| | 0.75 | 0.643 | 0.643 | 0.2 | 0.034 | 0.036 | 0.035 | 0.034 | 0.034 | 0.034 |
| | | | | 0.4 | 0.034 | 0.036 | 0.036 | 0.033 | 0.034 | 0.034 |
| | | | | 0.6 | 0.034 | 0.036 | 0.036 | 0.034 | 0.035 | 0.034 |

Table S44: RMSE of estimators when the alternative hypothesis is true, and relationship between the treatment group and binomial probability is linear, and $G = 2$.

| | | | | | Estimator of $\xi_s$ | | | Estimator of $\pi_s$ | | |
|---|---|---|---|---|---|---|---|---|---|---|
| $N$ | $\tau$ | $E[\xi_s]$ | $E[\pi_s]$ | $\rho$ | MLE | CMAE | UMVCUE | MLE | CMAE | UMVCUE |
| 50 | 0.25 | 0.741 | 0.741 | 0.2 | 0.060 | 0.061 | 0.065 | 0.062 | 0.062 | 0.063 |
| | | | | 0.4 | 0.061 | 0.062 | 0.066 | 0.061 | 0.062 | 0.063 |
| | | | | 0.6 | 0.062 | 0.063 | 0.067 | 0.062 | 0.062 | 0.064 |
| | 0.50 | 0.756 | 0.756 | 0.2 | 0.060 | 0.062 | 0.069 | 0.060 | 0.061 | 0.061 |
| | | | | 0.4 | 0.060 | 0.061 | 0.068 | 0.061 | 0.061 | 0.063 |
| | | | | 0.6 | 0.061 | 0.063 | 0.070 | 0.061 | 0.062 | 0.065 |
| | 0.75 | 0.765 | 0.765 | 0.2 | 0.060 | 0.063 | 0.074 | 0.059 | 0.059 | 0.060 |
| | | | | 0.4 | 0.059 | 0.061 | 0.073 | 0.059 | 0.060 | 0.062 |
| | | | | 0.6 | 0.060 | 0.062 | 0.073 | 0.060 | 0.061 | 0.066 |
| 200 | 0.25 | 0.626 | 0.626 | 0.2 | 0.035 | 0.035 | 0.037 | 0.034 | 0.034 | 0.034 |
| | | | | 0.4 | 0.034 | 0.034 | 0.036 | 0.034 | 0.034 | 0.035 |
| | | | | 0.6 | 0.034 | 0.035 | 0.036 | 0.034 | 0.034 | 0.035 |
| | 0.50 | 0.633 | 0.633 | 0.2 | 0.034 | 0.035 | 0.038 | 0.034 | 0.034 | 0.034 |
| | | | | 0.4 | 0.034 | 0.035 | 0.038 | 0.034 | 0.034 | 0.035 |
| | | | | 0.6 | 0.034 | 0.035 | 0.038 | 0.034 | 0.034 | 0.035 |
| | 0.75 | 0.637 | 0.637 | 0.2 | 0.034 | 0.035 | 0.039 | 0.034 | 0.034 | 0.034 |
| | | | | 0.4 | 0.034 | 0.035 | 0.039 | 0.034 | 0.035 | 0.035 |
| | | | | 0.6 | 0.034 | 0.035 | 0.039 | 0.034 | 0.034 | 0.036 |

Table S45: RMSE of estimators when the alternative hypothesis is true, and relationship between the treatment group and binomial probability is linear, and $G = 3$.

| | | | | | Estimator of $\xi_s$ | | | Estimator of $\pi_s$ | | |
|---|---|---|---|---|---|---|---|---|---|---|
| $N$ | $\tau$ | $E[\xi_s]$ | $E[\pi_s]$ | $\rho$ | MLE | CMAE | UMVCUE | MLE | CMAE | UMVCUE |
| 50 | 0.25 | 0.725 | 0.725 | 0.2 | 0.064 | 0.065 | 0.069 | 0.062 | 0.062 | 0.063 |
| | | | | 0.4 | 0.063 | 0.064 | 0.068 | 0.063 | 0.064 | 0.064 |
| | | | | 0.6 | 0.063 | 0.063 | 0.068 | 0.062 | 0.063 | 0.065 |
| | 0.50 | 0.744 | 0.744 | 0.2 | 0.063 | 0.063 | 0.073 | 0.060 | 0.061 | 0.062 |
| | | | | 0.4 | 0.064 | 0.063 | 0.073 | 0.061 | 0.062 | 0.064 |
| | | | | 0.6 | 0.063 | 0.063 | 0.072 | 0.061 | 0.061 | 0.065 |
| | 0.75 | 0.755 | 0.755 | 0.2 | 0.062 | 0.063 | 0.082 | 0.061 | 0.061 | 0.063 |
| | | | | 0.4 | 0.063 | 0.063 | 0.081 | 0.060 | 0.061 | 0.065 |
| | | | | 0.6 | 0.062 | 0.063 | 0.082 | 0.061 | 0.061 | 0.070 |
| 200 | 0.25 | 0.619 | 0.619 | 0.2 | 0.035 | 0.035 | 0.037 | 0.034 | 0.034 | 0.035 |
| | | | | 0.4 | 0.035 | 0.035 | 0.037 | 0.034 | 0.034 | 0.035 |
| | | | | 0.6 | 0.035 | 0.035 | 0.037 | 0.035 | 0.035 | 0.035 |
| | 0.50 | 0.628 | 0.628 | 0.2 | 0.035 | 0.035 | 0.040 | 0.034 | 0.034 | 0.035 |
| | | | | 0.4 | 0.035 | 0.035 | 0.040 | 0.034 | 0.035 | 0.035 |
| | | | | 0.6 | 0.035 | 0.035 | 0.040 | 0.035 | 0.035 | 0.036 |
| | 0.75 | 0.632 | 0.632 | 0.2 | 0.035 | 0.035 | 0.044 | 0.033 | 0.033 | 0.034 |
| | | | | 0.4 | 0.035 | 0.035 | 0.045 | 0.034 | 0.034 | 0.036 |
| | | | | 0.6 | 0.035 | 0.035 | 0.044 | 0.035 | 0.035 | 0.039 |

Table S46: RMSE of estimators when the alternative hypothesis is true, and relationship between the treatment group and binomial probability is linear, and $G = 4$.

| | | | | | Estimator of $\xi_s$ | | | Estimator of $\pi_s$ | | |
|---|---|---|---|---|---|---|---|---|---|---|
| $N$ | $\tau$ | E[$\xi_s$] | E[$\pi_s$] | $\rho$ | MLE | CMAE | UMVCUE | MLE | CMAE | UMVCUE |
| 50 | 0.25 | 0.717 | 0.717 | 0.2 | 0.066 | 0.065 | 0.070 | 0.063 | 0.064 | 0.064 |
| | | | | 0.4 | 0.066 | 0.065 | 0.070 | 0.062 | 0.063 | 0.064 |
| | | | | 0.6 | 0.066 | 0.065 | 0.069 | 0.064 | 0.064 | 0.066 |
| | 0.50 | 0.738 | 0.738 | 0.2 | 0.066 | 0.064 | 0.076 | 0.062 | 0.063 | 0.064 |
| | | | | 0.4 | 0.065 | 0.064 | 0.075 | 0.061 | 0.062 | 0.065 |
| | | | | 0.6 | 0.066 | 0.064 | 0.075 | 0.063 | 0.063 | 0.068 |
| | 0.75 | 0.750 | 0.750 | 0.2 | 0.065 | 0.063 | 0.088 | 0.061 | 0.062 | 0.064 |
| | | | | 0.4 | 0.065 | 0.063 | 0.088 | 0.062 | 0.062 | 0.068 |
| | | | | 0.6 | 0.064 | 0.063 | 0.088 | 0.062 | 0.062 | 0.073 |
| 200 | 0.25 | 0.616 | 0.616 | 0.2 | 0.036 | 0.035 | 0.038 | 0.035 | 0.035 | 0.035 |
| | | | | 0.4 | 0.036 | 0.035 | 0.037 | 0.034 | 0.034 | 0.035 |
| | | | | 0.6 | 0.036 | 0.035 | 0.037 | 0.035 | 0.035 | 0.036 |
| | 0.50 | 0.625 | 0.625 | 0.2 | 0.036 | 0.035 | 0.041 | 0.034 | 0.034 | 0.034 |
| | | | | 0.4 | 0.036 | 0.035 | 0.040 | 0.034 | 0.034 | 0.035 |
| | | | | 0.6 | 0.036 | 0.035 | 0.040 | 0.035 | 0.035 | 0.037 |
| | 0.75 | 0.630 | 0.630 | 0.2 | 0.037 | 0.035 | 0.046 | 0.034 | 0.034 | 0.035 |
| | | | | 0.4 | 0.037 | 0.036 | 0.046 | 0.034 | 0.034 | 0.037 |
| | | | | 0.6 | 0.036 | 0.035 | 0.046 | 0.035 | 0.034 | 0.039 |

Table S47: RMSE of estimators when the alternative hypothesis is true, and all treatments are effective, and $G = 2$.

| | | | | | Estimator of $\xi_s$ | | | Estimator of $\pi_s$ | | |
|---|---|---|---|---|---|---|---|---|---|---|
| $N$ | $\tau$ | E[$\xi_s$] | E[$\pi_s$] | $\rho$ | MLE | CMAE | UMVCUE | MLE | CMAE | UMVCUE |
| 50 | 0.25 | 0.780 | 0.780 | 0.2 | 0.057 | 0.059 | 0.063 | 0.058 | 0.058 | 0.059 |
| | | | | 0.4 | 0.058 | 0.060 | 0.064 | 0.059 | 0.059 | 0.060 |
| | | | | 0.6 | 0.057 | 0.059 | 0.063 | 0.058 | 0.059 | 0.060 |
| | 0.50 | 0.780 | 0.780 | 0.2 | 0.058 | 0.060 | 0.071 | 0.058 | 0.059 | 0.060 |
| | | | | 0.4 | 0.057 | 0.058 | 0.069 | 0.058 | 0.059 | 0.062 |
| | | | | 0.6 | 0.057 | 0.059 | 0.070 | 0.058 | 0.059 | 0.064 |
| | 0.75 | 0.780 | 0.780 | 0.2 | 0.057 | 0.057 | 0.081 | 0.059 | 0.059 | 0.061 |
| | | | | 0.4 | 0.057 | 0.058 | 0.082 | 0.058 | 0.059 | 0.064 |
| | | | | 0.6 | 0.057 | 0.057 | 0.081 | 0.057 | 0.058 | 0.068 |
| 200 | 0.25 | 0.645 | 0.645 | 0.2 | 0.034 | 0.034 | 0.036 | 0.034 | 0.034 | 0.034 |
| | | | | 0.4 | 0.034 | 0.034 | 0.036 | 0.034 | 0.034 | 0.035 |
| | | | | 0.6 | 0.034 | 0.034 | 0.036 | 0.034 | 0.034 | 0.035 |
| | 0.50 | 0.645 | 0.645 | 0.2 | 0.034 | 0.034 | 0.039 | 0.034 | 0.034 | 0.034 |
| | | | | 0.4 | 0.034 | 0.034 | 0.040 | 0.034 | 0.034 | 0.035 |
| | | | | 0.6 | 0.034 | 0.034 | 0.040 | 0.034 | 0.034 | 0.036 |
| | 0.75 | 0.645 | 0.645 | 0.2 | 0.034 | 0.034 | 0.046 | 0.034 | 0.034 | 0.035 |
| | | | | 0.4 | 0.033 | 0.033 | 0.045 | 0.034 | 0.034 | 0.036 |
| | | | | 0.6 | 0.034 | 0.033 | 0.045 | 0.034 | 0.033 | 0.038 |

Table S48: RMSE of estimators when the alternative hypothesis is true, and all treatments are effective, and $G = 3$.

| | | | | | Estimator of $\xi_s$ | | | Estimator of $\pi_s$ | | |
|---|---|---|---|---|---|---|---|---|---|---|
| $N$ | $\tau$ | $E[\xi_s]$ | $E[\pi_s]$ | $\rho$ | MLE | CMAE | UMVCUE | MLE | CMAE | UMVCUE |
| 50 | 0.25 | 0.780 | 0.780 | 0.2 | 0.059 | 0.060 | 0.065 | 0.059 | 0.059 | 0.060 |
| | | | | 0.4 | 0.060 | 0.061 | 0.066 | 0.058 | 0.059 | 0.060 |
| | | | | 0.6 | 0.060 | 0.060 | 0.065 | 0.059 | 0.060 | 0.062 |
| | 0.50 | 0.780 | 0.780 | 0.2 | 0.061 | 0.060 | 0.074 | 0.058 | 0.059 | 0.060 |
| | | | | 0.4 | 0.061 | 0.060 | 0.074 | 0.058 | 0.059 | 0.063 |
| | | | | 0.6 | 0.060 | 0.059 | 0.072 | 0.058 | 0.059 | 0.065 |
| | 0.75 | 0.780 | 0.780 | 0.2 | 0.062 | 0.058 | 0.090 | 0.059 | 0.059 | 0.062 |
| | | | | 0.4 | 0.063 | 0.059 | 0.090 | 0.059 | 0.059 | 0.067 |
| | | | | 0.6 | 0.063 | 0.059 | 0.090 | 0.059 | 0.059 | 0.073 |
| 200 | 0.25 | 0.645 | 0.645 | 0.2 | 0.035 | 0.035 | 0.037 | 0.034 | 0.034 | 0.034 |
| | | | | 0.4 | 0.035 | 0.035 | 0.037 | 0.034 | 0.034 | 0.035 |
| | | | | 0.6 | 0.035 | 0.035 | 0.037 | 0.034 | 0.034 | 0.035 |
| | 0.50 | 0.645 | 0.645 | 0.2 | 0.036 | 0.035 | 0.042 | 0.034 | 0.034 | 0.034 |
| | | | | 0.4 | 0.036 | 0.034 | 0.042 | 0.034 | 0.034 | 0.036 |
| | | | | 0.6 | 0.036 | 0.034 | 0.041 | 0.035 | 0.034 | 0.037 |
| | 0.75 | 0.645 | 0.645 | 0.2 | 0.037 | 0.034 | 0.050 | 0.033 | 0.033 | 0.034 |
| | | | | 0.4 | 0.037 | 0.034 | 0.049 | 0.034 | 0.034 | 0.037 |
| | | | | 0.6 | 0.037 | 0.034 | 0.049 | 0.035 | 0.034 | 0.040 |

Table S49: RMSE of estimators when the alternative hypothesis is true, and all treatments are effective, and $G = 4$.

| | | | | | Estimator of $\xi_s$ | | | Estimator of $\pi_s$ | | |
|---|---|---|---|---|---|---|---|---|---|---|
| $N$ | $\tau$ | $E[\xi_s]$ | $E[\pi_s]$ | $\rho$ | MLE | CMAE | UMVCUE | MLE | CMAE | UMVCUE |
| 50 | 0.25 | 0.780 | 0.780 | 0.2 | 0.061 | 0.061 | 0.066 | 0.059 | 0.060 | 0.060 |
| | | | | 0.4 | 0.061 | 0.061 | 0.066 | 0.058 | 0.059 | 0.060 |
| | | | | 0.6 | 0.060 | 0.061 | 0.066 | 0.058 | 0.059 | 0.062 |
| | 0.50 | 0.780 | 0.780 | 0.2 | 0.063 | 0.061 | 0.075 | 0.058 | 0.059 | 0.061 |
| | | | | 0.4 | 0.064 | 0.060 | 0.074 | 0.058 | 0.059 | 0.063 |
| | | | | 0.6 | 0.064 | 0.061 | 0.075 | 0.060 | 0.060 | 0.066 |
| | 0.75 | 0.780 | 0.780 | 0.2 | 0.067 | 0.060 | 0.093 | 0.059 | 0.060 | 0.063 |
| | | | | 0.4 | 0.067 | 0.059 | 0.094 | 0.059 | 0.060 | 0.069 |
| | | | | 0.6 | 0.067 | 0.059 | 0.093 | 0.061 | 0.060 | 0.075 |
| 200 | 0.25 | 0.645 | 0.645 | 0.2 | 0.036 | 0.035 | 0.037 | 0.033 | 0.033 | 0.033 |
| | | | | 0.4 | 0.036 | 0.035 | 0.037 | 0.034 | 0.034 | 0.035 |
| | | | | 0.6 | 0.036 | 0.035 | 0.037 | 0.034 | 0.034 | 0.035 |
| | 0.50 | 0.645 | 0.645 | 0.2 | 0.038 | 0.035 | 0.042 | 0.034 | 0.034 | 0.034 |
| | | | | 0.4 | 0.038 | 0.035 | 0.042 | 0.034 | 0.034 | 0.036 |
| | | | | 0.6 | 0.038 | 0.035 | 0.043 | 0.035 | 0.034 | 0.037 |
| | 0.75 | 0.645 | 0.645 | 0.2 | 0.040 | 0.034 | 0.051 | 0.034 | 0.034 | 0.035 |
| | | | | 0.4 | 0.040 | 0.034 | 0.051 | 0.035 | 0.034 | 0.037 |
| | | | | 0.6 | 0.040 | 0.034 | 0.051 | 0.036 | 0.034 | 0.041 |

Table S50: Type-I error rate (%) when the null hypothesis is true, $\xi_g = \pi_g = 0.1$, and $G = 2$.

| | | | | | Exact | | | Mid-$p$ | | |
|---|---|---|---|---|---|---|---|---|---|---|
| $N$ | $\tau$ | E[$\xi_s$] | E[$\pi_s$] | $\rho$ | MLE | CMAE | UMVCUE | MLE | CMAE | UMVCUE |
| 50 | 0.25 | 0.100 | 0.100 | 0.2 | 0.97 | 0.96 | 0.97 | 1.90 | 1.89 | 1.91 |
| | | | | 0.4 | 1.07 | 1.05 | 1.07 | 2.08 | 2.04 | 2.08 |
| | | | | 0.6 | 1.17 | 1.14 | 1.17 | 2.21 | 2.14 | 2.20 |
| | 0.50 | 0.100 | 0.100 | 0.2 | 1.01 | 1.00 | 1.00 | 2.00 | 1.96 | 1.99 |
| | | | | 0.4 | 1.08 | 1.04 | 1.07 | 2.08 | 2.02 | 2.06 |
| | | | | 0.6 | 1.19 | 1.12 | 1.17 | 2.21 | 2.11 | 2.18 |
| | 0.75 | 0.100 | 0.100 | 0.2 | 1.00 | 0.98 | 0.99 | 2.03 | 2.00 | 2.02 |
| | | | | 0.4 | 1.13 | 1.08 | 1.11 | 2.21 | 2.10 | 2.20 |
| | | | | 0.6 | 1.17 | 1.10 | 1.15 | 2.26 | 2.09 | 2.25 |
| 200 | 0.25 | 0.100 | 0.100 | 0.2 | 1.69 | 1.65 | 1.68 | 2.37 | 2.31 | 2.36 |
| | | | | 0.4 | 1.81 | 1.75 | 1.80 | 2.52 | 2.44 | 2.50 |
| | | | | 0.6 | 1.79 | 1.70 | 1.77 | 2.49 | 2.39 | 2.47 |
| | 0.50 | 0.100 | 0.100 | 0.2 | 1.86 | 1.81 | 1.84 | 2.65 | 2.57 | 2.61 |
| | | | | 0.4 | 1.78 | 1.68 | 1.74 | 2.47 | 2.36 | 2.43 |
| | | | | 0.6 | 1.80 | 1.66 | 1.76 | 2.52 | 2.32 | 2.46 |
| | 0.75 | 0.100 | 0.100 | 0.2 | 1.82 | 1.74 | 1.77 | 2.53 | 2.43 | 2.47 |
| | | | | 0.4 | 1.93 | 1.80 | 1.86 | 2.69 | 2.51 | 2.60 |
| | | | | 0.6 | 1.89 | 1.71 | 1.83 | 2.72 | 2.47 | 2.63 |

Table S51: Type-I error rate (%) when the null hypothesis is true, $\xi_g = \pi_g = 0.1$, and $G = 3$.

| | | | | | Exact | | | Mid-$p$ | | |
|---|---|---|---|---|---|---|---|---|---|---|
| $N$ | $\tau$ | E[$\xi_s$] | E[$\pi_s$] | $\rho$ | MLE | CMAE | UMVCUE | MLE | CMAE | UMVCUE |
| 50 | 0.25 | 0.100 | 0.100 | 0.2 | 1.06 | 1.05 | 1.06 | 2.01 | 1.96 | 2.00 |
| | | | | 0.4 | 1.10 | 1.07 | 1.09 | 2.13 | 2.04 | 2.11 |
| | | | | 0.6 | 1.23 | 1.14 | 1.21 | 2.30 | 2.14 | 2.26 |
| | 0.50 | 0.100 | 0.100 | 0.2 | 1.05 | 1.03 | 1.05 | 2.05 | 1.97 | 2.02 |
| | | | | 0.4 | 1.17 | 1.09 | 1.13 | 2.22 | 2.08 | 2.15 |
| | | | | 0.6 | 1.24 | 1.12 | 1.20 | 2.27 | 2.06 | 2.19 |
| | 0.75 | 0.100 | 0.100 | 0.2 | 1.06 | 1.03 | 1.04 | 2.05 | 1.96 | 2.01 |
| | | | | 0.4 | 1.20 | 1.10 | 1.16 | 2.30 | 2.09 | 2.20 |
| | | | | 0.6 | 1.24 | 1.10 | 1.20 | 2.29 | 2.03 | 2.22 |
| 200 | 0.25 | 0.100 | 0.100 | 0.2 | 1.72 | 1.67 | 1.71 | 2.46 | 2.38 | 2.43 |
| | | | | 0.4 | 1.88 | 1.77 | 1.85 | 2.59 | 2.46 | 2.55 |
| | | | | 0.6 | 1.82 | 1.70 | 1.79 | 2.55 | 2.35 | 2.50 |
| | 0.50 | 0.100 | 0.100 | 0.2 | 1.74 | 1.67 | 1.70 | 2.48 | 2.38 | 2.43 |
| | | | | 0.4 | 1.80 | 1.65 | 1.73 | 2.53 | 2.35 | 2.45 |
| | | | | 0.6 | 1.88 | 1.70 | 1.81 | 2.59 | 2.33 | 2.48 |
| | 0.75 | 0.100 | 0.100 | 0.2 | 1.84 | 1.74 | 1.76 | 2.60 | 2.44 | 2.47 |
| | | | | 0.4 | 1.94 | 1.74 | 1.80 | 2.78 | 2.52 | 2.60 |
| | | | | 0.6 | 1.82 | 1.57 | 1.71 | 2.59 | 2.30 | 2.44 |

Table S52: Type-I error rate (%) when the null hypothesis is true, $\xi_g = \pi_g = 0.1$, and $G = 4$.

| | | | | | Exact | | | Mid-$p$ | | |
|---|---|---|---|---|---|---|---|---|---|---|
| $N$ | $\tau$ | E[$\xi_s$] | E[$\pi_s$] | $\rho$ | MLE | CMAE | UMVCUE | MLE | CMAE | UMVCUE |
| 50 | 0.25 | 0.100 | 0.100 | 0.2 | 1.09 | 1.07 | 1.09 | 2.07 | 2.02 | 2.06 |
| | | | | 0.4 | 1.22 | 1.14 | 1.20 | 2.26 | 2.14 | 2.23 |
| | | | | 0.6 | 1.18 | 1.07 | 1.16 | 2.28 | 2.07 | 2.23 |
| | 0.50 | 0.100 | 0.100 | 0.2 | 1.15 | 1.11 | 1.13 | 2.19 | 2.11 | 2.15 |
| | | | | 0.4 | 1.15 | 1.05 | 1.10 | 2.21 | 2.02 | 2.13 |
| | | | | 0.6 | 1.26 | 1.10 | 1.20 | 2.29 | 2.04 | 2.18 |
| | 0.75 | 0.100 | 0.100 | 0.2 | 1.20 | 1.13 | 1.15 | 2.27 | 2.12 | 2.21 |
| | | | | 0.4 | 1.29 | 1.12 | 1.21 | 2.37 | 2.09 | 2.24 |
| | | | | 0.6 | 1.25 | 1.08 | 1.19 | 2.36 | 2.02 | 2.22 |
| 200 | 0.25 | 0.100 | 0.100 | 0.2 | 1.82 | 1.75 | 1.79 | 2.51 | 2.43 | 2.48 |
| | | | | 0.4 | 1.89 | 1.76 | 1.84 | 2.60 | 2.46 | 2.54 |
| | | | | 0.6 | 1.77 | 1.62 | 1.72 | 2.51 | 2.29 | 2.45 |
| | 0.50 | 0.100 | 0.100 | 0.2 | 1.81 | 1.70 | 1.74 | 2.59 | 2.44 | 2.49 |
| | | | | 0.4 | 1.84 | 1.63 | 1.72 | 2.65 | 2.40 | 2.50 |
| | | | | 0.6 | 1.89 | 1.66 | 1.77 | 2.70 | 2.35 | 2.53 |
| | 0.75 | 0.100 | 0.100 | 0.2 | 1.88 | 1.72 | 1.76 | 2.67 | 2.46 | 2.49 |
| | | | | 0.4 | 1.98 | 1.72 | 1.78 | 2.79 | 2.44 | 2.54 |
| | | | | 0.6 | 1.93 | 1.65 | 1.78 | 2.72 | 2.32 | 2.49 |

Table S53: Type-I error rate (%) when the null hypothesis is true, $\xi_g = \pi_g = 0.5$, and $G = 2$.

| | | | | | Exact | | | Mid-$p$ | | |
|---|---|---|---|---|---|---|---|---|---|---|
| $N$ | $\tau$ | E[$\xi_s$] | E[$\pi_s$] | $\rho$ | MLE | CMAE | UMVCUE | MLE | CMAE | UMVCUE |
| 50 | 0.25 | 0.500 | 0.500 | 0.2 | 1.77 | 1.68 | 1.75 | 2.36 | 2.29 | 2.34 |
| | | | | 0.4 | 1.68 | 1.57 | 1.65 | 2.24 | 2.17 | 2.23 |
| | | | | 0.6 | 1.67 | 1.58 | 1.66 | 2.36 | 2.26 | 2.34 |
| | 0.50 | 0.500 | 0.500 | 0.2 | 1.78 | 1.69 | 1.73 | 2.37 | 2.30 | 2.35 |
| | | | | 0.4 | 1.72 | 1.62 | 1.69 | 2.37 | 2.28 | 2.34 |
| | | | | 0.6 | 1.64 | 1.54 | 1.61 | 2.39 | 2.22 | 2.34 |
| | 0.75 | 0.500 | 0.500 | 0.2 | 1.80 | 1.67 | 1.72 | 2.41 | 2.34 | 2.37 |
| | | | | 0.4 | 1.75 | 1.63 | 1.70 | 2.44 | 2.30 | 2.36 |
| | | | | 0.6 | 1.80 | 1.66 | 1.75 | 2.56 | 2.36 | 2.46 |
| 200 | 0.25 | 0.500 | 0.500 | 0.2 | 2.13 | 2.05 | 2.11 | 2.62 | 2.52 | 2.60 |
| | | | | 0.4 | 2.07 | 2.00 | 2.05 | 2.56 | 2.47 | 2.55 |
| | | | | 0.6 | 2.07 | 1.93 | 2.05 | 2.58 | 2.44 | 2.56 |
| | 0.50 | 0.500 | 0.500 | 0.2 | 2.11 | 2.02 | 2.07 | 2.59 | 2.49 | 2.55 |
| | | | | 0.4 | 2.15 | 2.02 | 2.10 | 2.69 | 2.54 | 2.63 |
| | | | | 0.6 | 2.18 | 2.02 | 2.12 | 2.67 | 2.46 | 2.61 |
| | 0.75 | 0.500 | 0.500 | 0.2 | 2.08 | 1.97 | 2.01 | 2.59 | 2.48 | 2.53 |
| | | | | 0.4 | 2.21 | 2.07 | 2.14 | 2.72 | 2.53 | 2.62 |
| | | | | 0.6 | 2.12 | 1.93 | 2.02 | 2.65 | 2.39 | 2.54 |

Table S54: Type-I error rate (%) when the null hypothesis is true, $\xi_g = \pi_g = 0.5$, and $G = 3$.

| | | | | | Exact | | | Mid-$p$ | | |
|---|---|---|---|---|---|---|---|---|---|---|
| $N$ | $\tau$ | $\mathrm{E}[\xi_s]$ | $\mathrm{E}[\pi_s]$ | $\rho$ | MLE | CMAE | UMVCUE | MLE | CMAE | UMVCUE |
| 50 | 0.25 | 0.500 | 0.500 | 0.2 | 1.79 | 1.69 | 1.75 | 2.35 | 2.31 | 2.34 |
| | | | | 0.4 | 1.60 | 1.51 | 1.57 | 2.31 | 2.20 | 2.28 |
| | | | | 0.6 | 1.62 | 1.52 | 1.60 | 2.45 | 2.25 | 2.39 |
| | 0.50 | 0.500 | 0.500 | 0.2 | 1.66 | 1.54 | 1.60 | 2.31 | 2.21 | 2.25 |
| | | | | 0.4 | 1.77 | 1.63 | 1.69 | 2.52 | 2.34 | 2.42 |
| | | | | 0.6 | 1.73 | 1.54 | 1.64 | 2.61 | 2.34 | 2.50 |
| | 0.75 | 0.500 | 0.500 | 0.2 | 1.73 | 1.61 | 1.66 | 2.44 | 2.31 | 2.32 |
| | | | | 0.4 | 1.76 | 1.56 | 1.63 | 2.62 | 2.36 | 2.44 |
| | | | | 0.6 | 1.82 | 1.61 | 1.72 | 2.70 | 2.35 | 2.52 |
| 200 | 0.25 | 0.500 | 0.500 | 0.2 | 2.12 | 2.01 | 2.07 | 2.55 | 2.48 | 2.53 |
| | | | | 0.4 | 2.01 | 1.88 | 1.97 | 2.50 | 2.36 | 2.46 |
| | | | | 0.6 | 2.16 | 2.01 | 2.13 | 2.65 | 2.46 | 2.60 |
| | 0.50 | 0.500 | 0.500 | 0.2 | 2.08 | 1.95 | 1.99 | 2.54 | 2.41 | 2.47 |
| | | | | 0.4 | 2.15 | 1.97 | 2.05 | 2.67 | 2.44 | 2.54 |
| | | | | 0.6 | 2.22 | 1.96 | 2.12 | 2.71 | 2.42 | 2.59 |
| | 0.75 | 0.500 | 0.500 | 0.2 | 2.12 | 1.99 | 2.00 | 2.60 | 2.42 | 2.46 |
| | | | | 0.4 | 2.28 | 2.01 | 2.09 | 2.76 | 2.48 | 2.55 |
| | | | | 0.6 | 2.21 | 1.95 | 2.08 | 2.78 | 2.39 | 2.58 |

Table S55: Type-I error rate (%) when the null hypothesis is true, $\xi_g = \pi_g = 0.5$, and $G = 4$.

| | | | | | Exact | | | Mid-$p$ | | |
|---|---|---|---|---|---|---|---|---|---|---|
| $N$ | $\tau$ | $\mathrm{E}[\xi_s]$ | $\mathrm{E}[\pi_s]$ | $\rho$ | MLE | CMAE | UMVCUE | MLE | CMAE | UMVCUE |
| 50 | 0.25 | 0.500 | 0.500 | 0.2 | 1.72 | 1.63 | 1.68 | 2.36 | 2.29 | 2.33 |
| | | | | 0.4 | 1.60 | 1.48 | 1.55 | 2.39 | 2.25 | 2.34 |
| | | | | 0.6 | 1.68 | 1.53 | 1.63 | 2.53 | 2.30 | 2.44 |
| | 0.50 | 0.500 | 0.500 | 0.2 | 1.66 | 1.55 | 1.60 | 2.33 | 2.22 | 2.26 |
| | | | | 0.4 | 1.69 | 1.52 | 1.59 | 2.54 | 2.26 | 2.38 |
| | | | | 0.6 | 1.73 | 1.50 | 1.61 | 2.60 | 2.25 | 2.42 |
| | 0.75 | 0.500 | 0.500 | 0.2 | 1.85 | 1.70 | 1.73 | 2.50 | 2.38 | 2.37 |
| | | | | 0.4 | 1.77 | 1.54 | 1.59 | 2.63 | 2.30 | 2.37 |
| | | | | 0.6 | 1.78 | 1.49 | 1.62 | 2.67 | 2.29 | 2.46 |
| 200 | 0.25 | 0.500 | 0.500 | 0.2 | 2.09 | 1.99 | 2.05 | 2.55 | 2.47 | 2.52 |
| | | | | 0.4 | 2.10 | 1.96 | 2.05 | 2.62 | 2.40 | 2.54 |
| | | | | 0.6 | 2.06 | 1.87 | 2.01 | 2.57 | 2.32 | 2.48 |
| | 0.50 | 0.500 | 0.500 | 0.2 | 2.15 | 2.01 | 2.05 | 2.64 | 2.48 | 2.53 |
| | | | | 0.4 | 2.15 | 1.93 | 2.02 | 2.63 | 2.36 | 2.46 |
| | | | | 0.6 | 2.12 | 1.81 | 1.96 | 2.67 | 2.29 | 2.47 |
| | 0.75 | 0.500 | 0.500 | 0.2 | 2.13 | 1.96 | 1.98 | 2.65 | 2.43 | 2.46 |
| | | | | 0.4 | 2.25 | 1.94 | 2.00 | 2.76 | 2.39 | 2.46 |
| | | | | 0.6 | 2.19 | 1.82 | 1.97 | 2.76 | 2.31 | 2.50 |

Table S56: Type-I error rate (%) when the null hypothesis is true, $\xi_g = \pi_g = 0.7$, and $G = 2$.

| | | | | | Exact | | | Mid-$p$ | | |
|---|---|---|---|---|---|---|---|---|---|---|
| $N$ | $\tau$ | E[$\xi_s$] | E[$\pi_s$] | $\rho$ | MLE | CMAE | UMVCUE | MLE | CMAE | UMVCUE |
| 50 | 0.25 | 0.700 | 0.700 | 0.2 | 1.56 | 1.52 | 1.54 | 2.37 | 2.31 | 2.36 |
| | | | | 0.4 | 1.58 | 1.50 | 1.55 | 2.43 | 2.32 | 2.41 |
| | | | | 0.6 | 1.57 | 1.47 | 1.55 | 2.43 | 2.29 | 2.41 |
| | 0.50 | 0.700 | 0.700 | 0.2 | 1.60 | 1.54 | 1.58 | 2.47 | 2.36 | 2.43 |
| | | | | 0.4 | 1.61 | 1.52 | 1.57 | 2.52 | 2.37 | 2.46 |
| | | | | 0.6 | 1.64 | 1.51 | 1.59 | 2.56 | 2.34 | 2.50 |
| | 0.75 | 0.700 | 0.700 | 0.2 | 1.66 | 1.57 | 1.60 | 2.52 | 2.43 | 2.47 |
| | | | | 0.4 | 1.59 | 1.48 | 1.53 | 2.41 | 2.22 | 2.32 |
| | | | | 0.6 | 1.63 | 1.49 | 1.58 | 2.53 | 2.29 | 2.45 |
| 200 | 0.25 | 0.700 | 0.700 | 0.2 | 2.04 | 1.99 | 2.02 | 2.53 | 2.47 | 2.52 |
| | | | | 0.4 | 2.06 | 1.98 | 2.04 | 2.58 | 2.48 | 2.56 |
| | | | | 0.6 | 2.03 | 1.90 | 2.00 | 2.58 | 2.41 | 2.55 |
| | 0.50 | 0.700 | 0.700 | 0.2 | 2.04 | 1.97 | 2.00 | 2.55 | 2.46 | 2.50 |
| | | | | 0.4 | 2.10 | 1.96 | 2.04 | 2.67 | 2.47 | 2.59 |
| | | | | 0.6 | 2.06 | 1.91 | 2.02 | 2.64 | 2.42 | 2.56 |
| | 0.75 | 0.700 | 0.700 | 0.2 | 2.00 | 1.91 | 1.93 | 2.48 | 2.38 | 2.40 |
| | | | | 0.4 | 2.03 | 1.86 | 1.94 | 2.59 | 2.37 | 2.47 |
| | | | | 0.6 | 2.19 | 1.99 | 2.12 | 2.70 | 2.48 | 2.62 |

Table S57: Type-I error rate (%) when the null hypothesis is true, $\xi_g = \pi_g = 0.7$, and $G = 3$.

| | | | | | Exact | | | Mid-$p$ | | |
|---|---|---|---|---|---|---|---|---|---|---|
| $N$ | $\tau$ | E[$\xi_s$] | E[$\pi_s$] | $\rho$ | MLE | CMAE | UMVCUE | MLE | CMAE | UMVCUE |
| 50 | 0.25 | 0.700 | 0.700 | 0.2 | 1.53 | 1.47 | 1.51 | 2.40 | 2.32 | 2.36 |
| | | | | 0.4 | 1.57 | 1.47 | 1.53 | 2.44 | 2.29 | 2.39 |
| | | | | 0.6 | 1.54 | 1.42 | 1.51 | 2.38 | 2.18 | 2.33 |
| | 0.50 | 0.700 | 0.700 | 0.2 | 1.52 | 1.43 | 1.47 | 2.36 | 2.25 | 2.31 |
| | | | | 0.4 | 1.67 | 1.52 | 1.58 | 2.58 | 2.37 | 2.46 |
| | | | | 0.6 | 1.64 | 1.47 | 1.57 | 2.50 | 2.22 | 2.39 |
| | 0.75 | 0.700 | 0.700 | 0.2 | 1.63 | 1.54 | 1.56 | 2.48 | 2.32 | 2.35 |
| | | | | 0.4 | 1.68 | 1.50 | 1.57 | 2.57 | 2.29 | 2.39 |
| | | | | 0.6 | 1.68 | 1.41 | 1.57 | 2.62 | 2.26 | 2.44 |
| 200 | 0.25 | 0.700 | 0.700 | 0.2 | 2.02 | 1.96 | 1.99 | 2.54 | 2.43 | 2.49 |
| | | | | 0.4 | 2.01 | 1.89 | 1.97 | 2.48 | 2.34 | 2.43 |
| | | | | 0.6 | 1.98 | 1.81 | 1.93 | 2.51 | 2.30 | 2.46 |
| | 0.50 | 0.700 | 0.700 | 0.2 | 2.02 | 1.94 | 1.97 | 2.56 | 2.43 | 2.48 |
| | | | | 0.4 | 2.13 | 1.93 | 2.01 | 2.71 | 2.48 | 2.58 |
| | | | | 0.6 | 2.12 | 1.88 | 2.00 | 2.71 | 2.39 | 2.58 |
| | 0.75 | 0.700 | 0.700 | 0.2 | 2.08 | 1.96 | 1.96 | 2.68 | 2.50 | 2.51 |
| | | | | 0.4 | 2.23 | 1.98 | 2.06 | 2.83 | 2.52 | 2.64 |
| | | | | 0.6 | 2.19 | 1.89 | 2.03 | 2.80 | 2.39 | 2.59 |

Table S58: Type-I error rate (%) when the null hypothesis is true, $\xi_g = \pi_g = 0.7$, and $G = 4$.

| | | | | | Exact | | | Mid-$p$ | | |
|---|---|---|---|---|---|---|---|---|---|---|
| $N$ | $\tau$ | E[$\xi_s$] | E[$\pi_s$] | $\rho$ | MLE | CMAE | UMVCUE | MLE | CMAE | UMVCUE |
| 50 | 0.25 | 0.700 | 0.700 | 0.2 | 1.61 | 1.55 | 1.58 | 2.43 | 2.34 | 2.38 |
| | | | | 0.4 | 1.57 | 1.47 | 1.52 | 2.45 | 2.29 | 2.38 |
| | | | | 0.6 | 1.49 | 1.35 | 1.45 | 2.36 | 2.13 | 2.30 |
| | 0.50 | 0.700 | 0.700 | 0.2 | 1.70 | 1.61 | 1.64 | 2.49 | 2.35 | 2.40 |
| | | | | 0.4 | 1.66 | 1.49 | 1.55 | 2.57 | 2.29 | 2.40 |
| | | | | 0.6 | 1.58 | 1.34 | 1.46 | 2.46 | 2.11 | 2.31 |
| | 0.75 | 0.700 | 0.700 | 0.2 | 1.59 | 1.48 | 1.50 | 2.47 | 2.28 | 2.30 |
| | | | | 0.4 | 1.79 | 1.58 | 1.65 | 2.75 | 2.41 | 2.51 |
| | | | | 0.6 | 1.65 | 1.37 | 1.51 | 2.58 | 2.18 | 2.36 |
| 200 | 0.25 | 0.700 | 0.700 | 0.2 | 1.97 | 1.88 | 1.92 | 2.49 | 2.39 | 2.45 |
| | | | | 0.4 | 2.00 | 1.84 | 1.93 | 2.53 | 2.34 | 2.46 |
| | | | | 0.6 | 1.96 | 1.78 | 1.91 | 2.52 | 2.26 | 2.44 |
| | 0.50 | 0.700 | 0.700 | 0.2 | 2.11 | 1.98 | 2.01 | 2.69 | 2.51 | 2.56 |
| | | | | 0.4 | 2.08 | 1.86 | 1.94 | 2.66 | 2.36 | 2.46 |
| | | | | 0.6 | 2.18 | 1.88 | 2.04 | 2.74 | 2.35 | 2.55 |
| | 0.75 | 0.700 | 0.700 | 0.2 | 2.13 | 1.94 | 1.97 | 2.69 | 2.46 | 2.47 |
| | | | | 0.4 | 2.29 | 1.98 | 2.05 | 2.88 | 2.49 | 2.58 |
| | | | | 0.6 | 2.20 | 1.83 | 2.00 | 2.79 | 2.32 | 2.53 |

Table S59: Type-I error rate (%) when the null hypothesis is true, $\xi_g$ =0.3, $\pi_g = 0.5$, and $G = 2$.

| | | | | | Exact | | | Mid-$p$ | | |
|---|---|---|---|---|---|---|---|---|---|---|
| $N$ | $\tau$ | E[$\xi_s$] | E[$\pi_s$] | $\rho$ | MLE | CMAE | UMVCUE | MLE | CMAE | UMVCUE |
| 50 | 0.25 | 0.300 | 0.500 | 0.2 | 1.83 | 1.76 | 1.82 | 2.42 | 2.35 | 2.40 |
| | | | | 0.4 | 1.85 | 1.75 | 1.83 | 2.42 | 2.37 | 2.41 |
| | | | | 0.5 | 1.71 | 1.61 | 1.70 | 2.30 | 2.26 | 2.30 |
| | 0.50 | 0.300 | 0.500 | 0.2 | 1.79 | 1.69 | 1.73 | 2.34 | 2.27 | 2.32 |
| | | | | 0.4 | 1.71 | 1.60 | 1.68 | 2.40 | 2.31 | 2.37 |
| | | | | 0.5 | 1.76 | 1.68 | 1.75 | 2.48 | 2.38 | 2.45 |
| | 0.75 | 0.300 | 0.500 | 0.2 | 1.80 | 1.71 | 1.76 | 2.40 | 2.33 | 2.37 |
| | | | | 0.4 | 1.77 | 1.64 | 1.72 | 2.48 | 2.35 | 2.41 |
| | | | | 0.5 | 1.79 | 1.66 | 1.75 | 2.55 | 2.36 | 2.44 |
| 200 | 0.25 | 0.300 | 0.500 | 0.2 | 2.06 | 1.97 | 2.04 | 2.57 | 2.49 | 2.55 |
| | | | | 0.4 | 2.06 | 1.97 | 2.04 | 2.53 | 2.44 | 2.52 |
| | | | | 0.5 | 2.01 | 1.91 | 2.00 | 2.51 | 2.39 | 2.50 |
| | 0.50 | 0.300 | 0.500 | 0.2 | 2.16 | 2.05 | 2.11 | 2.66 | 2.56 | 2.62 |
| | | | | 0.4 | 2.13 | 2.01 | 2.08 | 2.60 | 2.47 | 2.55 |
| | | | | 0.5 | 2.17 | 2.04 | 2.12 | 2.67 | 2.51 | 2.61 |
| | 0.75 | 0.300 | 0.500 | 0.2 | 2.13 | 2.02 | 2.07 | 2.61 | 2.51 | 2.56 |
| | | | | 0.4 | 2.22 | 2.07 | 2.14 | 2.75 | 2.56 | 2.64 |
| | | | | 0.5 | 2.14 | 1.96 | 2.05 | 2.64 | 2.44 | 2.55 |

Table S60: Type-I error rate (%) when the null hypothesis is true, $\xi_g$ =0.3, $\pi_g = 0.5$, and $G = 3$.

| | | | | | Exact | | | Mid-$p$ | | |
|---|---|---|---|---|---|---|---|---|---|---|
| $N$ | $\tau$ | E[$\xi_s$] | E[$\pi_s$] | $\rho$ | MLE | CMAE | UMVCUE | MLE | CMAE | UMVCUE |
| 50 | 0.25 | 0.300 | 0.500 | 0.2 | 1.73 | 1.64 | 1.71 | 2.31 | 2.28 | 2.30 |
| | | | | 0.4 | 1.57 | 1.49 | 1.56 | 2.29 | 2.22 | 2.28 |
| | | | | 0.5 | 1.57 | 1.50 | 1.57 | 2.38 | 2.26 | 2.36 |
| | 0.50 | 0.300 | 0.500 | 0.2 | 1.57 | 1.45 | 1.51 | 2.20 | 2.12 | 2.16 |
| | | | | 0.4 | 1.63 | 1.51 | 1.57 | 2.40 | 2.25 | 2.32 |
| | | | | 0.5 | 1.64 | 1.50 | 1.59 | 2.47 | 2.25 | 2.37 |
| | 0.75 | 0.300 | 0.500 | 0.2 | 1.77 | 1.63 | 1.68 | 2.49 | 2.37 | 2.39 |
| | | | | 0.4 | 1.73 | 1.55 | 1.64 | 2.56 | 2.34 | 2.40 |
| | | | | 0.5 | 1.72 | 1.53 | 1.62 | 2.63 | 2.34 | 2.47 |
| 200 | 0.25 | 0.300 | 0.500 | 0.2 | 2.08 | 1.98 | 2.04 | 2.51 | 2.42 | 2.49 |
| | | | | 0.4 | 2.08 | 1.96 | 2.05 | 2.58 | 2.44 | 2.54 |
| | | | | 0.5 | 2.06 | 1.91 | 2.03 | 2.57 | 2.40 | 2.52 |
| | 0.50 | 0.300 | 0.500 | 0.2 | 2.08 | 1.97 | 2.02 | 2.54 | 2.43 | 2.48 |
| | | | | 0.4 | 2.11 | 1.93 | 2.01 | 2.59 | 2.38 | 2.48 |
| | | | | 0.5 | 2.21 | 1.96 | 2.10 | 2.76 | 2.48 | 2.62 |
| | 0.75 | 0.300 | 0.500 | 0.2 | 2.13 | 1.99 | 2.01 | 2.60 | 2.46 | 2.48 |
| | | | | 0.4 | 2.18 | 1.97 | 2.03 | 2.72 | 2.41 | 2.50 |
| | | | | 0.5 | 2.23 | 1.95 | 2.08 | 2.80 | 2.45 | 2.60 |

Table S61: Type-I error rate (%) when the null hypothesis is true, $\xi_g$ =0.3, $\pi_g = 0.5$, and $G = 4$.

| | | | | | Exact | | | Mid-$p$ | | |
|---|---|---|---|---|---|---|---|---|---|---|
| $N$ | $\tau$ | E[$\xi_s$] | E[$\pi_s$] | $\rho$ | MLE | CMAE | UMVCUE | MLE | CMAE | UMVCUE |
| 50 | 0.25 | 0.300 | 0.500 | 0.2 | 1.65 | 1.56 | 1.61 | 2.33 | 2.26 | 2.31 |
| | | | | 0.4 | 1.63 | 1.55 | 1.61 | 2.47 | 2.35 | 2.44 |
| | | | | 0.5 | 1.59 | 1.48 | 1.56 | 2.44 | 2.25 | 2.40 |
| | 0.50 | 0.300 | 0.500 | 0.2 | 1.73 | 1.62 | 1.67 | 2.38 | 2.29 | 2.32 |
| | | | | 0.4 | 1.72 | 1.57 | 1.65 | 2.50 | 2.28 | 2.37 |
| | | | | 0.5 | 1.64 | 1.46 | 1.57 | 2.50 | 2.22 | 2.38 |
| | 0.75 | 0.300 | 0.500 | 0.2 | 1.84 | 1.69 | 1.73 | 2.61 | 2.47 | 2.48 |
| | | | | 0.4 | 1.81 | 1.60 | 1.65 | 2.70 | 2.38 | 2.49 |
| | | | | 0.5 | 1.82 | 1.55 | 1.65 | 2.74 | 2.37 | 2.52 |
| 200 | 0.25 | 0.300 | 0.500 | 0.2 | 2.07 | 1.97 | 2.03 | 2.52 | 2.44 | 2.49 |
| | | | | 0.4 | 2.06 | 1.93 | 2.02 | 2.56 | 2.38 | 2.50 |
| | | | | 0.5 | 2.14 | 1.96 | 2.09 | 2.64 | 2.44 | 2.58 |
| | 0.50 | 0.300 | 0.500 | 0.2 | 2.15 | 2.00 | 2.05 | 2.66 | 2.50 | 2.55 |
| | | | | 0.4 | 2.17 | 1.95 | 2.05 | 2.66 | 2.41 | 2.52 |
| | | | | 0.5 | 2.12 | 1.88 | 2.00 | 2.64 | 2.33 | 2.45 |
| | 0.75 | 0.300 | 0.500 | 0.2 | 2.20 | 2.02 | 2.03 | 2.71 | 2.52 | 2.52 |
| | | | | 0.4 | 2.30 | 1.97 | 2.05 | 2.85 | 2.48 | 2.57 |
| | | | | 0.5 | 2.30 | 1.95 | 2.07 | 2.79 | 2.40 | 2.55 |

Table S62: Type-I error rate (%) when the null hypothesis is true, $\xi_g$ =0.7, $\pi_g = 0.5$, and $G = 2$.

| | | | | | Exact | | | Mid-$p$ | | |
|---|---|---|---|---|---|---|---|---|---|---|
| $N$ | $\tau$ | E[$\xi_s$] | E[$\pi_s$] | $\rho$ | MLE | CMAE | UMVCUE | MLE | CMAE | UMVCUE |
| 50 | 0.25 | 0.700 | 0.500 | 0.2 | 1.73 | 1.65 | 1.71 | 2.29 | 2.21 | 2.27 |
| | | | | 0.4 | 1.72 | 1.59 | 1.68 | 2.29 | 2.22 | 2.27 |
| | | | | 0.5 | 1.73 | 1.59 | 1.69 | 2.33 | 2.25 | 2.31 |
| | 0.50 | 0.700 | 0.500 | 0.2 | 1.73 | 1.65 | 1.69 | 2.33 | 2.26 | 2.30 |
| | | | | 0.4 | 1.73 | 1.62 | 1.69 | 2.36 | 2.25 | 2.30 |
| | | | | 0.5 | 1.69 | 1.56 | 1.65 | 2.37 | 2.24 | 2.31 |
| | 0.75 | 0.700 | 0.500 | 0.2 | 1.80 | 1.67 | 1.74 | 2.43 | 2.37 | 2.38 |
| | | | | 0.4 | 1.78 | 1.66 | 1.72 | 2.49 | 2.32 | 2.38 |
| | | | | 0.5 | 1.84 | 1.69 | 1.76 | 2.54 | 2.38 | 2.43 |
| 200 | 0.25 | 0.700 | 0.500 | 0.2 | 2.04 | 1.97 | 2.02 | 2.51 | 2.42 | 2.48 |
| | | | | 0.4 | 2.03 | 1.93 | 2.00 | 2.47 | 2.39 | 2.45 |
| | | | | 0.5 | 2.11 | 2.02 | 2.09 | 2.61 | 2.48 | 2.58 |
| | 0.50 | 0.700 | 0.500 | 0.2 | 2.07 | 1.97 | 2.02 | 2.52 | 2.44 | 2.48 |
| | | | | 0.4 | 2.19 | 2.04 | 2.12 | 2.69 | 2.53 | 2.62 |
| | | | | 0.5 | 2.09 | 1.95 | 2.02 | 2.56 | 2.39 | 2.49 |
| | 0.75 | 0.700 | 0.500 | 0.2 | 2.22 | 2.10 | 2.15 | 2.69 | 2.57 | 2.61 |
| | | | | 0.4 | 2.22 | 2.06 | 2.13 | 2.75 | 2.56 | 2.63 |
| | | | | 0.5 | 2.26 | 2.08 | 2.16 | 2.80 | 2.56 | 2.70 |

Table S63: Type-I error rate (%) when the null hypothesis is true, $\xi_g$ =0.7, $\pi_g = 0.5$, and $G = 3$.

| | | | | | Exact | | | Mid-$p$ | | |
|---|---|---|---|---|---|---|---|---|---|---|
| $N$ | $\tau$ | E[$\xi_s$] | E[$\pi_s$] | $\rho$ | MLE | CMAE | UMVCUE | MLE | CMAE | UMVCUE |
| 50 | 0.25 | 0.700 | 0.500 | 0.2 | 1.64 | 1.55 | 1.60 | 2.25 | 2.19 | 2.23 |
| | | | | 0.4 | 1.68 | 1.55 | 1.64 | 2.36 | 2.27 | 2.33 |
| | | | | 0.5 | 1.55 | 1.44 | 1.52 | 2.38 | 2.22 | 2.31 |
| | 0.50 | 0.700 | 0.500 | 0.2 | 1.78 | 1.67 | 1.72 | 2.45 | 2.35 | 2.39 |
| | | | | 0.4 | 1.77 | 1.63 | 1.70 | 2.56 | 2.35 | 2.43 |
| | | | | 0.5 | 1.70 | 1.54 | 1.62 | 2.57 | 2.31 | 2.41 |
| | 0.75 | 0.700 | 0.500 | 0.2 | 1.79 | 1.65 | 1.68 | 2.48 | 2.35 | 2.35 |
| | | | | 0.4 | 1.78 | 1.59 | 1.66 | 2.60 | 2.30 | 2.36 |
| | | | | 0.5 | 1.75 | 1.53 | 1.61 | 2.60 | 2.24 | 2.37 |
| 200 | 0.25 | 0.700 | 0.500 | 0.2 | 2.08 | 1.98 | 2.04 | 2.53 | 2.43 | 2.50 |
| | | | | 0.4 | 2.04 | 1.90 | 1.99 | 2.54 | 2.38 | 2.48 |
| | | | | 0.5 | 2.01 | 1.84 | 1.97 | 2.52 | 2.32 | 2.46 |
| | 0.50 | 0.700 | 0.500 | 0.2 | 2.10 | 1.98 | 2.02 | 2.58 | 2.45 | 2.51 |
| | | | | 0.4 | 2.15 | 1.95 | 2.03 | 2.69 | 2.45 | 2.54 |
| | | | | 0.5 | 2.17 | 1.93 | 2.04 | 2.70 | 2.39 | 2.54 |
| | 0.75 | 0.700 | 0.500 | 0.2 | 2.11 | 1.95 | 1.99 | 2.61 | 2.44 | 2.46 |
| | | | | 0.4 | 2.28 | 2.01 | 2.07 | 2.81 | 2.49 | 2.56 |
| | | | | 0.5 | 2.26 | 1.94 | 2.07 | 2.85 | 2.43 | 2.58 |

Table S64: Type-I error rate (%) when the null hypothesis is true, $\xi_g$ =0.7, $\pi_g = 0.5$, and $G = 4$.

| | | | | | Exact | | | Mid-$p$ | | |
|---|---|---|---|---|---|---|---|---|---|---|
| $N$ | $\tau$ | E[$\xi_s$] | E[$\pi_s$] | $\rho$ | MLE | CMAE | UMVCUE | MLE | CMAE | UMVCUE |
| 50 | 0.25 | 0.700 | 0.500 | 0.2 | 1.70 | 1.59 | 1.64 | 2.33 | 2.26 | 2.30 |
| | | | | 0.4 | 1.59 | 1.48 | 1.54 | 2.37 | 2.20 | 2.29 |
| | | | | 0.5 | 1.62 | 1.49 | 1.55 | 2.48 | 2.26 | 2.39 |
| | 0.50 | 0.700 | 0.500 | 0.2 | 1.72 | 1.56 | 1.62 | 2.44 | 2.32 | 2.35 |
| | | | | 0.4 | 1.74 | 1.55 | 1.62 | 2.59 | 2.29 | 2.38 |
| | | | | 0.5 | 1.78 | 1.57 | 1.66 | 2.65 | 2.29 | 2.41 |
| | 0.75 | 0.700 | 0.500 | 0.2 | 1.79 | 1.61 | 1.63 | 2.56 | 2.38 | 2.37 |
| | | | | 0.4 | 1.85 | 1.60 | 1.66 | 2.75 | 2.37 | 2.46 |
| | | | | 0.5 | 1.80 | 1.48 | 1.58 | 2.77 | 2.28 | 2.43 |
| 200 | 0.25 | 0.700 | 0.500 | 0.2 | 2.02 | 1.92 | 1.99 | 2.52 | 2.42 | 2.48 |
| | | | | 0.4 | 1.99 | 1.83 | 1.92 | 2.50 | 2.30 | 2.43 |
| | | | | 0.5 | 2.14 | 1.93 | 2.05 | 2.66 | 2.41 | 2.56 |
| | 0.50 | 0.700 | 0.500 | 0.2 | 2.11 | 1.96 | 2.01 | 2.58 | 2.41 | 2.46 |
| | | | | 0.4 | 2.19 | 1.93 | 2.00 | 2.70 | 2.38 | 2.48 |
| | | | | 0.5 | 2.23 | 1.91 | 2.04 | 2.77 | 2.40 | 2.52 |
| | 0.75 | 0.700 | 0.500 | 0.2 | 2.26 | 2.06 | 2.08 | 2.81 | 2.58 | 2.58 |
| | | | | 0.4 | 2.31 | 1.96 | 2.05 | 2.90 | 2.46 | 2.57 |
| | | | | 0.5 | 2.25 | 1.85 | 1.98 | 2.81 | 2.34 | 2.51 |

Table S65: Power (%) when the alternative hypothesis is true, and only one treatment is effective, and $G = 2$.

| | | | | | Exact | | | Mid-$p$ | | |
|---|---|---|---|---|---|---|---|---|---|---|
| $N$ | $\tau$ | E[$\xi_s$] | E[$\pi_s$] | $\rho$ | MLE | CMAE | UMVCUE | MLE | CMAE | UMVCUE |
| 50 | 0.25 | 0.755 | 0.755 | 0.2 | 71.9 | 71.8 | 71.9 | 76.4 | 76.2 | 76.4 |
| | | | | 0.4 | 71.7 | 71.4 | 71.7 | 76.3 | 75.8 | 76.2 |
| | | | | 0.6 | 71.1 | 70.7 | 71.0 | 75.3 | 75.0 | 75.3 |
| | 0.50 | 0.773 | 0.773 | 0.2 | 77.4 | 77.3 | 77.3 | 82.6 | 82.5 | 82.6 |
| | | | | 0.4 | 76.9 | 76.7 | 76.8 | 82.0 | 81.8 | 81.9 |
| | | | | 0.6 | 76.6 | 76.2 | 76.6 | 81.3 | 80.9 | 81.2 |
| | 0.75 | 0.778 | 0.778 | 0.2 | 77.8 | 77.8 | 77.8 | 83.3 | 83.2 | 83.3 |
| | | | | 0.4 | 78.0 | 77.8 | 78.0 | 82.8 | 82.6 | 82.8 |
| | | | | 0.6 | 77.6 | 77.3 | 77.6 | 82.4 | 82.2 | 82.4 |
| 200 | 0.25 | 0.633 | 0.633 | 0.2 | 74.4 | 74.3 | 74.4 | 76.9 | 76.7 | 76.8 |
| | | | | 0.4 | 74.2 | 74.0 | 74.1 | 76.5 | 76.3 | 76.5 |
| | | | | 0.6 | 73.8 | 73.4 | 73.7 | 76.0 | 75.7 | 75.9 |
| | 0.50 | 0.642 | 0.642 | 0.2 | 79.4 | 79.3 | 79.3 | 81.6 | 81.5 | 81.6 |
| | | | | 0.4 | 78.5 | 78.3 | 78.4 | 81.4 | 81.2 | 81.3 |
| | | | | 0.6 | 78.3 | 78.0 | 78.2 | 80.6 | 80.3 | 80.5 |
| | 0.75 | 0.644 | 0.644 | 0.2 | 80.1 | 80.1 | 80.1 | 82.9 | 82.9 | 82.9 |
| | | | | 0.4 | 80.6 | 80.4 | 80.6 | 83.2 | 83.0 | 83.2 |
| | | | | 0.6 | 80.0 | 79.8 | 80.0 | 82.3 | 82.1 | 82.3 |

Table S66: Power (%) when the alternative hypothesis is true, and only one treatment is effective, and $G = 3$.

| | | | | | Exact | | | Mid-$p$ | | |
|---|---|---|---|---|---|---|---|---|---|---|
| $N$ | $\tau$ | $E[\xi_s]$ | $E[\pi_s]$ | $\rho$ | MLE | CMAE | UMVCUE | MLE | CMAE | UMVCUE |
| 50 | 0.25 | 0.736 | 0.736 | 0.2 | 66.9 | 66.7 | 66.8 | 71.2 | 70.9 | 71.1 |
| | | | | 0.4 | 66.1 | 65.5 | 66.0 | 70.0 | 69.8 | 70.0 |
| | | | | 0.6 | 66.2 | 65.6 | 66.2 | 70.3 | 69.7 | 70.2 |
| | 0.50 | 0.768 | 0.768 | 0.2 | 75.7 | 75.6 | 75.7 | 80.2 | 80.0 | 80.2 |
| | | | | 0.4 | 74.9 | 74.5 | 74.8 | 79.4 | 79.1 | 79.3 |
| | | | | 0.6 | 74.6 | 74.0 | 74.5 | 79.1 | 78.5 | 79.0 |
| | 0.75 | 0.777 | 0.777 | 0.2 | 77.8 | 77.6 | 77.8 | 82.8 | 82.6 | 82.7 |
| | | | | 0.4 | 77.3 | 77.1 | 77.3 | 82.0 | 81.7 | 81.9 |
| | | | | 0.6 | 76.4 | 75.9 | 76.3 | 81.2 | 80.7 | 81.1 |
| 200 | 0.25 | 0.624 | 0.624 | 0.2 | 70.0 | 69.9 | 70.0 | 72.2 | 72.1 | 72.2 |
| | | | | 0.4 | 69.9 | 69.5 | 69.8 | 71.8 | 71.6 | 71.7 |
| | | | | 0.6 | 69.2 | 68.7 | 69.1 | 71.4 | 70.9 | 71.3 |
| | 0.50 | 0.639 | 0.639 | 0.2 | 77.6 | 77.4 | 77.6 | 80.1 | 80.0 | 80.0 |
| | | | | 0.4 | 77.6 | 77.2 | 77.5 | 80.0 | 79.7 | 80.0 |
| | | | | 0.6 | 78.2 | 77.8 | 78.1 | 80.5 | 80.2 | 80.4 |
| | 0.75 | 0.643 | 0.643 | 0.2 | 80.3 | 80.3 | 80.3 | 82.8 | 82.7 | 82.8 |
| | | | | 0.4 | 79.2 | 78.9 | 79.1 | 81.9 | 81.6 | 81.8 |
| | | | | 0.6 | 78.6 | 78.1 | 78.5 | 81.2 | 80.8 | 81.1 |

Table S67: Power (%) when the alternative hypothesis is true, and only one treatment is effective, and $G = 4$.

| | | | | | Exact | | | Mid-$p$ | | |
|---|---|---|---|---|---|---|---|---|---|---|
| $N$ | $\tau$ | $E[\xi_s]$ | $E[\pi_s]$ | $\rho$ | MLE | CMAE | UMVCUE | MLE | CMAE | UMVCUE |
| 50 | 0.25 | 0.723 | 0.723 | 0.2 | 63.1 | 62.9 | 63.0 | 66.5 | 66.3 | 66.4 |
| | | | | 0.4 | 62.8 | 62.3 | 62.6 | 66.5 | 66.1 | 66.4 |
| | | | | 0.6 | 62.0 | 61.2 | 61.8 | 65.8 | 65.2 | 65.7 |
| | 0.50 | 0.763 | 0.763 | 0.2 | 74.6 | 74.3 | 74.5 | 79.2 | 79.0 | 79.1 |
| | | | | 0.4 | 73.0 | 72.5 | 72.8 | 77.6 | 77.2 | 77.5 |
| | | | | 0.6 | 72.3 | 71.6 | 72.1 | 77.2 | 76.5 | 77.0 |
| | 0.75 | 0.775 | 0.775 | 0.2 | 77.9 | 77.7 | 77.9 | 82.5 | 82.3 | 82.5 |
| | | | | 0.4 | 77.3 | 77.1 | 77.3 | 82.0 | 81.7 | 81.9 |
| | | | | 0.6 | 75.1 | 74.5 | 75.0 | 80.1 | 79.4 | 79.9 |
| 200 | 0.25 | 0.618 | 0.618 | 0.2 | 66.2 | 66.0 | 66.1 | 68.4 | 68.1 | 68.3 |
| | | | | 0.4 | 66.5 | 66.1 | 66.4 | 68.4 | 68.0 | 68.3 |
| | | | | 0.6 | 65.6 | 64.9 | 65.4 | 67.8 | 67.2 | 67.6 |
| | 0.50 | 0.637 | 0.637 | 0.2 | 76.2 | 76.0 | 76.1 | 78.8 | 78.6 | 78.7 |
| | | | | 0.4 | 75.3 | 74.9 | 75.1 | 77.9 | 77.5 | 77.8 |
| | | | | 0.6 | 75.1 | 74.5 | 75.0 | 77.5 | 77.0 | 77.4 |
| | 0.75 | 0.643 | 0.643 | 0.2 | 79.2 | 79.1 | 79.2 | 81.7 | 81.5 | 81.7 |
| | | | | 0.4 | 79.2 | 78.8 | 79.1 | 81.8 | 81.4 | 81.7 |
| | | | | 0.6 | 78.2 | 77.8 | 78.1 | 80.6 | 80.2 | 80.5 |

Table S68: Power (%) when the alternative hypothesis is true, and relationship between the treatment group and binomial probability is linear, and $G = 2$.

| $N$ | $\tau$ | $E[\xi_s]$ | $E[\pi_s]$ | $\rho$ | Exact | | | Mid-$p$ | | |
|---|---|---|---|---|---|---|---|---|---|---|
| | | | | | MLE | CMAE | UMVCUE | MLE | CMAE | UMVCUE |
| 50 | 0.25 | 0.741 | 0.741 | 0.2 | 63.7 | 63.4 | 63.6 | 68.6 | 68.2 | 68.5 |
| | | | | 0.4 | 63.6 | 63.0 | 63.5 | 68.5 | 67.7 | 68.3 |
| | | | | 0.6 | 63.9 | 62.7 | 63.7 | 68.9 | 67.8 | 68.6 |
| | 0.50 | 0.756 | 0.756 | 0.2 | 70.2 | 69.7 | 69.9 | 75.2 | 74.9 | 75.1 |
| | | | | 0.4 | 69.5 | 68.6 | 69.1 | 74.1 | 73.2 | 73.7 |
| | | | | 0.6 | 68.6 | 67.3 | 68.2 | 73.6 | 72.3 | 73.2 |
| | 0.75 | 0.765 | 0.765 | 0.2 | 73.1 | 72.5 | 72.7 | 77.9 | 77.5 | 77.7 |
| | | | | 0.4 | 72.6 | 71.4 | 71.9 | 77.5 | 76.5 | 76.9 |
| | | | | 0.6 | 71.8 | 70.3 | 71.1 | 77.2 | 75.7 | 76.6 |
| 200 | 0.25 | 0.626 | 0.626 | 0.2 | 67.6 | 67.3 | 67.5 | 70.1 | 69.8 | 70.0 |
| | | | | 0.4 | 67.4 | 66.9 | 67.3 | 69.8 | 69.2 | 69.6 |
| | | | | 0.6 | 67.7 | 66.6 | 67.6 | 70.2 | 69.4 | 70.1 |
| | 0.50 | 0.633 | 0.633 | 0.2 | 73.4 | 73.0 | 73.1 | 76.3 | 75.8 | 76.0 |
| | | | | 0.4 | 72.4 | 71.5 | 72.0 | 75.0 | 74.2 | 74.7 |
| | | | | 0.6 | 72.6 | 71.2 | 72.1 | 75.4 | 74.2 | 74.9 |
| | 0.75 | 0.637 | 0.637 | 0.2 | 75.5 | 75.0 | 75.2 | 78.2 | 77.8 | 77.9 |
| | | | | 0.4 | 75.8 | 75.0 | 75.3 | 78.5 | 77.6 | 78.1 |
| | | | | 0.6 | 74.6 | 73.3 | 73.9 | 77.5 | 76.0 | 76.7 |

Table S69: Power (%) when the alternative hypothesis is true, and relationship between the treatment group and binomial probability is linear, and $G = 3$.

| $N$ | $\tau$ | $E[\xi_s]$ | $E[\pi_s]$ | $\rho$ | Exact | | | Mid-$p$ | | |
|---|---|---|---|---|---|---|---|---|---|---|
| | | | | | MLE | CMAE | UMVCUE | MLE | CMAE | UMVCUE |
| 50 | 0.25 | 0.725 | 0.725 | 0.2 | 57.7 | 57.1 | 57.5 | 62.8 | 62.1 | 62.6 |
| | | | | 0.4 | 57.9 | 56.6 | 57.5 | 62.4 | 61.4 | 62.1 |
| | | | | 0.6 | 57.4 | 55.8 | 56.9 | 63.0 | 61.4 | 62.7 |
| | 0.50 | 0.744 | 0.744 | 0.2 | 65.5 | 64.8 | 65.1 | 70.5 | 69.7 | 70.1 |
| | | | | 0.4 | 64.9 | 63.6 | 64.3 | 70.0 | 68.4 | 69.1 |
| | | | | 0.6 | 64.4 | 62.2 | 63.6 | 69.7 | 67.7 | 68.7 |
| | 0.75 | 0.755 | 0.755 | 0.2 | 69.3 | 68.5 | 68.6 | 74.4 | 73.6 | 73.8 |
| | | | | 0.4 | 68.5 | 66.7 | 67.1 | 74.2 | 72.3 | 72.8 |
| | | | | 0.6 | 68.1 | 65.5 | 66.6 | 73.5 | 71.1 | 71.9 |
| 200 | 0.25 | 0.619 | 0.619 | 0.2 | 63.2 | 62.5 | 62.9 | 65.5 | 65.0 | 65.4 |
| | | | | 0.4 | 64.1 | 63.1 | 63.8 | 66.9 | 66.0 | 66.7 |
| | | | | 0.6 | 63.2 | 62.0 | 63.0 | 65.5 | 64.5 | 65.2 |
| | 0.50 | 0.628 | 0.628 | 0.2 | 69.5 | 68.8 | 69.1 | 72.0 | 71.4 | 71.7 |
| | | | | 0.4 | 69.5 | 68.2 | 68.8 | 72.0 | 71.0 | 71.5 |
| | | | | 0.6 | 69.0 | 67.1 | 68.1 | 71.9 | 69.9 | 70.9 |
| | 0.75 | 0.632 | 0.632 | 0.2 | 72.5 | 71.6 | 71.8 | 75.0 | 74.4 | 74.4 |
| | | | | 0.4 | 73.0 | 71.2 | 71.7 | 75.8 | 74.4 | 74.6 |
| | | | | 0.6 | 72.2 | 69.8 | 70.7 | 75.1 | 73.0 | 73.5 |

Table S70: Power (%) when the alternative hypothesis is true, and relationship between the treatment group and binomial probability is linear, and $G = 4$.

| | | | | | Exact | | | Mid-$p$ | | |
|---|---|---|---|---|---|---|---|---|---|---|
| $N$ | $\tau$ | $E[\xi_s]$ | $E[\pi_s]$ | $\rho$ | MLE | CMAE | UMVCUE | MLE | CMAE | UMVCUE |
| 50 | 0.25 | 0.717 | 0.717 | 0.2 | 54.3 | 53.5 | 53.9 | 59.4 | 58.6 | 59.0 |
| | | | | 0.4 | 54.6 | 53.4 | 54.1 | 59.8 | 58.6 | 59.2 |
| | | | | 0.6 | 54.0 | 52.4 | 53.5 | 59.6 | 57.8 | 59.1 |
| | 0.50 | 0.738 | 0.738 | 0.2 | 63.2 | 62.0 | 62.4 | 68.0 | 67.0 | 67.3 |
| | | | | 0.4 | 62.6 | 60.7 | 61.4 | 67.9 | 66.0 | 66.7 |
| | | | | 0.6 | 61.0 | 58.0 | 59.5 | 66.6 | 64.0 | 65.2 |
| | 0.75 | 0.750 | 0.750 | 0.2 | 66.9 | 65.7 | 65.8 | 72.1 | 70.8 | 70.9 |
| | | | | 0.4 | 67.3 | 64.8 | 65.2 | 72.4 | 70.1 | 70.1 |
| | | | | 0.6 | 65.5 | 61.9 | 63.1 | 71.4 | 67.8 | 68.7 |
| 200 | 0.25 | 0.616 | 0.616 | 0.2 | 60.7 | 59.9 | 60.3 | 63.4 | 62.9 | 63.2 |
| | | | | 0.4 | 60.3 | 59.3 | 59.8 | 63.4 | 62.1 | 63.0 |
| | | | | 0.6 | 59.7 | 58.2 | 59.2 | 62.4 | 61.0 | 62.0 |
| | 0.50 | 0.625 | 0.625 | 0.2 | 66.6 | 65.7 | 66.0 | 69.7 | 68.8 | 69.1 |
| | | | | 0.4 | 67.2 | 65.4 | 66.0 | 70.0 | 68.6 | 69.0 |
| | | | | 0.6 | 67.2 | 65.0 | 65.9 | 70.3 | 67.7 | 68.9 |
| | 0.75 | 0.630 | 0.630 | 0.2 | 71.9 | 70.8 | 70.8 | 74.3 | 73.5 | 73.4 |
| | | | | 0.4 | 71.1 | 69.1 | 69.5 | 73.9 | 72.0 | 71.9 |
| | | | | 0.6 | 70.2 | 67.0 | 67.7 | 73.0 | 70.2 | 70.6 |

Table S71: Power (%) when the alternative hypothesis is true, and all treatments are effective, and $G = 2$.

| | | | | | Exact | | | Mid-$p$ | | |
|---|---|---|---|---|---|---|---|---|---|---|
| $N$ | $\tau$ | $E[\xi_s]$ | $E[\pi_s]$ | $\rho$ | MLE | CMAE | UMVCUE | MLE | CMAE | UMVCUE |
| 50 | 0.25 | 0.780 | 0.780 | 0.2 | 79.4 | 78.9 | 79.2 | 84.1 | 83.6 | 83.9 |
| | | | | 0.4 | 80.1 | 79.0 | 79.8 | 84.5 | 83.6 | 84.2 |
| | | | | 0.6 | 79.8 | 78.4 | 79.5 | 84.2 | 83.0 | 83.9 |
| | 0.50 | 0.780 | 0.780 | 0.2 | 78.7 | 78.0 | 78.3 | 83.7 | 82.9 | 83.3 |
| | | | | 0.4 | 79.4 | 77.8 | 78.7 | 83.7 | 82.4 | 83.2 |
| | | | | 0.6 | 78.9 | 76.5 | 78.1 | 83.9 | 81.7 | 83.2 |
| | 0.75 | 0.780 | 0.780 | 0.2 | 79.4 | 78.7 | 78.7 | 84.0 | 83.1 | 83.3 |
| | | | | 0.4 | 79.7 | 77.6 | 78.1 | 84.1 | 82.6 | 82.8 |
| | | | | 0.6 | 79.0 | 76.2 | 77.1 | 83.8 | 81.2 | 82.1 |
| 200 | 0.25 | 0.645 | 0.645 | 0.2 | 81.4 | 80.9 | 81.3 | 83.8 | 83.4 | 83.7 |
| | | | | 0.4 | 81.4 | 80.7 | 81.2 | 83.6 | 83.0 | 83.4 |
| | | | | 0.6 | 81.6 | 80.6 | 81.3 | 83.8 | 82.8 | 83.6 |
| | 0.50 | 0.645 | 0.645 | 0.2 | 81.7 | 81.1 | 81.4 | 84.1 | 83.6 | 83.8 |
| | | | | 0.4 | 81.9 | 80.9 | 81.4 | 84.2 | 83.3 | 83.8 |
| | | | | 0.6 | 81.9 | 80.2 | 81.1 | 83.9 | 82.4 | 83.2 |
| | 0.75 | 0.645 | 0.645 | 0.2 | 81.3 | 80.6 | 80.7 | 83.5 | 83.0 | 83.1 |
| | | | | 0.4 | 82.0 | 80.5 | 80.7 | 84.4 | 83.1 | 83.3 |
| | | | | 0.6 | 82.1 | 79.7 | 80.4 | 84.6 | 82.4 | 83.0 |

Table S72: Power (%) when the alternative hypothesis is true, and all treatments are effective, and $G = 3$.

| | | | | | Exact | | | Mid-$p$ | | |
|---|---|---|---|---|---|---|---|---|---|---|
| $N$ | $\tau$ | $\mathrm{E}[\xi_s]$ | $\mathrm{E}[\pi_s]$ | $\rho$ | MLE | CMAE | UMVCUE | MLE | CMAE | UMVCUE |
| 50 | 0.25 | 0.780 | 0.780 | 0.2 | 78.5 | 77.8 | 78.3 | 83.4 | 82.7 | 83.1 |
| | | | | 0.4 | 79.2 | 78.0 | 78.7 | 84.0 | 82.7 | 83.4 |
| | | | | 0.6 | 77.8 | 76.0 | 77.2 | 82.4 | 81.0 | 81.9 |
| | 0.50 | 0.780 | 0.780 | 0.2 | 79.5 | 78.4 | 78.7 | 84.5 | 83.6 | 83.9 |
| | | | | 0.4 | 78.7 | 76.8 | 77.6 | 83.6 | 81.7 | 82.4 |
| | | | | 0.6 | 78.5 | 75.1 | 76.8 | 83.3 | 80.4 | 81.7 |
| | 0.75 | 0.780 | 0.780 | 0.2 | 79.3 | 77.9 | 77.8 | 84.0 | 82.8 | 82.6 |
| | | | | 0.4 | 79.7 | 76.9 | 77.0 | 84.4 | 81.9 | 81.7 |
| | | | | 0.6 | 79.2 | 74.6 | 75.4 | 83.8 | 80.0 | 80.4 |
| 200 | 0.25 | 0.645 | 0.645 | 0.2 | 82.0 | 81.4 | 81.8 | 84.0 | 83.6 | 83.9 |
| | | | | 0.4 | 81.4 | 80.4 | 81.0 | 83.9 | 83.0 | 83.5 |
| | | | | 0.6 | 81.2 | 79.6 | 80.8 | 83.5 | 82.1 | 83.1 |
| | 0.50 | 0.645 | 0.645 | 0.2 | 81.9 | 80.9 | 81.2 | 84.0 | 83.5 | 83.6 |
| | | | | 0.4 | 81.4 | 79.7 | 80.3 | 84.0 | 82.3 | 82.8 |
| | | | | 0.6 | 81.1 | 78.4 | 79.7 | 83.4 | 81.0 | 82.0 |
| | 0.75 | 0.645 | 0.645 | 0.2 | 82.3 | 81.2 | 81.0 | 84.4 | 83.5 | 83.4 |
| | | | | 0.4 | 82.5 | 80.6 | 80.3 | 84.9 | 82.8 | 82.8 |
| | | | | 0.6 | 82.4 | 79.0 | 79.2 | 84.7 | 81.8 | 81.8 |

Table S73: Power (%) when the alternative hypothesis is true, and all treatments are effective, and $G = 4$.

| | | | | | Exact | | | Mid-$p$ | | |
|---|---|---|---|---|---|---|---|---|---|---|
| $N$ | $\tau$ | $\mathrm{E}[\xi_s]$ | $\mathrm{E}[\pi_s]$ | $\rho$ | MLE | CMAE | UMVCUE | MLE | CMAE | UMVCUE |
| 50 | 0.25 | 0.780 | 0.780 | 0.2 | 79.0 | 78.2 | 78.6 | 83.7 | 83.0 | 83.2 |
| | | | | 0.4 | 78.6 | 77.1 | 77.8 | 83.0 | 82.0 | 82.5 |
| | | | | 0.6 | 78.6 | 76.5 | 77.8 | 83.1 | 81.7 | 82.6 |
| | 0.50 | 0.780 | 0.780 | 0.2 | 78.7 | 77.5 | 77.7 | 83.4 | 82.2 | 82.4 |
| | | | | 0.4 | 79.0 | 76.2 | 77.0 | 83.9 | 81.5 | 82.1 |
| | | | | 0.6 | 77.9 | 74.4 | 75.7 | 83.0 | 79.6 | 81.0 |
| | 0.75 | 0.780 | 0.780 | 0.2 | 79.3 | 77.6 | 77.2 | 84.1 | 82.8 | 82.3 |
| | | | | 0.4 | 78.8 | 75.2 | 75.1 | 83.3 | 80.4 | 79.8 |
| | | | | 0.6 | 77.8 | 72.5 | 72.6 | 82.9 | 78.1 | 78.0 |
| 200 | 0.25 | 0.645 | 0.645 | 0.2 | 82.3 | 81.6 | 82.0 | 84.4 | 83.8 | 84.1 |
| | | | | 0.4 | 81.4 | 80.2 | 80.9 | 83.9 | 82.8 | 83.4 |
| | | | | 0.6 | 81.0 | 79.0 | 80.3 | 83.4 | 81.9 | 83.0 |
| | 0.50 | 0.645 | 0.645 | 0.2 | 81.2 | 80.3 | 80.5 | 83.7 | 82.8 | 83.0 |
| | | | | 0.4 | 82.0 | 80.1 | 80.6 | 84.2 | 82.6 | 83.1 |
| | | | | 0.6 | 81.5 | 78.6 | 79.6 | 83.9 | 81.3 | 82.1 |
| | 0.75 | 0.645 | 0.645 | 0.2 | 81.8 | 80.6 | 80.3 | 84.2 | 83.1 | 82.7 |
| | | | | 0.4 | 82.7 | 79.7 | 79.2 | 84.9 | 82.4 | 81.8 |
| | | | | 0.6 | 82.1 | 77.7 | 77.5 | 84.6 | 80.6 | 80.4 |

Table S74: Coverage probability (%) when the null hypothesis is true, $\xi_g = \pi_g = 0.1$, and $G = 2$.

| | | | | | Exact | | | Mid-$p$ | | |
|---|---|---|---|---|---|---|---|---|---|---|
| $N$ | $\tau$ | $E[\xi_s]$ | $E[\pi_s]$ | $\rho$ | MLE | CMAE | UMVCUE | MLE | CMAE | UMVCUE |
| 50 | 0.25 | 0.100 | 0.100 | 0.2 | 98.0 | 98.0 | 98.1 | 96.2 | 96.2 | 96.2 |
| | | | | 0.4 | 98.0 | 97.9 | 98.0 | 96.1 | 96.0 | 96.1 |
| | | | | 0.6 | 97.9 | 97.9 | 97.9 | 96.0 | 96.0 | 96.0 |
| | 0.50 | 0.100 | 0.100 | 0.2 | 97.9 | 97.9 | 98.0 | 96.1 | 96.0 | 96.1 |
| | | | | 0.4 | 98.0 | 98.0 | 98.0 | 96.2 | 96.1 | 96.2 |
| | | | | 0.6 | 98.0 | 97.9 | 97.9 | 96.2 | 96.0 | 96.1 |
| | 0.75 | 0.100 | 0.100 | 0.2 | 98.1 | 98.0 | 98.1 | 96.1 | 96.0 | 96.1 |
| | | | | 0.4 | 98.0 | 97.9 | 98.0 | 96.2 | 96.1 | 96.1 |
| | | | | 0.6 | 98.1 | 98.0 | 98.0 | 96.2 | 96.1 | 96.1 |
| 200 | 0.25 | 0.100 | 0.100 | 0.2 | 96.7 | 96.7 | 96.7 | 95.3 | 95.3 | 95.3 |
| | | | | 0.4 | 96.6 | 96.6 | 96.6 | 95.3 | 95.2 | 95.3 |
| | | | | 0.6 | 96.7 | 96.6 | 96.7 | 95.3 | 95.2 | 95.3 |
| | 0.50 | 0.100 | 0.100 | 0.2 | 96.5 | 96.5 | 96.5 | 95.1 | 95.1 | 95.1 |
| | | | | 0.4 | 96.8 | 96.8 | 96.8 | 95.5 | 95.4 | 95.5 |
| | | | | 0.6 | 96.9 | 96.8 | 96.9 | 95.6 | 95.5 | 95.5 |
| | 0.75 | 0.100 | 0.100 | 0.2 | 96.6 | 96.6 | 96.6 | 95.3 | 95.3 | 95.3 |
| | | | | 0.4 | 96.7 | 96.6 | 96.6 | 95.3 | 95.3 | 95.2 |
| | | | | 0.6 | 96.9 | 96.9 | 96.8 | 95.6 | 95.5 | 95.4 |

Table S75: Coverage probability (%) when the null hypothesis is true, $\xi_g = \pi_g = 0.1$, and $G = 3$.

| | | | | | Exact | | | Mid-$p$ | | |
|---|---|---|---|---|---|---|---|---|---|---|
| $N$ | $\tau$ | $E[\xi_s]$ | $E[\pi_s]$ | $\rho$ | MLE | CMAE | UMVCUE | MLE | CMAE | UMVCUE |
| 50 | 0.25 | 0.100 | 0.100 | 0.2 | 97.9 | 97.8 | 97.9 | 96.0 | 96.0 | 96.1 |
| | | | | 0.4 | 97.9 | 97.8 | 97.9 | 96.0 | 95.9 | 96.0 |
| | | | | 0.6 | 97.8 | 97.8 | 97.8 | 95.9 | 95.9 | 95.9 |
| | 0.50 | 0.100 | 0.100 | 0.2 | 97.9 | 97.9 | 98.0 | 96.0 | 96.0 | 96.1 |
| | | | | 0.4 | 97.9 | 97.9 | 97.9 | 96.1 | 95.9 | 96.0 |
| | | | | 0.6 | 98.0 | 97.9 | 97.9 | 96.2 | 96.0 | 96.1 |
| | 0.75 | 0.100 | 0.100 | 0.2 | 97.9 | 97.9 | 97.9 | 96.1 | 96.0 | 96.0 |
| | | | | 0.4 | 98.0 | 97.8 | 97.9 | 96.1 | 96.0 | 95.9 |
| | | | | 0.6 | 98.1 | 98.0 | 97.9 | 96.4 | 96.2 | 96.1 |
| 200 | 0.25 | 0.100 | 0.100 | 0.2 | 96.7 | 96.6 | 96.7 | 95.3 | 95.2 | 95.3 |
| | | | | 0.4 | 96.6 | 96.5 | 96.6 | 95.2 | 95.1 | 95.2 |
| | | | | 0.6 | 96.6 | 96.6 | 96.6 | 95.3 | 95.2 | 95.3 |
| | 0.50 | 0.100 | 0.100 | 0.2 | 96.7 | 96.6 | 96.6 | 95.3 | 95.2 | 95.2 |
| | | | | 0.4 | 96.8 | 96.7 | 96.7 | 95.4 | 95.3 | 95.3 |
| | | | | 0.6 | 96.8 | 96.7 | 96.7 | 95.6 | 95.4 | 95.4 |
| | 0.75 | 0.100 | 0.100 | 0.2 | 96.7 | 96.6 | 96.6 | 95.2 | 95.2 | 95.2 |
| | | | | 0.4 | 96.7 | 96.7 | 96.5 | 95.3 | 95.2 | 95.1 |
| | | | | 0.6 | 97.1 | 97.0 | 96.8 | 95.9 | 95.6 | 95.4 |

Table S76: Coverage probability (%) when the null hypothesis is true, $\xi_g = \pi_g = 0.1$, and $G = 4$.

| | | | | | Exact | | | Mid-$p$ | | |
|---|---|---|---|---|---|---|---|---|---|---|
| $N$ | $\tau$ | E[$\xi_s$] | E[$\pi_s$] | $\rho$ | MLE | CMAE | UMVCUE | MLE | CMAE | UMVCUE |
| 50 | 0.25 | 0.100 | 0.100 | 0.2 | 97.8 | 97.8 | 97.9 | 95.9 | 95.8 | 95.9 |
| | | | | 0.4 | 97.8 | 97.7 | 97.8 | 95.9 | 95.8 | 95.9 |
| | | | | 0.6 | 97.9 | 97.7 | 97.9 | 95.9 | 95.8 | 95.9 |
| | 0.50 | 0.100 | 0.100 | 0.2 | 97.8 | 97.7 | 97.8 | 95.9 | 95.7 | 95.8 |
| | | | | 0.4 | 97.9 | 97.8 | 97.8 | 96.1 | 95.8 | 95.9 |
| | | | | 0.6 | 97.9 | 97.7 | 97.8 | 96.2 | 95.8 | 95.9 |
| | 0.75 | 0.100 | 0.100 | 0.2 | 97.8 | 97.7 | 97.7 | 95.9 | 95.8 | 95.7 |
| | | | | 0.4 | 97.9 | 97.7 | 97.6 | 96.1 | 95.9 | 95.7 |
| | | | | 0.6 | 98.1 | 97.9 | 97.7 | 96.4 | 96.1 | 95.9 |
| 200 | 0.25 | 0.100 | 0.100 | 0.2 | 96.5 | 96.5 | 96.5 | 95.2 | 95.1 | 95.2 |
| | | | | 0.4 | 96.6 | 96.5 | 96.5 | 95.2 | 95.1 | 95.2 |
| | | | | 0.6 | 96.9 | 96.8 | 96.8 | 95.5 | 95.3 | 95.4 |
| | 0.50 | 0.100 | 0.100 | 0.2 | 96.6 | 96.5 | 96.5 | 95.1 | 95.1 | 95.1 |
| | | | | 0.4 | 96.8 | 96.7 | 96.7 | 95.3 | 95.2 | 95.2 |
| | | | | 0.6 | 96.9 | 96.7 | 96.7 | 95.5 | 95.3 | 95.3 |
| | 0.75 | 0.100 | 0.100 | 0.2 | 96.6 | 96.6 | 96.6 | 95.2 | 95.2 | 95.1 |
| | | | | 0.4 | 96.8 | 96.7 | 96.5 | 95.4 | 95.3 | 95.1 |
| | | | | 0.6 | 97.2 | 96.9 | 96.6 | 95.9 | 95.6 | 95.2 |

Table S77: Coverage probability (%) when the null hypothesis is true, $\xi_g = \pi_g = 0.5$, and $G = 2$.

| | | | | | Exact | | | Mid-$p$ | | |
|---|---|---|---|---|---|---|---|---|---|---|
| $N$ | $\tau$ | E[$\xi_s$] | E[$\pi_s$] | $\rho$ | MLE | CMAE | UMVCUE | MLE | CMAE | UMVCUE |
| 50 | 0.25 | 0.500 | 0.500 | 0.2 | 96.7 | 96.7 | 96.7 | 95.2 | 95.2 | 95.2 |
| | | | | 0.4 | 96.9 | 96.9 | 96.9 | 95.4 | 95.4 | 95.4 |
| | | | | 0.6 | 97.0 | 96.9 | 96.9 | 95.5 | 95.4 | 95.5 |
| | 0.50 | 0.500 | 0.500 | 0.2 | 96.7 | 96.7 | 96.6 | 95.1 | 95.1 | 95.1 |
| | | | | 0.4 | 96.9 | 96.8 | 96.8 | 95.4 | 95.3 | 95.3 |
| | | | | 0.6 | 97.1 | 97.0 | 97.0 | 95.7 | 95.6 | 95.6 |
| | 0.75 | 0.500 | 0.500 | 0.2 | 96.8 | 96.9 | 96.8 | 95.3 | 95.3 | 95.2 |
| | | | | 0.4 | 97.0 | 97.0 | 96.9 | 95.6 | 95.5 | 95.4 |
| | | | | 0.6 | 97.1 | 97.0 | 96.9 | 95.7 | 95.6 | 95.5 |
| 200 | 0.25 | 0.500 | 0.500 | 0.2 | 96.0 | 96.0 | 96.0 | 95.1 | 95.1 | 95.1 |
| | | | | 0.4 | 96.2 | 96.1 | 96.1 | 95.2 | 95.1 | 95.2 |
| | | | | 0.6 | 96.1 | 96.1 | 96.1 | 95.2 | 95.1 | 95.1 |
| | 0.50 | 0.500 | 0.500 | 0.2 | 96.1 | 96.1 | 96.1 | 95.2 | 95.1 | 95.1 |
| | | | | 0.4 | 96.1 | 96.1 | 96.1 | 95.1 | 95.1 | 95.1 |
| | | | | 0.6 | 96.1 | 96.0 | 96.1 | 95.2 | 95.1 | 95.1 |
| | 0.75 | 0.500 | 0.500 | 0.2 | 96.1 | 96.1 | 96.1 | 95.2 | 95.1 | 95.1 |
| | | | | 0.4 | 96.2 | 96.2 | 96.1 | 95.3 | 95.3 | 95.2 |
| | | | | 0.6 | 96.4 | 96.3 | 96.2 | 95.5 | 95.4 | 95.3 |

Table S78: Coverage probability (%) when the null hypothesis is true, $\xi_g = \pi_g = 0.5$, and $G = 3$.

| | | | | | Exact | | | Mid-$p$ | | |
|---|---|---|---|---|---|---|---|---|---|---|
| $N$ | $\tau$ | E[$\xi_s$] | E[$\pi_s$] | $\rho$ | MLE | CMAE | UMVCUE | MLE | CMAE | UMVCUE |
| 50 | 0.25 | 0.500 | 0.500 | 0.2 | 96.7 | 96.7 | 96.7 | 95.2 | 95.2 | 95.2 |
| | | | | 0.4 | 96.9 | 96.8 | 96.9 | 95.4 | 95.4 | 95.4 |
| | | | | 0.6 | 96.9 | 96.8 | 96.9 | 95.4 | 95.2 | 95.3 |
| | 0.50 | 0.500 | 0.500 | 0.2 | 96.9 | 96.9 | 96.9 | 95.4 | 95.4 | 95.4 |
| | | | | 0.4 | 96.9 | 96.8 | 96.8 | 95.4 | 95.3 | 95.3 |
| | | | | 0.6 | 97.1 | 96.9 | 97.0 | 95.6 | 95.4 | 95.4 |
| | 0.75 | 0.500 | 0.500 | 0.2 | 96.9 | 96.8 | 96.7 | 95.3 | 95.3 | 95.2 |
| | | | | 0.4 | 97.1 | 97.0 | 96.8 | 95.6 | 95.4 | 95.2 |
| | | | | 0.6 | 97.2 | 97.0 | 96.8 | 95.8 | 95.6 | 95.3 |
| 200 | 0.25 | 0.500 | 0.500 | 0.2 | 95.9 | 95.9 | 95.9 | 95.0 | 95.0 | 95.0 |
| | | | | 0.4 | 96.2 | 96.1 | 96.2 | 95.2 | 95.2 | 95.2 |
| | | | | 0.6 | 96.0 | 95.9 | 96.0 | 95.1 | 95.0 | 95.0 |
| | 0.50 | 0.500 | 0.500 | 0.2 | 96.1 | 96.1 | 96.1 | 95.2 | 95.2 | 95.2 |
| | | | | 0.4 | 96.2 | 96.1 | 96.1 | 95.2 | 95.1 | 95.1 |
| | | | | 0.6 | 96.2 | 96.1 | 96.1 | 95.3 | 95.1 | 95.1 |
| | 0.75 | 0.500 | 0.500 | 0.2 | 96.2 | 96.2 | 96.1 | 95.3 | 95.3 | 95.2 |
| | | | | 0.4 | 96.2 | 96.1 | 96.0 | 95.3 | 95.2 | 95.0 |
| | | | | 0.6 | 96.5 | 96.3 | 96.1 | 95.6 | 95.4 | 95.1 |

Table S79: Coverage probability (%) when the null hypothesis is true, $\xi_g = \pi_g = 0.5$, and $G = 4$.

| | | | | | Exact | | | Mid-$p$ | | |
|---|---|---|---|---|---|---|---|---|---|---|
| $N$ | $\tau$ | E[$\xi_s$] | E[$\pi_s$] | $\rho$ | MLE | CMAE | UMVCUE | MLE | CMAE | UMVCUE |
| 50 | 0.25 | 0.500 | 0.500 | 0.2 | 96.8 | 96.8 | 96.8 | 95.3 | 95.3 | 95.3 |
| | | | | 0.4 | 96.9 | 96.9 | 96.9 | 95.5 | 95.3 | 95.4 |
| | | | | 0.6 | 96.9 | 96.8 | 96.9 | 95.4 | 95.2 | 95.3 |
| | 0.50 | 0.500 | 0.500 | 0.2 | 96.9 | 96.9 | 96.8 | 95.5 | 95.4 | 95.4 |
| | | | | 0.4 | 97.0 | 96.8 | 96.8 | 95.5 | 95.3 | 95.3 |
| | | | | 0.6 | 97.1 | 97.0 | 97.0 | 95.7 | 95.4 | 95.4 |
| | 0.75 | 0.500 | 0.500 | 0.2 | 96.8 | 96.7 | 96.6 | 95.3 | 95.2 | 95.1 |
| | | | | 0.4 | 97.1 | 97.0 | 96.8 | 95.7 | 95.5 | 95.2 |
| | | | | 0.6 | 97.3 | 97.1 | 96.8 | 95.9 | 95.6 | 95.2 |
| 200 | 0.25 | 0.500 | 0.500 | 0.2 | 96.0 | 96.0 | 96.0 | 95.1 | 95.1 | 95.1 |
| | | | | 0.4 | 96.1 | 96.0 | 96.0 | 95.1 | 95.1 | 95.1 |
| | | | | 0.6 | 96.2 | 96.1 | 96.2 | 95.3 | 95.1 | 95.2 |
| | 0.50 | 0.500 | 0.500 | 0.2 | 96.0 | 95.9 | 95.9 | 95.0 | 95.0 | 95.0 |
| | | | | 0.4 | 96.2 | 96.1 | 96.0 | 95.3 | 95.2 | 95.1 |
| | | | | 0.6 | 96.3 | 96.2 | 96.2 | 95.4 | 95.2 | 95.2 |
| | 0.75 | 0.500 | 0.500 | 0.2 | 96.2 | 96.2 | 96.1 | 95.3 | 95.2 | 95.1 |
| | | | | 0.4 | 96.2 | 96.1 | 95.9 | 95.3 | 95.2 | 94.9 |
| | | | | 0.6 | 96.6 | 96.4 | 96.0 | 95.7 | 95.4 | 95.0 |

Table S80: Coverage probability (%) when the null hypothesis is true, $\xi_g = \pi_g = 0.7$, and $G = 2$.

| | | | | | Exact | | | Mid-$p$ | | |
|---|---|---|---|---|---|---|---|---|---|---|
| $N$ | $\tau$ | $E[\xi_s]$ | $E[\pi_s]$ | $\rho$ | MLE | CMAE | UMVCUE | MLE | CMAE | UMVCUE |
| 50 | 0.25 | 0.700 | 0.700 | 0.2 | 97.0 | 97.0 | 97.0 | 95.5 | 95.5 | 95.5 |
| | | | | 0.4 | 97.0 | 97.0 | 97.0 | 95.4 | 95.3 | 95.3 |
| | | | | 0.6 | 97.1 | 97.0 | 97.1 | 95.4 | 95.4 | 95.4 |
| | 0.50 | 0.700 | 0.700 | 0.2 | 96.9 | 96.9 | 96.9 | 95.3 | 95.3 | 95.3 |
| | | | | 0.4 | 97.0 | 96.9 | 97.0 | 95.3 | 95.2 | 95.3 |
| | | | | 0.6 | 97.2 | 97.1 | 97.1 | 95.6 | 95.4 | 95.5 |
| | 0.75 | 0.700 | 0.700 | 0.2 | 97.0 | 97.0 | 97.0 | 95.4 | 95.4 | 95.3 |
| | | | | 0.4 | 97.2 | 97.1 | 97.0 | 95.6 | 95.6 | 95.5 |
| | | | | 0.6 | 97.3 | 97.2 | 97.1 | 95.7 | 95.6 | 95.5 |
| 200 | 0.25 | 0.700 | 0.700 | 0.2 | 96.0 | 96.0 | 96.0 | 95.0 | 95.0 | 95.0 |
| | | | | 0.4 | 96.1 | 96.1 | 96.1 | 95.1 | 95.1 | 95.1 |
| | | | | 0.6 | 96.2 | 96.2 | 96.2 | 95.2 | 95.1 | 95.2 |
| | 0.50 | 0.700 | 0.700 | 0.2 | 96.0 | 96.0 | 96.0 | 95.0 | 95.0 | 95.0 |
| | | | | 0.4 | 96.2 | 96.1 | 96.1 | 95.1 | 95.1 | 95.1 |
| | | | | 0.6 | 96.4 | 96.3 | 96.3 | 95.4 | 95.3 | 95.3 |
| | 0.75 | 0.700 | 0.700 | 0.2 | 96.2 | 96.2 | 96.2 | 95.2 | 95.2 | 95.2 |
| | | | | 0.4 | 96.3 | 96.3 | 96.2 | 95.3 | 95.3 | 95.2 |
| | | | | 0.6 | 96.3 | 96.2 | 96.1 | 95.3 | 95.2 | 95.1 |

Table S81: Coverage probability (%) when the null hypothesis is true, $\xi_g = \pi_g = 0.7$, and $G = 3$.

| | | | | | Exact | | | Mid-$p$ | | |
|---|---|---|---|---|---|---|---|---|---|---|
| $N$ | $\tau$ | $E[\xi_s]$ | $E[\pi_s]$ | $\rho$ | MLE | CMAE | UMVCUE | MLE | CMAE | UMVCUE |
| 50 | 0.25 | 0.700 | 0.700 | 0.2 | 97.0 | 97.0 | 97.0 | 95.3 | 95.3 | 95.3 |
| | | | | 0.4 | 97.0 | 97.0 | 97.0 | 95.4 | 95.4 | 95.4 |
| | | | | 0.6 | 97.1 | 97.0 | 97.1 | 95.6 | 95.4 | 95.5 |
| | 0.50 | 0.700 | 0.700 | 0.2 | 97.1 | 97.0 | 97.0 | 95.5 | 95.4 | 95.4 |
| | | | | 0.4 | 97.0 | 96.9 | 96.9 | 95.4 | 95.3 | 95.3 |
| | | | | 0.6 | 97.2 | 97.1 | 97.1 | 95.7 | 95.4 | 95.5 |
| | 0.75 | 0.700 | 0.700 | 0.2 | 97.0 | 96.9 | 96.9 | 95.4 | 95.4 | 95.3 |
| | | | | 0.4 | 97.1 | 97.0 | 96.9 | 95.6 | 95.5 | 95.3 |
| | | | | 0.6 | 97.3 | 97.2 | 97.0 | 95.8 | 95.6 | 95.4 |
| 200 | 0.25 | 0.700 | 0.700 | 0.2 | 96.1 | 96.1 | 96.1 | 95.1 | 95.1 | 95.1 |
| | | | | 0.4 | 96.2 | 96.1 | 96.1 | 95.2 | 95.1 | 95.2 |
| | | | | 0.6 | 96.3 | 96.2 | 96.3 | 95.3 | 95.2 | 95.3 |
| | 0.50 | 0.700 | 0.700 | 0.2 | 96.1 | 96.1 | 96.1 | 95.1 | 95.0 | 95.0 |
| | | | | 0.4 | 96.1 | 96.1 | 96.0 | 95.1 | 95.0 | 95.0 |
| | | | | 0.6 | 96.4 | 96.2 | 96.2 | 95.3 | 95.1 | 95.2 |
| | 0.75 | 0.700 | 0.700 | 0.2 | 96.2 | 96.2 | 96.2 | 95.1 | 95.1 | 95.1 |
| | | | | 0.4 | 96.4 | 96.3 | 96.1 | 95.4 | 95.3 | 95.1 |
| | | | | 0.6 | 96.5 | 96.3 | 96.1 | 95.5 | 95.4 | 95.1 |

Table S82: Coverage probability (%) when the null hypothesis is true, $\xi_g = \pi_g = 0.7$, and $G = 4$.

| | | | | | Exact | | | Mid-$p$ | | |
|---|---|---|---|---|---|---|---|---|---|---|
| $N$ | $\tau$ | E[$\xi_s$] | E[$\pi_s$] | $\rho$ | MLE | CMAE | UMVCUE | MLE | CMAE | UMVCUE |
| 50 | 0.25 | 0.700 | 0.700 | 0.2 | 96.9 | 96.8 | 96.9 | 95.3 | 95.2 | 95.3 |
| | | | | 0.4 | 97.0 | 97.0 | 97.0 | 95.4 | 95.3 | 95.4 |
| | | | | 0.6 | 97.1 | 97.0 | 97.1 | 95.5 | 95.4 | 95.4 |
| | 0.50 | 0.700 | 0.700 | 0.2 | 96.8 | 96.8 | 96.8 | 95.3 | 95.2 | 95.2 |
| | | | | 0.4 | 97.1 | 97.0 | 97.0 | 95.5 | 95.4 | 95.3 |
| | | | | 0.6 | 97.2 | 97.1 | 97.1 | 95.7 | 95.4 | 95.4 |
| | 0.75 | 0.700 | 0.700 | 0.2 | 97.1 | 97.0 | 96.9 | 95.4 | 95.3 | 95.2 |
| | | | | 0.4 | 97.1 | 97.0 | 96.8 | 95.5 | 95.4 | 95.1 |
| | | | | 0.6 | 97.5 | 97.3 | 97.0 | 96.0 | 95.7 | 95.3 |
| 200 | 0.25 | 0.700 | 0.700 | 0.2 | 96.1 | 96.1 | 96.1 | 95.1 | 95.1 | 95.1 |
| | | | | 0.4 | 96.2 | 96.1 | 96.2 | 95.1 | 95.1 | 95.1 |
| | | | | 0.6 | 96.3 | 96.2 | 96.2 | 95.2 | 95.1 | 95.2 |
| | 0.50 | 0.700 | 0.700 | 0.2 | 96.1 | 96.1 | 96.1 | 95.0 | 95.0 | 95.0 |
| | | | | 0.4 | 96.3 | 96.2 | 96.2 | 95.3 | 95.2 | 95.2 |
| | | | | 0.6 | 96.3 | 96.1 | 96.1 | 95.3 | 95.1 | 95.1 |
| | 0.75 | 0.700 | 0.700 | 0.2 | 96.2 | 96.1 | 96.0 | 95.1 | 95.1 | 95.0 |
| | | | | 0.4 | 96.2 | 96.1 | 95.9 | 95.2 | 95.1 | 94.8 |
| | | | | 0.6 | 96.6 | 96.4 | 96.1 | 95.6 | 95.4 | 95.0 |

Table S83: Coverage probability (%) when the null hypothesis is true, $\xi_g$ =0.3, $\pi_g = 0.5$, and $G = 2$.

| | | | | | Exact | | | Mid-$p$ | | |
|---|---|---|---|---|---|---|---|---|---|---|
| $N$ | $\tau$ | E[$\xi_s$] | E[$\pi_s$] | $\rho$ | MLE | CMAE | UMVCUE | MLE | CMAE | UMVCUE |
| 50 | 0.25 | 0.300 | 0.500 | 0.2 | 96.6 | 96.7 | 96.6 | 95.0 | 95.0 | 95.0 |
| | | | | 0.4 | 96.7 | 96.7 | 96.7 | 95.2 | 95.2 | 95.2 |
| | | | | 0.5 | 96.9 | 96.9 | 96.9 | 95.5 | 95.4 | 95.4 |
| | 0.50 | 0.300 | 0.500 | 0.2 | 96.7 | 96.7 | 96.7 | 95.2 | 95.2 | 95.2 |
| | | | | 0.4 | 96.9 | 96.9 | 96.9 | 95.4 | 95.3 | 95.3 |
| | | | | 0.5 | 96.9 | 96.8 | 96.9 | 95.5 | 95.4 | 95.5 |
| | 0.75 | 0.300 | 0.500 | 0.2 | 96.7 | 96.8 | 96.7 | 95.2 | 95.2 | 95.2 |
| | | | | 0.4 | 97.0 | 96.9 | 96.9 | 95.5 | 95.5 | 95.4 |
| | | | | 0.5 | 97.1 | 97.0 | 96.9 | 95.6 | 95.5 | 95.5 |
| 200 | 0.25 | 0.300 | 0.500 | 0.2 | 96.1 | 96.1 | 96.1 | 95.1 | 95.1 | 95.1 |
| | | | | 0.4 | 96.1 | 96.1 | 96.1 | 95.2 | 95.2 | 95.2 |
| | | | | 0.5 | 96.2 | 96.2 | 96.2 | 95.2 | 95.2 | 95.2 |
| | 0.50 | 0.300 | 0.500 | 0.2 | 96.1 | 96.1 | 96.0 | 95.1 | 95.1 | 95.1 |
| | | | | 0.4 | 96.2 | 96.1 | 96.1 | 95.3 | 95.2 | 95.2 |
| | | | | 0.5 | 96.2 | 96.1 | 96.1 | 95.2 | 95.1 | 95.1 |
| | 0.75 | 0.300 | 0.500 | 0.2 | 96.1 | 96.1 | 96.1 | 95.2 | 95.2 | 95.2 |
| | | | | 0.4 | 96.1 | 96.1 | 96.0 | 95.2 | 95.2 | 95.1 |
| | | | | 0.5 | 96.4 | 96.3 | 96.2 | 95.5 | 95.3 | 95.3 |

Table S84: Coverage probability (%) when the null hypothesis is true, $\xi_g$ =0.3, $\pi_g = 0.5$, and $G = 3$.

| | | | | | Exact | | | Mid-$p$ | | |
|---|---|---|---|---|---|---|---|---|---|---|
| $N$ | $\tau$ | E[$\xi_s$] | E[$\pi_s$] | $\rho$ | MLE | CMAE | UMVCUE | MLE | CMAE | UMVCUE |
| 50 | 0.25 | 0.300 | 0.500 | 0.2 | 96.8 | 96.9 | 96.8 | 95.3 | 95.3 | 95.3 |
| | | | | 0.4 | 97.0 | 96.9 | 97.0 | 95.5 | 95.4 | 95.5 |
| | | | | 0.5 | 97.1 | 96.9 | 97.0 | 95.6 | 95.4 | 95.5 |
| | 0.50 | 0.300 | 0.500 | 0.2 | 97.0 | 97.0 | 97.0 | 95.4 | 95.4 | 95.4 |
| | | | | 0.4 | 97.1 | 96.9 | 97.0 | 95.6 | 95.4 | 95.5 |
| | | | | 0.5 | 97.1 | 97.0 | 97.0 | 95.6 | 95.5 | 95.5 |
| | 0.75 | 0.300 | 0.500 | 0.2 | 96.9 | 96.9 | 96.8 | 95.3 | 95.3 | 95.3 |
| | | | | 0.4 | 97.1 | 97.0 | 96.9 | 95.6 | 95.5 | 95.4 |
| | | | | 0.5 | 97.2 | 97.1 | 97.0 | 95.6 | 95.5 | 95.4 |
| 200 | 0.25 | 0.300 | 0.500 | 0.2 | 96.0 | 96.0 | 96.0 | 95.2 | 95.1 | 95.1 |
| | | | | 0.4 | 96.1 | 96.1 | 96.1 | 95.2 | 95.1 | 95.2 |
| | | | | 0.5 | 96.2 | 96.2 | 96.2 | 95.3 | 95.2 | 95.3 |
| | 0.50 | 0.300 | 0.500 | 0.2 | 96.0 | 96.0 | 96.0 | 95.1 | 95.1 | 95.1 |
| | | | | 0.4 | 96.2 | 96.1 | 96.1 | 95.3 | 95.2 | 95.2 |
| | | | | 0.5 | 96.2 | 96.2 | 96.2 | 95.3 | 95.2 | 95.2 |
| | 0.75 | 0.300 | 0.500 | 0.2 | 96.1 | 96.1 | 96.0 | 95.2 | 95.1 | 95.1 |
| | | | | 0.4 | 96.3 | 96.2 | 96.0 | 95.4 | 95.2 | 95.1 |
| | | | | 0.5 | 96.4 | 96.3 | 96.2 | 95.5 | 95.4 | 95.2 |

Table S85: Coverage probability (%) when the null hypothesis is true, $\xi_g$ =0.3, $\pi_g = 0.5$, and $G = 4$.

| | | | | | Exact | | | Mid-$p$ | | |
|---|---|---|---|---|---|---|---|---|---|---|
| $N$ | $\tau$ | E[$\xi_s$] | E[$\pi_s$] | $\rho$ | MLE | CMAE | UMVCUE | MLE | CMAE | UMVCUE |
| 50 | 0.25 | 0.300 | 0.500 | 0.2 | 96.9 | 96.9 | 96.9 | 95.3 | 95.3 | 95.3 |
| | | | | 0.4 | 96.9 | 96.8 | 96.9 | 95.4 | 95.2 | 95.3 |
| | | | | 0.5 | 97.0 | 96.9 | 96.9 | 95.5 | 95.3 | 95.4 |
| | 0.50 | 0.300 | 0.500 | 0.2 | 96.8 | 96.8 | 96.8 | 95.4 | 95.3 | 95.3 |
| | | | | 0.4 | 97.0 | 96.9 | 96.9 | 95.5 | 95.4 | 95.4 |
| | | | | 0.5 | 97.2 | 97.0 | 97.0 | 95.6 | 95.5 | 95.5 |
| | 0.75 | 0.300 | 0.500 | 0.2 | 96.8 | 96.7 | 96.7 | 95.3 | 95.2 | 95.1 |
| | | | | 0.4 | 97.0 | 96.9 | 96.8 | 95.6 | 95.4 | 95.2 |
| | | | | 0.5 | 97.1 | 97.0 | 96.9 | 95.7 | 95.5 | 95.3 |
| 200 | 0.25 | 0.300 | 0.500 | 0.2 | 96.0 | 96.0 | 96.0 | 95.2 | 95.1 | 95.1 |
| | | | | 0.4 | 96.0 | 95.9 | 96.0 | 95.1 | 95.0 | 95.0 |
| | | | | 0.5 | 96.1 | 96.0 | 96.1 | 95.2 | 95.0 | 95.1 |
| | 0.50 | 0.300 | 0.500 | 0.2 | 96.1 | 96.0 | 96.0 | 95.1 | 95.0 | 95.0 |
| | | | | 0.4 | 96.2 | 96.0 | 96.0 | 95.2 | 95.1 | 95.1 |
| | | | | 0.5 | 96.3 | 96.1 | 96.1 | 95.4 | 95.2 | 95.2 |
| | 0.75 | 0.300 | 0.500 | 0.2 | 96.0 | 96.0 | 95.9 | 95.1 | 95.0 | 95.0 |
| | | | | 0.4 | 96.2 | 96.1 | 96.0 | 95.3 | 95.1 | 95.0 |
| | | | | 0.5 | 96.4 | 96.2 | 96.0 | 95.5 | 95.3 | 95.0 |

Table S86: Coverage probability (%) when the null hypothesis is true, $\xi_g$ =0.7, $\pi_g = 0.5$, and $G = 2$.

| | | | | | Exact | | | Mid-$p$ | | |
|---|---|---|---|---|---|---|---|---|---|---|
| $N$ | $\tau$ | E[$\xi_s$] | E[$\pi_s$] | $\rho$ | MLE | CMAE | UMVCUE | MLE | CMAE | UMVCUE |
| 50 | 0.25 | 0.700 | 0.500 | 0.2 | 96.7 | 96.8 | 96.7 | 95.2 | 95.3 | 95.2 |
| | | | | 0.4 | 96.8 | 96.8 | 96.8 | 95.4 | 95.3 | 95.3 |
| | | | | 0.5 | 96.8 | 96.8 | 96.8 | 95.4 | 95.3 | 95.4 |
| | 0.50 | 0.700 | 0.500 | 0.2 | 96.8 | 96.8 | 96.8 | 95.3 | 95.4 | 95.3 |
| | | | | 0.4 | 97.0 | 96.9 | 96.9 | 95.5 | 95.4 | 95.5 |
| | | | | 0.5 | 97.0 | 96.9 | 96.9 | 95.6 | 95.4 | 95.5 |
| | 0.75 | 0.700 | 0.500 | 0.2 | 96.7 | 96.8 | 96.7 | 95.2 | 95.2 | 95.1 |
| | | | | 0.4 | 96.9 | 96.8 | 96.7 | 95.4 | 95.4 | 95.2 |
| | | | | 0.5 | 96.9 | 96.8 | 96.7 | 95.5 | 95.4 | 95.2 |
| 200 | 0.25 | 0.700 | 0.500 | 0.2 | 96.1 | 96.1 | 96.1 | 95.1 | 95.2 | 95.1 |
| | | | | 0.4 | 96.1 | 96.1 | 96.1 | 95.2 | 95.1 | 95.2 |
| | | | | 0.5 | 96.1 | 96.0 | 96.0 | 95.1 | 95.0 | 95.1 |
| | 0.50 | 0.700 | 0.500 | 0.2 | 96.1 | 96.1 | 96.1 | 95.2 | 95.2 | 95.2 |
| | | | | 0.4 | 96.1 | 96.0 | 96.0 | 95.1 | 95.0 | 95.0 |
| | | | | 0.5 | 96.3 | 96.2 | 96.2 | 95.4 | 95.3 | 95.3 |
| | 0.75 | 0.700 | 0.500 | 0.2 | 96.0 | 96.0 | 95.9 | 95.1 | 95.0 | 95.0 |
| | | | | 0.4 | 96.1 | 96.0 | 96.0 | 95.1 | 95.1 | 95.0 |
| | | | | 0.5 | 96.2 | 96.1 | 96.1 | 95.3 | 95.3 | 95.1 |

Table S87: Coverage probability (%) when the null hypothesis is true, $\xi_g$ =0.7, $\pi_g = 0.5$, and $G = 3$.

| | | | | | Exact | | | Mid-$p$ | | |
|---|---|---|---|---|---|---|---|---|---|---|
| $N$ | $\tau$ | E[$\xi_s$] | E[$\pi_s$] | $\rho$ | MLE | CMAE | UMVCUE | MLE | CMAE | UMVCUE |
| 50 | 0.25 | 0.700 | 0.500 | 0.2 | 96.8 | 96.9 | 96.8 | 95.2 | 95.3 | 95.3 |
| | | | | 0.4 | 96.9 | 96.8 | 96.9 | 95.5 | 95.4 | 95.4 |
| | | | | 0.5 | 97.0 | 96.9 | 97.0 | 95.6 | 95.4 | 95.5 |
| | 0.50 | 0.700 | 0.500 | 0.2 | 96.8 | 96.8 | 96.8 | 95.3 | 95.3 | 95.3 |
| | | | | 0.4 | 97.0 | 96.9 | 96.9 | 95.5 | 95.4 | 95.4 |
| | | | | 0.5 | 97.0 | 96.8 | 96.8 | 95.5 | 95.3 | 95.3 |
| | 0.75 | 0.700 | 0.500 | 0.2 | 96.8 | 96.8 | 96.7 | 95.4 | 95.3 | 95.2 |
| | | | | 0.4 | 97.1 | 97.0 | 96.7 | 95.6 | 95.5 | 95.2 |
| | | | | 0.5 | 97.2 | 97.1 | 96.8 | 95.8 | 95.7 | 95.3 |
| 200 | 0.25 | 0.700 | 0.500 | 0.2 | 96.1 | 96.0 | 96.0 | 95.1 | 95.1 | 95.1 |
| | | | | 0.4 | 96.1 | 96.1 | 96.1 | 95.2 | 95.1 | 95.1 |
| | | | | 0.5 | 96.2 | 96.1 | 96.2 | 95.2 | 95.2 | 95.2 |
| | 0.50 | 0.700 | 0.500 | 0.2 | 96.1 | 96.1 | 96.1 | 95.2 | 95.1 | 95.1 |
| | | | | 0.4 | 96.2 | 96.1 | 96.1 | 95.2 | 95.1 | 95.1 |
| | | | | 0.5 | 96.2 | 96.1 | 96.1 | 95.3 | 95.2 | 95.1 |
| | 0.75 | 0.700 | 0.500 | 0.2 | 96.1 | 96.1 | 96.0 | 95.2 | 95.1 | 95.0 |
| | | | | 0.4 | 96.2 | 96.2 | 96.0 | 95.3 | 95.2 | 95.0 |
| | | | | 0.5 | 96.4 | 96.3 | 96.0 | 95.4 | 95.3 | 95.0 |

Table S88: Coverage probability (%) when the null hypothesis is true, $\xi_g$ =0.7, $\pi_g = 0.5$, and $G = 4$.

| | | | | | Exact | | | Mid-$p$ | | |
|---|---|---|---|---|---|---|---|---|---|---|
| $N$ | $\tau$ | E[$\xi_s$] | E[$\pi_s$] | $\rho$ | MLE | CMAE | UMVCUE | MLE | CMAE | UMVCUE |
| 50 | 0.25 | 0.700 | 0.500 | 0.2 | 96.8 | 96.7 | 96.7 | 95.2 | 95.2 | 95.2 |
| | | | | 0.4 | 97.0 | 96.9 | 97.0 | 95.5 | 95.4 | 95.5 |
| | | | | 0.5 | 97.0 | 96.9 | 96.9 | 95.4 | 95.3 | 95.3 |
| | 0.50 | 0.700 | 0.500 | 0.2 | 96.8 | 96.8 | 96.8 | 95.3 | 95.2 | 95.2 |
| | | | | 0.4 | 96.9 | 96.7 | 96.7 | 95.3 | 95.2 | 95.1 |
| | | | | 0.5 | 96.9 | 96.8 | 96.7 | 95.4 | 95.2 | 95.2 |
| | 0.75 | 0.700 | 0.500 | 0.2 | 96.8 | 96.8 | 96.6 | 95.3 | 95.2 | 95.1 |
| | | | | 0.4 | 97.0 | 96.9 | 96.5 | 95.5 | 95.3 | 94.9 |
| | | | | 0.5 | 97.2 | 97.1 | 96.6 | 95.7 | 95.5 | 94.9 |
| 200 | 0.25 | 0.700 | 0.500 | 0.2 | 96.0 | 96.0 | 96.0 | 95.1 | 95.0 | 95.0 |
| | | | | 0.4 | 96.1 | 96.1 | 96.1 | 95.2 | 95.1 | 95.1 |
| | | | | 0.5 | 96.0 | 95.9 | 96.0 | 95.0 | 94.9 | 94.9 |
| | 0.50 | 0.700 | 0.500 | 0.2 | 96.1 | 96.1 | 96.0 | 95.2 | 95.1 | 95.1 |
| | | | | 0.4 | 96.2 | 96.1 | 96.0 | 95.2 | 95.1 | 95.1 |
| | | | | 0.5 | 96.1 | 96.0 | 96.0 | 95.2 | 95.1 | 95.0 |
| | 0.75 | 0.700 | 0.500 | 0.2 | 96.0 | 96.0 | 95.9 | 95.1 | 95.0 | 94.9 |
| | | | | 0.4 | 96.2 | 96.2 | 95.9 | 95.3 | 95.3 | 94.9 |
| | | | | 0.5 | 96.4 | 96.4 | 95.9 | 95.6 | 95.4 | 94.9 |

Table S89: Coverage probability (%) when the alternative hypothesis is true, and only one treatment is effective, and $G = 2$.

| | | | | | Exact | | | Mid-$p$ | | |
|---|---|---|---|---|---|---|---|---|---|---|
| $N$ | $\tau$ | E[$\xi_s$] | E[$\pi_s$] | $\rho$ | MLE | CMAE | UMVCUE | MLE | CMAE | UMVCUE |
| 50 | 0.25 | 0.755 | 0.755 | 0.2 | 97.0 | 97.0 | 97.0 | 95.2 | 95.2 | 95.2 |
| | | | | 0.4 | 96.7 | 96.7 | 96.7 | 95.2 | 95.2 | 95.2 |
| | | | | 0.6 | 97.1 | 97.0 | 97.0 | 95.5 | 95.4 | 95.5 |
| | 0.50 | 0.773 | 0.773 | 0.2 | 97.1 | 97.0 | 97.0 | 95.4 | 95.3 | 95.3 |
| | | | | 0.4 | 97.2 | 97.2 | 97.2 | 95.5 | 95.5 | 95.5 |
| | | | | 0.6 | 97.0 | 97.0 | 97.0 | 95.5 | 95.5 | 95.5 |
| | 0.75 | 0.778 | 0.778 | 0.2 | 96.9 | 97.0 | 96.9 | 95.2 | 95.2 | 95.2 |
| | | | | 0.4 | 97.1 | 97.0 | 97.1 | 95.5 | 95.4 | 95.5 |
| | | | | 0.6 | 96.9 | 96.8 | 96.8 | 95.2 | 95.1 | 95.2 |
| 200 | 0.25 | 0.633 | 0.633 | 0.2 | 96.2 | 96.2 | 96.2 | 95.3 | 95.3 | 95.3 |
| | | | | 0.4 | 96.2 | 96.2 | 96.2 | 95.2 | 95.2 | 95.2 |
| | | | | 0.6 | 96.1 | 96.0 | 96.1 | 95.0 | 94.9 | 95.0 |
| | 0.50 | 0.642 | 0.642 | 0.2 | 96.2 | 96.1 | 96.2 | 95.1 | 95.1 | 95.1 |
| | | | | 0.4 | 95.9 | 95.8 | 95.9 | 94.8 | 94.8 | 94.8 |
| | | | | 0.6 | 96.0 | 96.0 | 96.0 | 95.0 | 94.9 | 95.0 |
| | 0.75 | 0.644 | 0.644 | 0.2 | 96.2 | 96.2 | 96.2 | 95.3 | 95.3 | 95.3 |
| | | | | 0.4 | 96.0 | 96.0 | 96.0 | 95.0 | 95.0 | 95.0 |
| | | | | 0.6 | 95.9 | 95.9 | 95.9 | 95.0 | 95.0 | 95.0 |

Table S90: Coverage probability (%) when the alternative hypothesis is true, and only one treatment is effective, and $G = 3$.

| | | | | | Exact | | | Mid-$p$ | | |
|---|---|---|---|---|---|---|---|---|---|---|
| $N$ | $\tau$ | $E[\xi_s]$ | $E[\pi_s]$ | $\rho$ | MLE | CMAE | UMVCUE | MLE | CMAE | UMVCUE |
| 50 | 0.25 | 0.736 | 0.736 | 0.2 | 97.2 | 97.1 | 97.1 | 95.6 | 95.4 | 95.6 |
| | | | | 0.4 | 97.0 | 96.9 | 96.9 | 95.3 | 95.3 | 95.3 |
| | | | | 0.6 | 97.1 | 97.0 | 97.0 | 95.3 | 95.3 | 95.3 |
| | 0.50 | 0.768 | 0.768 | 0.2 | 97.0 | 96.9 | 97.0 | 95.3 | 95.3 | 95.3 |
| | | | | 0.4 | 96.9 | 96.9 | 96.9 | 95.2 | 95.2 | 95.2 |
| | | | | 0.6 | 96.9 | 96.8 | 96.9 | 95.3 | 95.2 | 95.2 |
| | 0.75 | 0.777 | 0.777 | 0.2 | 97.2 | 97.2 | 97.1 | 95.6 | 95.6 | 95.6 |
| | | | | 0.4 | 96.8 | 96.7 | 96.8 | 95.1 | 95.1 | 95.1 |
| | | | | 0.6 | 97.0 | 97.0 | 97.0 | 95.3 | 95.3 | 95.3 |
| 200 | 0.25 | 0.624 | 0.624 | 0.2 | 96.1 | 96.2 | 96.1 | 95.3 | 95.3 | 95.3 |
| | | | | 0.4 | 96.2 | 96.3 | 96.3 | 95.3 | 95.2 | 95.2 |
| | | | | 0.6 | 96.4 | 96.5 | 96.4 | 95.6 | 95.6 | 95.5 |
| | 0.50 | 0.639 | 0.639 | 0.2 | 96.0 | 96.0 | 96.0 | 94.9 | 94.9 | 94.9 |
| | | | | 0.4 | 96.1 | 96.1 | 96.1 | 95.3 | 95.2 | 95.2 |
| | | | | 0.6 | 95.8 | 95.7 | 95.8 | 95.0 | 94.9 | 94.9 |
| | 0.75 | 0.643 | 0.643 | 0.2 | 96.2 | 96.1 | 96.1 | 95.4 | 95.3 | 95.4 |
| | | | | 0.4 | 96.0 | 96.0 | 96.0 | 94.9 | 94.9 | 94.9 |
| | | | | 0.6 | 96.2 | 96.1 | 96.2 | 95.2 | 95.1 | 95.1 |

Table S91: Coverage probability (%) when the alternative hypothesis is true, and only one treatment is effective, and $G = 4$.

| | | | | | Exact | | | Mid-$p$ | | |
|---|---|---|---|---|---|---|---|---|---|---|
| $N$ | $\tau$ | $E[\xi_s]$ | $E[\pi_s]$ | $\rho$ | MLE | CMAE | UMVCUE | MLE | CMAE | UMVCUE |
| 50 | 0.25 | 0.723 | 0.723 | 0.2 | 96.9 | 96.8 | 96.8 | 95.1 | 95.1 | 95.1 |
| | | | | 0.4 | 96.9 | 96.8 | 96.9 | 95.4 | 95.3 | 95.4 |
| | | | | 0.6 | 97.3 | 97.1 | 97.2 | 95.6 | 95.5 | 95.5 |
| | 0.50 | 0.763 | 0.763 | 0.2 | 97.0 | 97.0 | 97.1 | 95.4 | 95.4 | 95.4 |
| | | | | 0.4 | 96.4 | 96.4 | 96.4 | 95.0 | 94.9 | 95.0 |
| | | | | 0.6 | 97.0 | 96.9 | 97.0 | 95.3 | 95.3 | 95.4 |
| | 0.75 | 0.775 | 0.775 | 0.2 | 96.8 | 96.7 | 96.7 | 95.3 | 95.3 | 95.3 |
| | | | | 0.4 | 97.2 | 97.2 | 97.2 | 95.4 | 95.3 | 95.3 |
| | | | | 0.6 | 97.0 | 97.0 | 97.0 | 95.3 | 95.2 | 95.3 |
| 200 | 0.25 | 0.618 | 0.618 | 0.2 | 96.1 | 96.2 | 96.1 | 95.3 | 95.3 | 95.3 |
| | | | | 0.4 | 95.7 | 95.7 | 95.7 | 94.7 | 94.7 | 94.7 |
| | | | | 0.6 | 95.9 | 95.8 | 95.9 | 94.8 | 94.8 | 94.9 |
| | 0.50 | 0.637 | 0.637 | 0.2 | 96.0 | 96.0 | 96.0 | 94.8 | 94.8 | 94.8 |
| | | | | 0.4 | 96.4 | 96.4 | 96.4 | 95.4 | 95.4 | 95.4 |
| | | | | 0.6 | 96.1 | 96.1 | 96.1 | 95.0 | 95.0 | 95.0 |
| | 0.75 | 0.643 | 0.643 | 0.2 | 96.2 | 96.2 | 96.2 | 95.2 | 95.2 | 95.2 |
| | | | | 0.4 | 96.3 | 96.3 | 96.3 | 95.4 | 95.3 | 95.3 |
| | | | | 0.6 | 95.8 | 95.7 | 95.8 | 94.8 | 94.6 | 94.7 |

Table S92: Coverage probability (%) when the alternative hypothesis is true, and relationship between the treatment group and binomial probability is linear, and $G = 2$.

| | | | | | Exact | | | Mid-$p$ | | |
|---|---|---|---|---|---|---|---|---|---|---|
| $N$ | $\tau$ | $\mathrm{E}[\xi_s]$ | $\mathrm{E}[\pi_s]$ | $\rho$ | MLE | CMAE | UMVCUE | MLE | CMAE | UMVCUE |
| 50 | 0.25 | 0.741 | 0.741 | 0.2 | 96.5 | 96.4 | 96.5 | 95.0 | 95.0 | 95.0 |
| | | | | 0.4 | 96.7 | 96.6 | 96.7 | 95.0 | 94.9 | 94.9 |
| | | | | 0.6 | 96.9 | 96.7 | 96.8 | 95.1 | 95.0 | 95.1 |
| | 0.50 | 0.756 | 0.756 | 0.2 | 97.0 | 97.0 | 97.0 | 95.2 | 95.2 | 95.2 |
| | | | | 0.4 | 96.8 | 96.8 | 96.8 | 95.3 | 95.1 | 95.2 |
| | | | | 0.6 | 97.0 | 96.8 | 96.9 | 95.2 | 95.0 | 95.1 |
| | 0.75 | 0.765 | 0.765 | 0.2 | 96.9 | 96.9 | 96.9 | 95.3 | 95.3 | 95.3 |
| | | | | 0.4 | 97.0 | 97.0 | 96.9 | 95.4 | 95.3 | 95.3 |
| | | | | 0.6 | 97.0 | 96.9 | 96.9 | 95.4 | 95.2 | 95.3 |
| 200 | 0.25 | 0.626 | 0.626 | 0.2 | 96.0 | 96.0 | 96.0 | 95.0 | 95.0 | 95.0 |
| | | | | 0.4 | 96.1 | 96.1 | 96.1 | 95.2 | 95.1 | 95.1 |
| | | | | 0.6 | 96.2 | 96.2 | 96.2 | 95.2 | 95.2 | 95.2 |
| | 0.50 | 0.633 | 0.633 | 0.2 | 96.3 | 96.3 | 96.3 | 95.4 | 95.4 | 95.4 |
| | | | | 0.4 | 96.2 | 96.2 | 96.2 | 95.1 | 95.0 | 95.0 |
| | | | | 0.6 | 95.9 | 95.9 | 95.9 | 94.9 | 94.9 | 94.9 |
| | 0.75 | 0.637 | 0.637 | 0.2 | 96.3 | 96.3 | 96.3 | 95.4 | 95.4 | 95.4 |
| | | | | 0.4 | 96.0 | 96.0 | 96.0 | 95.2 | 95.2 | 95.2 |
| | | | | 0.6 | 96.4 | 96.2 | 96.3 | 95.4 | 95.2 | 95.3 |

Table S93: Coverage probability (%) when the alternative hypothesis is true, and relationship between the treatment group and binomial probability is linear, and $G = 3$.

| | | | | | Exact | | | Mid-$p$ | | |
|---|---|---|---|---|---|---|---|---|---|---|
| $N$ | $\tau$ | $\mathrm{E}[\xi_s]$ | $\mathrm{E}[\pi_s]$ | $\rho$ | MLE | CMAE | UMVCUE | MLE | CMAE | UMVCUE |
| 50 | 0.25 | 0.725 | 0.725 | 0.2 | 97.0 | 97.0 | 97.1 | 95.6 | 95.6 | 95.6 |
| | | | | 0.4 | 96.7 | 96.7 | 96.8 | 95.2 | 95.2 | 95.2 |
| | | | | 0.6 | 97.4 | 97.3 | 97.4 | 95.6 | 95.4 | 95.5 |
| | 0.50 | 0.744 | 0.744 | 0.2 | 96.8 | 96.8 | 96.8 | 95.1 | 95.0 | 95.0 |
| | | | | 0.4 | 97.2 | 97.1 | 97.0 | 95.3 | 95.1 | 95.2 |
| | | | | 0.6 | 97.1 | 97.0 | 97.0 | 95.6 | 95.4 | 95.5 |
| | 0.75 | 0.755 | 0.755 | 0.2 | 97.1 | 97.0 | 97.0 | 95.4 | 95.4 | 95.3 |
| | | | | 0.4 | 97.2 | 97.2 | 97.2 | 95.8 | 95.7 | 95.7 |
| | | | | 0.6 | 97.3 | 97.0 | 96.9 | 95.5 | 95.1 | 95.1 |
| 200 | 0.25 | 0.619 | 0.619 | 0.2 | 96.0 | 95.9 | 95.9 | 94.9 | 94.9 | 94.9 |
| | | | | 0.4 | 96.1 | 96.1 | 96.1 | 95.0 | 95.0 | 95.1 |
| | | | | 0.6 | 96.1 | 96.0 | 96.0 | 95.1 | 95.0 | 95.1 |
| | 0.50 | 0.628 | 0.628 | 0.2 | 95.7 | 95.8 | 95.7 | 94.8 | 94.8 | 94.8 |
| | | | | 0.4 | 96.1 | 96.0 | 96.1 | 95.1 | 95.1 | 95.1 |
| | | | | 0.6 | 96.3 | 96.2 | 96.2 | 95.3 | 95.2 | 95.2 |
| | 0.75 | 0.632 | 0.632 | 0.2 | 96.2 | 96.2 | 96.2 | 95.3 | 95.3 | 95.3 |
| | | | | 0.4 | 96.2 | 96.2 | 96.1 | 95.5 | 95.4 | 95.3 |
| | | | | 0.6 | 96.3 | 96.1 | 96.2 | 95.3 | 95.2 | 95.1 |

Table S94: Coverage probability (%) when the alternative hypothesis is true, and relationship between the treatment group and binomial probability is linear, and $G = 4$.

| | | | | | Exact | | | Mid-$p$ | | |
|---|---|---|---|---|---|---|---|---|---|---|
| $N$ | $\tau$ | $E[\xi_s]$ | $E[\pi_s]$ | $\rho$ | MLE | CMAE | UMVCUE | MLE | CMAE | UMVCUE |
| 50 | 0.25 | 0.717 | 0.717 | 0.2 | 96.9 | 96.8 | 96.8 | 95.2 | 95.3 | 95.2 |
| | | | | 0.4 | 97.1 | 97.0 | 97.1 | 95.5 | 95.5 | 95.5 |
| | | | | 0.6 | 97.0 | 96.9 | 96.9 | 95.5 | 95.3 | 95.4 |
| | 0.50 | 0.738 | 0.738 | 0.2 | 96.8 | 96.8 | 96.8 | 95.3 | 95.1 | 95.1 |
| | | | | 0.4 | 97.5 | 97.2 | 97.3 | 95.8 | 95.6 | 95.6 |
| | | | | 0.6 | 97.1 | 96.8 | 96.9 | 95.5 | 95.3 | 95.3 |
| | 0.75 | 0.750 | 0.750 | 0.2 | 97.1 | 97.0 | 97.0 | 95.6 | 95.5 | 95.4 |
| | | | | 0.4 | 97.1 | 97.0 | 96.9 | 95.8 | 95.7 | 95.4 |
| | | | | 0.6 | 97.2 | 97.0 | 96.9 | 95.6 | 95.3 | 95.2 |
| 200 | 0.25 | 0.616 | 0.616 | 0.2 | 96.1 | 96.1 | 96.0 | 95.1 | 95.1 | 95.1 |
| | | | | 0.4 | 96.3 | 96.2 | 96.2 | 95.3 | 95.2 | 95.3 |
| | | | | 0.6 | 96.0 | 95.9 | 96.0 | 95.2 | 95.0 | 95.2 |
| | 0.50 | 0.625 | 0.625 | 0.2 | 96.2 | 96.1 | 96.1 | 95.0 | 95.0 | 95.0 |
| | | | | 0.4 | 96.2 | 96.2 | 96.2 | 95.2 | 95.1 | 95.1 |
| | | | | 0.6 | 96.2 | 96.1 | 96.1 | 95.0 | 94.9 | 95.0 |
| | 0.75 | 0.630 | 0.630 | 0.2 | 96.2 | 96.0 | 96.1 | 95.1 | 95.1 | 95.0 |
| | | | | 0.4 | 96.1 | 96.0 | 96.0 | 95.1 | 95.0 | 95.0 |
| | | | | 0.6 | 96.4 | 96.2 | 96.0 | 95.4 | 95.2 | 94.9 |

Table S95: Coverage probability (%) when the alternative hypothesis is true, and all treatments are effective, and $G = 2$.

| | | | | | Exact | | | Mid-$p$ | | |
|---|---|---|---|---|---|---|---|---|---|---|
| $N$ | $\tau$ | $E[\xi_s]$ | $E[\pi_s]$ | $\rho$ | MLE | CMAE | UMVCUE | MLE | CMAE | UMVCUE |
| 50 | 0.25 | 0.780 | 0.780 | 0.2 | 97.0 | 96.8 | 96.9 | 95.3 | 95.3 | 95.3 |
| | | | | 0.4 | 97.1 | 97.0 | 97.1 | 95.2 | 95.2 | 95.1 |
| | | | | 0.6 | 97.1 | 97.0 | 97.1 | 95.6 | 95.4 | 95.5 |
| | 0.50 | 0.780 | 0.780 | 0.2 | 96.9 | 96.8 | 96.8 | 95.1 | 95.1 | 95.1 |
| | | | | 0.4 | 97.2 | 97.0 | 97.1 | 95.3 | 95.2 | 95.2 |
| | | | | 0.6 | 97.2 | 97.1 | 97.1 | 95.4 | 95.2 | 95.3 |
| | 0.75 | 0.780 | 0.780 | 0.2 | 96.9 | 96.9 | 96.8 | 95.2 | 95.1 | 95.0 |
| | | | | 0.4 | 97.2 | 97.0 | 96.9 | 95.7 | 95.5 | 95.5 |
| | | | | 0.6 | 97.6 | 97.4 | 97.3 | 96.0 | 95.8 | 95.8 |
| 200 | 0.25 | 0.645 | 0.645 | 0.2 | 95.9 | 95.9 | 95.9 | 94.8 | 94.9 | 94.9 |
| | | | | 0.4 | 95.8 | 95.8 | 95.8 | 94.7 | 94.7 | 94.7 |
| | | | | 0.6 | 96.0 | 95.9 | 96.0 | 95.0 | 94.9 | 94.9 |
| | 0.50 | 0.645 | 0.645 | 0.2 | 96.0 | 96.0 | 96.0 | 95.2 | 95.1 | 95.1 |
| | | | | 0.4 | 96.0 | 96.0 | 96.0 | 95.2 | 95.1 | 95.1 |
| | | | | 0.6 | 96.4 | 96.2 | 96.3 | 95.4 | 95.2 | 95.4 |
| | 0.75 | 0.645 | 0.645 | 0.2 | 95.8 | 95.8 | 95.9 | 94.9 | 94.8 | 94.9 |
| | | | | 0.4 | 96.2 | 96.1 | 96.0 | 95.1 | 95.0 | 95.0 |
| | | | | 0.6 | 96.3 | 96.1 | 96.1 | 95.4 | 95.3 | 95.2 |

Table S96: Coverage probability (%) when the alternative hypothesis is true, and all treatments are effective, and $G = 3$.

| | | | | | Exact | | | Mid-$p$ | | |
|---|---|---|---|---|---|---|---|---|---|---|
| $N$ | $\tau$ | $\mathrm{E}[\xi_s]$ | $\mathrm{E}[\pi_s]$ | $\rho$ | MLE | CMAE | UMVCUE | MLE | CMAE | UMVCUE |
| 50 | 0.25 | 0.780 | 0.780 | 0.2 | 97.2 | 97.1 | 97.2 | 95.4 | 95.4 | 95.4 |
| | | | | 0.4 | 97.2 | 97.0 | 97.2 | 95.4 | 95.3 | 95.4 |
| | | | | 0.6 | 97.3 | 97.1 | 97.3 | 95.6 | 95.4 | 95.5 |
| | 0.50 | 0.780 | 0.780 | 0.2 | 96.9 | 96.8 | 96.8 | 95.1 | 95.1 | 95.0 |
| | | | | 0.4 | 96.8 | 96.7 | 96.6 | 95.0 | 94.9 | 95.0 |
| | | | | 0.6 | 97.3 | 97.2 | 97.2 | 95.8 | 95.6 | 95.7 |
| | 0.75 | 0.780 | 0.780 | 0.2 | 97.0 | 96.8 | 96.8 | 95.5 | 95.5 | 95.4 |
| | | | | 0.4 | 97.3 | 97.2 | 97.1 | 95.7 | 95.5 | 95.3 |
| | | | | 0.6 | 97.8 | 97.5 | 97.4 | 96.3 | 96.0 | 95.7 |
| 200 | 0.25 | 0.645 | 0.645 | 0.2 | 96.0 | 96.0 | 96.0 | 94.8 | 94.9 | 94.9 |
| | | | | 0.4 | 96.0 | 95.9 | 95.9 | 94.9 | 94.9 | 94.9 |
| | | | | 0.6 | 96.2 | 96.1 | 96.2 | 95.3 | 95.1 | 95.3 |
| | 0.50 | 0.645 | 0.645 | 0.2 | 95.8 | 95.9 | 95.9 | 94.9 | 94.8 | 94.8 |
| | | | | 0.4 | 96.1 | 96.1 | 96.0 | 95.2 | 95.0 | 95.1 |
| | | | | 0.6 | 96.1 | 95.8 | 95.9 | 95.1 | 94.8 | 95.0 |
| | 0.75 | 0.645 | 0.645 | 0.2 | 96.2 | 96.1 | 96.1 | 95.4 | 95.2 | 95.2 |
| | | | | 0.4 | 95.9 | 95.8 | 95.8 | 94.9 | 94.7 | 94.5 |
| | | | | 0.6 | 96.6 | 96.4 | 96.2 | 95.7 | 95.5 | 95.3 |

Table S97: Coverage probability (%) when the alternative hypothesis is true, and all treatments are effective, and $G = 4$.

| | | | | | Exact | | | Mid-$p$ | | |
|---|---|---|---|---|---|---|---|---|---|---|
| $N$ | $\tau$ | $\mathrm{E}[\xi_s]$ | $\mathrm{E}[\pi_s]$ | $\rho$ | MLE | CMAE | UMVCUE | MLE | CMAE | UMVCUE |
| 50 | 0.25 | 0.780 | 0.780 | 0.2 | 96.8 | 96.7 | 96.8 | 95.3 | 95.3 | 95.3 |
| | | | | 0.4 | 97.2 | 97.0 | 97.1 | 95.3 | 95.1 | 95.2 |
| | | | | 0.6 | 97.0 | 96.8 | 97.0 | 95.5 | 95.3 | 95.4 |
| | 0.50 | 0.780 | 0.780 | 0.2 | 97.1 | 97.0 | 97.0 | 95.5 | 95.3 | 95.3 |
| | | | | 0.4 | 97.2 | 97.1 | 97.0 | 95.7 | 95.4 | 95.4 |
| | | | | 0.6 | 97.0 | 96.7 | 96.8 | 95.3 | 95.0 | 95.0 |
| | 0.75 | 0.780 | 0.780 | 0.2 | 96.7 | 96.6 | 96.5 | 95.0 | 94.9 | 94.7 |
| | | | | 0.4 | 97.3 | 97.1 | 96.8 | 95.8 | 95.5 | 95.0 |
| | | | | 0.6 | 97.2 | 96.9 | 96.6 | 95.5 | 95.4 | 94.9 |
| 200 | 0.25 | 0.645 | 0.645 | 0.2 | 96.2 | 96.2 | 96.2 | 95.2 | 95.2 | 95.2 |
| | | | | 0.4 | 96.2 | 96.1 | 96.2 | 95.0 | 95.0 | 95.0 |
| | | | | 0.6 | 96.3 | 96.2 | 96.3 | 95.2 | 95.2 | 95.2 |
| | 0.50 | 0.645 | 0.645 | 0.2 | 96.2 | 96.1 | 96.1 | 95.1 | 95.2 | 95.1 |
| | | | | 0.4 | 96.3 | 96.3 | 96.2 | 95.4 | 95.3 | 95.3 |
| | | | | 0.6 | 96.3 | 96.2 | 96.2 | 95.3 | 95.2 | 95.2 |
| | 0.75 | 0.645 | 0.645 | 0.2 | 96.2 | 96.2 | 96.1 | 95.2 | 95.2 | 95.1 |
| | | | | 0.4 | 96.5 | 96.3 | 96.0 | 95.5 | 95.4 | 95.0 |
| | | | | 0.6 | 96.5 | 96.4 | 96.0 | 95.6 | 95.5 | 95.0 |

Table S98: Probability (%) that the lower confidence limit is greater than the true value when the null hypothesis is true, $\xi_g = \pi_g = 0.1$, and $G = 2$.

| $N$ | $\tau$ | E[$\xi_s$] | E[$\pi_s$] | $\rho$ | Exact MLE | Exact CMAE | Exact UMVCUE | Mid-$p$ MLE | Mid-$p$ CMAE | Mid-$p$ UMVCUE |
|---|---|---|---|---|---|---|---|---|---|---|
| 50 | 0.25 | 0.100 | 0.100 | 0.2 | 0.97 | 0.96 | 0.97 | 1.90 | 1.89 | 1.91 |
| | | | | 0.4 | 1.07 | 1.05 | 1.07 | 2.08 | 2.04 | 2.08 |
| | | | | 0.6 | 1.17 | 1.14 | 1.17 | 2.21 | 2.14 | 2.20 |
| | 0.50 | 0.100 | 0.100 | 0.2 | 1.01 | 1.00 | 1.00 | 2.00 | 1.96 | 1.99 |
| | | | | 0.4 | 1.08 | 1.04 | 1.07 | 2.08 | 2.02 | 2.06 |
| | | | | 0.6 | 1.19 | 1.12 | 1.17 | 2.21 | 2.11 | 2.18 |
| | 0.75 | 0.100 | 0.100 | 0.2 | 1.00 | 0.98 | 0.99 | 2.03 | 2.00 | 2.02 |
| | | | | 0.4 | 1.13 | 1.08 | 1.11 | 2.21 | 2.10 | 2.20 |
| | | | | 0.6 | 1.17 | 1.10 | 1.15 | 2.26 | 2.09 | 2.25 |
| 200 | 0.25 | 0.100 | 0.100 | 0.2 | 1.69 | 1.65 | 1.68 | 2.37 | 2.31 | 2.36 |
| | | | | 0.4 | 1.82 | 1.75 | 1.80 | 2.52 | 2.45 | 2.50 |
| | | | | 0.6 | 1.79 | 1.70 | 1.77 | 2.49 | 2.39 | 2.47 |
| | 0.50 | 0.100 | 0.100 | 0.2 | 1.86 | 1.81 | 1.84 | 2.65 | 2.57 | 2.61 |
| | | | | 0.4 | 1.78 | 1.68 | 1.74 | 2.47 | 2.36 | 2.43 |
| | | | | 0.6 | 1.80 | 1.66 | 1.76 | 2.52 | 2.32 | 2.46 |
| | 0.75 | 0.100 | 0.100 | 0.2 | 1.82 | 1.74 | 1.77 | 2.53 | 2.43 | 2.47 |
| | | | | 0.4 | 1.93 | 1.80 | 1.86 | 2.69 | 2.51 | 2.60 |
| | | | | 0.6 | 1.89 | 1.71 | 1.83 | 2.72 | 2.47 | 2.63 |

Table S99: Probability (%) that the lower confidence limit is greater than the true value when the null hypothesis is true, $\xi_g = \pi_g = 0.1$, and $G = 3$.

| $N$ | $\tau$ | E[$\xi_s$] | E[$\pi_s$] | $\rho$ | Exact MLE | Exact CMAE | Exact UMVCUE | Mid-$p$ MLE | Mid-$p$ CMAE | Mid-$p$ UMVCUE |
|---|---|---|---|---|---|---|---|---|---|---|
| 50 | 0.25 | 0.100 | 0.100 | 0.2 | 1.06 | 1.05 | 1.06 | 2.01 | 1.96 | 2.00 |
| | | | | 0.4 | 1.10 | 1.07 | 1.09 | 2.13 | 2.04 | 2.11 |
| | | | | 0.6 | 1.23 | 1.14 | 1.21 | 2.30 | 2.14 | 2.26 |
| | 0.50 | 0.100 | 0.100 | 0.2 | 1.05 | 1.03 | 1.05 | 2.05 | 1.97 | 2.02 |
| | | | | 0.4 | 1.17 | 1.09 | 1.13 | 2.22 | 2.08 | 2.15 |
| | | | | 0.6 | 1.24 | 1.12 | 1.20 | 2.27 | 2.06 | 2.19 |
| | 0.75 | 0.100 | 0.100 | 0.2 | 1.06 | 1.03 | 1.04 | 2.05 | 1.96 | 2.01 |
| | | | | 0.4 | 1.20 | 1.10 | 1.16 | 2.30 | 2.09 | 2.20 |
| | | | | 0.6 | 1.24 | 1.10 | 1.20 | 2.29 | 2.03 | 2.22 |
| 200 | 0.25 | 0.100 | 0.100 | 0.2 | 1.72 | 1.67 | 1.71 | 2.46 | 2.38 | 2.43 |
| | | | | 0.4 | 1.88 | 1.77 | 1.85 | 2.59 | 2.46 | 2.55 |
| | | | | 0.6 | 1.82 | 1.70 | 1.80 | 2.55 | 2.35 | 2.50 |
| | 0.50 | 0.100 | 0.100 | 0.2 | 1.74 | 1.67 | 1.70 | 2.48 | 2.38 | 2.43 |
| | | | | 0.4 | 1.80 | 1.65 | 1.73 | 2.53 | 2.35 | 2.45 |
| | | | | 0.6 | 1.88 | 1.70 | 1.81 | 2.59 | 2.33 | 2.48 |
| | 0.75 | 0.100 | 0.100 | 0.2 | 1.84 | 1.74 | 1.76 | 2.60 | 2.44 | 2.47 |
| | | | | 0.4 | 1.94 | 1.74 | 1.80 | 2.78 | 2.52 | 2.60 |
| | | | | 0.6 | 1.82 | 1.57 | 1.71 | 2.59 | 2.30 | 2.44 |

Table S100: Probability (%) that the lower confidence limit is greater than the true value when the null hypothesis is true, $\xi_g = \pi_g = 0.1$, and $G = 4$.

| | | | | | Exact | | | Mid-$p$ | | |
|---|---|---|---|---|---|---|---|---|---|---|
| $N$ | $\tau$ | E[$\xi_s$] | E[$\pi_s$] | $\rho$ | MLE | CMAE | UMVCUE | MLE | CMAE | UMVCUE |
| 50 | 0.25 | 0.100 | 0.100 | 0.2 | 1.09 | 1.07 | 1.09 | 2.07 | 2.02 | 2.06 |
| | | | | 0.4 | 1.22 | 1.14 | 1.20 | 2.26 | 2.14 | 2.23 |
| | | | | 0.6 | 1.18 | 1.07 | 1.16 | 2.28 | 2.07 | 2.23 |
| | 0.50 | 0.100 | 0.100 | 0.2 | 1.15 | 1.11 | 1.13 | 2.19 | 2.11 | 2.15 |
| | | | | 0.4 | 1.15 | 1.05 | 1.10 | 2.21 | 2.02 | 2.13 |
| | | | | 0.6 | 1.26 | 1.10 | 1.20 | 2.29 | 2.04 | 2.18 |
| | 0.75 | 0.100 | 0.100 | 0.2 | 1.20 | 1.13 | 1.15 | 2.27 | 2.12 | 2.21 |
| | | | | 0.4 | 1.29 | 1.12 | 1.21 | 2.37 | 2.09 | 2.24 |
| | | | | 0.6 | 1.25 | 1.08 | 1.19 | 2.36 | 2.02 | 2.22 |
| 200 | 0.25 | 0.100 | 0.100 | 0.2 | 1.82 | 1.75 | 1.79 | 2.51 | 2.43 | 2.48 |
| | | | | 0.4 | 1.89 | 1.76 | 1.84 | 2.60 | 2.46 | 2.54 |
| | | | | 0.6 | 1.77 | 1.62 | 1.72 | 2.51 | 2.29 | 2.45 |
| | 0.50 | 0.100 | 0.100 | 0.2 | 1.81 | 1.70 | 1.74 | 2.59 | 2.44 | 2.49 |
| | | | | 0.4 | 1.84 | 1.64 | 1.72 | 2.65 | 2.40 | 2.50 |
| | | | | 0.6 | 1.89 | 1.66 | 1.77 | 2.70 | 2.35 | 2.53 |
| | 0.75 | 0.100 | 0.100 | 0.2 | 1.88 | 1.72 | 1.76 | 2.67 | 2.46 | 2.49 |
| | | | | 0.4 | 1.98 | 1.72 | 1.78 | 2.79 | 2.44 | 2.54 |
| | | | | 0.6 | 1.93 | 1.65 | 1.78 | 2.72 | 2.32 | 2.49 |

Table S101: Probability (%) that the lower confidence limit is greater than the true value when the null hypothesis is true, $\xi_g = \pi_g = 0.5$, and $G = 2$.

| | | | | | Exact | | | Mid-$p$ | | |
|---|---|---|---|---|---|---|---|---|---|---|
| $N$ | $\tau$ | E[$\xi_s$] | E[$\pi_s$] | $\rho$ | MLE | CMAE | UMVCUE | MLE | CMAE | UMVCUE |
| 50 | 0.25 | 0.500 | 0.500 | 0.2 | 1.77 | 1.68 | 1.75 | 2.36 | 2.29 | 2.34 |
| | | | | 0.4 | 1.68 | 1.57 | 1.65 | 2.24 | 2.17 | 2.23 |
| | | | | 0.6 | 1.67 | 1.58 | 1.66 | 2.36 | 2.26 | 2.34 |
| | 0.50 | 0.500 | 0.500 | 0.2 | 1.78 | 1.69 | 1.73 | 2.37 | 2.30 | 2.35 |
| | | | | 0.4 | 1.72 | 1.62 | 1.69 | 2.37 | 2.28 | 2.34 |
| | | | | 0.6 | 1.64 | 1.54 | 1.61 | 2.39 | 2.22 | 2.34 |
| | 0.75 | 0.500 | 0.500 | 0.2 | 1.80 | 1.67 | 1.72 | 2.41 | 2.34 | 2.37 |
| | | | | 0.4 | 1.75 | 1.63 | 1.70 | 2.44 | 2.30 | 2.36 |
| | | | | 0.6 | 1.80 | 1.66 | 1.75 | 2.56 | 2.36 | 2.46 |
| 200 | 0.25 | 0.500 | 0.500 | 0.2 | 2.13 | 2.05 | 2.11 | 2.62 | 2.52 | 2.60 |
| | | | | 0.4 | 2.07 | 2.00 | 2.05 | 2.56 | 2.47 | 2.55 |
| | | | | 0.6 | 2.07 | 1.93 | 2.05 | 2.58 | 2.44 | 2.56 |
| | 0.50 | 0.500 | 0.500 | 0.2 | 2.11 | 2.02 | 2.07 | 2.59 | 2.49 | 2.55 |
| | | | | 0.4 | 2.15 | 2.02 | 2.10 | 2.69 | 2.54 | 2.63 |
| | | | | 0.6 | 2.18 | 2.02 | 2.12 | 2.67 | 2.46 | 2.61 |
| | 0.75 | 0.500 | 0.500 | 0.2 | 2.08 | 1.97 | 2.01 | 2.59 | 2.48 | 2.53 |
| | | | | 0.4 | 2.21 | 2.07 | 2.14 | 2.72 | 2.53 | 2.62 |
| | | | | 0.6 | 2.12 | 1.93 | 2.02 | 2.65 | 2.39 | 2.54 |

Table S102: Probability (%) that the lower confidence limit is greater than the true value when the null hypothesis is true, $\xi_g = \pi_g = 0.5$, and $G = 3$.

| | | | | | Exact | | | Mid-$p$ | | |
|---|---|---|---|---|---|---|---|---|---|---|
| $N$ | $\tau$ | E[$\xi_s$] | E[$\pi_s$] | $\rho$ | MLE | CMAE | UMVCUE | MLE | CMAE | UMVCUE |
| 50 | 0.25 | 0.500 | 0.500 | 0.2 | 1.79 | 1.69 | 1.75 | 2.35 | 2.31 | 2.34 |
| | | | | 0.4 | 1.60 | 1.51 | 1.57 | 2.31 | 2.20 | 2.28 |
| | | | | 0.6 | 1.62 | 1.52 | 1.60 | 2.45 | 2.25 | 2.39 |
| | 0.50 | 0.500 | 0.500 | 0.2 | 1.66 | 1.54 | 1.60 | 2.31 | 2.21 | 2.25 |
| | | | | 0.4 | 1.77 | 1.63 | 1.69 | 2.52 | 2.34 | 2.42 |
| | | | | 0.6 | 1.73 | 1.54 | 1.64 | 2.61 | 2.34 | 2.49 |
| | 0.75 | 0.500 | 0.500 | 0.2 | 1.73 | 1.61 | 1.66 | 2.44 | 2.31 | 2.32 |
| | | | | 0.4 | 1.76 | 1.56 | 1.63 | 2.62 | 2.36 | 2.44 |
| | | | | 0.6 | 1.82 | 1.61 | 1.72 | 2.70 | 2.35 | 2.52 |
| 200 | 0.25 | 0.500 | 0.500 | 0.2 | 2.12 | 2.01 | 2.07 | 2.55 | 2.48 | 2.53 |
| | | | | 0.4 | 2.01 | 1.88 | 1.97 | 2.50 | 2.36 | 2.46 |
| | | | | 0.6 | 2.16 | 2.01 | 2.13 | 2.65 | 2.46 | 2.60 |
| | 0.50 | 0.500 | 0.500 | 0.2 | 2.08 | 1.95 | 1.99 | 2.54 | 2.42 | 2.47 |
| | | | | 0.4 | 2.15 | 1.97 | 2.05 | 2.67 | 2.44 | 2.54 |
| | | | | 0.6 | 2.22 | 1.96 | 2.12 | 2.71 | 2.42 | 2.59 |
| | 0.75 | 0.500 | 0.500 | 0.2 | 2.12 | 1.99 | 2.00 | 2.60 | 2.42 | 2.46 |
| | | | | 0.4 | 2.28 | 2.01 | 2.09 | 2.76 | 2.48 | 2.55 |
| | | | | 0.6 | 2.21 | 1.95 | 2.08 | 2.78 | 2.39 | 2.58 |

Table S103: Probability (%) that the lower confidence limit is greater than the true value when the null hypothesis is true, $\xi_g = \pi_g = 0.5$, and $G = 4$.

| | | | | | Exact | | | Mid-$p$ | | |
|---|---|---|---|---|---|---|---|---|---|---|
| $N$ | $\tau$ | E[$\xi_s$] | E[$\pi_s$] | $\rho$ | MLE | CMAE | UMVCUE | MLE | CMAE | UMVCUE |
| 50 | 0.25 | 0.500 | 0.500 | 0.2 | 1.72 | 1.63 | 1.68 | 2.36 | 2.29 | 2.33 |
| | | | | 0.4 | 1.60 | 1.48 | 1.55 | 2.39 | 2.25 | 2.34 |
| | | | | 0.6 | 1.68 | 1.53 | 1.63 | 2.53 | 2.30 | 2.44 |
| | 0.50 | 0.500 | 0.500 | 0.2 | 1.66 | 1.55 | 1.60 | 2.33 | 2.22 | 2.26 |
| | | | | 0.4 | 1.69 | 1.52 | 1.59 | 2.54 | 2.26 | 2.38 |
| | | | | 0.6 | 1.73 | 1.50 | 1.61 | 2.60 | 2.25 | 2.42 |
| | 0.75 | 0.500 | 0.500 | 0.2 | 1.85 | 1.70 | 1.73 | 2.50 | 2.38 | 2.37 |
| | | | | 0.4 | 1.77 | 1.54 | 1.59 | 2.63 | 2.30 | 2.37 |
| | | | | 0.6 | 1.78 | 1.49 | 1.62 | 2.67 | 2.29 | 2.46 |
| 200 | 0.25 | 0.500 | 0.500 | 0.2 | 2.09 | 1.99 | 2.05 | 2.55 | 2.47 | 2.52 |
| | | | | 0.4 | 2.10 | 1.96 | 2.05 | 2.62 | 2.40 | 2.54 |
| | | | | 0.6 | 2.06 | 1.87 | 2.01 | 2.57 | 2.32 | 2.48 |
| | 0.50 | 0.500 | 0.500 | 0.2 | 2.15 | 2.01 | 2.05 | 2.64 | 2.48 | 2.53 |
| | | | | 0.4 | 2.15 | 1.93 | 2.02 | 2.63 | 2.36 | 2.46 |
| | | | | 0.6 | 2.12 | 1.81 | 1.96 | 2.67 | 2.29 | 2.47 |
| | 0.75 | 0.500 | 0.500 | 0.2 | 2.13 | 1.96 | 1.98 | 2.65 | 2.43 | 2.46 |
| | | | | 0.4 | 2.25 | 1.94 | 2.00 | 2.76 | 2.39 | 2.46 |
| | | | | 0.6 | 2.19 | 1.82 | 1.97 | 2.76 | 2.31 | 2.50 |

Table S104: Probability (%) that the lower confidence limit is greater than the true value when the null hypothesis is true, $\xi_g = \pi_g = 0.7$, and $G = 2$.

| | | | | | Exact | | | Mid-$p$ | | |
|---|---|---|---|---|---|---|---|---|---|---|
| $N$ | $\tau$ | E[$\xi_s$] | E[$\pi_s$] | $\rho$ | MLE | CMAE | UMVCUE | MLE | CMAE | UMVCUE |
| 50 | 0.25 | 0.700 | 0.700 | 0.2 | 1.56 | 1.52 | 1.54 | 2.37 | 2.31 | 2.36 |
| | | | | 0.4 | 1.58 | 1.50 | 1.55 | 2.43 | 2.32 | 2.41 |
| | | | | 0.6 | 1.57 | 1.47 | 1.55 | 2.43 | 2.29 | 2.41 |
| | 0.50 | 0.700 | 0.700 | 0.2 | 1.60 | 1.54 | 1.58 | 2.47 | 2.36 | 2.43 |
| | | | | 0.4 | 1.61 | 1.52 | 1.57 | 2.52 | 2.37 | 2.46 |
| | | | | 0.6 | 1.64 | 1.51 | 1.59 | 2.56 | 2.34 | 2.50 |
| | 0.75 | 0.700 | 0.700 | 0.2 | 1.66 | 1.57 | 1.60 | 2.52 | 2.42 | 2.47 |
| | | | | 0.4 | 1.59 | 1.48 | 1.53 | 2.41 | 2.22 | 2.32 |
| | | | | 0.6 | 1.63 | 1.49 | 1.58 | 2.53 | 2.29 | 2.45 |
| 200 | 0.25 | 0.700 | 0.700 | 0.2 | 2.04 | 1.99 | 2.02 | 2.53 | 2.47 | 2.52 |
| | | | | 0.4 | 2.06 | 1.98 | 2.04 | 2.58 | 2.48 | 2.56 |
| | | | | 0.6 | 2.03 | 1.90 | 2.00 | 2.58 | 2.41 | 2.55 |
| | 0.50 | 0.700 | 0.700 | 0.2 | 2.04 | 1.97 | 2.00 | 2.55 | 2.46 | 2.50 |
| | | | | 0.4 | 2.10 | 1.96 | 2.04 | 2.67 | 2.47 | 2.59 |
| | | | | 0.6 | 2.06 | 1.91 | 2.02 | 2.64 | 2.42 | 2.56 |
| | 0.75 | 0.700 | 0.700 | 0.2 | 2.00 | 1.91 | 1.93 | 2.48 | 2.38 | 2.40 |
| | | | | 0.4 | 2.03 | 1.86 | 1.94 | 2.59 | 2.37 | 2.47 |
| | | | | 0.6 | 2.19 | 1.99 | 2.12 | 2.70 | 2.48 | 2.62 |

Table S105: Probability (%) that the lower confidence limit is greater than the true value when the null hypothesis is true, $\xi_g = \pi_g = 0.7$, and $G = 3$.

| | | | | | Exact | | | Mid-$p$ | | |
|---|---|---|---|---|---|---|---|---|---|---|
| $N$ | $\tau$ | E[$\xi_s$] | E[$\pi_s$] | $\rho$ | MLE | CMAE | UMVCUE | MLE | CMAE | UMVCUE |
| 50 | 0.25 | 0.700 | 0.700 | 0.2 | 1.53 | 1.47 | 1.51 | 2.40 | 2.32 | 2.36 |
| | | | | 0.4 | 1.57 | 1.47 | 1.53 | 2.43 | 2.29 | 2.39 |
| | | | | 0.6 | 1.54 | 1.42 | 1.51 | 2.38 | 2.18 | 2.33 |
| | 0.50 | 0.700 | 0.700 | 0.2 | 1.52 | 1.43 | 1.47 | 2.36 | 2.25 | 2.31 |
| | | | | 0.4 | 1.67 | 1.52 | 1.58 | 2.58 | 2.37 | 2.46 |
| | | | | 0.6 | 1.64 | 1.47 | 1.57 | 2.50 | 2.22 | 2.39 |
| | 0.75 | 0.700 | 0.700 | 0.2 | 1.63 | 1.54 | 1.56 | 2.48 | 2.32 | 2.35 |
| | | | | 0.4 | 1.68 | 1.50 | 1.57 | 2.56 | 2.29 | 2.39 |
| | | | | 0.6 | 1.68 | 1.41 | 1.57 | 2.62 | 2.26 | 2.44 |
| 200 | 0.25 | 0.700 | 0.700 | 0.2 | 2.02 | 1.96 | 1.99 | 2.54 | 2.43 | 2.49 |
| | | | | 0.4 | 2.01 | 1.89 | 1.97 | 2.48 | 2.34 | 2.43 |
| | | | | 0.6 | 1.98 | 1.81 | 1.93 | 2.51 | 2.30 | 2.46 |
| | 0.50 | 0.700 | 0.700 | 0.2 | 2.02 | 1.94 | 1.97 | 2.56 | 2.43 | 2.48 |
| | | | | 0.4 | 2.13 | 1.93 | 2.01 | 2.71 | 2.48 | 2.58 |
| | | | | 0.6 | 2.12 | 1.88 | 2.00 | 2.71 | 2.39 | 2.58 |
| | 0.75 | 0.700 | 0.700 | 0.2 | 2.08 | 1.96 | 1.96 | 2.68 | 2.50 | 2.51 |
| | | | | 0.4 | 2.23 | 1.98 | 2.06 | 2.83 | 2.52 | 2.64 |
| | | | | 0.6 | 2.19 | 1.89 | 2.03 | 2.80 | 2.39 | 2.59 |

Table S106: Probability (%) that the lower confidence limit is greater than the true value when the null hypothesis is true, $\xi_g = \pi_g = 0.7$, and $G = 4$.

| | | | | | Exact | | | Mid-$p$ | | |
|---|---|---|---|---|---|---|---|---|---|---|
| $N$ | $\tau$ | E[$\xi_s$] | E[$\pi_s$] | $\rho$ | MLE | CMAE | UMVCUE | MLE | CMAE | UMVCUE |
| 50 | 0.25 | 0.700 | 0.700 | 0.2 | 1.61 | 1.55 | 1.58 | 2.43 | 2.34 | 2.39 |
| | | | | 0.4 | 1.57 | 1.47 | 1.52 | 2.45 | 2.29 | 2.38 |
| | | | | 0.6 | 1.49 | 1.35 | 1.45 | 2.36 | 2.13 | 2.30 |
| | 0.50 | 0.700 | 0.700 | 0.2 | 1.70 | 1.61 | 1.64 | 2.49 | 2.35 | 2.40 |
| | | | | 0.4 | 1.66 | 1.49 | 1.55 | 2.57 | 2.29 | 2.40 |
| | | | | 0.6 | 1.58 | 1.34 | 1.46 | 2.46 | 2.11 | 2.31 |
| | 0.75 | 0.700 | 0.700 | 0.2 | 1.59 | 1.48 | 1.50 | 2.47 | 2.28 | 2.30 |
| | | | | 0.4 | 1.79 | 1.58 | 1.65 | 2.75 | 2.41 | 2.51 |
| | | | | 0.6 | 1.65 | 1.37 | 1.51 | 2.58 | 2.18 | 2.35 |
| 200 | 0.25 | 0.700 | 0.700 | 0.2 | 1.97 | 1.88 | 1.92 | 2.49 | 2.39 | 2.45 |
| | | | | 0.4 | 2.00 | 1.83 | 1.93 | 2.53 | 2.34 | 2.46 |
| | | | | 0.6 | 1.96 | 1.78 | 1.91 | 2.52 | 2.26 | 2.44 |
| | 0.50 | 0.700 | 0.700 | 0.2 | 2.11 | 1.98 | 2.01 | 2.69 | 2.51 | 2.56 |
| | | | | 0.4 | 2.08 | 1.86 | 1.94 | 2.66 | 2.36 | 2.46 |
| | | | | 0.6 | 2.18 | 1.88 | 2.04 | 2.74 | 2.35 | 2.55 |
| | 0.75 | 0.700 | 0.700 | 0.2 | 2.13 | 1.94 | 1.96 | 2.69 | 2.46 | 2.47 |
| | | | | 0.4 | 2.29 | 1.98 | 2.05 | 2.88 | 2.49 | 2.58 |
| | | | | 0.6 | 2.20 | 1.83 | 2.00 | 2.79 | 2.32 | 2.53 |

Table S107: Probability (%) that the lower confidence limit is greater than the true value when the null hypothesis is true, $\xi_g$ =0.3, $\pi_g = 0.5$, and $G = 2$.

| | | | | | Exact | | | Mid-$p$ | | |
|---|---|---|---|---|---|---|---|---|---|---|
| $N$ | $\tau$ | E[$\xi_s$] | E[$\pi_s$] | $\rho$ | MLE | CMAE | UMVCUE | MLE | CMAE | UMVCUE |
| 50 | 0.25 | 0.300 | 0.500 | 0.2 | 1.83 | 1.76 | 1.82 | 2.42 | 2.36 | 2.41 |
| | | | | 0.4 | 1.85 | 1.75 | 1.83 | 2.42 | 2.37 | 2.41 |
| | | | | 0.5 | 1.71 | 1.61 | 1.70 | 2.30 | 2.26 | 2.30 |
| | 0.50 | 0.300 | 0.500 | 0.2 | 1.79 | 1.69 | 1.73 | 2.34 | 2.27 | 2.32 |
| | | | | 0.4 | 1.71 | 1.60 | 1.68 | 2.40 | 2.31 | 2.38 |
| | | | | 0.5 | 1.76 | 1.68 | 1.75 | 2.48 | 2.38 | 2.45 |
| | 0.75 | 0.300 | 0.500 | 0.2 | 1.80 | 1.71 | 1.76 | 2.41 | 2.33 | 2.38 |
| | | | | 0.4 | 1.77 | 1.64 | 1.72 | 2.48 | 2.35 | 2.41 |
| | | | | 0.5 | 1.79 | 1.66 | 1.75 | 2.55 | 2.36 | 2.44 |
| 200 | 0.25 | 0.300 | 0.500 | 0.2 | 2.06 | 1.97 | 2.04 | 2.58 | 2.49 | 2.55 |
| | | | | 0.4 | 2.06 | 1.97 | 2.04 | 2.53 | 2.44 | 2.52 |
| | | | | 0.5 | 2.01 | 1.91 | 2.00 | 2.51 | 2.39 | 2.50 |
| | 0.50 | 0.300 | 0.500 | 0.2 | 2.16 | 2.05 | 2.11 | 2.66 | 2.56 | 2.62 |
| | | | | 0.4 | 2.13 | 2.01 | 2.08 | 2.60 | 2.47 | 2.55 |
| | | | | 0.5 | 2.17 | 2.04 | 2.12 | 2.68 | 2.51 | 2.61 |
| | 0.75 | 0.300 | 0.500 | 0.2 | 2.13 | 2.02 | 2.07 | 2.61 | 2.51 | 2.56 |
| | | | | 0.4 | 2.22 | 2.07 | 2.14 | 2.75 | 2.56 | 2.64 |
| | | | | 0.5 | 2.14 | 1.96 | 2.05 | 2.64 | 2.44 | 2.55 |

Table S108: Probability (%) that the lower confidence limit is greater than the true value when the null hypothesis is true, $\xi_g$ =0.3, $\pi_g = 0.5$, and $G = 3$.

| | | | | | Exact | | | Mid-$p$ | | |
|---|---|---|---|---|---|---|---|---|---|---|
| $N$ | $\tau$ | E[$\xi_s$] | E[$\pi_s$] | $\rho$ | MLE | CMAE | UMVCUE | MLE | CMAE | UMVCUE |
| 50 | 0.25 | 0.300 | 0.500 | 0.2 | 1.73 | 1.64 | 1.71 | 2.31 | 2.28 | 2.30 |
| | | | | 0.4 | 1.57 | 1.49 | 1.56 | 2.29 | 2.22 | 2.28 |
| | | | | 0.5 | 1.57 | 1.50 | 1.57 | 2.38 | 2.26 | 2.36 |
| | 0.50 | 0.300 | 0.500 | 0.2 | 1.57 | 1.45 | 1.51 | 2.20 | 2.12 | 2.16 |
| | | | | 0.4 | 1.63 | 1.51 | 1.57 | 2.40 | 2.25 | 2.32 |
| | | | | 0.5 | 1.64 | 1.50 | 1.59 | 2.47 | 2.25 | 2.37 |
| | 0.75 | 0.300 | 0.500 | 0.2 | 1.77 | 1.63 | 1.68 | 2.49 | 2.37 | 2.39 |
| | | | | 0.4 | 1.73 | 1.55 | 1.64 | 2.56 | 2.34 | 2.41 |
| | | | | 0.5 | 1.72 | 1.53 | 1.62 | 2.63 | 2.34 | 2.47 |
| 200 | 0.25 | 0.300 | 0.500 | 0.2 | 2.08 | 1.98 | 2.04 | 2.51 | 2.42 | 2.49 |
| | | | | 0.4 | 2.08 | 1.96 | 2.05 | 2.58 | 2.44 | 2.54 |
| | | | | 0.5 | 2.06 | 1.91 | 2.03 | 2.57 | 2.40 | 2.52 |
| | 0.50 | 0.300 | 0.500 | 0.2 | 2.08 | 1.97 | 2.02 | 2.54 | 2.43 | 2.48 |
| | | | | 0.4 | 2.11 | 1.93 | 2.01 | 2.59 | 2.38 | 2.48 |
| | | | | 0.5 | 2.21 | 1.96 | 2.10 | 2.76 | 2.48 | 2.62 |
| | 0.75 | 0.300 | 0.500 | 0.2 | 2.13 | 1.99 | 2.01 | 2.60 | 2.46 | 2.48 |
| | | | | 0.4 | 2.18 | 1.97 | 2.03 | 2.72 | 2.41 | 2.50 |
| | | | | 0.5 | 2.23 | 1.95 | 2.08 | 2.80 | 2.45 | 2.60 |

Table S109: Probability (%) that the lower confidence limit is greater than the true value when the null hypothesis is true, $\xi_g$ =0.3, $\pi_g = 0.5$, and $G = 4$.

| | | | | | Exact | | | Mid-$p$ | | |
|---|---|---|---|---|---|---|---|---|---|---|
| $N$ | $\tau$ | E[$\xi_s$] | E[$\pi_s$] | $\rho$ | MLE | CMAE | UMVCUE | MLE | CMAE | UMVCUE |
| 50 | 0.25 | 0.300 | 0.500 | 0.2 | 1.65 | 1.56 | 1.61 | 2.33 | 2.26 | 2.31 |
| | | | | 0.4 | 1.63 | 1.55 | 1.61 | 2.47 | 2.35 | 2.44 |
| | | | | 0.5 | 1.59 | 1.48 | 1.56 | 2.44 | 2.25 | 2.40 |
| | 0.50 | 0.300 | 0.500 | 0.2 | 1.73 | 1.62 | 1.67 | 2.38 | 2.29 | 2.32 |
| | | | | 0.4 | 1.72 | 1.57 | 1.65 | 2.50 | 2.28 | 2.37 |
| | | | | 0.5 | 1.64 | 1.46 | 1.57 | 2.50 | 2.22 | 2.38 |
| | 0.75 | 0.300 | 0.500 | 0.2 | 1.84 | 1.69 | 1.73 | 2.61 | 2.47 | 2.48 |
| | | | | 0.4 | 1.81 | 1.60 | 1.65 | 2.70 | 2.38 | 2.49 |
| | | | | 0.5 | 1.82 | 1.55 | 1.65 | 2.74 | 2.37 | 2.52 |
| 200 | 0.25 | 0.300 | 0.500 | 0.2 | 2.07 | 1.97 | 2.03 | 2.52 | 2.44 | 2.49 |
| | | | | 0.4 | 2.06 | 1.93 | 2.02 | 2.56 | 2.38 | 2.50 |
| | | | | 0.5 | 2.14 | 1.96 | 2.09 | 2.64 | 2.44 | 2.58 |
| | 0.50 | 0.300 | 0.500 | 0.2 | 2.15 | 2.00 | 2.05 | 2.66 | 2.50 | 2.55 |
| | | | | 0.4 | 2.17 | 1.95 | 2.05 | 2.66 | 2.41 | 2.52 |
| | | | | 0.5 | 2.12 | 1.88 | 2.00 | 2.64 | 2.33 | 2.45 |
| | 0.75 | 0.300 | 0.500 | 0.2 | 2.20 | 2.02 | 2.03 | 2.72 | 2.52 | 2.52 |
| | | | | 0.4 | 2.30 | 1.97 | 2.05 | 2.85 | 2.48 | 2.57 |
| | | | | 0.5 | 2.30 | 1.95 | 2.07 | 2.79 | 2.40 | 2.55 |

Table S110: Probability (%) that the lower confidence limit is greater than the true value when the null hypothesis is true, $\xi_g$ =0.7, $\pi_g = 0.5$, and $G = 2$.

| | | | | | Exact | | | Mid-$p$ | | |
|---|---|---|---|---|---|---|---|---|---|---|
| $N$ | $\tau$ | E[$\xi_s$] | E[$\pi_s$] | $\rho$ | MLE | CMAE | UMVCUE | MLE | CMAE | UMVCUE |
| 50 | 0.25 | 0.700 | 0.500 | 0.2 | 1.73 | 1.65 | 1.71 | 2.29 | 2.22 | 2.27 |
| | | | | 0.4 | 1.72 | 1.59 | 1.68 | 2.29 | 2.23 | 2.27 |
| | | | | 0.5 | 1.73 | 1.59 | 1.69 | 2.33 | 2.25 | 2.31 |
| | 0.50 | 0.700 | 0.500 | 0.2 | 1.73 | 1.65 | 1.69 | 2.33 | 2.26 | 2.30 |
| | | | | 0.4 | 1.73 | 1.62 | 1.69 | 2.36 | 2.25 | 2.30 |
| | | | | 0.5 | 1.69 | 1.56 | 1.65 | 2.37 | 2.24 | 2.31 |
| | 0.75 | 0.700 | 0.500 | 0.2 | 1.80 | 1.67 | 1.74 | 2.43 | 2.37 | 2.38 |
| | | | | 0.4 | 1.78 | 1.66 | 1.72 | 2.49 | 2.32 | 2.38 |
| | | | | 0.5 | 1.84 | 1.69 | 1.76 | 2.54 | 2.38 | 2.43 |
| 200 | 0.25 | 0.700 | 0.500 | 0.2 | 2.04 | 1.97 | 2.02 | 2.51 | 2.42 | 2.48 |
| | | | | 0.4 | 2.03 | 1.93 | 2.00 | 2.47 | 2.39 | 2.45 |
| | | | | 0.5 | 2.11 | 2.02 | 2.09 | 2.61 | 2.48 | 2.58 |
| | 0.50 | 0.700 | 0.500 | 0.2 | 2.07 | 1.97 | 2.02 | 2.52 | 2.44 | 2.48 |
| | | | | 0.4 | 2.19 | 2.04 | 2.12 | 2.69 | 2.53 | 2.62 |
| | | | | 0.5 | 2.09 | 1.95 | 2.02 | 2.56 | 2.39 | 2.49 |
| | 0.75 | 0.700 | 0.500 | 0.2 | 2.22 | 2.10 | 2.15 | 2.69 | 2.57 | 2.61 |
| | | | | 0.4 | 2.22 | 2.06 | 2.13 | 2.75 | 2.56 | 2.63 |
| | | | | 0.5 | 2.26 | 2.08 | 2.16 | 2.80 | 2.56 | 2.70 |

Table S111: Probability (%) that the lower confidence limit is greater than the true value when the null hypothesis is true, $\xi_g$ =0.7, $\pi_g = 0.5$, and $G = 3$.

| | | | | | Exact | | | Mid-$p$ | | |
|---|---|---|---|---|---|---|---|---|---|---|
| $N$ | $\tau$ | E[$\xi_s$] | E[$\pi_s$] | $\rho$ | MLE | CMAE | UMVCUE | MLE | CMAE | UMVCUE |
| 50 | 0.25 | 0.700 | 0.500 | 0.2 | 1.64 | 1.55 | 1.60 | 2.25 | 2.19 | 2.23 |
| | | | | 0.4 | 1.68 | 1.55 | 1.64 | 2.36 | 2.27 | 2.33 |
| | | | | 0.5 | 1.55 | 1.44 | 1.52 | 2.38 | 2.22 | 2.31 |
| | 0.50 | 0.700 | 0.500 | 0.2 | 1.78 | 1.67 | 1.72 | 2.45 | 2.35 | 2.39 |
| | | | | 0.4 | 1.77 | 1.63 | 1.70 | 2.56 | 2.35 | 2.43 |
| | | | | 0.5 | 1.70 | 1.54 | 1.62 | 2.57 | 2.31 | 2.41 |
| | 0.75 | 0.700 | 0.500 | 0.2 | 1.79 | 1.65 | 1.68 | 2.48 | 2.35 | 2.35 |
| | | | | 0.4 | 1.78 | 1.59 | 1.66 | 2.60 | 2.30 | 2.36 |
| | | | | 0.5 | 1.75 | 1.53 | 1.61 | 2.60 | 2.24 | 2.37 |
| 200 | 0.25 | 0.700 | 0.500 | 0.2 | 2.08 | 1.98 | 2.04 | 2.53 | 2.43 | 2.50 |
| | | | | 0.4 | 2.04 | 1.90 | 1.99 | 2.54 | 2.38 | 2.48 |
| | | | | 0.5 | 2.01 | 1.84 | 1.97 | 2.52 | 2.32 | 2.46 |
| | 0.50 | 0.700 | 0.500 | 0.2 | 2.10 | 1.97 | 2.02 | 2.58 | 2.45 | 2.51 |
| | | | | 0.4 | 2.15 | 1.95 | 2.03 | 2.69 | 2.45 | 2.54 |
| | | | | 0.5 | 2.17 | 1.92 | 2.04 | 2.70 | 2.39 | 2.54 |
| | 0.75 | 0.700 | 0.500 | 0.2 | 2.11 | 1.95 | 1.99 | 2.61 | 2.44 | 2.46 |
| | | | | 0.4 | 2.28 | 2.01 | 2.07 | 2.81 | 2.49 | 2.56 |
| | | | | 0.5 | 2.26 | 1.94 | 2.07 | 2.85 | 2.43 | 2.58 |

Table S112: Probability (%) that the lower confidence limit is greater than the true value when the null hypothesis is true, $\xi_g$ =0.7, $\pi_g = 0.5$, and $G = 4$.

| | | | | | Exact | | | Mid-$p$ | | |
|---|---|---|---|---|---|---|---|---|---|---|
| $N$ | $\tau$ | E[$\xi_s$] | E[$\pi_s$] | $\rho$ | MLE | CMAE | UMVCUE | MLE | CMAE | UMVCUE |
| 50 | 0.25 | 0.700 | 0.500 | 0.2 | 1.70 | 1.59 | 1.64 | 2.33 | 2.26 | 2.30 |
| | | | | 0.4 | 1.59 | 1.48 | 1.54 | 2.37 | 2.20 | 2.29 |
| | | | | 0.5 | 1.62 | 1.49 | 1.55 | 2.48 | 2.26 | 2.39 |
| | 0.50 | 0.700 | 0.500 | 0.2 | 1.72 | 1.56 | 1.62 | 2.44 | 2.32 | 2.35 |
| | | | | 0.4 | 1.74 | 1.55 | 1.62 | 2.59 | 2.29 | 2.38 |
| | | | | 0.5 | 1.78 | 1.57 | 1.66 | 2.65 | 2.29 | 2.41 |
| | 0.75 | 0.700 | 0.500 | 0.2 | 1.79 | 1.61 | 1.63 | 2.56 | 2.38 | 2.37 |
| | | | | 0.4 | 1.85 | 1.60 | 1.66 | 2.75 | 2.37 | 2.46 |
| | | | | 0.5 | 1.80 | 1.48 | 1.58 | 2.77 | 2.28 | 2.43 |
| 200 | 0.25 | 0.700 | 0.500 | 0.2 | 2.02 | 1.92 | 1.99 | 2.52 | 2.42 | 2.48 |
| | | | | 0.4 | 1.99 | 1.83 | 1.92 | 2.50 | 2.30 | 2.44 |
| | | | | 0.5 | 2.14 | 1.93 | 2.05 | 2.66 | 2.41 | 2.56 |
| | 0.50 | 0.700 | 0.500 | 0.2 | 2.11 | 1.96 | 2.01 | 2.58 | 2.41 | 2.46 |
| | | | | 0.4 | 2.19 | 1.93 | 2.00 | 2.70 | 2.38 | 2.48 |
| | | | | 0.5 | 2.23 | 1.91 | 2.04 | 2.77 | 2.40 | 2.52 |
| | 0.75 | 0.700 | 0.500 | 0.2 | 2.26 | 2.06 | 2.08 | 2.81 | 2.58 | 2.58 |
| | | | | 0.4 | 2.31 | 1.96 | 2.05 | 2.90 | 2.46 | 2.57 |
| | | | | 0.5 | 2.25 | 1.85 | 1.98 | 2.81 | 2.34 | 2.51 |

Table S113: Probability (%) that the lower confidence limit is greater than the true value when the alternative hypothesis is true, and only one treatment is effective, and $G = 2$.

| | | | | | Exact | | | Mid-$p$ | | |
|---|---|---|---|---|---|---|---|---|---|---|
| $N$ | $\tau$ | E[$\xi_s$] | E[$\pi_s$] | $\rho$ | MLE | CMAE | UMVCUE | MLE | CMAE | UMVCUE |
| 50 | 0.25 | 0.755 | 0.755 | 0.2 | 1.26 | 1.22 | 1.26 | 2.12 | 2.11 | 2.12 |
| | | | | 0.4 | 1.54 | 1.48 | 1.53 | 2.40 | 2.31 | 2.37 |
| | | | | 0.6 | 1.34 | 1.29 | 1.33 | 2.06 | 2.03 | 2.06 |
| | 0.50 | 0.773 | 0.773 | 0.2 | 1.45 | 1.43 | 1.45 | 2.28 | 2.28 | 2.28 |
| | | | | 0.4 | 1.51 | 1.50 | 1.50 | 2.33 | 2.30 | 2.31 |
| | | | | 0.6 | 1.46 | 1.44 | 1.45 | 2.19 | 2.17 | 2.19 |
| | 0.75 | 0.778 | 0.778 | 0.2 | 1.41 | 1.40 | 1.41 | 2.29 | 2.29 | 2.29 |
| | | | | 0.4 | 1.48 | 1.47 | 1.46 | 2.24 | 2.22 | 2.23 |
| | | | | 0.6 | 1.38 | 1.36 | 1.38 | 2.20 | 2.20 | 2.20 |
| 200 | 0.25 | 0.633 | 0.633 | 0.2 | 1.82 | 1.80 | 1.82 | 2.26 | 2.22 | 2.25 |
| | | | | 0.4 | 1.90 | 1.86 | 1.89 | 2.40 | 2.35 | 2.38 |
| | | | | 0.6 | 1.81 | 1.76 | 1.79 | 2.30 | 2.26 | 2.29 |
| | 0.50 | 0.642 | 0.642 | 0.2 | 1.91 | 1.90 | 1.90 | 2.40 | 2.38 | 2.39 |
| | | | | 0.4 | 2.06 | 2.02 | 2.02 | 2.55 | 2.52 | 2.53 |
| | | | | 0.6 | 2.06 | 2.04 | 2.06 | 2.56 | 2.53 | 2.56 |
| | 0.75 | 0.644 | 0.644 | 0.2 | 1.89 | 1.89 | 1.89 | 2.30 | 2.29 | 2.30 |
| | | | | 0.4 | 1.96 | 1.94 | 1.96 | 2.44 | 2.42 | 2.43 |
| | | | | 0.6 | 2.01 | 1.99 | 2.01 | 2.49 | 2.48 | 2.48 |

Table S114: Probability (%) that the lower confidence limit is greater than the true value when the alternative hypothesis is true, and only one treatment is effective, and $G = 3$.

| | | | | | Exact | | | Mid-$p$ | | |
|---|---|---|---|---|---|---|---|---|---|---|
| $N$ | $\tau$ | $E[\xi_s]$ | $E[\pi_s]$ | $\rho$ | MLE | CMAE | UMVCUE | MLE | CMAE | UMVCUE |
| 50 | 0.25 | 0.736 | 0.736 | 0.2 | 1.41 | 1.40 | 1.40 | 2.23 | 2.23 | 2.23 |
| | | | | 0.4 | 1.49 | 1.46 | 1.49 | 2.40 | 2.33 | 2.37 |
| | | | | 0.6 | 1.52 | 1.41 | 1.50 | 2.36 | 2.25 | 2.35 |
| | 0.50 | 0.768 | 0.768 | 0.2 | 1.48 | 1.47 | 1.48 | 2.29 | 2.25 | 2.26 |
| | | | | 0.4 | 1.47 | 1.42 | 1.45 | 2.40 | 2.34 | 2.38 |
| | | | | 0.6 | 1.55 | 1.50 | 1.53 | 2.43 | 2.38 | 2.42 |
| | 0.75 | 0.777 | 0.777 | 0.2 | 1.33 | 1.28 | 1.32 | 2.02 | 2.03 | 2.01 |
| | | | | 0.4 | 1.62 | 1.60 | 1.62 | 2.47 | 2.44 | 2.45 |
| | | | | 0.6 | 1.38 | 1.38 | 1.38 | 2.22 | 2.19 | 2.22 |
| 200 | 0.25 | 0.624 | 0.624 | 0.2 | 1.93 | 1.87 | 1.91 | 2.34 | 2.28 | 2.32 |
| | | | | 0.4 | 1.98 | 1.91 | 1.93 | 2.48 | 2.39 | 2.44 |
| | | | | 0.6 | 1.69 | 1.57 | 1.68 | 2.05 | 1.93 | 2.03 |
| | 0.50 | 0.639 | 0.639 | 0.2 | 2.03 | 2.00 | 2.00 | 2.50 | 2.47 | 2.49 |
| | | | | 0.4 | 1.87 | 1.85 | 1.86 | 2.33 | 2.29 | 2.32 |
| | | | | 0.6 | 2.00 | 1.99 | 2.00 | 2.39 | 2.39 | 2.39 |
| | 0.75 | 0.643 | 0.643 | 0.2 | 1.91 | 1.91 | 1.91 | 2.28 | 2.28 | 2.28 |
| | | | | 0.4 | 1.96 | 1.96 | 1.96 | 2.44 | 2.41 | 2.44 |
| | | | | 0.6 | 1.88 | 1.86 | 1.87 | 2.36 | 2.31 | 2.32 |

Table S115: Probability (%) that the lower confidence limit is greater than the true value when the alternative hypothesis is true, and only one treatment is effective, and $G = 4$.

| | | | | | Exact | | | Mid-$p$ | | |
|---|---|---|---|---|---|---|---|---|---|---|
| $N$ | $\tau$ | $E[\xi_s]$ | $E[\pi_s]$ | $\rho$ | MLE | CMAE | UMVCUE | MLE | CMAE | UMVCUE |
| 50 | 0.25 | 0.723 | 0.723 | 0.2 | 1.56 | 1.53 | 1.55 | 2.46 | 2.42 | 2.44 |
| | | | | 0.4 | 1.48 | 1.45 | 1.46 | 2.28 | 2.19 | 2.25 |
| | | | | 0.6 | 1.25 | 1.16 | 1.24 | 2.18 | 1.95 | 2.15 |
| | 0.50 | 0.763 | 0.763 | 0.2 | 1.38 | 1.34 | 1.36 | 2.22 | 2.16 | 2.19 |
| | | | | 0.4 | 1.80 | 1.77 | 1.79 | 2.61 | 2.56 | 2.58 |
| | | | | 0.6 | 1.47 | 1.42 | 1.44 | 2.22 | 2.14 | 2.16 |
| | 0.75 | 0.775 | 0.775 | 0.2 | 1.51 | 1.51 | 1.51 | 2.23 | 2.22 | 2.23 |
| | | | | 0.4 | 1.29 | 1.29 | 1.29 | 2.30 | 2.22 | 2.29 |
| | | | | 0.6 | 1.57 | 1.54 | 1.57 | 2.48 | 2.44 | 2.46 |
| 200 | 0.25 | 0.618 | 0.618 | 0.2 | 1.92 | 1.84 | 1.89 | 2.38 | 2.34 | 2.36 |
| | | | | 0.4 | 2.15 | 2.08 | 2.14 | 2.64 | 2.52 | 2.61 |
| | | | | 0.6 | 2.00 | 1.93 | 1.98 | 2.62 | 2.47 | 2.55 |
| | 0.50 | 0.637 | 0.637 | 0.2 | 1.89 | 1.86 | 1.88 | 2.54 | 2.50 | 2.53 |
| | | | | 0.4 | 1.80 | 1.78 | 1.79 | 2.28 | 2.21 | 2.23 |
| | | | | 0.6 | 1.78 | 1.71 | 1.76 | 2.32 | 2.22 | 2.28 |
| | 0.75 | 0.643 | 0.643 | 0.2 | 1.93 | 1.91 | 1.93 | 2.47 | 2.45 | 2.47 |
| | | | | 0.4 | 1.88 | 1.88 | 1.88 | 2.37 | 2.35 | 2.37 |
| | | | | 0.6 | 2.03 | 2.02 | 2.03 | 2.51 | 2.48 | 2.50 |

Table S116: Probability (%) that the lower confidence limit is greater than the true value when the alternative hypothesis is true, and relationship between the treatment group and binomial probability is linear, and $G = 2$.

| | | | | | Exact | | | Mid-$p$ | | |
|---|---|---|---|---|---|---|---|---|---|---|
| $N$ | $\tau$ | $\mathrm{E}[\xi_s]$ | $\mathrm{E}[\pi_s]$ | $\rho$ | MLE | CMAE | UMVCUE | MLE | CMAE | UMVCUE |
| 50 | 0.25 | 0.741 | 0.741 | 0.2 | 1.83 | 1.77 | 1.79 | 2.60 | 2.55 | 2.59 |
| | | | | 0.4 | 1.70 | 1.62 | 1.69 | 2.43 | 2.37 | 2.43 |
| | | | | 0.6 | 1.65 | 1.57 | 1.62 | 2.67 | 2.54 | 2.64 |
| | 0.50 | 0.756 | 0.756 | 0.2 | 1.60 | 1.49 | 1.59 | 2.54 | 2.48 | 2.50 |
| | | | | 0.4 | 1.57 | 1.46 | 1.49 | 2.41 | 2.32 | 2.38 |
| | | | | 0.6 | 1.67 | 1.62 | 1.65 | 2.68 | 2.48 | 2.65 |
| | 0.75 | 0.765 | 0.765 | 0.2 | 1.55 | 1.54 | 1.54 | 2.33 | 2.22 | 2.28 |
| | | | | 0.4 | 1.65 | 1.56 | 1.62 | 2.54 | 2.44 | 2.50 |
| | | | | 0.6 | 1.66 | 1.58 | 1.65 | 2.58 | 2.49 | 2.56 |
| 200 | 0.25 | 0.626 | 0.626 | 0.2 | 2.06 | 2.05 | 2.05 | 2.58 | 2.53 | 2.57 |
| | | | | 0.4 | 1.98 | 1.88 | 1.95 | 2.42 | 2.36 | 2.42 |
| | | | | 0.6 | 2.04 | 1.94 | 2.04 | 2.63 | 2.50 | 2.61 |
| | 0.50 | 0.633 | 0.633 | 0.2 | 2.01 | 1.97 | 2.00 | 2.50 | 2.45 | 2.49 |
| | | | | 0.4 | 1.84 | 1.77 | 1.83 | 2.41 | 2.32 | 2.39 |
| | | | | 0.6 | 2.36 | 2.17 | 2.31 | 2.96 | 2.76 | 2.88 |
| | 0.75 | 0.637 | 0.637 | 0.2 | 1.96 | 1.89 | 1.92 | 2.35 | 2.34 | 2.34 |
| | | | | 0.4 | 2.15 | 2.07 | 2.10 | 2.63 | 2.53 | 2.54 |
| | | | | 0.6 | 1.93 | 1.84 | 1.90 | 2.46 | 2.36 | 2.40 |

Table S117: Probability (%) that the lower confidence limit is greater than the true value when the alternative hypothesis is true, and relationship between the treatment group and binomial probability is linear, and $G = 3$.

| | | | | | Exact | | | Mid-$p$ | | |
|---|---|---|---|---|---|---|---|---|---|---|
| $N$ | $\tau$ | $\mathrm{E}[\xi_s]$ | $\mathrm{E}[\pi_s]$ | $\rho$ | MLE | CMAE | UMVCUE | MLE | CMAE | UMVCUE |
| 50 | 0.25 | 0.725 | 0.725 | 0.2 | 1.41 | 1.37 | 1.40 | 2.16 | 2.00 | 2.09 |
| | | | | 0.4 | 1.70 | 1.62 | 1.65 | 2.46 | 2.33 | 2.42 |
| | | | | 0.6 | 1.49 | 1.36 | 1.46 | 2.56 | 2.37 | 2.51 |
| | 0.50 | 0.744 | 0.744 | 0.2 | 1.50 | 1.45 | 1.49 | 2.48 | 2.36 | 2.43 |
| | | | | 0.4 | 1.48 | 1.35 | 1.45 | 2.53 | 2.30 | 2.41 |
| | | | | 0.6 | 1.58 | 1.45 | 1.53 | 2.46 | 2.29 | 2.36 |
| | 0.75 | 0.755 | 0.755 | 0.2 | 1.57 | 1.51 | 1.56 | 2.47 | 2.33 | 2.40 |
| | | | | 0.4 | 1.54 | 1.39 | 1.44 | 2.35 | 2.13 | 2.19 |
| | | | | 0.6 | 1.47 | 1.36 | 1.45 | 2.32 | 2.18 | 2.28 |
| 200 | 0.25 | 0.619 | 0.619 | 0.2 | 2.00 | 1.94 | 1.98 | 2.57 | 2.47 | 2.52 |
| | | | | 0.4 | 2.12 | 2.00 | 2.10 | 2.67 | 2.55 | 2.60 |
| | | | | 0.6 | 2.10 | 1.96 | 2.06 | 2.71 | 2.45 | 2.69 |
| | 0.50 | 0.628 | 0.628 | 0.2 | 2.38 | 2.30 | 2.32 | 2.85 | 2.73 | 2.78 |
| | | | | 0.4 | 1.96 | 1.84 | 1.90 | 2.46 | 2.25 | 2.32 |
| | | | | 0.6 | 2.04 | 1.88 | 1.98 | 2.62 | 2.32 | 2.53 |
| | 0.75 | 0.632 | 0.632 | 0.2 | 1.98 | 1.87 | 1.92 | 2.37 | 2.30 | 2.30 |
| | | | | 0.4 | 2.13 | 1.98 | 2.03 | 2.52 | 2.39 | 2.42 |
| | | | | 0.6 | 2.28 | 2.15 | 2.19 | 2.83 | 2.58 | 2.71 |

Table S118: Probability (%) that the lower confidence limit is greater than the true value when the alternative hypothesis is true, and relationship between the treatment group and binomial probability is linear, and $G = 4$.

| | | | | | Exact | | | Mid-$p$ | | |
|---|---|---|---|---|---|---|---|---|---|---|
| $N$ | $\tau$ | E[$\xi_s$] | E[$\pi_s$] | $\rho$ | MLE | CMAE | UMVCUE | MLE | CMAE | UMVCUE |
| 50 | 0.25 | 0.717 | 0.717 | 0.2 | 1.52 | 1.45 | 1.49 | 2.38 | 2.18 | 2.28 |
| | | | | 0.4 | 1.61 | 1.53 | 1.57 | 2.42 | 2.23 | 2.35 |
| | | | | 0.6 | 1.49 | 1.39 | 1.47 | 2.41 | 2.15 | 2.32 |
| | 0.50 | 0.738 | 0.738 | 0.2 | 1.71 | 1.57 | 1.61 | 2.50 | 2.47 | 2.49 |
| | | | | 0.4 | 1.28 | 1.16 | 1.22 | 2.15 | 1.94 | 2.05 |
| | | | | 0.6 | 1.63 | 1.49 | 1.57 | 2.42 | 2.20 | 2.36 |
| | 0.75 | 0.750 | 0.750 | 0.2 | 1.44 | 1.40 | 1.36 | 2.21 | 2.13 | 2.12 |
| | | | | 0.4 | 1.60 | 1.49 | 1.54 | 2.35 | 2.10 | 2.25 |
| | | | | 0.6 | 1.60 | 1.47 | 1.51 | 2.59 | 2.29 | 2.43 |
| 200 | 0.25 | 0.616 | 0.616 | 0.2 | 1.91 | 1.80 | 1.87 | 2.43 | 2.34 | 2.41 |
| | | | | 0.4 | 2.06 | 1.95 | 2.02 | 2.46 | 2.34 | 2.42 |
| | | | | 0.6 | 2.10 | 1.93 | 2.06 | 2.58 | 2.42 | 2.55 |
| | 0.50 | 0.625 | 0.625 | 0.2 | 1.85 | 1.80 | 1.81 | 2.39 | 2.25 | 2.29 |
| | | | | 0.4 | 1.90 | 1.68 | 1.76 | 2.37 | 2.21 | 2.30 |
| | | | | 0.6 | 2.09 | 1.85 | 1.95 | 2.79 | 2.38 | 2.54 |
| | 0.75 | 0.630 | 0.630 | 0.2 | 1.86 | 1.76 | 1.77 | 2.42 | 2.24 | 2.32 |
| | | | | 0.4 | 2.13 | 2.02 | 2.03 | 2.65 | 2.44 | 2.45 |
| | | | | 0.6 | 2.06 | 1.78 | 1.90 | 2.70 | 2.37 | 2.53 |

Table S119: Probability (%) that the lower confidence limit is greater than the true value when the alternative hypothesis is true, and all treatments are effective, and $G = 2$.

| | | | | | Exact | | | Mid-$p$ | | |
|---|---|---|---|---|---|---|---|---|---|---|
| $N$ | $\tau$ | E[$\xi_s$] | E[$\pi_s$] | $\rho$ | MLE | CMAE | UMVCUE | MLE | CMAE | UMVCUE |
| 50 | 0.25 | 0.780 | 0.780 | 0.2 | 1.54 | 1.54 | 1.54 | 2.30 | 2.21 | 2.30 |
| | | | | 0.4 | 1.56 | 1.40 | 1.48 | 2.61 | 2.44 | 2.60 |
| | | | | 0.6 | 1.43 | 1.35 | 1.41 | 2.26 | 2.14 | 2.25 |
| | 0.50 | 0.780 | 0.780 | 0.2 | 1.65 | 1.60 | 1.63 | 2.52 | 2.40 | 2.47 |
| | | | | 0.4 | 1.45 | 1.36 | 1.40 | 2.42 | 2.27 | 2.38 |
| | | | | 0.6 | 1.64 | 1.44 | 1.62 | 2.68 | 2.47 | 2.66 |
| | 0.75 | 0.780 | 0.780 | 0.2 | 1.60 | 1.51 | 1.55 | 2.53 | 2.42 | 2.47 |
| | | | | 0.4 | 1.73 | 1.57 | 1.67 | 2.50 | 2.32 | 2.41 |
| | | | | 0.6 | 1.45 | 1.29 | 1.39 | 2.31 | 2.15 | 2.28 |
| 200 | 0.25 | 0.645 | 0.645 | 0.2 | 2.04 | 1.96 | 2.01 | 2.65 | 2.53 | 2.60 |
| | | | | 0.4 | 2.29 | 2.24 | 2.27 | 2.97 | 2.81 | 2.92 |
| | | | | 0.6 | 2.03 | 1.93 | 1.99 | 2.60 | 2.42 | 2.58 |
| | 0.50 | 0.645 | 0.645 | 0.2 | 2.17 | 2.08 | 2.10 | 2.53 | 2.48 | 2.51 |
| | | | | 0.4 | 2.10 | 1.97 | 2.02 | 2.55 | 2.42 | 2.53 |
| | | | | 0.6 | 2.00 | 1.91 | 2.00 | 2.54 | 2.32 | 2.43 |
| | 0.75 | 0.645 | 0.645 | 0.2 | 2.10 | 1.98 | 1.99 | 2.57 | 2.50 | 2.49 |
| | | | | 0.4 | 2.36 | 2.18 | 2.23 | 2.95 | 2.74 | 2.82 |
| | | | | 0.6 | 2.16 | 2.00 | 2.11 | 2.69 | 2.37 | 2.53 |

Table S120: Probability (%) that the lower confidence limit is greater than the true value when the alternative hypothesis is true, and all treatments are effective, and $G = 3$.

| | | | | | Exact | | | Mid-$p$ | | |
|---|---|---|---|---|---|---|---|---|---|---|
| $N$ | $\tau$ | $E[\xi_s]$ | $E[\pi_s]$ | $\rho$ | MLE | CMAE | UMVCUE | MLE | CMAE | UMVCUE |
| 50 | 0.25 | 0.780 | 0.780 | 0.2 | 1.46 | 1.42 | 1.42 | 2.34 | 2.27 | 2.30 |
| | | | | 0.4 | 1.43 | 1.38 | 1.41 | 2.48 | 2.30 | 2.40 |
| | | | | 0.6 | 1.48 | 1.33 | 1.43 | 2.43 | 2.28 | 2.39 |
| | 0.50 | 0.780 | 0.780 | 0.2 | 1.70 | 1.64 | 1.68 | 2.63 | 2.45 | 2.56 |
| | | | | 0.4 | 1.82 | 1.68 | 1.75 | 2.81 | 2.57 | 2.62 |
| | | | | 0.6 | 1.51 | 1.31 | 1.40 | 2.42 | 2.02 | 2.23 |
| | 0.75 | 0.780 | 0.780 | 0.2 | 1.59 | 1.56 | 1.56 | 2.45 | 2.24 | 2.28 |
| | | | | 0.4 | 1.51 | 1.25 | 1.31 | 2.58 | 2.38 | 2.40 |
| | | | | 0.6 | 1.45 | 1.29 | 1.40 | 2.31 | 2.00 | 2.21 |
| 200 | 0.25 | 0.645 | 0.645 | 0.2 | 2.14 | 2.10 | 2.12 | 2.82 | 2.69 | 2.77 |
| | | | | 0.4 | 2.17 | 2.06 | 2.15 | 2.72 | 2.57 | 2.65 |
| | | | | 0.6 | 2.08 | 1.93 | 2.04 | 2.54 | 2.39 | 2.48 |
| | 0.50 | 0.645 | 0.645 | 0.2 | 2.20 | 2.04 | 2.10 | 2.60 | 2.46 | 2.50 |
| | | | | 0.4 | 2.10 | 1.87 | 2.00 | 2.55 | 2.38 | 2.42 |
| | | | | 0.6 | 2.38 | 2.16 | 2.31 | 2.88 | 2.60 | 2.68 |
| | 0.75 | 0.645 | 0.645 | 0.2 | 2.03 | 1.89 | 1.86 | 2.40 | 2.28 | 2.30 |
| | | | | 0.4 | 2.54 | 2.31 | 2.34 | 3.05 | 2.75 | 2.88 |
| | | | | 0.6 | 2.24 | 1.98 | 2.10 | 2.83 | 2.47 | 2.65 |

Table S121: Probability (%) that the lower confidence limit is greater than the true value when the alternative hypothesis is true, and all treatments are effective, and $G = 4$.

| | | | | | Exact | | | Mid-$p$ | | |
|---|---|---|---|---|---|---|---|---|---|---|
| $N$ | $\tau$ | $E[\xi_s]$ | $E[\pi_s]$ | $\rho$ | MLE | CMAE | UMVCUE | MLE | CMAE | UMVCUE |
| 50 | 0.25 | 0.780 | 0.780 | 0.2 | 1.70 | 1.68 | 1.68 | 2.49 | 2.45 | 2.45 |
| | | | | 0.4 | 1.52 | 1.46 | 1.49 | 2.59 | 2.52 | 2.55 |
| | | | | 0.6 | 1.50 | 1.43 | 1.44 | 2.21 | 2.03 | 2.13 |
| | 0.50 | 0.780 | 0.780 | 0.2 | 1.46 | 1.40 | 1.42 | 2.30 | 2.14 | 2.16 |
| | | | | 0.4 | 1.64 | 1.43 | 1.54 | 2.49 | 2.27 | 2.39 |
| | | | | 0.6 | 1.68 | 1.45 | 1.60 | 2.71 | 2.30 | 2.50 |
| | 0.75 | 0.780 | 0.780 | 0.2 | 1.92 | 1.83 | 1.84 | 2.95 | 2.75 | 2.80 |
| | | | | 0.4 | 1.61 | 1.42 | 1.49 | 2.59 | 2.27 | 2.41 |
| | | | | 0.6 | 1.77 | 1.47 | 1.63 | 2.81 | 2.33 | 2.62 |
| 200 | 0.25 | 0.645 | 0.645 | 0.2 | 1.94 | 1.85 | 1.90 | 2.45 | 2.37 | 2.43 |
| | | | | 0.4 | 2.01 | 1.87 | 1.97 | 2.69 | 2.47 | 2.63 |
| | | | | 0.6 | 1.86 | 1.71 | 1.79 | 2.47 | 2.14 | 2.37 |
| | 0.50 | 0.645 | 0.645 | 0.2 | 1.87 | 1.78 | 1.81 | 2.51 | 2.33 | 2.39 |
| | | | | 0.4 | 2.02 | 1.81 | 1.88 | 2.63 | 2.32 | 2.48 |
| | | | | 0.6 | 2.27 | 1.94 | 2.07 | 2.88 | 2.37 | 2.64 |
| | 0.75 | 0.645 | 0.645 | 0.2 | 1.95 | 1.82 | 1.75 | 2.61 | 2.32 | 2.35 |
| | | | | 0.4 | 2.10 | 1.81 | 1.85 | 2.66 | 2.24 | 2.39 |
| | | | | 0.6 | 2.35 | 1.93 | 2.06 | 2.93 | 2.44 | 2.59 |

Table S122: Mean of $\hat{\rho}$ when the null hypothesis is true and $\xi_g = \pi_g = 0.1$.

| | | | $\rho = 0.2$ | | | $\rho = 0.4$ | | | $\rho = 0.6$ | | |
|---|---|---|---|---|---|---|---|---|---|---|---|
| $N$ | $\tau$ | $G$ | MLE | CMAE | UMVCUE | MLE | CMAE | UMVCUE | MLE | CMAE | UMVCUE |
| 50 | 0.25 | 2 | 0.20 | 0.20 | 0.19 | 0.40 | 0.40 | 0.39 | 0.60 | 0.59 | 0.59 |
| | | 3 | 0.21 | 0.20 | 0.19 | 0.41 | 0.40 | 0.39 | 0.61 | 0.60 | 0.59 |
| | | 4 | 0.21 | 0.20 | 0.20 | 0.41 | 0.40 | 0.39 | 0.61 | 0.60 | 0.59 |
| | 0.50 | 2 | 0.21 | 0.20 | 0.19 | 0.41 | 0.40 | 0.39 | 0.61 | 0.60 | 0.58 |
| | | 3 | 0.21 | 0.20 | 0.19 | 0.42 | 0.40 | 0.39 | 0.62 | 0.60 | 0.58 |
| | | 4 | 0.21 | 0.20 | 0.19 | 0.42 | 0.41 | 0.39 | 0.62 | 0.60 | 0.58 |
| | 0.75 | 2 | 0.21 | 0.20 | 0.19 | 0.41 | 0.40 | 0.38 | 0.61 | 0.60 | 0.57 |
| | | 3 | 0.22 | 0.21 | 0.19 | 0.42 | 0.41 | 0.37 | 0.62 | 0.61 | 0.56 |
| | | 4 | 0.22 | 0.21 | 0.19 | 0.43 | 0.41 | 0.37 | 0.63 | 0.61 | 0.56 |
| 200 | 0.25 | 2 | 0.20 | 0.20 | 0.20 | 0.40 | 0.40 | 0.40 | 0.60 | 0.60 | 0.60 |
| | | 3 | 0.21 | 0.20 | 0.20 | 0.41 | 0.40 | 0.40 | 0.61 | 0.60 | 0.60 |
| | | 4 | 0.21 | 0.20 | 0.20 | 0.41 | 0.40 | 0.40 | 0.61 | 0.60 | 0.60 |
| | 0.50 | 2 | 0.20 | 0.20 | 0.20 | 0.41 | 0.40 | 0.40 | 0.61 | 0.60 | 0.60 |
| | | 3 | 0.21 | 0.20 | 0.20 | 0.41 | 0.40 | 0.40 | 0.61 | 0.60 | 0.60 |
| | | 4 | 0.21 | 0.20 | 0.20 | 0.41 | 0.40 | 0.40 | 0.61 | 0.60 | 0.60 |
| | 0.75 | 2 | 0.21 | 0.20 | 0.20 | 0.41 | 0.40 | 0.40 | 0.61 | 0.60 | 0.59 |
| | | 3 | 0.21 | 0.20 | 0.20 | 0.41 | 0.41 | 0.39 | 0.61 | 0.61 | 0.59 |
| | | 4 | 0.21 | 0.20 | 0.20 | 0.42 | 0.41 | 0.39 | 0.62 | 0.61 | 0.59 |

Table S123: Mean of $\hat{\rho}$ when the null hypothesis is true and $\xi_g = \pi_g = 0.5$.

| | | | $\rho = 0.2$ | | | $\rho = 0.4$ | | | $\rho = 0.6$ | | |
|---|---|---|---|---|---|---|---|---|---|---|---|
| $N$ | $\tau$ | $G$ | MLE | CMAE | UMVCUE | MLE | CMAE | UMVCUE | MLE | CMAE | UMVCUE |
| 50 | 0.25 | 2 | 0.20 | 0.20 | 0.20 | 0.40 | 0.40 | 0.40 | 0.60 | 0.60 | 0.60 |
| | | 3 | 0.20 | 0.20 | 0.20 | 0.40 | 0.40 | 0.40 | 0.60 | 0.60 | 0.60 |
| | | 4 | 0.20 | 0.20 | 0.20 | 0.40 | 0.40 | 0.40 | 0.60 | 0.60 | 0.60 |
| | 0.50 | 2 | 0.20 | 0.20 | 0.20 | 0.40 | 0.40 | 0.40 | 0.60 | 0.60 | 0.60 |
| | | 3 | 0.20 | 0.20 | 0.20 | 0.40 | 0.40 | 0.40 | 0.60 | 0.60 | 0.60 |
| | | 4 | 0.20 | 0.20 | 0.20 | 0.40 | 0.40 | 0.40 | 0.60 | 0.60 | 0.60 |
| | 0.75 | 2 | 0.20 | 0.20 | 0.20 | 0.40 | 0.40 | 0.40 | 0.60 | 0.60 | 0.60 |
| | | 3 | 0.20 | 0.20 | 0.20 | 0.40 | 0.40 | 0.40 | 0.60 | 0.60 | 0.60 |
| | | 4 | 0.20 | 0.20 | 0.20 | 0.40 | 0.40 | 0.40 | 0.60 | 0.60 | 0.59 |
| 200 | 0.25 | 2 | 0.20 | 0.20 | 0.20 | 0.40 | 0.40 | 0.40 | 0.60 | 0.60 | 0.60 |
| | | 3 | 0.20 | 0.20 | 0.20 | 0.40 | 0.40 | 0.40 | 0.60 | 0.60 | 0.60 |
| | | 4 | 0.20 | 0.20 | 0.20 | 0.40 | 0.40 | 0.40 | 0.60 | 0.60 | 0.60 |
| | 0.50 | 2 | 0.20 | 0.20 | 0.20 | 0.40 | 0.40 | 0.40 | 0.60 | 0.60 | 0.60 |
| | | 3 | 0.20 | 0.20 | 0.20 | 0.40 | 0.40 | 0.40 | 0.60 | 0.60 | 0.60 |
| | | 4 | 0.20 | 0.20 | 0.20 | 0.40 | 0.40 | 0.40 | 0.60 | 0.60 | 0.60 |
| | 0.75 | 2 | 0.20 | 0.20 | 0.20 | 0.40 | 0.40 | 0.40 | 0.60 | 0.60 | 0.60 |
| | | 3 | 0.20 | 0.20 | 0.20 | 0.40 | 0.40 | 0.40 | 0.60 | 0.60 | 0.60 |
| | | 4 | 0.20 | 0.20 | 0.20 | 0.40 | 0.40 | 0.40 | 0.60 | 0.60 | 0.60 |

Table S124: Mean of $\hat{\rho}$ when the null hypothesis is true and $\xi_g = \pi_g = 0.7$.

| | | | $\rho = 0.2$ | | | $\rho = 0.4$ | | | $\rho = 0.6$ | | |
|---|---|---|---|---|---|---|---|---|---|---|---|
| $N$ | $\tau$ | $G$ | MLE | CMAE | UMVCUE | MLE | CMAE | UMVCUE | MLE | CMAE | UMVCUE |
| 50 | 0.25 | 2 | 0.20 | 0.20 | 0.20 | 0.39 | 0.40 | 0.40 | 0.60 | 0.60 | 0.60 |
| | | 3 | 0.19 | 0.20 | 0.20 | 0.39 | 0.40 | 0.40 | 0.59 | 0.60 | 0.60 |
| | | 4 | 0.19 | 0.20 | 0.20 | 0.39 | 0.40 | 0.40 | 0.59 | 0.60 | 0.60 |
| | 0.50 | 2 | 0.19 | 0.20 | 0.20 | 0.39 | 0.40 | 0.40 | 0.59 | 0.60 | 0.60 |
| | | 3 | 0.19 | 0.20 | 0.20 | 0.39 | 0.39 | 0.40 | 0.59 | 0.60 | 0.60 |
| | | 4 | 0.19 | 0.20 | 0.20 | 0.39 | 0.39 | 0.40 | 0.59 | 0.59 | 0.60 |
| | 0.75 | 2 | 0.19 | 0.20 | 0.20 | 0.39 | 0.40 | 0.40 | 0.59 | 0.60 | 0.59 |
| | | 3 | 0.19 | 0.20 | 0.20 | 0.39 | 0.39 | 0.39 | 0.59 | 0.59 | 0.59 |
| | | 4 | 0.19 | 0.19 | 0.20 | 0.38 | 0.39 | 0.39 | 0.58 | 0.59 | 0.59 |
| 200 | 0.25 | 2 | 0.20 | 0.20 | 0.20 | 0.40 | 0.40 | 0.40 | 0.60 | 0.60 | 0.60 |
| | | 3 | 0.20 | 0.20 | 0.20 | 0.40 | 0.40 | 0.40 | 0.60 | 0.60 | 0.60 |
| | | 4 | 0.20 | 0.20 | 0.20 | 0.40 | 0.40 | 0.40 | 0.60 | 0.60 | 0.60 |
| | 0.50 | 2 | 0.20 | 0.20 | 0.20 | 0.40 | 0.40 | 0.40 | 0.60 | 0.60 | 0.60 |
| | | 3 | 0.20 | 0.20 | 0.20 | 0.39 | 0.40 | 0.40 | 0.60 | 0.60 | 0.60 |
| | | 4 | 0.20 | 0.20 | 0.20 | 0.39 | 0.40 | 0.40 | 0.59 | 0.60 | 0.60 |
| | 0.75 | 2 | 0.20 | 0.20 | 0.20 | 0.40 | 0.40 | 0.40 | 0.60 | 0.60 | 0.60 |
| | | 3 | 0.20 | 0.20 | 0.20 | 0.39 | 0.40 | 0.40 | 0.59 | 0.60 | 0.60 |
| | | 4 | 0.20 | 0.20 | 0.20 | 0.39 | 0.40 | 0.40 | 0.59 | 0.60 | 0.60 |

Table S125: Mean of $\hat{\rho}$ when the null hypothesis is true, $\xi_g$ =0.3, and $\pi_g = 0.5$.

| | | | $\rho = 0.2$ | | | $\rho = 0.4$ | | | $\rho = 0.5$ | | |
|---|---|---|---|---|---|---|---|---|---|---|---|
| $N$ | $\tau$ | $G$ | MLE | CMAE | UMVCUE | MLE | CMAE | UMVCUE | MLE | CMAE | UMVCUE |
| 50 | 0.25 | 2 | 0.20 | 0.20 | 0.20 | 0.41 | 0.40 | 0.40 | 0.51 | 0.50 | 0.50 |
| | | 3 | 0.21 | 0.20 | 0.20 | 0.41 | 0.40 | 0.40 | 0.51 | 0.50 | 0.50 |
| | | 4 | 0.21 | 0.20 | 0.20 | 0.41 | 0.40 | 0.40 | 0.51 | 0.50 | 0.50 |
| | 0.50 | 2 | 0.20 | 0.20 | 0.20 | 0.41 | 0.40 | 0.40 | 0.51 | 0.50 | 0.50 |
| | | 3 | 0.21 | 0.20 | 0.20 | 0.41 | 0.40 | 0.40 | 0.52 | 0.51 | 0.50 |
| | | 4 | 0.21 | 0.20 | 0.20 | 0.42 | 0.40 | 0.40 | 0.52 | 0.51 | 0.50 |
| | 0.75 | 2 | 0.21 | 0.20 | 0.20 | 0.41 | 0.41 | 0.40 | 0.51 | 0.51 | 0.49 |
| | | 3 | 0.21 | 0.20 | 0.20 | 0.42 | 0.41 | 0.39 | 0.52 | 0.51 | 0.49 |
| | | 4 | 0.21 | 0.20 | 0.20 | 0.42 | 0.41 | 0.39 | 0.52 | 0.51 | 0.49 |
| 200 | 0.25 | 2 | 0.20 | 0.20 | 0.20 | 0.40 | 0.40 | 0.40 | 0.50 | 0.50 | 0.50 |
| | | 3 | 0.20 | 0.20 | 0.20 | 0.41 | 0.40 | 0.40 | 0.51 | 0.50 | 0.50 |
| | | 4 | 0.20 | 0.20 | 0.20 | 0.41 | 0.40 | 0.40 | 0.51 | 0.50 | 0.50 |
| | 0.50 | 2 | 0.20 | 0.20 | 0.20 | 0.40 | 0.40 | 0.40 | 0.51 | 0.50 | 0.50 |
| | | 3 | 0.20 | 0.20 | 0.20 | 0.41 | 0.40 | 0.40 | 0.51 | 0.50 | 0.50 |
| | | 4 | 0.20 | 0.20 | 0.20 | 0.41 | 0.40 | 0.40 | 0.51 | 0.50 | 0.50 |
| | 0.75 | 2 | 0.20 | 0.20 | 0.20 | 0.41 | 0.40 | 0.40 | 0.51 | 0.50 | 0.50 |
| | | 3 | 0.20 | 0.20 | 0.20 | 0.41 | 0.40 | 0.40 | 0.51 | 0.50 | 0.50 |
| | | 4 | 0.21 | 0.20 | 0.20 | 0.41 | 0.40 | 0.40 | 0.51 | 0.51 | 0.50 |

Table S126: Mean of $\hat{\rho}$ when the null hypothesis is true, $\xi_g$ =0.7, and $\pi_g = 0.5$.

| | | | $\rho = 0.2$ | | | $\rho = 0.4$ | | | $\rho = 0.5$ | | |
|---|---|---|---|---|---|---|---|---|---|---|---|
| $N$ | $\tau$ | $G$ | MLE | CMAE | UMVCUE | MLE | CMAE | UMVCUE | MLE | CMAE | UMVCUE |
| 50 | 0.25 | 2 | 0.20 | 0.20 | 0.20 | 0.39 | 0.40 | 0.40 | 0.49 | 0.50 | 0.50 |
| | | 3 | 0.19 | 0.20 | 0.20 | 0.39 | 0.40 | 0.40 | 0.49 | 0.50 | 0.50 |
| | | 4 | 0.19 | 0.20 | 0.20 | 0.39 | 0.40 | 0.40 | 0.48 | 0.50 | 0.50 |
| | 0.50 | 2 | 0.19 | 0.20 | 0.20 | 0.39 | 0.39 | 0.40 | 0.49 | 0.49 | 0.50 |
| | | 3 | 0.19 | 0.20 | 0.20 | 0.38 | 0.39 | 0.40 | 0.48 | 0.49 | 0.50 |
| | | 4 | 0.19 | 0.20 | 0.20 | 0.38 | 0.39 | 0.40 | 0.47 | 0.49 | 0.50 |
| | 0.75 | 2 | 0.19 | 0.20 | 0.20 | 0.39 | 0.39 | 0.40 | 0.48 | 0.49 | 0.50 |
| | | 3 | 0.19 | 0.20 | 0.20 | 0.38 | 0.39 | 0.39 | 0.48 | 0.49 | 0.49 |
| | | 4 | 0.19 | 0.19 | 0.20 | 0.38 | 0.39 | 0.39 | 0.47 | 0.49 | 0.49 |
| 200 | 0.25 | 2 | 0.20 | 0.20 | 0.20 | 0.40 | 0.40 | 0.40 | 0.50 | 0.50 | 0.50 |
| | | 3 | 0.20 | 0.20 | 0.20 | 0.39 | 0.40 | 0.40 | 0.49 | 0.50 | 0.50 |
| | | 4 | 0.20 | 0.20 | 0.20 | 0.39 | 0.40 | 0.40 | 0.49 | 0.50 | 0.50 |
| | 0.50 | 2 | 0.20 | 0.20 | 0.20 | 0.39 | 0.40 | 0.40 | 0.49 | 0.50 | 0.50 |
| | | 3 | 0.20 | 0.20 | 0.20 | 0.39 | 0.40 | 0.40 | 0.49 | 0.50 | 0.50 |
| | | 4 | 0.20 | 0.20 | 0.20 | 0.39 | 0.40 | 0.40 | 0.49 | 0.50 | 0.50 |
| | 0.75 | 2 | 0.20 | 0.20 | 0.20 | 0.39 | 0.40 | 0.40 | 0.49 | 0.50 | 0.50 |
| | | 3 | 0.20 | 0.20 | 0.20 | 0.39 | 0.40 | 0.40 | 0.49 | 0.50 | 0.50 |
| | | 4 | 0.19 | 0.20 | 0.20 | 0.39 | 0.40 | 0.40 | 0.49 | 0.49 | 0.50 |

Table S127: Mean of $\hat{\rho}$ when the alternative hypothesis is true, and only one treatment is effective.

| | | | $\rho = 0.2$ | | | $\rho = 0.4$ | | | $\rho = 0.6$ | | |
|---|---|---|---|---|---|---|---|---|---|---|---|
| $N$ | $\tau$ | $G$ | MLE | CMAE | UMVCUE | MLE | CMAE | UMVCUE | MLE | CMAE | UMVCUE |
| 50 | 0.25 | 2 | 0.19 | 0.20 | 0.20 | 0.40 | 0.40 | 0.40 | 0.60 | 0.60 | 0.60 |
| | | 3 | 0.20 | 0.20 | 0.20 | 0.40 | 0.40 | 0.40 | 0.60 | 0.60 | 0.60 |
| | | 4 | 0.20 | 0.20 | 0.20 | 0.40 | 0.40 | 0.40 | 0.60 | 0.60 | 0.60 |
| | 0.50 | 2 | 0.20 | 0.20 | 0.20 | 0.40 | 0.40 | 0.40 | 0.60 | 0.60 | 0.60 |
| | | 3 | 0.20 | 0.20 | 0.20 | 0.40 | 0.40 | 0.40 | 0.60 | 0.60 | 0.60 |
| | | 4 | 0.20 | 0.20 | 0.20 | 0.39 | 0.40 | 0.40 | 0.60 | 0.60 | 0.60 |
| | 0.75 | 2 | 0.20 | 0.20 | 0.20 | 0.40 | 0.40 | 0.40 | 0.60 | 0.60 | 0.60 |
| | | 3 | 0.20 | 0.20 | 0.20 | 0.40 | 0.40 | 0.40 | 0.60 | 0.60 | 0.60 |
| | | 4 | 0.20 | 0.20 | 0.20 | 0.40 | 0.40 | 0.40 | 0.60 | 0.60 | 0.60 |
| 200 | 0.25 | 2 | 0.20 | 0.20 | 0.20 | 0.40 | 0.40 | 0.40 | 0.60 | 0.60 | 0.60 |
| | | 3 | 0.20 | 0.20 | 0.20 | 0.40 | 0.40 | 0.40 | 0.60 | 0.60 | 0.60 |
| | | 4 | 0.20 | 0.20 | 0.20 | 0.40 | 0.40 | 0.40 | 0.60 | 0.60 | 0.60 |
| | 0.50 | 2 | 0.20 | 0.20 | 0.20 | 0.40 | 0.40 | 0.40 | 0.60 | 0.60 | 0.60 |
| | | 3 | 0.20 | 0.20 | 0.20 | 0.40 | 0.40 | 0.40 | 0.60 | 0.60 | 0.60 |
| | | 4 | 0.20 | 0.20 | 0.20 | 0.40 | 0.40 | 0.40 | 0.60 | 0.60 | 0.60 |
| | 0.75 | 2 | 0.20 | 0.20 | 0.20 | 0.40 | 0.40 | 0.40 | 0.60 | 0.60 | 0.60 |
| | | 3 | 0.20 | 0.20 | 0.20 | 0.40 | 0.40 | 0.40 | 0.60 | 0.60 | 0.60 |
| | | 4 | 0.20 | 0.20 | 0.20 | 0.40 | 0.40 | 0.40 | 0.60 | 0.60 | 0.60 |

Table S128: Mean of $\hat{\rho}$ when the alternative hypothesis is true, and relationship between the treatment group and binomial probability is linear.

| | | | $\rho = 0.2$ | | | $\rho = 0.4$ | | | $\rho = 0.6$ | | |
|---|---|---|---|---|---|---|---|---|---|---|---|
| $N$ | $\tau$ | $G$ | MLE | CMAE | UMVCUE | MLE | CMAE | UMVCUE | MLE | CMAE | UMVCUE |
| 50 | 0.25 | 2 | 0.20 | 0.20 | 0.20 | 0.39 | 0.40 | 0.40 | 0.60 | 0.60 | 0.60 |
| | | 3 | 0.19 | 0.20 | 0.20 | 0.39 | 0.39 | 0.40 | 0.59 | 0.60 | 0.60 |
| | | 4 | 0.19 | 0.20 | 0.20 | 0.39 | 0.39 | 0.40 | 0.59 | 0.60 | 0.60 |
| | 0.50 | 2 | 0.20 | 0.20 | 0.20 | 0.39 | 0.40 | 0.40 | 0.59 | 0.59 | 0.59 |
| | | 3 | 0.19 | 0.20 | 0.20 | 0.39 | 0.40 | 0.40 | 0.59 | 0.60 | 0.60 |
| | | 4 | 0.19 | 0.20 | 0.20 | 0.39 | 0.40 | 0.40 | 0.59 | 0.60 | 0.60 |
| | 0.75 | 2 | 0.20 | 0.20 | 0.20 | 0.40 | 0.40 | 0.40 | 0.59 | 0.60 | 0.60 |
| | | 3 | 0.19 | 0.20 | 0.20 | 0.39 | 0.40 | 0.39 | 0.59 | 0.59 | 0.59 |
| | | 4 | 0.19 | 0.19 | 0.19 | 0.38 | 0.39 | 0.39 | 0.59 | 0.59 | 0.59 |
| 200 | 0.25 | 2 | 0.20 | 0.20 | 0.20 | 0.40 | 0.40 | 0.40 | 0.60 | 0.60 | 0.60 |
| | | 3 | 0.20 | 0.20 | 0.20 | 0.40 | 0.40 | 0.40 | 0.60 | 0.60 | 0.60 |
| | | 4 | 0.20 | 0.20 | 0.20 | 0.40 | 0.40 | 0.40 | 0.60 | 0.60 | 0.60 |
| | 0.50 | 2 | 0.20 | 0.20 | 0.20 | 0.40 | 0.40 | 0.40 | 0.60 | 0.60 | 0.60 |
| | | 3 | 0.20 | 0.20 | 0.20 | 0.40 | 0.40 | 0.40 | 0.60 | 0.60 | 0.60 |
| | | 4 | 0.20 | 0.20 | 0.20 | 0.40 | 0.40 | 0.40 | 0.60 | 0.60 | 0.60 |
| | 0.75 | 2 | 0.20 | 0.20 | 0.20 | 0.40 | 0.40 | 0.40 | 0.60 | 0.60 | 0.60 |
| | | 3 | 0.20 | 0.20 | 0.20 | 0.40 | 0.40 | 0.40 | 0.60 | 0.60 | 0.60 |
| | | 4 | 0.20 | 0.20 | 0.20 | 0.40 | 0.40 | 0.40 | 0.60 | 0.60 | 0.60 |

Table S129: Mean of $\hat{\rho}$ when the alternative hypothesis is true, and all treatments are effective.

| | | | $\rho = 0.2$ | | | $\rho = 0.4$ | | | $\rho = 0.6$ | | |
|---|---|---|---|---|---|---|---|---|---|---|---|
| $N$ | $\tau$ | $G$ | MLE | CMAE | UMVCUE | MLE | CMAE | UMVCUE | MLE | CMAE | UMVCUE |
| 50 | 0.25 | 2 | 0.19 | 0.19 | 0.19 | 0.39 | 0.39 | 0.39 | 0.59 | 0.60 | 0.60 |
| | | 3 | 0.19 | 0.20 | 0.20 | 0.39 | 0.39 | 0.40 | 0.59 | 0.59 | 0.59 |
| | | 4 | 0.19 | 0.20 | 0.20 | 0.38 | 0.39 | 0.39 | 0.58 | 0.59 | 0.60 |
| | 0.50 | 2 | 0.19 | 0.19 | 0.20 | 0.38 | 0.39 | 0.39 | 0.59 | 0.59 | 0.59 |
| | | 3 | 0.19 | 0.19 | 0.19 | 0.38 | 0.39 | 0.39 | 0.58 | 0.59 | 0.59 |
| | | 4 | 0.19 | 0.19 | 0.20 | 0.38 | 0.39 | 0.39 | 0.58 | 0.59 | 0.59 |
| | 0.75 | 2 | 0.19 | 0.19 | 0.19 | 0.39 | 0.39 | 0.39 | 0.58 | 0.59 | 0.59 |
| | | 3 | 0.19 | 0.19 | 0.20 | 0.38 | 0.39 | 0.39 | 0.58 | 0.59 | 0.59 |
| | | 4 | 0.18 | 0.19 | 0.19 | 0.38 | 0.39 | 0.39 | 0.57 | 0.58 | 0.58 |
| 200 | 0.25 | 2 | 0.20 | 0.20 | 0.20 | 0.40 | 0.40 | 0.40 | 0.60 | 0.60 | 0.60 |
| | | 3 | 0.20 | 0.20 | 0.20 | 0.40 | 0.40 | 0.40 | 0.60 | 0.60 | 0.60 |
| | | 4 | 0.20 | 0.20 | 0.20 | 0.40 | 0.40 | 0.40 | 0.60 | 0.60 | 0.60 |
| | 0.50 | 2 | 0.20 | 0.20 | 0.20 | 0.40 | 0.40 | 0.40 | 0.60 | 0.60 | 0.60 |
| | | 3 | 0.20 | 0.20 | 0.20 | 0.40 | 0.40 | 0.40 | 0.60 | 0.60 | 0.60 |
| | | 4 | 0.20 | 0.20 | 0.20 | 0.40 | 0.40 | 0.40 | 0.60 | 0.60 | 0.60 |
| | 0.75 | 2 | 0.20 | 0.20 | 0.20 | 0.40 | 0.40 | 0.40 | 0.60 | 0.60 | 0.60 |
| | | 3 | 0.20 | 0.20 | 0.20 | 0.39 | 0.40 | 0.40 | 0.60 | 0.60 | 0.60 |
| | | 4 | 0.20 | 0.20 | 0.20 | 0.40 | 0.40 | 0.40 | 0.59 | 0.60 | 0.60 |
